# Attitudes towards Addressing Medical Absenteeism of Students: A Qualitative Study among Principals and Special Education Needs Coordinators in Dutch Secondary Schools

**DOI:** 10.1371/journal.pone.0148427

**Published:** 2016-02-04

**Authors:** Yvonne Vanneste, Marlou van de Loo, Frans Feron, Carin Rots – de Vries, Ien van de Goor

**Affiliations:** 1 Department of Youth Health Care, Regional Public Health Service West Brabant, Tilburg, The Netherlands; 2 Department of Social Medicine, Maastricht University, Maastricht, The Netherlands; 3 School of Social and Behavioural Sciences, Tilburg University, Tilburg, The Netherlands; TNO, NETHERLANDS

## Abstract

**Background:**

Reducing school absenteeism benefits the health and educational opportunities of young people. The Dutch intervention Medical Advice for Sick-reported Students (abbreviated as MASS) was developed to address school absenteeism due to sickness reporting, also called medical absenteeism. This study is part of a research project on the effectiveness of MASS and explores factors that influence the implementation and dissemination of the intervention, from schools’ perspectives. The research questions include reasons schools have to implement MASS, their experiences in the implementation of MASS and their views on what is needed to ensure sustainable implementation.

**Methods:**

A qualitative research method was used. Semi-structured interviews were held with nine principals and eight special education needs coordinators, working in nine secondary schools that apply MASS. Inductive content analysis was carried out.

**Findings:**

The main reasons for schools to address medical absenteeism were their concerns about students’ well-being and future prospects and their wish to share these concerns with students’ parents. Participants also mentioned the wish to raise the threshold for reporting sick. According to the participants, MASS makes it easier for teachers to enter into conversation with students and their parents about medical absence. MASS prevents damage to the relationship with parents and medical problems being missed. In implementing MASS the main obstacles are teachers’ dialogue about medical absence with students and their parents, teachers’ follow-up of the feedback of the youth health care physicians (YHCPs), and correct registration. The participants were convinced that MASS also improves collaboration with parents regarding the optimization of care for students.

**Conclusions:**

MASS allows schools to identify students at risk of dropout at an early stage and to optimise guidance of these students. The intervention matches schools’ need to address medical absenteeism by providing a clear framework, an approach from concern rather than control, and socio-medical expertise through the collaboration with YHCPs. MASS can support schools to maximize the number of students graduating and to improve parental involvement in school. These outcomes may help to put the subject of addressing medical absenteeism on the agenda of all schools, and contribute to prioritization, support adoption and secure sustainable implementation and dissemination of MASS.

## Introduction

**Fig 1:**
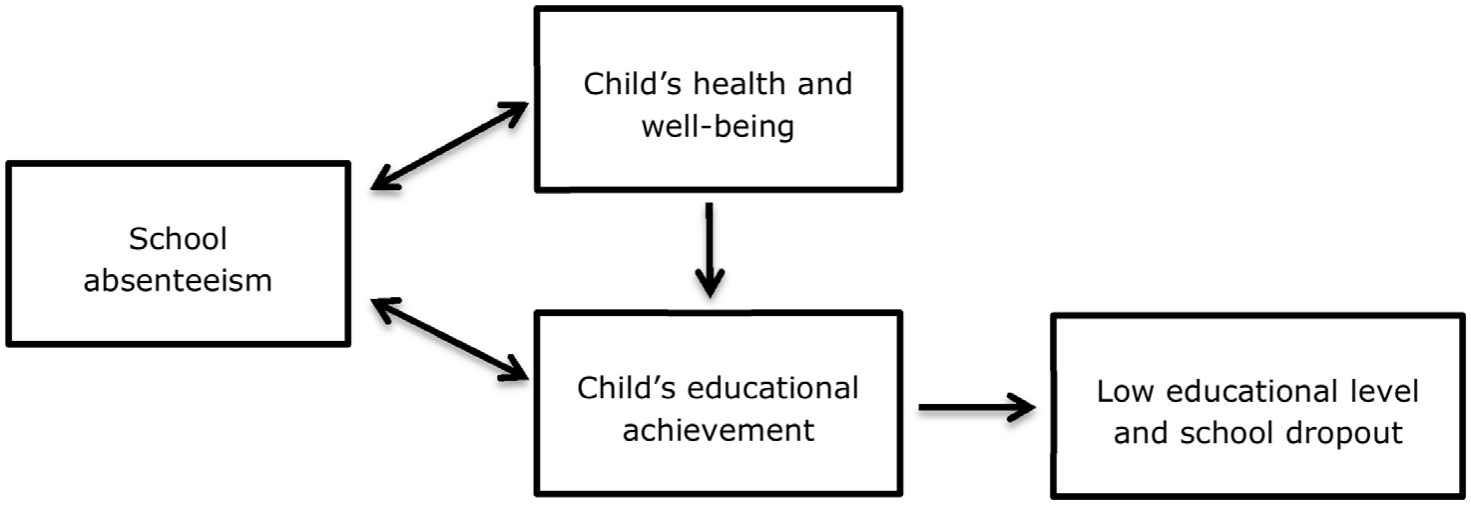
Visualisation of the interrelationship between school absenteeism, young people’s development and low educational level and school dropout.

Substantial school absenteeism may negatively affect children’s social and emotional development, and can cause children’s educational development to stagnate, which, in turn, may lead to moving down to a lower level of education or even early school dropout [1–8]. The impact of school absenteeism on educational achievement can partly be explained by the fact that the moment school absenteeism is caused by health-related issues, which is the case in medical absenteeism, these impaired health conditions can also affect educational outcomes [9–13]. See Fig 1 for the visualisation of the interrelationship between school absenteeism, young people’s development and low educational level and school dropout.

In the Netherlands, half of school absenteeism is medical absenteeism, that is school absenteeism resulting from parental sickness reporting of the child [14]. Schools are confronted daily with medical absenteeism. It is school’s authority to decide how to deal with it and as we know from everyday practice, medical absenteeism is not adequately addressed. Therefore, the intervention ‘Medical Advice for Sick-reported Students’ (abbreviated as MASS; see Box 1) has been developed by the preventive youth health care department (YHC) [15,16] of the Dutch Regional Public Health Service West Brabant, in collaboration with secondary schools and the municipal school attendance service for compulsory education in West Brabant. Since 2010, MASS has been applied at sixteen secondary schools in the West Brabant region. After piloting [17], all these schools are currently involved in a study to collect data on the intervention’s effectiveness. For the purpose of this study, two different routes of the MASS intervention were implemented.

**Box 1. Description of the Dutch intervention ‘Medical Advice for Sick-reported Students’, abbreviated as MASS.**

The MASS intervention consists of an integrated approach in a public health setting. MASS provides a clear framework in which schools, in direct collaboration with youth health care physicians (YHCPs), are able to reach students and their parents, discuss aspects of the student’s medical absence, and design and monitor a management plan that aims to optimize students’ health and maximize students’ participation in school activities. In summary, the aim of the MASS intervention is to limit the absenteeism by arranging appropriate care, educational adjustments and adequate support for students and parents. A systematic routine is followed.

**Step 1 School’s policy:**

The school communicates with students and parents about the new policy in case of absenteeism because of medical reasons.

**Step 2 Referral to the YHCP:**

Schools actively identify students with extensive medical absence, and can choose between two different routes. The first route has well-defined threshold criteria for referring to the YHCP: four times reported sick in 12 school weeks or more than six consecutive school days (MASS-criteria). Meeting the criteria always leads to a referral to the YHCP for student and parents. In the second route, the school decides if and when to confront students and their parents in cases of absence reported as sickness. This dialogue may lead to consultation with the YHCP. In both routes the consultation is compulsory. If the appointment is missed, the medical absence is registered as ‘not excused’ and is reported to the school attendance officer, who will undertake further action.

**Step 3 Consultation of student and parents with the YHCP:**

During the interview and medical assessment YHCPs look for biological, psychological and social factors that contribute to the students’ medical absenteeism. The YHCP identifies whether there is a specific somatic or psychiatric diagnosis to account for the absence. If the diagnosis is clear the focus will be on optimising the (adherence to) treatment. In cases of frequent physical complaints and psychosocial problems with no clear medical diagnosis, the YHCP considers diagnostics, and looks for family and school related factors, as well as health risk behaviours and lifestyle aspects that contribute to the physical complaints and psychosocial problems. If needed, the YHCP refers to a medical specialist or a psychosocial support network. A management plan is then designed together with student, parents and school, and with curative professionals, if applicable. This plan includes agreements on cure, care and school attendance.

**Step 4 Monitoring the management plan:**

School and YHCP monitor the execution of the management plan.

The impact of an intervention is determined not only by its effectiveness but also by its dissemination, allowing widespread adoption and implementation beyond the region of origin [18–21]. The implementation and dissemination of an intervention can be improved by providing insights into motives for its adoption and into preconditions for implementation, and by ensuring that the intervention matches the needs [22–24]. Schools and preventive youth health care services are both key adopters and users of MASS. Because youth health care physicians’ (YHCPs) main objective is to arrange adequate care for students with substantial medical absenteeism they should be motivated to carry out MASS. In contrast, school staff are not legally obliged to address medical absenteeism and paying attention to medical absenteeism is at best a tool for helping students towards graduation. Consequently, this study focused on the attitudes of school staff towards addressing medical absenteeism. To gain insight into implementation aspects it is clearly necessary to consult school staff who are already using MASS. By asking principals, who are key players regarding school policy and decision making, and special education needs coordinators (SENCO; see Box 2) who are key players regarding the actual implementation, you gain insight into different and complementary views on adopting and implementing an intervention [19].

**Box 2. Description of the special education needs coordinator (SENCO).**

In the Netherlands, special education needs coordinators are responsible, in contact with external experts, for arranging the care of students with special needs, both within and outside the school. They coordinate the care policy of the school, and advice and coach colleagues how to act in the best interests of students that need extra care. In school, they are responsible for the implementation of policy changes that are related to the care of students.

The research questions that guided the study included: (1) what reasons motivate school staff to pay attention to extensive medical absenteeism? (2) What are their experiences in applying MASS? (3) What are facilitators and obstacles for sustainable implementation? The first question provides insight into how the intervention should be brought to the attention of schools so as to promote adoption. The second question provides insight into how the intervention matches the needs, and into preconditions for improving implementation. The third question provides insight into the need for sustainable implementation and widespread use.

## Methods

### Setting

This qualitative study is part of a practice-driven intervention study, which aims to evaluate the MASS intervention in pre-vocational secondary schools: by investigating the health condition of pre-vocational secondary students with extensive medical absence, the effectiveness of the intervention, and implementation and dissemination issues.

### Study participants

Participants were principals and SENCO’s of schools applying the MASS intervention. The schools varied in educational level, school size, the period of time from when they were implementing MASS and in using the first or second route (see Box 1 for an explanation of the routes). They were recruited using purposive sampling. All those invited agreed to be interviewed. Participants were added until data saturation was reached. The participants and school’s characteristics are presented in Table 1.

**Table 1:** Overview of participants and school’s characteristics.

Participants*	School characteristics			
	Educational levels	School size**	MASS***	Route
P_1_ and S_1_	Pre-vocational secondary education	Medium-sized	> 1 year	First
P_2_ and S_2_	Pre-vocational secondary education	Medium-sized	> 1 year	First
P_3_ and S_3_	Pre-vocational secondary education	Large	> 1 year	First
P_4_ and S_4_	Pre-vocational secondary education, senior general secondary education and pre-university education	Large	< 1 year	First
P_5_ and S_5_	Pre-vocational secondary education	Small	< 1 year	First
P_6_ and S_6_	Pre-vocational secondary education, senior general secondary education and pre-university education	Large	< 1 year	Second
P_7_ and S_7_	Pre-vocational secondary education	Medium-sized	< 1 year	Second
P_8_	Pre-vocational secondary education	Small	< 1 year	Second
P_9_ and S_9_	Pre-vocational secondary education	Small	> 1 year	Second

* P = Principal, S = SENCO, number refers to school.

** Size of the school: small (<250 students), medium-sized (250–750 students) and large (>750 students).

*** Period of time from when MASS has been implemented.

### Ethical considerations

The study, including consent procedure, was approved by the Medical Ethics Committee of the University Hospital Maastricht and Maastricht University (Dossier number 11-4-070.6/ivb). Participants were informed by letter, and again at the start of the interview, about the purpose of the research, the anonymously processed data-analysis, and the use of the information received, exclusively for research and in which confidentiality was guaranteed. They were made aware that they were free to refuse to participate at any time before, during, and after the interview. They were given a 3 day reflection period before an appointment was made. The interview was only initiated upon receipt of written informed consent at the beginning of the interview.

### Data collection

In order to gain insight into schools’ beliefs and perceptions, in-depth interviews were carried out. Semi-structured interviews were conducted face-to-face by two researchers. The topic guide originated from earlier experiences of the investigators [17] and from theoretical grounds derived from the literature [19]. The questions were broad, open-ended, and non-directive. This led to a detailed exploration of participants’ views and experiences with regard to the intervention. Topics put forward were reasons for paying attention to medical absenteeism, possible explanations for medical absenteeism, the possible added value of a YHCP in this intervention, opinions about and experiences with the intervention, and factors influencing the implementation of the intervention. The audio-recorded interviews lasted 45 to 90 minutes and were transcribed verbatim for analysis. Field notes taken during the interviews, for example about the atmosphere and body language, were added to the transcripts. After the first seven interviews there was an intermediate analysis to check whether all topics had been covered. Additional schools were selected until no new concepts were identified and the point of data saturation [25] was occurred. Finally, from December 2011 to February 2012, 9 principals and 8 SENCO’s were interviewed, in five of the eight schools implementing the first route and in four of the eight schools implementing the second route of the intervention (see Box 1 for an explanation of the routes).

### Data Analysis

The two researchers, independently from each other, read and open-coded the first seven transcripts and created categories, based on text fragments and the topic guide (see Box 3) being used, resulting in a code-book. Next, categories were created within the bounds of Rogers’ theoretical frameworks, leading to an inductive content analysis [26]. All transcripts were coded again by one of the researchers, using MAXQDA 10 software [27], with the other researcher reading and assessing the classified codes. Different interpretations of codes were discussed in the research team and refined until agreement was reached. All data (transcripts, coding tree, and findings) were available for inspection by the co-authors during the research, and are still available to other interested parties. In addition, we did a member check by asking the participants if the findings matched their experience. A multidisciplinary research team consisting of an epidemiologist, a pedagogue, a physician and a health scientist was asked whether they considered the findings to be clear, understandable, and logical.

**Box 3. Topic Guide.**

**Introduction**

Researchers: explanation about the goals of the interview and the handling of the received data (anonymous).

Participants: occupation, qualifications, background information, characteristics of the school concerned.

**Motives for the school to address medical absenteeism**

How was the decision-process at school for applying MASS?

Medical school absenteeism;

What are schools’ reasons to pay attention to student’s medical absenteeism?

The situation as it stands;

What is in their opinion the magnitude of the problem?

And what differences do they experience or see between educational levels or school years?

What do you experience as alarming signs?

What are their previous experiences with addressing sick reports?

Responsibility of school to pay attention to this group of students;

What are the tasks and/or roles of school?

Who’s the problem owner?

**Collaboration with youth health care**

Motives to collaborate with a health care organization

What do they know about youth health care?

What are their previous experiences regarding collaboration?

What do they know or experience about the division of tasks between school and the health care organization?

Expectations towards youth health care physician;

What do they expect to be or see as an added value of the youth health care physician?

What possible outcomes of the consultation and feedback towards school do they expect or wish?

**Thoughts and experiences about MASS**

What are their expectations (goals, relative advantages)?

What reasons do they have or are there to choose between route 1 or 2?

**Implementation process**

Running intervention;

Are you satisfied about the intervention? Why yes/no?

How are the conversations with students and parents experienced?

What can you tell about the reactions students and parents?

What can you tell about the experiences within the school

What can you tell about the compatibility of MASS?

Practical issues

How are the tasks divided within the school with regard to dealing with student’s medical absence and the execution of MASS?

How do you think about the finances? Who has to pay?

What facilitators can you mention?

What barriers can you mention?

What has to be done to implement MASS successfully in the long-term?

**Closing**

Is there anything not covered?

Would you advise MASS to other principals or SENCO’s? And if yes/ no, why?

Thank you for your time and participation.

## Findings

The findings are described in the order in which the research questions were posed.

### Reasons for paying attention to extensive medical absenteeism

#### Concerns about the negative impact of school absenteeism on the student

The reasons most often mentioned for paying attention to medical absenteeism were those related to the negative impact of absenteeism on the students. First, the participants expressed concerns about students’ educational achievements. Students miss course material and, as a result, their school performance can decrease, which increases the risk for drifting to a lower educational level and dropout. Moreover, since they have fallen behind, these students need to catch up. Participants observed that students with extensive medical absence often already have problems with structuring their school work and keeping motivated for school. Therefore, these students cannot be left with full responsibility for catching up on missed lessons and assignments. This causes a lot of extra work for teachers. The participants also had concerns about a student’s well-being, because absence means a lack of the social contacts, structure, and regularity, normally provided by school attendance. Consequently, students can go astray, which not only decreases school performance but also affects their socio-emotional development.

*“Students who are often sick miss out on subject matter*. *Their performance declines*. *They also lose out on social aspects and miss a lot of things that happen in class*. *Some of our students already struggle to keep up and to structure their school work*, *and if they are often sick*, *it becomes even more difficult*. *Teachers have to deal with that*. *Therefore*, *absence always has significant consequences for both students and their teachers*.*” S*_*2a*_

#### The wish to share concerns and responsibility with parents

Secondly, participants mentioned the wish to share their concerns with students’ parents. They were convinced that a child’s education and well-being should be more the collective concern and responsibility of both parents and school than is experienced currently. This applies particularly to medical absence, because they presumed that only some of the students really are sick.

*“Well*, *it is the parents who report their child sick*, *in any case*. *I consider it important to share care with the parents when a child is not doing well*.*” S*_*2b*_

The participants assumed that there is a wide range of ‘hidden’ reasons for reporting sick. They spontaneously mentioned domestic problems first. Problems they mentioned were: lack of stability, needed at home, and a disturbed relationship between parents and children. They supposed that nowadays there is an increase in domestic problems and broken families, and a lack of structure, safety, and control.

*“Frequent medical absenteeism has everything to do with the situation a student finds himself in*. *I feel that the home situation is a dominant factor here*.*” P*_*8a*_

When probed further, they mentioned problems at school such as bullying, a study pressure that is too high, a boring school system, difficulties with learning, problems with a teacher. Then child-related problems were mentioned: lifestyle problems such as going out in the weekend, busy with social media and going to bed too late, psychological problems, and social pressure. The participants noted that they have gone too far in caregiving for students, and that parental involvement in school matters is generally too low.

*“The student can feel bothered*, *be bullied*.*” S*_*6a*_

*“We also have a lot of trouble with students who are consistently sick on Monday morning; These are students who go out at weekends and party so hard that they are in no state to come to school*.*” S*_*7a*_

*“In our society*, *parents are increasingly pushing the responsibility for the students’ development onto school*, *and that is a completely wrong development*. *It is our ambition to increasingly support parents and involve them in the school*. *Parents are*, *logically*, *inextricably bound up with the development of their children*, *and the school can support that*, *but the school has different responsibilities*.*” P*_*3a*_

#### Schools’ wish to raise the threshold for reporting sick

The third reason was the wish to raise the threshold for reporting sick, which arose from the participants’ assumption that students are reported sick too easily and, with the approval of the parents, stay home for the least excuse. They noted that it is an obligation not only of the school but also of both parents and children to ensure school attendance. Some participants even mentioned that reporting sick too easily can result in negative future work ethics.

*“It is a slippery slope; when they realise they can easily report themselves sick*, *next time it will be a smaller step*.*” S*_*4a*_

*“It contributes to the changing attitude to standards throughout society; that it matters*, *that you have rights and duties*, *and that if you are still of school age you have to go to school*.*” P*_*4a*_

Moreover, they noted that the presumption of unjustified sick reporting can also result in the teachers developing a negative image of the child. They mentioned that feeling responsible and not being able to share (with parents), in combination with all the extra work involved in helping students to catch up and in supporting them to prevent dropout, causes a lot of frustration for teachers.

*“At any moment we interpret students’ behaviour; and we draw our conclusions*. *And these conclusions*, *this image*, *will only become stronger*. *Because we mostly see what we want to see—that fits the picture we have previously built up*. *You can call it ‘tunnel vision’*. *And*, *once an image has been formed that a student is ‘faking it’*, *it will be hard to break it*.*” P*_*8b*_

### Experiences in applying MASS

#### Problems experienced with students’ medical absenteeism before MASS

At the beginning of the interviews the participants were asked to describe the problems they experienced with medical absenteeism before MASS was implemented. For understanding, it is important to realise that in the Netherlands it is not possible to get sickness reports verified by an appropriately licensed medical professional. When trying to reach parents and students to discuss medical absence, the following problems were experienced: to begin with, for each individual teacher there were different considerations that influenced their decision on whether or not to contact parents and students in response to sick reporting. A shared and objective approach was lacking. And anyway, confronting students and parents was often experienced as unpleasant and difficult. This was caused by doubts about the right tone and content of the dialogue, and was related to the personality, social skills, and frame of reference of the teacher. They feared damage to their relationship with the parents.

*“Yes*, *one teacher might be ‘bolder’ or ‘more forward’ than another*. *And that is the case for all duties one has of course*. *It is like that at every work place; one person takes his duties very seriously and gets right down to it*.*” S*_*2c*_

*“I doubt whether those conversations would be held properly*. *Because you can have a conversation in many different ways*, *and one person will deal with it more easily than another*.*” S*_*4b*_

*“I think that some teachers are scared at that moment*, *like ‘now I have to have a horrible conversation*, *because you as a parent report your child as sick too easily*, *or at least we feel that way’*. *So I think that some teachers then show avoidance behaviour themselves (laughs)*. *I’d better not deal with this now’*. *‘I will just let it slip*, *and not go into it too much*, *that way the conversation will not become too difficult*.*” S*_*2d*_

They also feared that they might overlook something important because of their limited medical knowledge and emphasised that these fears often causes a delay in entering into conversation with students and their parents about the medical absence.

*“I do not want to step into the physician’s shoes*.

*Perhaps*, *with my limited medical knowledge*, *I would overlook something which a physician would not*.*” P*_*2a*_

When confronted, a number of parents thought that it was none of the schools’ business. Parents did not want to share their personal affairs. And when parents were willing to share information, the information was often perceived as subjective and unreliable, and when parents did refer the schools to their general practitioner (GP), allowing the GP to provide information after explicit written consent, the information received was often not relevant, because the information did not contain advices about necessary educational adjustments or supervision of the student by the school.

*“People are so quick to go on the defensive*, *even when no attack is intended*.*” S*_*1a*_

*“Information via the parents is often very biased and rather unreliable*.*” S*_*7b*_

“*I assume the parents think that their personal affairs are being interfered with*, *and that they are being accused of incompetence*, *as it were*.” *P*_*1a*_

*“Well*, *if I call the GP*, *I never receive an answer*. *And that is how it ought to be*, *I think*. *But that is not for us to do*, *as a school*.*” S*_*9a*_

*“We are seldom any wiser after speaking to a GP*, *if we get any information at all*.*” S*_*7c*_

#### Experienced advantages of MASS

The participants mentioned that the prime advantage of MASS for the teachers could be the approach in general. The approach provides a framework for talking about medical absence by providing clear criteria and the emphasis on the dialogue being conducted for reasons of concern and not from a wish to control the absence and to enforce compulsory education. This lowers the threshold for entering into dialogue by simplifying the contact with parents, because now teachers can say: ‘This is just how MASS works.’ When choosing the first route, that is, the use of fixed criteria for referral to the YHCP, it is not even necessary to argue aspects or judge reasons for medical absenteeism. It is easy to explain to parents that all students that meet the criteria will be invited to see a YHCP. When choosing the second route, that is, teachers themselves confronting students and parents about medical absence initially, participants mention that having the dialogue can strengthen the relationship between school and parents.

*“It is now very easy for me to explain to parents ‘this applies to everyone’*, *‘everyone just has to do this and we are not doctors*.*” S*_*2e*_

*“If parents were to go to the YHCP straightaway*, *they would miss the connection with the school*, *that the school cares*. *It is a conversation based on concerns*. *Other matters will arise from it*. *Because the student can feel bothered*, *be bullied*, *a student can eh…*, *and if these matters turn out to be the cause of the absenteeism*, *teachers can take them up themselves*.*” S*_*6b*_

*‘‘If the communication with parents is very clear from the beginning*, *they will see it as an extra aid*, *and not as an evil means of control*.*” S*_*5a*_

Moreover, some issues brought up by students and parents can be taken up immediately by the school itself, for example bullying or a problematic relationship with teachers. The participants noticed that the approach also provides the possibility of identifying children at risk at an early stage and arranging care. This early identification is needed because participants are convinced that the stress on the student caused by having to catch up can lead to further medical complaints and absence, and prolonged absence can, in turn, become a deterrent to school attendance. Both situations can lead to a vicious circle of increased school absence, making it increasingly difficult to support the student adequately.

*“I am convinced that a student in a family situation where parents are getting divorced or where one of the parents has passed away can suffer from the situation and develop symptoms that we can identify with MASS at an early stage*.*” P*_*2b*_

*“Whenever the student went to school*, *there would be ten teachers grabbing him to tell him to ‘catch up with this and hand in that’*, *and the student wouldn’t be able to figure out how to manage everything*. *The stress might then become too much*, *resulting in more absence*.*” S*_*1b*_

Secondly, the collaboration with YHCPs (as against GPs) was experienced as an advantage. The participants described YHCPs as being objective medical experts who act independently and have a holistic view of students, who also check whether there are other important issues that influence the absence. Parents (because of the professional contacts of the YHCPs within the health care sector): experience their involvement as ‘formal’ and feel that complaints are taken seriously; are discouraged from distorting the facts; are given insight into symptoms that make it necessary for students to stay at home; and, if necessary, are given more directive and confrontational support. In addition to this, YHCPs can give medical advice. Participants observed that teachers find clear feedback from YHCPs (such as guidelines on participation, hints on supporting students in school, confirmation of support that has already been started, and possible agreements on coping with the students’ problems) to be really useful.

*“If an independent third party*, *a qualified doctor*, *with a certain status in the eyes of the parents’*, *gives his opinion*, *this is received differently from when an educational institution does so*.*” P*_*2c*_

*“And because of MASS we shall be able to refer a student for more care*, *ensuring that the student will not end up in mental distress*. *School can provide this student with support*, *but not the medical support he might need*.*” P*_*9a*_

*“It is just the fact that somebody with a medical background confirms that what is happening is correct*. *That is essential*. *But it also helps when somebody with a medical background ascertains that a student has been kept home for a long time unjustly and asks the parents*: *‘Why did you keep him home that long*?*’ It is not even about blame*, *but it is a matter of making people aware of the situation*.*” S*_*5b*_

*“A YHCP discusses these subjects differently from a GP*.*” S*_*5c*_

*“The feedback received from the YHCP gives you something to get hold of*.*” S*_*3a*_

*“Since MASS*, *the connections are short and are really being made*. *That is a substantial difference with how it used to be*.*” S*_*5d*_

Finally, according to the participants, another advantage was the acquisition of a better understanding of what influences a student’s well-being and what a certain disease or circumstance means for a student’s functioning. A majority of the participants found that these insights help them to react properly to problems and causes, and increases the engagement with students, which leads to better support and school progress.

*“Firstly*, *I think there could be much more understanding*, *not just at an emotional level*, *but literal understanding*, *about situations*. *Knowledge about what goes on in students’ lives and what reasons there can be for not attending school*. *Students and teachers can learn how to deal with absenteeism more satisfactorily and*, *eventually*, *it decreases*.*” P*_*7a*_

*“Absenteeism*, *home situation*, *and school situation are all aspects of a school career*. *By monitoring medical absenteeism*, *giving good feedback*, *also to parents*, *I think you gain a better perspective of the well-being of a student*, *at school as well as at home*, *from which study results and definitely school development subsequently benefit*.*” P*_*3b*_

### Facilitators and obstacles for sustainable implementation

#### Communication and dialogue about medical absenteeism

Questions about facilitators and obstacles, show up the challenges to a successful implementation. Participants who were implementing the second route unanimously mentioned that the main challenge is the questioning by teachers of students and their parents about the context of medical absence. Participants agreed that this dialogue is the trickiest and most difficult part of the intervention, as the course of the dialogue is of vital importance for the results of the intervention. Some suggested a need for training in asking the right questions and how to interpret the answers.

*“If the communication with parents is very clear from the beginning*, *and they see it as an extra aid and not as an evil means of control*, *you avoid angry phone calls from parents saying ‘What are you doing with my child*?*” S*_*5e*_

*“Discussing sickness absence often provokes resistance from parents*. *That makes it hard*.*” S*_*2f*_

*“I think that talking to parents about medical absenteeism can be learned*.*” S*_*3b*_

#### Follow-up of the feedback

Secondly, follow-up of the feedback from the YHCP was experienced as most difficult when implementing the first route, especially in cases of persistent absenteeism without appropriate reasons. In those cases, enforcement and reporting to the school attendance officer is needed. This also requires correct registration, which is a third challenge, as mentioned by the participants.

*“The downside of the first route would be that our school would not develop its own expertise and learn to handle contacts about absenteeism*. *I see that teachers find it difficult to discuss and perform the advice of the YHCP*.*” P*_*7b*_

*“Absenteeism registration has to be accurate*, *throughout the time at school*. *Otherwise*, *if the student is referred to the school attendance officer and the case goes to court*, *a judge can say ‘Get lost*, *do your homework first and then we shall see*.*’ If you can't refute the contention that a student has attended school*, *some students and parents will feel victorious*.*” P*_*1b*_

#### Full support of all involved

Finally, it is a challenge to achieve the full support of all involved. Commitment is required at all organizational levels within school. And all professionals need to collaborate appropriately on matters that are mostly delicate and confidential.

*“Parents are playing us off against each other*. *Those are matters we have to deal with together*.*” P*_*2d*_

#### Expectations of intervention outcomes

When questioning factors that can influence the sustainability of MASS, principals noted that observable positive effects will stimulate all those involved to invest the time needed. In response to questions about observable effects, participants unanimously mention an increase in awareness in school of the level of medical absenteeism, which is often more extensive than expected. Parents are also made aware: some of them do not even realise that absence is this high; others do not appreciate the consequences of reporting sick or do not realise that children need to be stimulated to go to school. A noticeable decrease in medical absence as a result of greater attention, control, and guidance is mentioned repeatedly.

*“We have to feel that it brings results*.*” S*_*1c*_

*“The number of sickness reports we receive has been mapped by MASS*, *and that has opened our eyes*. *We had been blind before*, *because the number was huge*! *We were so alarmed by it that we now treat it with greater emphasis*.*” P*_*2e*_

*“The effect of it might be in the fact that the parents now also become aware of the frequency with which they report their children sick*.*” S*_*5f*_

*“I am convinced that absenteeism has decreased since the application of MASS*.*” P*_*4b*_

Exaggerated expectations of intervention outcomes that are then not realised in the short-term can be an obstacle to the sustainability of MASS. Expected long-term effects are the saving of time and investment resulting from decreased absenteeism, which leaves more time for their ‘core-business’, a change in attitude towards reporting sick among students and parents, and a decrease of school dropout. Participants also noted that, realistically, school dropout cannot be solved with only this intervention. They noted that MASS can be seen as an investment in that specific vulnerable group of students, where many risk factors play a role.

“*Because when it comes to premature dropping out*, *there are so many factors of influence*. *Perhaps MASS contributes greatly to decreasing dropouts*, *or less sickness reports at work*, *but you will find out only in a couple of years’ time*.*” P*_*8c*_

*“Perhaps MASS contributes greatly to decreasing dropouts*, *or less sickness reports at work*, *but you will find out only in a couple of years’ time*.*” P*_*3c*_

#### Agenda setting

Furthermore, participants raised that addressing medical absenteeism is not an issue on schools’ agendas, which is needed to secure sustainability. However, their experience was that MASS can be a tool for improving parental involvement and collaboration between all involved, and that MASS enables preventive action. This can result in better quality of care for students. They noted that all these advantages contribute positively to achieving their own direct goal of helping as many students as possible to graduate.

*“Eventually*, *we are judged on graduation rates*.*” P*_*5a*_

*“Paying attention to medical absenteeism is not on our agenda*. *However*, *now that I think about it*, *increasing parental involvement in school is on schools’ agendas and has a high priority*.*” P*_*6a*_

*“And I see that MASS can help us to achieve this*.*” P*_*3d*_

Finally, funding is also required to finance the assessments and consultations by YHCPs. If the dialogues with students and parents are allocated to school employees, time and energy need to be invested in creating a strategy and in carrying out these dialogues. MASS operates on the border between school and health care. Many participants therefore saw difficulties in the structural financing of this intervention.

## Discussion

This study highlights schools’ attitudes towards addressing medical absenteeism, in particular the use of MASS, with the aim of optimising its implementation and dissemination. Principals and SENCO’s from nine schools where the intervention was implemented were interviewed.

Regarding reasons for paying attention to medical absenteeism our findings show that schools’ initial reason is their wish to support students with extensive medical absenteeism adequately, to start by reducing their absence rate because of its negative impact on the student. According to the participants this should be done by handling the home situation and by raising the threshold for reporting sick in the first place. Participants assume that only some of the students really are sick and are convinced that student’s medical absence often is caused by domestic problems and by parents reporting sick too easily. Teachers want to share not only their concerns but also the responsibility for child’s well-being with the parents adequately. They notice that parents make the school too much responsible for the child’s development. The participants perceive that teachers do not feel comfortable to talk with students and their parents about extensive medical absence. This may be caused by their wish to control and raise the threshold for sickness reporting. Furthermore, teachers have serious doubts whether students and parents tell the truth about the underlying reasons for the absence. By entering a conversation from this perspective there is a risk of damaging the relationship with students and parents. Starting the conversation from a shared concern rather than control however, will help teachers to discuss the student’s absence adequately. Adequate communication about this perspective and the shared responsibility for the child’s development seems to be a prerequisite for implementation of MASS.

Regarding schools’ experiences in applying MASS our findings show that the clear and structured approach in general and the collaboration with YHCPs are the main advantages of MASS. These benefits address the problems that schools experience (when willing to handle medical absenteeism). The objective approach helps them to reach parents and students, and to share concerns and responsibility. The support by YHCPs prevents them from missing medical problems. Moreover, it helps them to gain insights into a child’s functioning and situation. A better understanding prevents teachers from developing a negative image of a child, and helps them to provide adequate support. A vicious circle of increased absence can then be prevented. It is known from the literature that many cases of sickness can be prevented by changing underlying behaviour or by supporting the psychosocial support system, and that not all illnesses make it necessary to stay at home [28]. Therefore, MASS makes it possible to identify students who are at risk of dropout and to prevent them from dropout proactively, as noticed by the participants.

Regarding the facilitators and obstacles for sustainable implementation our findings show that the participants think the main challenges to teachers for successful implementation are the dialogue with the parents about the context of medical absence and the follow-up of the feedback from the YHCP. When using fixed criteria for referral to the YHCP there is no need for teachers to discuss the absence with students and parents themselves. However, the follow-up is experienced as difficult. When teachers themselves discuss the absence with students and parents initially, it makes it easier to follow-up of the feedback because, from the start, teachers are more actively involved Moreover, as shown in our study on students’ health condition [29], extensive medical absenteeism may also be caused by problems in school. This is an additional reason to have a dialogue by school before referring to the YHCP. It is conceivable that, when it is clear that the purpose of the approach is the promotion of a student’s well-being and educational chances, parents’ resistance to discussing a student’s medical absence will be reduced. And if it is a schools’ general policy, parents and students will get used to it. You can then expect that the dialogue will become easier and less troublesome. In addition, teachers can be trained.

This study also highlights the fact that medical absenteeism is not an issue on schools’ agendas. However, the participants feel that MASS can optimise educational outcomes and prevent school dropout, not only through improving the quality of care for students but also through improving parental involvement in school. Improving parental involvement is part of a schools’ agenda, because it strongly impacts on students’ achievements and learning [30, 31], and therefore helps students to graduate. MASS can thus contribute to schools’ primary objectives. This should be considered when bringing MASS to schools’ attention and in motivating them to adopt it.

The greatest challenge at organizational level for a successful implementation of MASS may be getting the full support of all those involved [23]. For this, adequate communication about the results to be expected, is needed [18, 19]. The short term effects are not only increased awareness of medical absenteeism and increased parental involvement, as noticed by the participants, but also better understanding of what influences a student’s well-being and what a certain disease or circumstance means for a student’s functioning. Visible effects could be a decrease in medical absenteeism, as shown in the preliminary results of the pilot study [17], and better school performance. The ongoing study of effects will provide conclusive evidence in this respect. When considering using MASS to address medical absenteeism, it should be realised that, although MASS is a well-defined intervention, it is not easy to implement. This is typical when focusing on a group at risk, and also when effectuation and funding involve multiple sectors [20, 32, 33]. Exaggerated expectations can discourage people. Therefore, a long-term view of program dissemination and a simultaneous focus on short-term outcomes are needed to maintain motivation when implementing MASS [19, 23].

Finally, prioritizing is needed to ensure complete records, and a professional approach to the dialogue by the investment in training. Prioritization can be achieved when the addressing of medical absenteeism is put on schools’ agendas. Intersectoral health policy of both the educational and health sector is needed for achieving overall agenda setting, including overall dissemination and sustainability [24, 34].

Children’s development can benefit from the reduction of medical absenteeism. Their educational and health outcomes can be promoted, whereupon the adult health status of the students receiving the intervention as well as the health of their future children can ultimately be improved [35–37]. Therefore, MASS can be helpful to diminish socioeconomic health inequalities. In 2014, Hawkrigg and Payne [38] recommended health professionals to intervene with school absenteeism and proposed an approach to tackle it. In our study we looked at implementation aspects of such an approach, from schools’ perspectives. To the best of our knowledge, this study is the first to explore schools’ attitudes towards the addressing of medical. Our results therefore add to the international knowledge on this topic, where other (international) readers can benefit from. The study outcomes can be used to study teachers’ and parents’ attitudes towards addressing the issue. It would also be recommendable to explore how teachers can best be trained.

Some limitations must be considered. MASS has been developed within the boundaries of Dutch legislation and policy on school absenteeism, and public and school health care. To some extent this affects the general applicability of this study, for example with regard to the role and position of the YHCP that can differ in other countries. In some countries schools decide that sick reports must be verified by an appropriately licensed medical professional in order to be accepted. However, GPs mostly do not advice about the consequences of a disease for the functioning and caregiving of the student at school, or about the educational adjustments needed. That makes that schools cannot act upon this information by providing necessary adjustments for the student. This should be considered when addressing medical absenteeism. A strength of the study is that dealing with sick reports is a universal problem and that discussing student’s medical absence should be based on concern rather than a control point of view. Strengths of the study also include a design that ensures broad, complete, and detailed information, and the minimization of socially desirable answers through the interviewing of individuals rather than groups. In a drive for high internal validity, a topic guide was used to channel the interviews for collecting the required data, and the interviewer was always accompanied by a second researcher. To counter the pro-innovation bias, the findings were presented to the participants and a multidisciplinary research team, and their comments were incorporated.

## Supporting Information

S1 File Dataset interviews. (DOCX)

S2 File Translation of quotes. (DOCX)
