# Supplementary material for: Attitudes towards Addressing Medical Absenteeism of Students: A Qualitative Study among Principals and Special Education Needs Coordinators in Dutch Secondary Schools
Source: PLoS One. 2016 Feb 4;11(2):e0148427. doi: 10.1371/journal.pone.0148427 (PMC4742281; doi:10.1371/journal.pone.0148427)
Supplement: S1 File — (DOCX) [file pone.0148427.s001.docx]

**Interview 1. M@ZL onderzoek Datum: 13-12-2011**

**Aanwezig: Yvonne Vanneste (onderzoeker), Marlou van de Loo (semi-arts)**

**Zorgcoördinator school 1 / S_1_**

**Algemene gegevens**

Het onderzoek vindt plaats in een van de middelbare scholen die mee doen aan het onderzoek in de kantoorruimte van geïnterviewde. Interviewer geeft een korte introductie over de bedoeling van het onderzoek. De sfeer is informeel, aangezien betrokkenen elkaar al jarenlang kennen. Op het begin is het nog wat onwennig, maar al snel ontstaat er een natuurlijk gesprek. Er is goed oogcontact en er is sprake van een bijdragende non-verbale communicatie. Deze is in het begin nog wat gesloten, maar wordt naarmate het gesprek vordert steeds opener. Er worden soms grapjes gemaakt tussendoor. Er worden veel open vragen gesteld. Je merkt dat de interviewer al voorkennis heeft over de school en de gang van zaken aan de hand van bepaalde vragen. De geïnterviewde geeft eerlijk antwoord op de vragen, ook als dat soms wat moeilijk (kwetsend) is ten opzichte van bepaalde personen / groepen. Betrokkenen zijn gemotiveerd om het gesprek te voeren en tot een goed einde te brengen.

**Verslag interview**

**Vragen/opmerkingen door interviewster dikgedrukt**

Antwoorden/opmerkingen door geïnterviewde in normale opmaak

**Ik heb al uitgelegd waar het interview toe dient. Nu wil ik eerst een paar vragen stellen over jou zelf, (..). Hoe lang ben je werkzaam op deze school?**

Sinds maart 1981, dus bijna 30 jaar.

**Wat zijn je achtergronden, wat is je opleiding?**

Ik heb PA gedaan en toen ben ik hier begonnen en ik werk hier dus nog steeds als docent. Ik geef Nederlands, doe RT en sinds een jaar of tien ben ik zorgcoördinator.

**Wat houdt zorgcoördinator in?**

Alle leerlingen die meer zorg behoeven dan dat de mentor aankan, komen in principe bij mij en ik ga dan kijken wat er nodig is. Of we externen moeten inschakelen, of dat het gewoon in de leerlingenbegeleiding kan, via een handelingsplan, via het ZAT.

**Je zit het ZAT ook voor volgens mij?**

Ja, dat klopt.

**Als je iets mag vertellen over deze school, wat is dit voor een school?**

Een hele leuke school, natuurlijk. Dat meen ik echt, ik denk nog steeds dat dit echt een leuke school is, met over het algemeen ook veel lieve, aardige leerlingen. Natuurlijk zitten er ook wel wat vervelende leerlingen tussen. Ze zijn ook lief naar elkaar toe.

**Wat is lief?**

Nou, aardig voor elkaar zijn en voor elkaar opkomen als het nodig is. Over het algemeen dan, want er zitten natuurlijk ook wel vervelende leerlingen tussen. Ik heb ook wel het idee dat, als ik het vergelijk met toen ik hier begon en nu, dat de leerling wel moeilijker aan het worden is.

**Hoe bedoel je dat?**

Gedragsmatig meer problemen.

**Zoals?**

Noem alle gedragsproblemen maar op. ADHD-ers zijn er volgens mij meer, ook REK4 kinderen, met grotere problematiek. Véél meer problemen in thuissituaties. Ik vind het altijd opvallend dat bij de school maatschappelijk werkster, dat leerlingen die daar in gesprek zijn, dat zijn leerlingen waarvan óf een van de ouders overleden is óf de ouders gescheiden zijn.

Dit wil niet zeggen dat álle kinderen van alle gescheiden ouders daar terecht komen, maar ik vind het wel opvallen.

**Dus jullie hebben ook een maatschappelijk werkende, wie hebben jullie nog meer hier voor de leerlingbegeleiding?**

De maatschappelijk werkster zit 1 dag in de week echt hier op school, verder hebben we natuurlijk intern de mentoren en ik als zorgcoördinator en twee algemeen coördinatoren. Extern heb je dan ook nog de leerplichtambtenaar die ook in het ZAT zit en een aantal ambulant begeleiders die de rugzak-kinderen begeleiden.

**En die blijven?**

Dat denk ik niet, die zullen allemaal wel verdwijnen binnen nu en een paar jaar.

**Hoe zie je die ontwikkelingen?**

Nou, geen idee. Dat vind ik lastig. Ik denk dat het een hele klus gaat worden. Wat ik net al zei, het idee dat het aantal gedragsmatige problemen al groter aan het worden is, er zijn zwaardere problemen en je krijgt er minder begeleiding voor. Dit jaar is de begeleiding van de ambulant begeleiders al aardig terug geschroefd en eigenlijk komt het erop neer dat ze alleen komen als het écht nodig is.

**Welke steun hebben jullie als school aan een ambulant begeleider?**

De meerwaarde is dat die intensiever contact heeft in de thuissituatie en met name als het gaat over heel specialistische zaken. REC3kinderen bijvoorbeeld, met een of andere ziekte zeg maar.

**Wat is REC3?**

Dat zijn de langdurig zieken, maar goed, als je er een hebt met een zeldzame afwijkingen, dan weet zo’n ambulant begeleider daar veel meer van dan wij natuurlijk. Want die heeft zich daar in verdiept.

**Hoeveel kinderen hebben jullie in die categorie?**

We hebben ongeveer 15 rugzak kinderen, dat is REC 2,3 en 4. REC 2 zijn de spraak- en taalgestoorden.

**Op een school van hoeveel leerlingen?**

Iets meer dan 500.

**Hoe komen die hier naartoe? Die zitten hier en worden begeleid?**

JA, sommigen hebben wel op REC 2,3,4 gezeten.

**Hoe komen ze bij de ambulante begeleiding?**

Als je een rugzakje hebt aangevraagd, dan krijg je die erbij. Als je een indicatie krijgt. Ik heb geen idee hoe dat in de toekomst zal gaan. Of dat het er helemaal af gaat of dat je dan als school het budget mag beheren. Nu is het natuurlijk dat als je een rugzak-kind hebt, dan krijgt de ambulant begeleider, die dienst, een stuk van het budget voor de begeleiding en je krijgt als school een stuk budget voor de begeleiding die je mag inzetten voor die leerling, op welke manier dan ook nodig is. Maar hoe dat straks zal gaan?

**Het blijft natuurlijk in ontwikkeling, het onderwijs.**

**Je zei iets over lieve leerlingen, kun je iets zeggen over de ouders van deze leerlingen?**

Over het algemeen zijn dit goede, positieve ouders, maar je merkt wel vind ik in de loop van de jaren, dat ouders wel steeds kritischer worden. En je komt vaker tegen dat de ouders minder achter de school staan dan een aantal jaren terug. Ouders gaan blindelings af op wat hun kind vertelt en niet op wat school vertelt.

**En dan?**

Dan heb je een probleem. Wij proberen in gesprek te gaan en dat lukt uiteindelijk vaak wel, maar dat kost wel eens heel veel moeite. Soms moet dat echt op directieniveau worden uitgesproken, sommige ouders willen ook alleen maar met de directie spreken. Maar goed, dan is het al wel heel hoog opgelopen, maar dat komt wel voor. Al denk ik dat je vroeger dat soort ouders ook wel had, maar de tendens op het ogenblik is gewoon een beetje meer dat ouders er meer bovenop zitten. Ze laten de kinderen niet zelfstandig worden. Op het moment dat hier iets gebeurt en ze sms’en al naar huis, dat staat binnen 5 minuten papa of mama al op de stoep. Dan denk ik wel eens: waarom laat je het je kind zelf niet oplossen?

**En dan zegt Marja Bijsterveld dat de ouders zich er meer mee moeten bemoeien (lacht).**

Ja, maar de vraag is of dat op de goede manier is, natuurlijk.

**Beetje een dubbele boodschap.**

Er zullen vast kinderen zijn, waar ouders zich helemaal niet of veel te weinig met hun kind bemoeien. Die zijn er ook.

**Kun je iets vertellen over het lerarenteam? De samenstelling van de mensen die hier werken?**

Ik heb geen precieze getallen, maar ik weet wel dat we gemiddeld gezien een oud team hebben. En we hadden een aantal jaren terug wel wat jongere collega’s, maar door terugloop van het leerlingenaantal, moesten zij er als eerste weer uit. Dat vind ik wel heel jammer, want die zijn over het algemeen enthousiast, ze brengen weer frisse ideeën mee. Die ouderen zijn niet zo geneigd om dingen te veranderen natuurlijk. Of natuurlijk, ik hoor er nu zelf ook langzamerhand ook bij (dat moet je niet noteren). Je merkt dat toch zeker diegenen die tegen het pensioen aan beginnen te lopen, dat ze iets hebben van ‘het zal mijn tijd wel duren’. En dat is moeilijk, want eigenlijk moet iedereen mee in de veranderingen.

**En jullie hebben een nieuwe directeur?**

Ja, een interim directeur. In het voorjaar is die er alweer 2 jaar en ik heb geen idee hoe dat verder gaat.

**Hoe is dat?**

Anders, heel anders. Onze vorige directeur heb ik al die jaren, zo lang als ik hier werk, mee samen gewerkt. Je hebt een band met elkaar opgebouwd en er was ook wel een klik. En deze is een interim, die komt alleen maar om de zaken te regelen en die staat heel anders in persoonlijke relaties naar collega’s toe. Dat vind ik voor mezelf een verarming, een gemis.

**Heb je er ook last van? Merk je dat in je werk, in je uitvoering?**

Ja, ik weet dat hij mij wel waardeert, dus in die zin krijg ik wel ruimte en medewerking van hem, maar als het gaat over een stukje persoonlijke aandacht, vind ik het een gemis. Dat vind ik gewoon jammer. Maar dat is niet meer dan dat. Het belemmert me niet in m’n werk.

**Ik wil met jou naar het onderwerp ziekteverzuim. Van leerlingen.**

Niet van ons?

**Daar mogen we het ook over hebben. Dat is ook interessant.**

Ik ben bijna nooit ziek, gelukkig.

**Hoe kijk je tegen ziekteverzuim aan van leerlingen?**

Kijk, iedereen kan wel eens ziek zijn. En ik vind het soms heel lastig om te oordelen over anderen. Je ziet toch wel, vooral nu ik daar een paar jaar bewuster mee bezig ben, toch altijd dezelfde namen terugkomen. En er zitten erbij die gewoon echt iets mankeren en dan snap ik ook wel dat ze er regelmatig niet zijn, maar van anderen snap ik het niet. Daar kom je gewoon niet doorheen heb ik het idee.

**En hoezo ben je er bewuster mee bezig?**

Omdat ik iedere keer ga kijken wie moet ik aanmelden, wie voldoet aan de criteria en dan zie je toch iedere keer dezelfde namen langskomen. Sommige dus al een paar jaar, dan denk ik ja, het was vorig jaar precies hetzelfde en het jaar daarvoor ook, maar toen deden we het onderzoek nog niet.

Maar goed, sommige kinderen mankeren ook gewoon echt iets en dan snap ik het ook wel. En in deze tijd van het jaar, weet je ook gewoon dat mensen sowieso in het algemeen wat vaker ziek zijn dan in de zomermaanden. En sommige kinderen zijn natuurlijk wat vatbaarder. Dan is er verder geen ‘kwade wil bij’. Maar, die ongrijpbare groep…

**We hadden het net over de afgelopen jaren, zie je in het verzuim ook een ontwikkeling?**

Nee, ik zie daar op dit moment geen ontwikkeling in. Dat had ik eigenlijk gehoopt met het M@ZL project, dat we vrij snel het ziekteverzuim wat zouden kunnen terugdringen. Maar dat zie ik zo nog niet gebeuren. Maar dat is puur op mijn gevoel gebaseerd.

**En dat had je wel verwacht?**

Ja.

**Hoe komt dat?**

Ja, die kinderen die echt ziek zijn, die blijven ook echt ziek. Ook met het project, want die zijn gewoon ziek en die melden zich ook ziek als ze ziek zijn. Dat blijft zo. En die groep met het vagere verzuim, dat is denk ik ook de meest ongrijpbare groep.

**Wat voor effect had je verwacht van M@ZL? Dit is nu het 2^e^ jaar. Je zegt ‘ik had verwacht dat het ziekteverzuim omlaag zou gaan’, had je nog meer verwacht?**

Nou, misschien ook wel dat je, vooral van de wat vage meldingen, dat je daar wat meer duidelijkheid in zou kunnen krijgen. Wat speelt er nou eigenlijk precies rondom zo’n kind? En hoe komt het dat er zoveel verzuim is. Maar misschien waren mijn verwachtingen ook wel niet goed of te hoog gespannen, dat kan natuurlijk ook.

**Je suggereert dat die verwachting niet is uitgekomen.**

Ja.

**Hoe komt dat?**

Geen idee.

**Laten we kijken naar het volgende. Hoe gaat het hier op school, hoe doen jullie het?**

Wij hebben PARS en ik heb me erop geabonneerd dat ik iedere week een melding krijg van kinderen die voor de 4^e^ keer ziek zijn en dan ga ik kijken of dat inderdaad klopt. De afspraak is dat ook de mentoren het mee in de gaten houden. Ik krijg ook regelmatig van bepaalde mentoren een mailtje dat een bepaalde leerling aan de criteria voldoet en of ik daar dan naar wil kijken.

**En jij coördineert dat?**

Ja, ik coördineer dat en ik doe het eigenlijk ook nog helemaal. Als ik ziek word, dan word er niet meer gemeld ben ik bang. Het moet nog steeds meer ‘body’ krijgen denk ik. Daar ben ik nog naar op zoek.

**Intern bedoel je?**

Ja.

**Je zei net ‘geabonneerd op’ en dan bedoel je op de nieuwe software?**

Nee, je kan je in PARS abonneren als mentor of als coördinator en dan kun je aangeven wanneer je een melding wil krijgen.

**Want er is nu nieuwe software, dat weet je he?**

Ja, door die nieuwe software, zodat bij leerlingen met te veel verzuim een automatische melding komt. Dat maakt het wel een stuk gemakkelijker, dat kost nu veel minder tijd.

**Dat was ook een van de grootste hobbels en daarom hebben we het nieuwe systeem laten bouwen, dus daar zou ik echt gebruik van maken. Dat scheelt je heel veel werk.**

Maar bij mentoren leeft het ook nog niet genoeg. Want ik krijg van een aantal mentoren wel een melding over leerlingen en vaak zie je dan wel, oh, dat heb ik net ook in m’n mailtje gehad. Maar er zijn ook mentoren die dat nog helemaal niet in de gaten houden.

**Hoe komt dat?**

Omdat mentoren zo verschrikkelijk veel moeten doen. Het gaat pas opvallen bij mentoren als kinderen echt heel veel afwezig zijn. En wat is echt heel veel? Dat is ook maar een gevoel van docenten. En als ik een klas op bijvoorbeeld maandag heb en dat kind is altijd op maandag ziek, valt het mij op dat het kind er nooit is op maandag. Maar als een mentor dat kind/die klas juist niet op maandag heeft en het kind is de rest van de weet wel op school, dan heeft hij dat niet zo snel in de gaten. Dan duurt het veel langer, als je dus niet in PARS zou kijken.

**Want zo was het dus voordat we M@ZL hebben ingevoerd.**

Ja.

**Dat was een van de grootste problemen eigenlijk, dat het soms pas heel laat opvalt als een kind veel ziek is. Dat we dat veel eerder willen signaleren, waarvoor we dus ook criteria hebben opgezet. Die ergens arbitrair gekozen zijn, maar waar ergens ook heel goed over nagedacht hebben. ‘Dit is veel verzuim’. Je gebruikt dus criteria, die het verhaal moeten ondersteunen, maar toch loopt het soms mis in bepaalde klassen. Hoe komt dat?**

Wat ik al zei, mentoren hebben al zo veel wat ze moeten doen, waar ze naar moeten kijken.

**Is dat de enige reden?**

Dat is wel een hele belangrijke reden in ieder geval.

**Zouden er nog meer redenen zijn?**

Dat weet ik niet. Als mentoren verder nergens naar hoeven te kijken, alleen naar het ziekteverzuim en dat met kinderen bespreken, dan zou het lopen als een trein denk ik. Maar zo zit het niet. Er is veel meer, kinderen die hun huiswerk niet bij zich hebben of niet gemaakt hebben, die geen spullen bij zich hebben, die gedragsmatig vervelend zijn, die eruit gestuurd zijn, ouders die ergens een vraag over hebben of bellen. Cijfers. Er is zoveel.

**En dan is ziekteverzuim eigenlijk…**

Dat heeft nog geen prioriteit. Terwijl het misschien bij sommigen wel de oorzaak is van alle problemen die er spelen. Als een kind veel moet inhalen of veel gemist heeft, komt dat soms voort uit het ziekteverzuim.

**Ja, en ziekteverzuim is ook een signaal he?**

Ja.

**Hebben jullie het daar wel eens over? Over ziekteverzuim van leerlingen? In de wandelgangen?**

Ja, zoals je het hebt over mensen die ziek zijn. De een is vaak ziek, de ander niet. Waar ligt dat aan?

**Jullie waren uiteindelijk, als ik terug kijk, eigenlijk een van de eerste scholen die begonnen zijn met ziekteverzuim begeleiding. Een voorloper, al voordat M@ZL is opgezet.**

We hebben toen al wel een ziekteverzuim-project gehad ja.

**Ik zie jullie eigenlijk als een van de meest gemotiveerde scholen om het te doen. Toch proef ik ook dat het heel moeilijk is om dat in de hele school te laten zingen. Ik ben op zoek naar het antwoord op de vraag: ‘hoe komt dat nou’. Door het ‘druk hebben’, dat geloof ik meteen. Zijn er andere redenen te bedenken die ook meespelen?**

Ja, dan komen we meteen op de resultaten van aanmeldingen misschien. Dan denk ik vooral dat die hardnekkige verzuimers, die blijven hardnekkig verzuimen. Dus ik denk dat er dan op een gegeven ogenblik ook een gevoel ontstaat van ‘ja, ik kan ze allemaal wel aanmelden, maar er verandert toch niets’. Dus waarom zou ik daar mijn tijd nog insteken.

**Dus eigenlijk, wat levert het op?**

Ja.

**Dat is niet genoeg, na wat ik investeer.**

Ja, ik denk dat dat ook wel echt meespeelt.

**Dan zeg je eigenlijk weer dat het resultaat een afname van het ziekteverzuim is, zou je andere resultaten kunnen noemen van zo’n project?**

Nou, misschien zijn er ook wel kinderen bij wie het verzuim niet af kan nemen, maar dat er meer begrip voor kan komen. Dat niet alleen de mentor, maar een heel docententeam begrijpt waarom een kind er regelmatig niet is. Wat dan ‘regelmatig’ dan ook mag zijn. Maar daar kan dan begrip voor komen. En als je dat zou kunnen bereiken, dan vind ik dat ook al heel wat.

**Ik krijg nu het idee dat dat hiermee niet bereikt wordt?**

Nee, nog niet.

**Hoe komt het, dat het begrip voor leerlingen die meer moeten verzuimen, om welke reden dan ook, nog niet is verbeterd?**

Dat is moeilijk, dan moet je ook begrijpen wat een bepaalde ziekte inhoudt, waarom een kind er niet kan zijn. Als je bijvoorbeeld migraine noemt, waarin je heel veel gradaties hebt, dan denk ik zeker dat er mensen zijn die zeggen ‘jeetje kind, als je wat hoofdpijn hebt, kan je toch wel naar school komen?’. Oké, nou migraine, dan ben je misschien wel eens een dag thuis. Maar er zijn er ook die er veel meer last van hebben en daardoor meer verzuim hebben. En dan moet je dus al kennis over de migraine hebben, om dat te kunnen snappen. Dat is dan alleen nog maar migraine, maar zo heb je natuurlijk nog 1001 andere aandoeningen.

**Bedoel je dan ook eigen ervaringen?**

Nee, dat hoef je niet zelf te hebben om te snappen hoe het is. Maar de kennis daarvan. Iedereen heeft wel eens van migraine gehoord, maar denkt ‘nou met een dag in je bed, ben je er wel weer’. Dat denken mensen vaak, maar dat geldt natuurlijk ook voor allerlei andere ziektes.

**Op dat niveau, krijg je dus geen terugkoppeling van een jeugdarts?**

Nee, maar ik weet ook niet of dat haalbaar is, want dat geldt voor alles wat kinderen mankeren. Kijk, dan kom je misschien terug bij direct 3 kinderen met name, waar echt medisch iets / chronisch iets hebben, waar je de begeleiding krijgt. Die komt een keer uitleggen wat het kind mankeert en dan is er begrip. We hebben vorig jaar een leerling gehad, die heeft vier jaar hier gezeten, en die bleek twee zeldzame syndromen te hebben, waardoor die heel veel dingen niet kon en de prognose van het kind anders was. En toen hebben we daar uitleg over gekregen en had iedereen zoiets van ‘wauw’, dat is wel heftig, dat had ik niet in de gaten. En dan heeft ineens iedereen begrip voor dat kind en dat is ook wat je wil natuurlijk.

**Maar dat zijn er 15 op 500.**

Dat is een utopie om dat van alle ziektes….

**Ziekteverzuimbegeleiding volgens M@ZL, daar zullen die kinderen misschien ook bij zitten, of niet. Het is niet eens gezegd dat je met een chronische aandoening meer moet verzuimen.**

Bij dit kind was dat toevallig wel het geval.

**Dat is een heel anders uitgangspunt waar je van uitgaat. Daar ga je uit van ‘ik heb een ziekte en ik leg uit wat voor consequenties dat heeft’. Ziekteverzuimbegeleiding volgens M@ZL heeft veel meer te maken met ‘wij gaan structureel aandacht besteden aan ziekteverzuim’.**

Jawel, en ik snap ook wel dat het altijd tussen kinderen ook een verschil blijft. Vorig jaar hadden we twee kinderen met een gebroken been. En de een kwam na een week in een rolstoel op school en de ander is drie maanden thuis geweest.

**Aan wie ligt dat nou? Wat denk je?**

Ik denk vaak aan ouders. ‘Het kind is zo zielig’ en ‘hoe moet je toch naar school in een rolstoel’. Terwijl we hier toch ook een lift hebben, dat maakt allemaal niet uit.

**Hoe bespreken jullie en wie bespreekt op school het ziekteverzuim?**

De mentor.

**Hoe doet die dat?**

In een gesprek. Kind wordt uitgenodigd, of als zoiets gebeurt heb je telefonisch contact met de ouders. Wat is er aan de hand? Hoe lang gaat het duren? Dan hoop ik dat er ook wel eens gezegd wordt ‘goh, maar zou je misschien niet met de rolstoel naar school kunnen komen’. Maar dan kom je weer terug bij de mentor, dat is natuurlijk ook een verschil per mentor denk ik. Hoe die zo’n gesprek aangaat. Want als de mentor mee gaat met de ouders dat het zo zielig is en ‘ik snap wel dat die thuis blijft’… En je moet daar altijd zo voorzichtig mee zijn ook, want ouders voelen zich al heel snel aangevallen. Zo van ‘je denkt toch niet dat ik mijn kind zomaar thuis hou’.

**Dat is jullie ervaring? Ouders die zich snel aangevallen voelen? Jammer he?**

Ja, dat merk ik ook duidelijk als ik de aanmelding heb gedaan. Ik krijg zoveel telefoontjes.

**Nog steeds?**

Ja, nog steeds.

**Wat zeggen ze dan?**

Nou, ik vind het niet nodig om te gaan. We zijn in behandeling bij de huisarts en dat vinden we genoeg. Ik ga niet nog een keer mijn kind van school houden om naar de GGD te gaan, want daar heb je toch niets aan. En jullie kunnen daar toch niets aan veranderen, en de GGD ook niet. Dat soort opmerkingen, ik kan daar niet zoveel mee.

**En wat zeggen jullie dan?**

Ik blijf zeggen dat ik vind dat ze daar eigenlijk toch naartoe moeten gaan, omdat de bedoeling is dat ze kijken hoe we het zo comfortabel mogelijk kunnen maken voor het kind. Zodat er zo weinig mogelijk verzuim is. ‘Ja, daar kunnen jullie als school toch niets aan veranderen. Hij heeft nu eenmaal dat en daar kunnen jullie toch niets aan doen’.

**Moeilijk hè.**

Ja, vind ik heel moeilijk. En er staat er artikel in de schoolgids, het is op alle algemene ouderavonden dit jaar al uitgelegd over het project.

**Krijg je dan ook reacties?**

Niet veel nee. Maar goed, niet alle ouders komen op de algemene ouderavond en ik durf niet te zeggen of de ouders die nu bellen niet op die algemene ouderavond geweest zijn. Als ik dat ook nog bij moet gaan houden..

**Dan krijg je eigenlijk van de ouders te horen ‘jullie kunnen daar toch niets aan doen’. Hoe zie jij de rol van school in het ziekteverzuim? We hebben bij ziekteverzuim natuurlijk een school, de GGD, de gemeente, de leerplichtambtenaar.**

Ik vind dat heel lastig. Ik snap ouders soms ook wel zelfs. Dan denk ik, je kind heeft nu eenmaal die ziekte of afwijking of reden voor veel verzuim. Maar moeten we het dan meteen zo hoog gaan spelen dat we de leerplicht daarbij in gaan schakelen. Ik vind dat ook heel lastig. En dan snap ik ook wel dat de ouders boos worden.

**Dan ga je meteen door naar de leerplicht. Maar ze worden ook al boos als je de jeugdgezondheidszorg inschakelt?**

Ja, goed, ze worden niet allemaal onmiddellijk boos, maar ze gaan dan bijvoorbeeld niet.

**En degenen die wel zijn geweest, wat krijg je daarvan terug?**

Van ouders eigenlijk heel weinig. Of eigenlijk niets.

**En van de leerlingen? Hoor je daar wel eens wat van?**

Ja, nou nu niet meer, vorig jaar toen het net gestart was wel. Toen ben ik meteen veel gaan melden en toen hoorde ik wel verhalen van ‘als je daar naar toe moet, krijg je van dat mens toch geen gelijk, dat is een kreng’. En ‘daar ga ik niet naar toe, daar heb je niets aan’. Dat was niet zo positief.

**Maar nu hoor je niets meer?**

Nee.

**Wat vindt je daarvan?**

Ja, dat vind ik moeilijk. Omdat ik het soms ook wel kan begrijpen, vanwege mijn gevoel wat ik dan heb om sommige kinderen te moeten melden. Dan denk ik, ik snap ook wel dat je vind dat je daar niets te halen hebt / geen winst kan halen. Dus in die zin kan ik dat wel snappen. En aan de andere kant snap ik het niet, dan denk ik ‘ja, je hebt ook eenmaal veel verzuim’. Natuurlijk moet daar wel eens tegen in gegaan worden en dat is wat er ook gebeurt. Zet je daar maar eens overheen. Maar dat willen ze natuurlijk ook niet allemaal horen. Ik probeer ook wel, als ik dat soort verhalen van vorig jaar hoor, om daar wat van te zeggen. Dat het de bedoeling is dat ze juist minder gaan verzuimen en om te kijken wat wij daar aan kunnen doen, als school zijnde. Dus wat we als school kunnen doen om jou meer op school te krijgen. Dat is de bedoeling. Maar dat komt dan toch niet helemaal aan bij de leerlingen.

**Bij ouders ook niet?**

Nee, bij ouders ook niet.

En sommige ouders denk je dan een goede uitleg te hebben gegeven en die verzetten netjes de afspraak als ze niet op dat tijdstip kunnen, maar vervolgens gaan ze dan toch niet zonder zich af te melden. Dat kan ik dan niet volgen.

**En als je ze daarmee confronteert, wat zeggen ze dan?**

Dat heb ik nog niet gedaan. Eigenlijk zou ik de ouders dan moeten bellen en vragen waarom ze niet zijn gegaan, maar dat heb ik nog niet gedaan.

**En waarom niet?**

Geen tijd.

**En als je de tijd wel zou hebben? Zou je het dan wel of niet doen?**

Dan zou je het misschien niet doen, omdat je dan de confrontatie aan moet gaan. En dat is ook niet altijd leuk. *Want mensen voelen zich zo snel aangevallen, terwijl het juist niet als aanval bedoeld is. S_1a_*

**Je zei net dat je de kinderen soms ook wel begrijpt. Wat begrijp je dan precies van die kinderen?**

Nou, hoe de arts overkomt op kinderen, dat het een kreng is en pinnig.. zo komt ze op mij namelijk ook over. Daar heb ik het wel met jou over gehad, toen is het ook een tijdje wel beter geweest, maar soms krijg ik van die mailtjes, dat ik denk ‘kan dat niet anders?’. ‘Heb je dit en heb je dat’, niet altijd even prettig.

**Heeft dat invloed op het project?**

Dat denk ik wel. Tenminste, wat er dan vorig jaar gebeurde, als kinderen van elkaar horen hoe zo’n gesprek op de GGD verlopen is, dan zeggen anderen ‘ja, als het zo gaat, dan ga ik niet, dat wil ik niet’. Of ze komen met een verhaal dat ze tegen iemand zeggen ‘ik heb een goed gesprek, ze luisteren naar je, we kunnen daar wat mee, daar heb je wat aan’. Dat is wel heel anders. Maar goed, ik hoor er nu niets meer over, dus ik weet niet of ze elkaar nu onderling ook weer informeren. Mij viel het vorig jaar op, omdat ik toen zelf een klas had waar heel veel verzuim was en waar ik dus de helft van de klas ongeveer had aangemeld. En mijn naam staat onder de brief. Dus dan deed ze natuurlijk ook nog een beetje expres, dat ik het zou horen.

**Want wat wil je dan bereiken met zo’n gesprek bij de jeugdarts?**

Nou, wat ik eerder zei, begrip en duidelijkheid. Voor alle partijen denk ik. En dat ouders ook kunnen aangeven van ‘als er op school nou alleen maar eens begrip zou zijn voor dit of dit wat mijn kind mankeert’, dan zou het al veel helpen. Dus het moet van twee kanten komen. Er moeten ook dingen bij de ouders veranderen, maar ook op school moeten er dingen veranderen naar een kind toe.

**Dat begrip werd vorig jaar, zoals je dat mag verwachten van de jeugdarts, gemist.**

Door een aantal wel denk ik ja. Maar dat komt alleen al door de toon waarop een gesprek wordt gevoerd denk ik. Ik denk dat als jij of je collega een gesprek voert, dat is al anders. Dat is hier ook, de ene of de andere mentor. Dat zijn personen waar je mee te maken hebt natuurlijk.

**We komen zo nog even terug op het genoemde.**

**Ik wil nu even terug naar ziekteverzuim van leerlingen. Hoe ervaar jij het probleem? Waar lopen jullie als school tegen aan? Als we het hebben over ziekteverzuim. Hebben jullie daar als school last van?**

Ja, je hebt er vooral last van, omdat kinderen dingen missen. Ze missen uitleg van je les, waar je als docent persoonlijk geen last van hebt. Waar je wel last van hebt is dat als een kind er niet is, als er een proefwerk gemaakt moet worden of er niet is als ze iets moeten inleveren. En de afspraak is eigenlijk dat de leerling er zelf achteraan moet om dingen in te halen. In de praktijk gebeurt dat niet. Dus als leerkracht moet ik iedere keer als een kind iets heeft gemist, die leerling daarop aanspreken. Dat vind ik niet erg, maar als dat iedere keer bij hetzelfde kind is en dat heel veel wordt, dan wordt dat wel lastiger. Dan moeten ze niet alleen bij mijn vak dingen inhalen, maar ook bij al die andere vakken*. En dat zou er ook weer aan mee kunnen helpen dat een kind nog meer gaat verzuimen, want op het moment dat het kind dan op school is, wordt hij door tien docenten ineens bij z’n kladden gegrepen van ‘jij moet dit en dit nog inhalen en inleveren’ en dat kind ziet dan ook niet meer hoe hij dat voor elkaar moet krijgen. De stress wordt dan misschien zo hoog dat ze daardoor weer gaan verzuimen. - S_1b_*

**Dus het missen, daar hebben jullie de meeste last van?**

Ja. En daar komt dan voor het kind ook nog bij, dat ze uitleg missen over bepaalde onderdelen, maar ze moeten wel die stof verwerken en daar een proefwerk van maken. En dan zou het ook nog kunnen zijn dat ik de ene keer aan de ene leerling wat extra’s uitleg en de andere keer aan de ander.

**Wat is nu de reden waarom je als school iets aan ziekteverzuimbegeleiding zou moeten doen? Hoe zou je dit aan een andere school uitleggen?**

Je hoopt toch het verzuim terug te dringen, waardoor je op het gebied van werk inhalen enzo ook wat meer rust krijgt. En je jezelf meer kan richten op je les, in plaats van dat je eerst met verschillende leerlingen nog allerlei dingen af moet spreken, voordat je aan je les kan beginnen. Of je moet tegen die leerlingen zeggen ‘blijven jullie in de pauze maar even’, maar dan heb ik geen pauze meer. Ik denk dat, als je minder verzuim hebt, meer rust krijgt over het algemeen genomen.

**En als ik het terugpak, meer begrip?**

Ja, maar als er begrip is, ben ik ook wel bereid om een stapje extra te doen voor een leerling als het nodig is.

**Het motiveert jezelf om het te blijven doen.**

Ja, en vooral als je dan ook nog ziet dat het kind ook bereid is een stapje erbij te doen om dingen weer in te halen, dan gaat dan bijna vanzelf.

**Waarom denk jij, als zorgcoördinator, dat sommige kinderen zoveel ziekteverzuim hebben? Wat zit daarachter?**

Ik denk dat het in een aantal thuissituaties misschien handig is, als het kind thuis is om te helpen, als er nog broertjes en zusjes zijn, om op te passen. Misschien vindt moeder het soms ook gezellig als het kind een dagje thuis is. Maar dat kan ik natuurlijk niet hard maken.

**Nog andere redenen?**

Misschien maken ze zich wel zo druk over iets, dat ze denken ‘als ik thuis blijf, hoef ik dat niet te doen’. Bijvoorbeeld dingen over school. Dat ze het niet naar hun zin hebben, teveel spanningen op school, geen leuke klas. Lekker een dagje thuis, lekker niets doen, eigenlijk spijbelen, geen zin hebben. Dat zal ook wel meespelen.

**Ja, en in de thuissituatie nog meer redenen?**

Misschien dat de rollen omgedraaid zijn, dat kinderen voor hun ouders moeten zorgen. Of ouders in de gaten willen houden, die zijn er ook.

Sommigen zijn echt ziek.

**Sommigen zijn echt ziek, ja.**

Maar ja, wie bepaalt wat echt ziek is? Ik vond jullie uitspraak de laatste keer erg mooi, ‘ziek zijn overkomt je en je ziek melden is een keuze’.

Maar dat is toch zo in de hele maatschappij, niet alleen bij de leerlingen, maar ook bij mijn collega’s / docenten. Ik kan ze zo aanwijzen. Wat willen we dan van de kinderen, die krijgen toch het voorbeeld zo. Dat is een hele mentaliteitskwestie, in de hele maatschappij natuurlijk.

**En ergens moeten we beginnen met het omdraaien van die mentaliteit.**

Ja, maar dat is natuurlijk een klus van jaren.

**Ja, dan komen we bij het effect. Wanneer zie je effect? Wat verwacht je op korte termijn?**

Dan krijg je ook wat we toen straks zeiden, de mentaliteit. Als er hier iets gebeurt met kinderen, dan zijn erbij die sms’en naar pa of ma en die staan dan gelijk op de stoep om dingen te regelen voor zo’n kind. Dus op het moment dat het even niet zo lekker loopt, dan springen pa of ma al in de bres. Dus als een kind ’s morgens zegt ‘ik voel me eigenlijk niet zo heel lekker’.. in plaats van dat ze dan zeggen ‘kom op, je gaat gewoon naar school’, zijn er ook een aantal die zeggen ‘blijf maar lekker thuis vandaag’.

**Dan zegt de jeugdarts: ‘kom op we gaan naar school’**

Maar die kinderen gaan niet naar de jeugdarts.

**Wie gaan er wel naar de jeugdarts? Je meldt ze aan, ongeveer de helft komt opdagen. Wie komt wel en wie niet? Wat zegt je gevoel?**

Ik denk dat in ieder geval gaan degenen waar medisch gezien echt iets aan de hand is. Waar ouders positief tegenover school en begeleiding staan. De kinderen waarvan ik eigenlijk zou zeggen ‘die hoef ik niet te melden, want het komt wel goed met dat kind’, die gaan. Dan krijg ik een terugkoppeling van ‘dit en dit’ en denk ik, ja dat wist ik al. Dat heeft dus geen meerwaarde, maar is meer een bevestiging. Dat kan ook fijn zijn.

**Hoor je van die ouders ook wat terug?**

Ja, dat zijn de ouders die ook positief reageren op een telefoontje van de mentor met de uitleg wat er aan de hand is en zeggen ‘logisch dat jullie dit doen, prima’. Maar ik hoor niets nadat ze daar geweest zijn hoor.

Wie gaan er nog meer? Ja, een aantal twijfelgevallen gaan ook wel, maar wat daar de opbrengst van is, weet ik niet. Ik vind dat ik ook nogal wat terugkoppelingen krijg, en dan met name bij kinderen waar heel veel verzuim is, dat ik gesprekken met school maatschappelijk werk (SMW) moet opstarten. Dit gaat vaak over problemen in de thuissituatie, wat een oorzaak van het verzuim kan zijn. Dan denk ik, dat schiet niet op! Want zij zijn er juist niet. Wat heeft het voor zin als ik die gesprekken met SMW opstart, en dat zijn er best veel die zo teruggekoppeld worden, terwijl de SMW al heel vol zit. Moeten we die kinderen er dan ook nog allemaal bij inplannen? Terwijl ze niet op school zijn. Die nemen dan de plek in van een kind dat echt heel graag een gesprek wil hebben en die ook op school is. Dan moet ik zo’n kind wat verzuimt daarbij indelen, maar die is er niet.

**Breng je dat dan in het ZAT in of koppel je dat terug?**

Ja, dat koppel ik terug. Dan krijgen we vaak het advies een gesprek met de leerplicht te doen. Dan wordt er nog te weinig gezegd, vind ik, ‘zullen wij dan nog eens oproepen en eens kijken wat er nog moet gebeuren’.

**Dus je zegt eigenlijk: de jeugdgezondheidszorg zou meer kunnen doen.**

Ja, dan denk ik jeugdgezondheidszorg, jullie zouden ook andere adviezen kunnen geven in problematische thuissituaties dan alleen maar zeggen: het kind moet gesprek hebben met SMW, want er speelt thuis van alles. Dat is dan op dat moment niet de oplossing.

**Welke adviezen verwacht jij dan?**

Misschien verwijzen naar een GGZ of een maatschappelijk werk of ‘weet ik veel wat nodig is’. Als er financiële problemen zijn binnen een gezin, zijn er ook instanties waar mensen terecht kunnen. Dat soort adviezen zie ik niet. Ik zie alleen maar ‘gesprekken SMW opstarten’. En misschien moet je dat ook wel doen, maar daarnaast misschien ook in de thuissituatie wat meer adviseren.

**Goede tip.**

Soms heb ik best goede ideeën.

**Het is heel belangrijk om juist die afstemming te blijven zoeken. Wat ik bijvoorbeeld heel opvallend vind bij het noemen van redenen waarom kinderen verzuimen noem jij pas als laatste dat het kind ook echt ziek kan zijn. Daar wordt een jeugdarts natuurlijk ook mee geconfronteerd. Eigenlijk zijn wij ervan overtuigd dat er heel veel ziekmeldingen eigenlijk een keuze zijn geweest. Maar dat kunnen wij ook niet hard maken. Wij gebruiken ziekteverzuim als signaal. En daarbij hoort ook dat je in het gesprek aangeeft dat je denkt dat iemand zich helemaal niet ziek had hoeven melden en ‘het gaat goed met jou’. Dat is iets wat kinderen niet graag willen horen. Los van alle andere opmerkingen is dat wel iets waarvan ik denk, ‘daarin zit ook een stukje kwetsbaarheid van de jeugdgezondheidszorg’.**

**Wat zijn jouw verwachtingen als je naar de jeugdarts verwijst?**

Ik verwacht dan dat het verzuim onmiddellijk afneemt.

**Dat is misschien wat overtrokken, dat is niet reëel, want als je ziekteverzuim als signaal gebruikt, wil het niet zeggen dat je altijd het ziekteverzuim kan stoppen, maar je gebruikt het als signaal om…**

Om misschien hulp op te starten, of misschien ook niet, want in sommige gevallen is er ook gewoon niets nodig denk ik. Ik denk dat je ook moet oppassen dat je teveel op de hulpverlening gaat zitten. Daar moet je wel een weg in zoeken, want lang niet alles hoort in de jeugdhulpverlening thuis.

**(Grapje over geld, reknr)**

**Jullie betalen M@ZL, wat vind je daarvan?**

Daar heb ik nog niet over nagedacht. Want dat gaat helemaal buiten mij om. Er is tegen mij gezegd ‘wij gaan aan het M@ZL project deelnemen’ en ik weet niet eens hoeveel we daarvoor per leerling betalen.

**Er is jou eigenlijk verteld ‘dat gaan we doen’, had je daar iets in te zeggen?**

Er is volgens mij op het begin wel iets van gezegd dat het ging om VSV gelden, dat jij dat had ingediend. Toen is er gezegd, zou dat niet iets voor ons zijn? En toen heb ik gezegd, ja, want we hebben best veel verzuim. Verder heb ik daar niets meer over gehoord. Er is wel iets gezegd van geld, maar dat ontgaat mij hier altijd.

**Dus jou is opgedragen dat het project ging starten en toen?**

Nou, toen bleek dat ik dat werk er allemaal bij kreeg. Het kost heel veel tijd. Maar ik hoop nog steeds dat het uiteindelijk ook wat gaat opleveren en daarom hou ik vol. En kijk, nu met PARS moet dat weer beter worden, dus daar heb ik nu mijn hoop weer op gevestigd. Want op dit moment kost het me denk ik een halve dag per week en dat vind ik eigenlijk niet in verhouding staan tot wat het oplevert.

**(advies nieuwe systeem te proberen, programma aanvragen en dan krijg je toegang. Ria zegt ‘volgens mij heeft elke mentor nu toegang’. Straks even naar kijken)**

**Ben je geholpen in het opzetten van het project?**

Jawel, (..) heeft mij natuurlijk in het begin mee geholpen met opstarten.

**Wat hield dat opstarten in?**

Die bijeenkomsten bij jullie, daar zijn we een paar keer geweest met z’n tweeën. En toen ben ik het eigenlijk meteen al helemaal gaan doen.

**En wat heb je hier in de school gedaan om het op te starten?**

Een artikel in de schoolgids, op de portal, de mentoren ingelicht via de info die 1 x per week verschijnt. Daar heb ik het ingezet. Vorig jaar hadden we nog reguliere leerling-besprekingen en liet ik het ook iedere keer even aan bod komen. Dan zei ik ‘denken jullie aan ziekte meldingen en M@ZL’. Nu hebben we geen leerling-bespreking meer, maar heeft de mentor regelmatig een bespreking met de algemeen coördinator en is het ook een vast agendapunt.

**Dus het komt iedere keer terug.**

Ja.

**En hoe reageren de mentoren daarop?**

Daar hebben we het eigenlijk al over gehad. De een goed en de ander wat minder.

**Waar ligt dat aan? Heb je enig idee?**

Het ligt aan de persoon. Personen die het doen vinden dat het bij zijn mentortaak hoort en dus gaat die dat doen. Ik denk dat als de mentor op gegeven ogenblik in de gaten heeft dat het ook wat opbrengt, dat die dan blijft melden. Maar als een mentor een paar keer een leerling heeft gehad die bij M@Zl is geweest en er verandert verder niets. Dan denkt zo’n mentor ook ‘wat heeft het voor zin’.

**Hoor je dat ook terug of is dat meer jouw idee?**

Nee, dat hoor ik ook wel terug.

**Je zegt, het hangt van de mentor af, hoe die erin staat. Kun je nog wat eigenschappen noemen van een mentor waarvan je denkt, dat is goed?**

Je hebt van die mentoren die gaan gewoon voor hun leerlingen. Nee, dat is ook niet waar. Want er zijn mentoren die gaan ook voor hun leerlingen, maar die hebben lak aan alle administratieve rompslomp en die doen dat niet. Terwijl een mentor er eigenlijk verder geen administratieve rompslomp aan heeft, behalve het melden aan mij en een telefoontje met de ouders wat de reden van het verzuim is. Dat probeer ik er ook meer in te krijgen.

**Hoe gaat dat, dat telefoontje?**

Weet ik niet, dat doet de mentor.

In sommige gevallen hoor ik van de mentor terug, ‘die ouders snappen het en die wachten de uitnodiging af’, maar dat zijn juist die leerlingen, waar we het net ook al over hadden, die netjes gaan en waar je begrip en overleg met de ouders kan hebben. En er zijn ook ouders die vinden het vervelend en die doen ook tegen de mentor vervelend. Dat zijn dan dezelfde gesprekken die ik ook heb met de ouders als ze de brief hebben gehad. En dat willen de mentoren ook niet altijd. Dat kan ze er ook van weerhouden om te melden denk ik. ‘Krijg je weer zo’n vervelend telefoongesprek’.

**Dat kan ik me voorstellen.**

**Wat weet jij eigenlijk over de jeugdgezondheidszorg?**

(eigenlijk heel weinig)

Ze doen die PGO’s, vanuit het consultatiebureau, toch? Die PGO’s zijn een paar jaar amper geweest, dus ik heb het idee dat er ook bij de GGD erg veel bezuinigd moest worden, waardoor het allemaal op een heel laag pitje is komen te staan. Dit jaar hebben we ineens weer een verpleegkundige die alle kinderen uit 1997 oproept en daar komen soms ook weer opmerkelijke dingen uit, maar vaak komt er iets uit wat wij al lang weten. Dan hoor ik dingen terug die wij al weten.

**Wat is nou het doel van de jeugdgezondheidszorg, waarom hebben we dat?**

Preventief is volgens mij met name de bedoeling, hè?

Groei en dergelijke in de gaten houden. Oren, ogen, dat soort opvallende dingen, allerlei afwijkingen die zichtbaar zijn. Groeiafwijkingen, scheve rug enzo. Een jeugdarts kan ze dan op tijd verwijzen, niet echt preventief want er is dan natuurlijk al wat aan de hand.

**Lichamelijke dingen.**

Ja.

**Nog meer?**

Ik denk ook dat als je in zo’n gesprek andere dingen constateert, dat je ze ook kan verwijzen. Maar dat is wat ik zei, wat me nu opvalt is dat het meeste wat teruggekoppeld wordt, dingen zijn die wij toch al weten. En in een enkel geval nu, komen er nieuwe feiten boven.

**Zinvol?**

Ja, absoluut. Dus dan kun je zeggen: het is zinvol al die leerlingen uit 1997 op te roepen om er 2 uit te halen die nu naar de hulpverlening gaan. Maar die waren misschien toch boven komen drijven of niet. Dat kan ook natuurlijk.

**Wat je eigenlijk al zei, dat hoort eigenlijk tot het basistakenpakket, heb je daar ooit wat van gehoord?**

Ja, wel eens iets van gehoord, maar..

**Dat is eigenlijk landelijk vastgesteld wat er tot de taken behoort. Weet je door wie JGZ betaald wordt? Wie onze opdrachtgever is?**

Nee.

**De gemeente betaalt de GGD om de JGZ uit te voeren.**

Betekent dat dat het per gemeente dus anders geregeld kan zijn?

**Dat was vroeger dus wel zo, maar door het landelijke aspect gaan we dat nu tegen. Daarom is er een landelijk basis takenpakket, waar de gemeente minimaal aan moet voldoen. Dat takenpakket wordt door de beroepsgroep afgesproken en dat beschrijft van 0 -23 jaar wat er gedaan moet worden. De inspectie controleert of wij dat netjes doen.**

**Ik vraag me wel eens af als jeugdarts hoe jullie dat als scholen ervaren. De JGZ heeft scholen nodig om samen te werken om die kinderen te signaleren.**

**Stel nou dat we stoppen met die reguliere onderzoeken en we zeggen dat we ziekteverzuimbegeleiding gaan doen. Hoe zou jij daar tegenaan kijken?**

Ik denk dat het niet zo verkeerd zou zijn. Ik heb het gevoel dat bij die reguliere oproepen weinig nieuws naar buiten komt en dat ook daar de opbrengst nihil is. Nu zie ik bijvoorbeeld een aantal kinderen die veel te zwaar zijn, die worden verwezen naar de huisarts. Maar goed,dat die te zwaar zijn, dat hadden wij natuurlijk ook al geconstateerd en wel eens besproken met de ouders in een aantal gevallen. Dus misschien dat je als je op het verzuim gaat zitten, meer opbrengst zou kunnen krijgen. Ik weet het niet. Aan de andere kant kun je ook zeggen dat: ‘als je preventief niets meer doet, wordt het verzuim hoger’. Maar dat kan je zo ook niet stellen.

**Laat ik het anders stellen. We hebben jeugdgezondheidszorg in Nederland. Hoe zou jij als school willen samenwerken? Stel we hebben een budget en er zijn jeugdartsen, jeugdverpleegkundigen.**

Misschien dat er op een hele laagdrempelige manier om advies gevraagd kan worden. Door ons, door de school bij de GGD. Als ik vragen heb over een kind op medisch vlak, dat ik dat op een hele simpele manier bij jullie kan krijgen. Zonder dat ik daar allerlei toestanden voor handen moet halen en misschien ook wel een hoop moet betalen. Dan moet je waarschijnlijk per individu gaan betalen ofzo en dan is het denk ik weer minder laagdrempelig. Terwijl als ik een leerling heb die veel ziek is, dan kan ik tegen jou zeggen, ‘roep die eens op’. Telefoontje, oproepen, geregeld.

**En dan komen ze?**

Dat weet ik niet, waarschijnlijk ook niet. Of wel. Dan heb je eigenlijk hetzelfde. Of bijvoorbeeld ik maak me zorgen over een kind dat ik veel te zwaar vind, die zie ik elke keer spijbelen bij de gymles bijvoorbeeld, telefoontje naar jou, roep die eens op.

**En gaat dat werken?**

Dat weet ik niet. Misschien als je iemand binnen de school zou hebben, een verpleegkundige die helemaal laagdrempelig bereikbaar is voor mij, maar ook voor de kinderen zelf die daar misschien met vragen binnen kunnen lopen. En mij als docent kan adviseren ‘wat moeten we met zo’n kind’. Die misschien contact met de ouders op het medisch vlak kan onderhouden. Dat is een utopie waarschijnlijk. Als er op alle scholen een arts of verpleegkundige aanwezig moet zijn..

**Zou dat het zijn?**

Ik weet het niet. Ik denk dat je altijd, wat je ook bedenkt, altijd een groep overhoudt die je niet kunt bereiken. Die hou je altijd denk ik. En dat is een hele mentaliteitskwestie. Waarom kun je die niet bereiken?

**En als ze nu bij wet gaan regelen dat, net als binnen de bedrijfsgeneeskunde (arbo-arts), we dit doortrekken naar kinderen. Zou het dan opgelost zijn?**

Nee, mensen worden denk ik heel handig om dingen te verbergen. Dat gebeurt denk ik ook bij de bedrijfsartsen. Volgens mij blijft er altijd een groep die je niet kunt bereiken, wat je ook bedenkt. En dat is nu ook de groep met het hardnekkigste verzuim. En wat we daaraan moeten doen….

**Dat is de uitdaging.**

**Als je kijkt naar M@ZL, dan hebben jullie M@ZL compleet gekozen. (uitleg over M@ZL compleet en M@ZL smal, school die zelf verzuimgesprekken voert, criteria loslaten, eventueel doorverwijzen naar de jeugdarts)**

**Als jij mag kiezen, waar zou je dan voor kiezen?**

Toch voor wat we nu doen denk ik. Omdat je toch een meer onafhankelijk iemand hebt, die het gesprek met ouders kan voeren. Ik denk dat dat voor ouders dan ook prettig is. Als het door school gedaan wordt, denk ik dat ze eerder het gevoel hebben dat het een ‘wijzend vingertje is’ en als het door jullie gedaan wordt is dat gevoel een stuk minder denk ik.

**Dat zou je ook als argument kunnen gebruiken als ouders bellen en zeggen ‘waarom moet ik daar naartoe’.**

Dat doe ik ook, dan beginnen ze aan mij hun hele medische verhaal te vertellen. Dan zeg ik meneer/mevrouw, dat wil ik allemaal niet weten. Daar ga ik niet over, dat vind ik prettig en wil ik ook zo houden. Ik ben geen dokter, daarom verwijzen wij u. Naar een onafhankelijk iemand.

**Zijn er nog meer redenen waarom je dan toch opnieuw voor M@ZL compleet zou kiezen?**

Nou ik denk dat als je het allemaal zelf moet gaan doen, dat je daar ook tijd in moet investeren. Om die gesprekken te voeren.

**Dat besteedt je nu uit.**

Ja. En ik denk wel dat als je het zelf moet gaan doen, dat je dat pas doet als het verzuim echt heel groot wordt. Dat je dat minder snel zal doen dan wanneer je nu verwijst.

**En, dan?**

Dan zijn de problemen nog groter denk ik.

**Hoe zou je dat dan doen? Stel dat je M@ZL smal had gehad?**

Dan denk ik dat dat ook weer bij de mentor terecht zou komen. En dan ben je ook weer afhankelijk van welke mentor er is.

**Ik proef wel, het behoort tot het mentoraat, vind je. Het voeren van verzuimgesprekken.**

Ja, dat is mijn eerste ingeving. Maar ik heb daar verder nooit over nagedacht. Je kunt ook zeggen dat het bij de algemeen- of zorgcoördinator hoort.

**Wanneer gaan mentoren dan een verzuimgesprek voeren?**

Als het verzuim gaat opvallen. Als het teveel is, frequent of bijvoorbeeld iedere maandag. Of altijd dezelfde les. Maar goed, als je het echt allemaal zelf moet gaan doen, dan zul je daar intern afspraken over moeten maken. Welke criteria gaan wij hanteren? Wie gaan die gesprekken voeren?

**Het vraagt meer van de school zelf dan.**

Ja. Dus hoe dat dan precies zou gaan, dat weet ik niet precies.

**Hoe past ziekteverzuimbegeleiding in de cultuur van jullie school?**

Volgens mij past dat daar wel in. Omdat wij vinden dat wij een goede leerlingbegeleiding hebben. Zorg om de leerling. En daar hoort ziek zijn ook bij. Als een kind ziek is, heb je er zorgen om.

**En dat wordt ook gedragen door de mensen hier op school?**

Door het merendeel wel ja.

**Je zei in het begin ‘we hebben een wat oud team’, die hebben niet altijd zo’n zin in verandering. Heb je het idee dat dit bij de implementatie van invloed is?**

Ja, maar dat is niet alleen M@ZL. Dat geldt voor alle veranderingen die we aan het doorvoeren zijn, zoals passend onderwijs. Dat moet je veel breder zien.

**Passend onderwijs noem je, de rugzakjes die mogelijk gaan verdwijnen / anders verdeeld gaan worden…**

Mentoren hebben wel behoefte aan ondersteuning. En op wat voor vlak dat dan is, dat kan van alles zijn. Maar in feite is een mentor altijd blij met ondersteuning.

**En zie je daar dan een rol van JGZ in?**

Ja, dat kan. Ook als onderdeel van die ondersteuning.

**We hebben al veel besproken.**

**Na dit schooljaar loopt het project en gaan we door met M@ZL, maar als geïntegreerde werkwijze binnen de school en de GGD. Wat moet er nog gebeuren om dat echt goed weg te zetten, zodat het op lange termijn ook beklijft?**

Als het verzuim echt af kan nemen door M@ZL, dan moet je zeggen ‘dit is geweldig, hier gaan we mee door’. *We moeten het gevoel hebben dat het wat oplevert. S_1c_*

**In de vorm van..**

Afname ziekteverzuim en een beter beeld op wat er speelt, want dat heb je nu natuurlijk ook niet altijd.

**Dat komt ook omdat de helft nu niet komt opdagen, denk ik.**

**De groep die zo graag wil bereiken, bereik je nog steeds niet, dat proef ik een beetje uit jouw verhaal. Wat zouden we daar aan kunnen doen?**

Weet jij de oplossing? Ik weet het niet, want dat is net de moeilijkste groep om te bereiken. Dat is die groep die wat te verbergen heeft, wat speelt daar om een kind ziek te melden. Dan moet je de mentaliteitskwestie aanpakken. Dat kunnen wij hier niet zomaar even oplossen.

**Oké dat kunnen we niet oplossen, misschien moeten we accepteren dat we die groep gewoon niet bereiken.**

Maar dat is nu juist de groep die de meeste frustraties oplevert binnen de school, dus die zou je wel graag aanpakken.

**Kun je verbeterpunten noemen die ons kunnen helpen om ze beter te begrijpen? En om die groep kleiner te maken?**

Ja, om ze beter naar de GGD te laten gaan. Daar begint het natuurlijk, om ze naar de GGD te krijgen. Ik weet het niet. Als je die eerst uitnodigt voor een gesprek op school en vervolgens doorzet naar de GGD, denk ik dat je hetzelfde blijft houden. Dat is wat ik nu met die telefoontjes heb, dat ik aan ouders probeer uit te leggen. Een enkeling gaat dan uiteindelijk wel, maar de meesten gaan uiteindelijk toch niet. Ondanks dat ze in zo’n gesprek vaak tegen mij zeggen dat ze het wel snappen en erover nadenken / toch maar gaan. Dan gaan ze toch niet. Dus ik weet niet hoe het moet in die groep.

**Wat zijn de neveneffecten van M@ZL? Dingen dat je denkt, dankzij M@ZL....**

Nou misschien dat je toch wel wat alerter op het verzuim bent, toch wel iets vaker of sneller daar iets mee doet. Je hoeft niet meteen door te zetten naar de GGD, maar dat je er alleen maar oog voor hebt.

**Dat is een belangrijk neveneffect, dat je er alerter op bent.**

**Je mag ook negatieve neveneffecten noemen. Heeft het ook een nadeel dat jullie aan M@ZL meedoen?**

Voor mij persoonlijk dat het veel tijd kost, maar dat vind ik eigenlijk niet belangrijk.

**We gaan het afronden. Wat wil je me nog meegeven?**

Volgens mij heb ik alles wel kunnen spuien wat ik zou willen. Alles is wel gezegd.

**Dan stoppen we ermee.**

Dat is goed.

**Interview 2. M@ZL onderzoek Datum: 14-12-2011**

**Aanwezig: Yvonne Vanneste (onderzoeker), Marlou van de Loo (semi-arts)**

**Directeur school 2 / P_2_**

**Algemene gegevens**

Op school, kamer directeur.

Informele sfeer.

Veel non-verbaal contact.

Geïnterviewde praat erg makkelijk, weinig sturing nodig!

Je merkt dat hij al achtergrondkennis heeft over M@ZL en er al veel vanaf weet / er vaker over na heeft gedacht.

**Verslag interview**

**Vragen/opmerkingen door interviewster dikgedrukt**

Antwoorden/opmerkingen door geïnterviewde in normale opmaak

**Kun je iets vertellen over jezelf? Hoe lang ben je hier al op school, wat zijn je achtergronden?**

Ik ben op dit moment aan mijn 31^e^ jaar bezig op deze school. Ik heb in verschillende functies op deze school gewerkt. Eerst als docent nederlands en godsdienst, ik heb een pedagogische academie achtergrond. Toen ik eenmaal godsdienst gaf, kwam ik tot de conclusie dat ik daar toch iets meer van moest weten en ben ik theologie gaan studeren. Toen heb ik mijn MO (?) theologie gehaald, terwijl dat ik werkte. Daarom heb ik daar ook wel 6 jaar over gedaan, omdat ik andere dingen belangrijker vond. Heel veel geleerd waar ik nooit meer iets aan heb, maar naarmate ik meer theologie begon te bestuderen, ben ik minder gaan geloven. Maar daarmee is de beleving niet minder geworden.

Ik ben schooldecaan geweest, ben in de lerarencoördinatie werkzaam geweest, toen een half jaar adjunct-directeur, waarna mijn voorganger met pensioen ging en ik werd aangewezen deze school te leiden. Het is geen bewuste keuze geweest om directeur te worden, maar achteraf gezien wel een goede stap in mijn carrière. Vanaf 15 aug jl ben ik ook ad interim directeur van een school in Zeeland, Tholen. Daar was een probleem en zijn we met een oplossing bezig. Dat loopt rond februari af en dan ben ik weer terug hier, voor in ieder geval 1 jaar en dan gaan we kijken wat mijn ambities dan verder zijn.

**Waarom ben je ooit gestopt met docent zijn en ben je decaan geworden? Was dat wel een bewuste keuze?**

Dat was een heel bewuste keuze. Ik vind lesgeven heel erg leuk, maar een vak als godsdienst, dan ben je eigenlijk constant topsport aan het bedrijven om te zorgen dat kinderen het ook nog aanspreekt. Ik heb vroeger altijd toneel willen spelen, mijn vader zei dan ‘je mag zoveel studeren als je wil, maar ga eerst zorgen dat je een beroep leert’. Toen ben ik het onderwijs in gegaan en sindsdien heb ik volle zalen. Maar dat is wel heel erg vermoeiend, want werkelijk elk lesuur probeer je het onderste uit de kan te halen. Ik had het idee dat ik dat heel leuk vind en dat ook wilde blijven doen, maar tot mijn 65^e^ houd ik dat niet vol. Dus de combinatie van schooldecaan zijn en het lesgeven vond ik heel leuk. Die combinatie had ik ook nog toen ik adjunct-directeur was, alleen het lesgeven komt er nu niet meer van. Er zijn zoveel andere afspraken en je bent met zulke andere dingen bezig, zoals bijvoorbeeld een rooster en dan gaat de zoemer en denk je ‘och jee’ ik moet ook nog lesgeven. Dat vind ik heel vervelend.

**Wat vind je leuk aan het decaan zijn?**

De begeleiding van leerlingen naar een nieuwe fase in hun leven. Dat is heel belangrijk. Dat was voor mij ook wel iets waarvan ik dacht ‘nou, dat vind ik wel.. ehh’. De gesprekken met die kinderen waren ook gewoon heel leuk. Op het moment dat je laat zien aan kinderen dat je oprecht in ze geïnteresseerd bent, zijn ze heel open en krijg je hele leuke gesprekken met kinderen. En ook hun ouders komen dan, en dat zijn altijd positieve gesprekken, want je bent bezig met de keuze van hun kind, wat het kan of wat het niet kan. Daar moet je het met elkaar over hebben, maar in principe ga je altijd uit van het positieve. Je zit daar voor dat kind. Dus dat was altijd heel leuk.

**Gesprekken met ouders en kinderen, dat was altijd positief en dat vond je leuk. Je had ook zorgcoördinator kunnen worden. Maar dan heb je het misschien over minder leuke dingen.**

Ja, maar die hadden we al.

**Had je dat ook willen doen?**

Ja, op zich wel. De gesprekken die ik nu met kinderen en ouders voer, zijn in eerste instantie vaak gesprekken waarin je niet zo’n goed nieuws hebt, waarin je bezig bent met een vermaning of soms zelfs een schorsing of verwijdering van een leerling. Dus de gesprekken die ik nu voer, zijn in principe als uitgangspunt negatiever. Ouders komen nu met klachten of ik kom met een klacht over hun kind. En daar een positieve draai aan weten te geven, zodat je toch allebei weer met een stevige handdruk en een lach op je gezicht de kamer verlaat, dat is wel een hele leuk opdracht ook.

**Een uitdaging.**

Ja. Ooit heeft iemand eens gezegd ‘nou meneer, ik denk dat het u wel gaat lukken, want volgens mij kunt u de meest vreselijke dingen nog met een vrolijk gezicht vertellen’. Dat is ergens een positieve wending aan geven. Dat is de uitdaging.

**Dat is een kracht. Heeft een zorgcoördinator ook wel nodig.**

Ja, zeker. Dat had ik denk ik ook leuk gevonden. Maar goed, zo lopen de dingen en ik ben heel blij met wat ik nu doe, want ik vind mijn werk heel leuk.

**Kun jij iets vertellen over de school? Wat voor kinderen zitten hier?**

Een MAVO-school is over het algemeen wel een afspiegeling van de samenleving. Bijvoorbeeld, omdat in Oosterhout zo’n 15% allochtonen wonen, zitten hier 15% allochtonen. Dat is ongeveer de afspiegeling van de samenleving. De kinderen die hier zitten zijn op zich ‘gewone kinderen’. Wat ik soms een nadeel in het onderwijs vind, is dat je het 97% van de tijd hebt over 3% van je leerlingen. Omdat daar iets mee aan de hand is. De rest loopt gewoon mee in de pas, doet het gewoon goed, dat zijn leuke kinderen. Die anderen zijn ook leuk, alleen die maken iets mee, of hebben een achtergrond waardoor ze zich soms minder leuk gedragen of vaker ziek zijn, of een ongeluk overkomt. Je weet niet waardoor, maar dat is maar een heel klein percentage van het aantal leerlingen waar je het over hebt. Dat zie ik steeds meer. Ik zie ook in vergelijking van de twee scholen waar ik zit, het verschil in de bevolking / de ouders van de kinderen. Doordat het niveau van onderwijs daar lager is (in Tholen) als hier, krijg je ook de minder begaafde ouders binnen en dan zie je dat daar grotere problematiek is. Daar zie je dus veel meer dan hier op de MAVO, dat kinderen niet naar school komen, maar gedekt worden door hun ouders. Zo van ‘nou hij is ziek’. En dan bijvoorbeeld iedere week op vrijdag. En dat kom ik hier veel minder tegen.

Ehm.. We hebben vorig jaar in het project waarin we samenwerken, ook heel erg volgens de regel gemeld. Dat bleek wel dat dat heel veel werd. Daarin hebben we dus gezegd, dat moeten we iets gaan verruimen, want anders geven we elkaar verschrikkelijk veel werk. Daarom heb ik wel het idee dat er in het ziekteverzuim in het algemeen en dan zie ik dat dat hier nog in verhouding iets minder is dan in Zeeland, maar dat we daar wel een probleem hebben. Want we zijn denk ik, een generatie aan het grootbrengen die minder arbeidsethiek heeft dan de vorige generatie. Omdat we er gemakkelijker mee omgaan, omdat we gemakkelijker zeggen ‘ach, blijf maar thuis’.

**En hoe komt dat dan?**

Ehm..

Ouders hebben het drukker. Ik denk dat het op het moment dat je allebei werkt en je zit als vader of moeder in de auto naar je werk, ’s morgens om 7.45 en om 8.00 belt je kind van thuis ‘ja, ik voel me toch niet zo lekker en ga niet naar school’. Dan ben je geneigd bent om het gauw op te lossen en te zeggen van ‘ik bel gauw eventjes naar de school om te zeggen dat je ziek bent’. Dus doordat ouders het drukker hebben, zoeken ze een stukje gemakzucht. Daarnaast zien we ook in andere gevallen, dat ouders de discussie met de kind niet zo snel meer aangaan, omdat de tijd die je samen hebt ‘kwaliteitstijd’ moet zijn. Omdat je het zo druk hebt.

**Dan voel ik een beetje dat je denkt dat ziekteverzuim vooral voorkomt bij ouders die allebei werken. Maar daar is de arbeidsethos juist groot denk ik dan..**

Of de nood om geld te verdienen is groot. Snap je?

Ik geloof niet dat er direct een relatie is tussen het werkende ouder-zijn en ziekteverzuim bij je kind. Ik geloof alleen in het feit dat een kind wat gemakkelijker thuis kan blijven, omdat ouders het druk hebben en er minder zicht op hebben. Kijk, mijn moeder was altijd thuis. Nou zeg ik niet dat dat een goede situatie is, maar mijn moeder was altijd thuis. Als ik ziek thuis was, dan werd dat constant gecontroleerd. Om de zoveel tijd ging er een thermometer in en er kwamen koekjes en thee naar boven. Als ze dan zag dat het een beetje ging, zei ze ‘morgen ga je weer naar school!’.

**Onder de ‘zoveel’ was je dus beter en ging je weer naar school.**

Ja, dat was dus een andere tijd.

Je merkt het ook in andere zaken hè. Ik heb het idee dat in deze tijd ontzettend veel van mensen geëist wordt. Mijn vader heeft altijd kei hard gewerkt, dat vind ik ook, alleen hij lag nog op bed als ik om 7.00 de fiets pakte en naar school ging. Dus hij kwam om 7.30 uit bed, ging naar kantoor toe, kwam tussen de middag thuis eten, deed hij even een dutje en ging ’s middags weer naar kantoor toe en we aten ’s avonds om 17.45 en dan was hij weer thuis.

Dus hij heeft wel hard gewerkt, want hij had ’s avonds ook nog nevenactiviteiten. Om maar aan te geven dat… als ik nu om 7.00 in de auto stap en van 8.00 tot 17.00 bezig ben, ben ik pas weer om 18.30-19.00 thuis. Dat is wel een verschil. De pauzes die je neemt, zijn anders dan toen.

**Er is een ander leefritme ontstaan.**

Het is een ander leefritme. Door alles. Door de informatietechnologie die we hebben. Er wordt denk ik veel meer van mensen geëist dan dat er vroeger van mensen geëist werd.

**In het continuïteit bestek zeg maar.**

Juist, in de tijd.

**Niet in wat je dan doet, maar veel meer in ‘altijd beschikbaar’.**

En in snelheid.

**Wat heeft dat met ziekteverzuim van kinderen van doen?**

Nou, dat heeft te maken met het feit dat er dus minder controle is op de kinderen. Doordat mensen, zoals mijn vader vroeger werkte, heel veel ouders werken nu allebei volledig of in ieder geval parttime.

**Is dat jouw ervaring?**

Ik denk dat er heel veel ouders van kinderen allebei werken of dat moeder in ieder geval (of vader) misschien een deel van de week thuis is, maar er wordt wel heel veel gewerkt door ouders. De nieuwe wijk ‘Vlindervallei’ is schertsend wel eens een sociale achterstandswijk genoemd. Niet zozeer vanwege het feit dat daar kleine huisjes staan en arme mensen wonen, maar omdat het er sociaal een stuk armer is. Je hoorde daar van basisscholen dat een kind echt ziek was en dat ze naar beide ouders gebeld hadden en dat ze door die ouders eigenlijk kwalijk genomen werden dat ze hen belden. ‘Ja, waarom bel je mijn partner niet’ of ‘ik heb eigenlijk geen tijd’. En dat kind bleef daar dan zitten, want ze hadden niemand om dat kind op te laten halen.

Om maar even aan te geven: doordat wij vinden dat we een steeds hoger niveau van leven moeten hebben, dus meer geld moeten verdienen, dat ouders allebei zeggen ‘we hebben ook ons eigen leven, ons eigen werk en ons eigen verhaal’, zeg ik niet dat ze minder van hun kind houden, maar er wel veel minder tijd voor hebben.

**Dan leg je een verband tussen het ziekteverzuim en de oorzaak daarvan vooral thuis?**

Ik leg een verband tussen de controle op ziekteverzuim en de mindere controle thuis.

**Wat is het probleem ‘ziekteverzuim’, wat speelt er dan volgens jou?**

Dat vind ik een hele moeilijke. Ik denk dat er bij sommige kinderen speelt, de bekende drang van de puberteit, zodat je je afzet tegen school, je ouders. Op het moment dat je dus iets doet wat niet mag, dat is spannend en dan doe je dat. Wij reden vroeger van school naar huis en dan was er een clubje en dat ging dat bij de kruidenier waar we langskwamen, kochten iets, maar namen ook iets mee zonder te betalen. Want dat mocht niet en was spannend.

Ik denk dat je een deel van het ziekteverzuim onder deze noemer kunt scharen. Bijvoorbeeld ‘ik heb een proefwerk, laat ik maar niet gaan, want ik heb niet zo goed geleerd’. Of ‘ik wil even iets anders doen’, want er zijn zoveel andere dingen te doen! Als ik ziek thuis was, dan lag ik op bed. Dan had je niets, misschien een pick-up dat je een keer een plaatje draaide. Maar nu kunnen kinderen op internet en zijn er 30 zenders op televisie.

**Het is eigenlijk niet zo erg om thuis te zitten.**

Nee, het is niet erg om thuis te zitten en dat was in het verleden toch anders. Ik denk dat het nu aantrekkelijker is geworden om thuis te zitten. En soms is het zelfs aantrekkelijker om thuis te zitten in plaats van op school te zitten.

**Wat zouden er nog meer voor redenen kunnen zijn om je ziek te melden?**

Je niet geaccepteerd voelen. Pestgedrag van kinderen wat van alle tijden is en op dit moment iets harder lijkt te worden. Het pesten ging vroeger verbaal en nu ook vaak via internet en een mailtje of smsje of tweet waarin iets heel vervelends gezegd wordt, heb je zo verzonden en dan ben je er vanaf. Maar heel veel kinderen beseffen zich niet dat dat ding over de wereld gaat en dat iedereen het kan lezen. Op het moment dat ik iets heel negatiefs tegen jou zeg, zie ik jou reageren en kan ik op dat moment meteen nuanceren. Zo van ‘ja, ik bedoel eigenlijk te zeggen…’. Op het moment dat je een smsje / tweet/ mailtje stuurt, dan zie je die ander niet reageren en komt het veel harder binnen.

**En dan noem je al twee dingen, wat kan er nog meer achter ziekteverzuim zitten?**

Ehm..

Het spijbelgedrag hebben we genoemd, problemen op school / pesten is genoemd.

Het niet geaccepteerd voelen en het pestgedrag hangt natuurlijk ook samen met hoe je in je vel zit. Ik kan me ook voorstellen dat daar ook lichamelijke klachten van komen. Dat je jezelf te dik voelt of te dun of niet mooi genoeg. Dat speelt natuurlijk bij de kinderen van deze leeftijd enorm. Dat is een heel belangrijk item. En als ik dan verder denk…

Zelfs groepsgedrag zoals ‘een vriendje blijft thuis, dus ik blijf ook thuis’, kan ook nog meespelen.

Daarmee wil ik dus niet zeggen dat al het ziekteverzuim dat we hier signaleren allemaal ongeoorloofd zou zijn. Maar ik ben wel geschrokken van de hoeveelheid.

Het sociale karakter van beide ouders kan natuurlijk ook meespelen. In Tholen kom ik dus veel meer dan hier tegen dat inderdaad ouders zeggen ‘mijn kind is ziek’ en dat iedere week. Ik heb een moeder gebeld en gezegd ‘mevrouw, als mijn kind iedere week op vrijdag ziek was, dan zou ik me grote zorgen maken! En ik maak me dus echt grote zorgen over uw zoon. En daar schrok ze echt van. Dat je zegt ‘hij gaat hier zo vaak ziek naar huis en u belt zo vaak op dat hij ziek is’. Als mijn kind zo vaak ziek was, ging ik ermee naar het ziekenhuis en liet ik ze heel erg goed onderzoeken.

**Dat was voor haar een eyeopener.**

Ja, dat was voor haar een eyeopener. Ik heb met haar ook afgesproken dat we dat kind niet meer naar huis sturen als hij zich bij ons ziek komt melden. Laten we dat met elkaar afspreken.

**Waarom moet je als school aandacht hebben voor ziekteverzuim?**

Nou, kijk, wettelijk ben je het verplicht. Een kind heeft leerplicht en daar zijn ouders verantwoordelijk voor, maar wij als school zijn verantwoordelijk om te signaleren. Aan de andere kant vind ik die signalering ook een hele belangrijke. Nogmaals, ik meen het dus werkelijk wat ik net zei. Als mijn kind iedere maand echt een keer ziek zou zijn, zou ik me grote zorgen over mijn kind maken. Want een kind van 12 t/m 16 is niet iedere maand ziek, of er is iets anders aan de hand.

Ik denk dat ik dan zou willen weten wat er aan de hand was.

**Je zegt ‘wij hebben de plicht om te signaleren’. Kun je daar nog iets meer over vertellen?**

Nou, ik heb niet alleen de plicht vanuit de leerplicht om te signaleren, maar ik heb ook de morele plicht om te kijken hoe het gaat met mijn kinderen. Ik bedoel, de kinderen die hier op school zitten, zijn ‘mijn kinderen’. Ik zeg ook wel eens tegen een moeder, en dat komt wel eens verkeerd aan, ‘oh, dus u bent de moeder van een van mijn kinderen’ en dan is de vader ‘not amused’.

Om maar even aan te geven, ik voel het wel zo. Als ik hier door de gangen loop. Er is vorige week een meisje aangereden, waar het gelukkig weer iets beter mee gaat, maar dan is het wel één van jouw kinderen die aangereden wordt. Ik voel een verantwoordelijkheid en je hebt als school een verantwoordelijkheid over het welzijn van onze kinderen die aan ons toevertrouwd zijn.

**Omdat?**

Omdat ouders je een stukje van de opvoeding toevertrouwen. Dat betekent niet dat zij van de opvoeding alleen nog ‘de voeding’ hoeven toe te dienen, maar wij hebben wel een stukje van die opvoeding. Dus ben ik, samen met de ouders, verantwoordelijk voor het stukje welzijn van de kinderen.

**Als je kijkt naar een breder maatschappelijk veld, hoe zie je daar de rol van een school of verantwoordelijkheden van een school?**

Dat is een hele moeilijke. Ik vind wel dat er af en toe teveel verantwoordelijkheden bij de school worden gelegd. Als het ergens over jeugd gaat, zeggen ze ‘dan moeten de scholen dat maar oppakken’. De maatschappelijke stage bijvoorbeeld, dan denk ik bij mezelf, een heleboel van al onze leerlingen zijn al maatschappelijk geëngageerd. Die doen al dingen voor niets voor de samenleving. Laten we dat alsjeblieft meetellen. Maar diegenen die dat niet zijn, die krijg je bijna niet zo ver, omdat ze dat niet mee hebben gekregen van thuis. En dan moet je dus gekunsteld een maatschappelijke stage gaan doen. Dat gedeelte, daarbij denk ik bij mezelf ‘daarin gaan we te ver’. Ik denk dat we maatschappelijk gezien als scholen, toch een stukje de maatschappij van de toekomst aan het vormen zijn. En dat mislukt ook op een heleboel fronten natuurlijk. In de milieus waar het niet zo goed gaat, zie je kinderen dat je denkt ‘die groeit daar bovenuit’, van hele eenvoudige mensen, waarvan de dochter hier op school zit. Dan kom je ze 1 jaar na het diploma toch tegen achter de kinderwagen tegen, dat je bij jezelf denkt ‘hè, het is niet gelukt’. Ik denk dat we toch breed maatschappelijk de opdracht hebben om opleiding te verzorgen, maar ook om een stuk opvoeding te verzorgen. Om zo de nieuwe generatie klaar te maken voor de maatschappij waarin ze aan de gang moeten.

**Dat is een hele mooie, maar ook grote opdracht.**

Ja, dat is een grote opdracht en ik denk niet dat we die altijd redden. Je moet er niet gefrustreerd van raken als het niet lukt. Het is de intentie..

**De ideologie..**

Het is een doel om de kinderen een denkniveau mee te geven, waar ze in het vervolgonderwijs mee verder kunnen. Dat is veel tastbaarder bij wijze van spreken, dan dat grote doel wat ik net noemde, maar het een is onderdeel van het ander.

**Hoe zie jij de rol van jeugdgezondheidszorg? Kun je iets vertellen over JGZ?**

Ik vind JGZ buitengewoon belangrijk, ik vind dat JGZ de taak heeft om iedereen die met jeugd bezig is, te ondersteunen waar het gaat over ziek en gezond zijn. Ook over allerlei jeugdgerelateerde gezondheidsproblemen, om die mede te ontdekken en mede om kinderen en ouders de weg te wijzen naar de reguliere gezondheidszorg als dat nodig is. Ik zie daarnaast ook een stukje controlerende taak, dat is waar ik de JGZ ook erg nodig heb. Als ik me zorgen maak over een kind dat vaak niet op school is, wil ik heel graag dat de JGZ daar naar kijkt en dat men mij daarin adviseert. Bijvoorbeeld met dit kind is ‘dat of dat’ aan de hand, dat moet of de reguliere gezondheidszorg in of misschien heb je als school een oplossing. Het gaat mij niet alleen om het controlerende en het bestraffende vingertje van ‘ouders, u moet uw kind sturen’. Als een kind écht een sociaal-emotioneel probleem heeft en daardoor niet naar school kan komen en het is gerelateerd aan de school, moeten wij van de JGZ de opmerking krijgen ‘het ligt daaraan’ en ‘kunnen jullie op school iets veranderen of verbeteren, zodat het kind wel durft / kan’. Dat vind ik heel belangrijke zaken. Dus daarnaast een adviserende rol, maar ook een rol naar ouders toe om aan te geven ‘hier gaat u te zachtzinnig met uw kind om’ of ‘hier gebeurt iets waarvan we vinden dat we dat als maatschappij niet goed kunnen keuren als u op deze manier met uw kind omgaat’.

De rol is wat mij betreft drieledig. Aan de ene kant een mogelijke doorverwijzende, een adviserende naar het onderwijs, maar ook een adviserende naar de ouders toe. En die adviserende rol, dat is in die rol altijd zo, dat loopt van vriendelijk verzoeken tot wat strenger toespreken. Ik bedoel, dat kan ook wanneer wij als school dingen niet goed doen. Dan verwacht ik dat ook van de JGZ te horen, dat er gezegd wordt ‘school, probeer dit nu eens even tegen het licht te houden, want dat gaat niet goed bij jullie’. En dat is ook naar ouders toe, ‘ouders, dit gaat niet goed bij jullie’.

**En hoe kijk je dan aan tegen de ziekteverzuimbegeleiding, het project MAZL?**

Nou, ik zie dat het project als heel waardevol. Ik denk dat M@ZL de vervanger kan zijn van het reguliere onderzoek. In de 2^e^ klas werden alle kinderen op een bepaald moment opgeroepen en als we dat doorspraken met elkaar, dan kwamen daar enkele dingen uit. Ik ben ervan overtuigd dat diezelfde dingen door M@ZL ook geconstateerd worden. Maar dan onderzoek je alleen diegenen die vaak verzuimen. Ik denk dat de kosten van het ene, de kosten van het andere kunnen dekken. Waardoor je resultaat gerichter gaat werken.

Daarnaast heb ik geen M@ZL op Westerpoort en ik zie daar veel meer ouders die de hand lichten, vind ik, bewust of niet bewust en misschien niet eens altijd verwijtbaar. Maar wel de hand lichten met het ziekteverzuim, dus kinderen te snel ziekmelden*. Het aantal ziekmeldingen wat wij krijgen, is door M@ZL van Effent in kaart gebracht en dat heeft onze ogen wel geopend. Er zaten daar wel ‘schellen’ voor, want het was enorm! Daar zijn wij zo van geschrokken, dat je er nadrukkelijker naar aan het kijken bent. P2e* Ik geloof niet dat wij op Westerpoort zo het ziekteverzuim in kaart hebben als dat we het hier hebben.

**Dat is eigenlijk een neveneffect.**

Nou, ja, ik vind dat eigenlijk een van de hoofdeffecten.

**Wat voor effecten wil je dan van M@ZL?**

Nou, het neveneffect is voor mij een heel belangrijk effect geworden: ik heb nu een beeld en ik heb op mijn netvlies staan hoe het ziekteverzuim bij ons op Effent is, en hoe het zich ontwikkelt. Daarnaast verwacht ik van de GGD, want dat is dan de volgende stap, dan ga je melden. Ik verwacht van de GGD dat ze mij en de ouders en mogelijk / indien noodzakelijk de gemeente, adviseert ‘dit is er met het kind aan de hand en dit zijn de vervolgstappen’. Als het kind écht ziek is, krijgen wij als school een opmerking van ‘luister eens, dit kind is echt ziek’. Maar ik kan me voorstellen dat er, vanuit de gesprekken die met het kind en de ouders gevoerd worden, advisering naar ouders en kinderen is van ‘luister eens, ik zou dit of dat doen’ zoals het doorverwijzen naar een psychiater of naar externe hulpverlening. Ik denk dat dat heel erg goed zou zijn en dat het ook gebeurt.

Daarnaast verwacht ik een terugkoppeling van ‘dit kind kan naar school of dit kind kan niet naar school’, want daar heb ik recht op. De rest hoef ik in principe niet te weten, dat is ook aan de ouders om me dat wel of niet te vertellen. Maar ik verwacht wel van de GGD dat als wij het niet goed doen of dat de problemen schoolgerelateerd zijn, dat dan wél die koppeling gemaakt wordt. Bijvoorbeeld ‘dit kind komt niet naar school, omdat ze bang is van die docent’. Dat soort signalen verwacht ik wel van M@ZL. En vervolgens moeten alle mentoren wel aan de slag met die terugkoppeling van de jeugdarts.

Wat ik ook verwacht heb, en dat is voor mij nog niet helemaal duidelijk, want daar moeten we langer voor in het project zitten. Maar ik verwacht ook een terugloop van het aantal ziektemeldingen op den duur.

**Vanwege?**

Nou, vanwege het feit dat ‘het bij de schoolarts opgeroepen worden’, toch een controlerend verhaal is. Ik kan me voorstellen dat als je, als ouder, heel veel werkt en soms vroeger weg bent dan dat je kind naar school gaat, dus inderdaad in die sfeer komt van ‘ik zit nu in de auto of net op m’n werk en ik krijg een telefoontje van ik voel me niet zo lekker en dan als ouder zegt, ja ik meld je wel even ziek’. Op het moment dat je opgeroepen wordt, omdat het verzuim zo vaak is en de GGD arts vertelt je in het gesprek dat het wel heel veel is, dan vallen bij die ouders de schellen ook van de ogen. Ze zullen daar dan toch omzichtiger mee omgaan denk ik. Gewoon omdat ze dan inderdaad ook zoiets hebben van ‘het is wel heel erg’.

**Er wordt nu aan de keukentafel over dit soort dingen gesproken, hè?**

Maar het is dus ook, vind ik, nogmaals, schokkend als mijn kind 1 keer in de maand ziek thuis is.

**(we gaan verder vanwege de tijd)**

**Hoe is jouw ervaring met jeugdgezondheidszorg?**

Mijn ervaring met de JGZ is in principe heel goed. Het valt of staat wel bij de jeugdarts die aan je toegewezen wordt. Daar hebben wij hele goede ervaringen en minder goede ervaringen mee.

**Wat maakt dat het een goede of minder goede ervaring is?**

Het accuraat zijn van de jeugdarts. Dus het afspraken nakomen, de terugkoppeling. Daarin hebben we het wel eens minder getroffen en treffen we het nu bijvoorbeeld weer goed. Wat voor mij van belang is, is dat we dingen met elkaar afspreken en die afspraken moeten ook nagekomen worden.

We moeten omzichtig met informatie omgaan die we elkaar toespelen. Wij denken ook wel eens dat het thuis niet goed gaat met een kind, we krijgen wel eens signalen die helemaal mis zijn bij kinderen thuis. Op het moment dat we die informatie doorspelen, zijn dat geen feitelijkheden, maar zijn dat veronderstellingen. Op het moment dat veronderstellingen door de jeugdarts dan ook naar ouders worden doorgegeven als zijnde ‘veronderstellingen van de school’, *dan speelt een ouder ons tegen elkaar uit. Dat zijn dingen die we goed moeten doen met elkaar. P_2d_*

**Dat bedoel je ook met accuraatheid?**

**Dat is voor mijn gevoel meer vertrouwelijk omgaan met gegevens, elkaar vertrouwen, afspraken nakomen.**

Ja, oké, dat is voor mij ook accuraatheid. Afspraken die met ons of ouders gemaakt worden, dat die ook nagekomen worden. Daar hebben we in het verleden ook wel eens van gezien dat we een gesprek of terugkoppeling verwachtten, die dan niet kwam. Dat is nu anders, maar dat is wel heel belangrijk.

**Nog meer competenties die je wil zien van een jeugdarts?**

Ja, alle competenties die ik van een jeugdarts mag verwachten. Inlevingsvermogen.

Verder, en dat is moeilijk, want informatie die wij verstrekken is niet altijd objectief (wij kennen een kind ‘van haver tot gort’), moet de arts door de informatie van ouders en leerlingen heen kunnen kijken, maar ook ‘door de informatie van de school kunnen kijken’, zodat je tóch een objectief beeld krijgt van de situatie. Dat is heel moeilijk.

Dat is eigenlijk hetzelfde dat als een leerling uit de klas gestuurd wordt en bij mij voor de deur staat en zegt ‘meneer, ik was het er helemaal niet mee eens dat ik eruit gestuurd werd’. Dan moet ik het verhaal van de leerling aanhoren, ik moet het verhaal van de docent aanhoren en moet voor allebei een oor hebben. Ik moet proberen om de waarheid, die altijd wel ergens in het midden ligt (soms wat meer naar de docent, soms wat meer naar de leerling) en zo zal ik dus het plaatje compleet moeten maken.

**En je niet laten uitspelen..**

En je niet laten uitspelen door de een of de ander. Dus dat is eigenlijk wat ik ook van de jeugdarts verwacht. Kies niet altijd automatisch partij voor de ouders, maar ook niet automatisch voor ons. Kijk en maak je eigen oordeel en doe dat objectief en professioneel. Maar luister wel goed naar de verhalen die wij met z’n allen over dat kind vertellen.

**Want die komen er ook niet zomaar..**

**Nog een belangrijke vraag.**

**Jullie hebben nu M@ZL compleet gekocht; we hebben ook M@ZL smal ontwikkeld (korte uitleg o.a. over criteria en zelf gesprek houden).**

Het gevaar van M@ZL smal vind ik eigenlijk, en dat loopt in het verlengde van wat ik net vertelde, op dat moment bepaalt de school of het noodzakelijk is om een leerling door te verwijzen. Dan krijg je dus de willekeur van de school. Als ik net duidelijk maak dat ik van de jeugdarts verwacht dat die ons verhaal weegt én het verhaal van het kind en de ouders, dan wordt de weging van het verhaal van de school in M@ZL-smal zó belangrijk. De school bepaalt dan of het kind doorgestuurd wordt. Dus je krijgt een willekeur in het doorsturen van leerlingen, terwijl ik de objectieve criteria heel belangrijk vindt. We hebben bijvoorbeeld een chronisch zieke jongen hier die regelmatig verzuimt, ja, dat we die niet doorsturen, dat vind ik een ander verhaal. Dat doen we in overleg en dat bespreek je met elkaar. Maar als ik het ene kind wat zich 4x in een bepaalde tijd ziek meldt, wel door stuur en het andere kind dat precies aan dezelfde criteria voldoet, niet doorstuur… omdat ik daarbij mijn eigen afweging maak, voel ik dat eigenlijk een beetje als ‘ik ga op de stoel van de dokter zitten’. Bijvoorbeeld net als ‘die mevrouw heeft bepaalde klacht en die stuur ik wél door en die mevrouw komt met eenzelfde klacht en die stuur ik niet door’. Waarom maak ik die afweging dan?

Dus ik heb bewust niet gekozen voor M@ZL smal. Niet omdat ik de verantwoordelijkheid van die verzuimgesprekken uit de weg wil gaan, want dat wil ik best doen met de ouders en kinderen, *maar omdat ik niet op de stoel van de dokter wil gaan zitten. Misschien zie ik iets met mijn beperkte medische kennis over het hoofd, wat die dokter niet over het hoofd ziet. P_2a_* Kan die een argument aanvoeren waardoor ouders wel overtuigd raken van het feit dat ze eens met hun kind naar de psychiater of huisarts of wat dan ook moeten. Die afweging kan ik niet maken. De dokter heeft, door zijn titel en studie, een andere blik op dat kind. Die kan ik niet hebben.

**Nu heb je als directeur mogelijk ook verzuimgesprekken met werknemers. Binnen de arbeidsgeneeskunde zeggen ze eigenlijk dat het frequente kortdurende verzuim ‘gedragsmatig verzuim’ is en veel met de verzuimdrempel te maken heeft en de werkgever moet dat zelf kunnen aanpakken. Langdurig verzuim is vaker gerelateerd aan echt ziek zijn en dat hoort bij de arts.**

Ik snap de vergelijking.

**Waarom werkt dat niet denk je?**

Ik voer ook verzuimgesprekken met mijn collega’s, maar heb ook de afspraak met onze arbo-arts dat ik bij frequent verzuim óók doorstuur. Waarom? Omdat ik me kan voorstellen dat, en dat vind ik bij M@ZL eigenlijk hetzelfde, dat als de problemen gerelateerd zijn aan de school of het werk, dan kan ik onderdeel zijn van het probleem. Op het moment dat ik dan met die collega om de tafel zit, heb ik een open gesprek met die collega. Maar op het moment dat ik onderdeel ben van het probleem, zal die dat niet benoemen. Of heeft hij er misschien meer moeite mee om het te benoemen. Als je dan naar de bedrijfsarts gaat en je bespreekt dat verhaal dan met de bedrijfsarts, die het teruglegt bij mij (en zegt ‘het ligt daar en daar aan’). En zegt ‘je kan het gesprek op die manier voortzetten, want je bent geen onderdeel van het probleem’, dan kan ik het gesprek zelf weer oppakken en kan ik er zelf mee door.

**De onafhankelijkheid..**

De onafhankelijkheid van de bedrijfsarts en van de GGD-arts is voor mij een hele waardevolle. Waardoor ik vind dat je sneller moet doorverwijzen. Bijvoorbeeld: ik heb een heel open gesprek gehad met een docent die veel problemen had in de privésfeer en een ouderschapsverlof had van 0.2, waardoor die een dag vrij had. Die andere 4 dagen bleef zij overeind, omdat ze die ene extra dag vrij had, om bij te tanken en problemen kon oplossen in de privésfeer. Toen heb ik haar naar de bedrijfsarts gestuurd om met hem te kunnen overleggen om haar 0.2 ziek te verklaren, totdat die problemen waren opgelost. Als we dat niet gedaan hadden, was ze omgevallen en was ik haar de hele week kwijt geweest.

Op die manier vind ik dat het samenspel zou moeten zijn tussen mij en de bedrijfsarts, maar ook tussen school en GGD-arts. Om te zoeken naar oplossingen om kinderen binnen de school te houden. Lang weg zijn bij arbeids- of schoolgerelateerde problemen, zal de drempel om terug te komen altijd verhogen. En op het moment dat je mensen voor in ieder geval een deel ‘binnen boord houdt’, wordt de weg van huis naar school minder moeilijk, dan wanneer je er een tijd uit bent. En dat vind ik een heel belangrijke taak, ook binnen M@ZL.

De jeugdarts is dus ook iemand die ervoor zorgt de schellen bij de ouders van de ogen vallen en denken ‘dit is een probleem waar we iets mee moeten’. *Als wij dat zeggen, zijn we volgens de ouders ‘de tegenpartij’ die een ander belang heeft. Wij moeten die kinderen binnen school houden. Op het moment dat een onafhankelijke derde, die ook nog arts is, heeft gestudeerd en een bepaalde status heeft bij ouders (alhoewel die, net als de leraren, van hun voetstuk zijn gestoten), er iets over zegt, komt dat anders binnen dan wanneer je dat als onderwijsinstelling doet*. *P_2c_* Daarmee kunnen we proberen om de drempel om naar school te gaan, toch laag te houden.

Ik heb liever dat de GGD-arts, wanneer iemand in psychische nood zit, zegt ‘laten we het eens met halve dagen proberen’, dan wanneer hij helemaal niet meer komt.

**Dan heb je hem nog, alles beter dan thuis zitten.**

(Voorbeeld over collega met psychische nood, niet werkgerelateerd, die huilend binnen kwam dat ze er zoveel moeite mee had om weer op haar werk te komen)

(..) Om te voorkomen dat iemand niet of huilend binnenkomt na een lange tijd van verzuim, moet je mensen binnenhouden in mijn beleving. Dan liever zeggen ‘ik meld je half ziek’, maar kom in ieder geval een aantal halve dagen. Blijf die weg gaan.

**Nog een laatste onderwerp dat ik belangrijk vind, zijn de financiën.**

**Jullie betalen nu voor jeugdgezondheidszorg. Ik zou heel graag jouw mening willen horen over de financiën met betrekking tot de GGD, de gemeente. Hoe zie je dat?**

Ehm…

Ik vind echt dat de gelden die nu gebruikt worden om al onze 2^e^ klassers uit te nodigen voor een gesprek.. dat kost ontzettend veel tijd en geld. Ik zou die gelden heel graag ook willen aanwenden om M@ZL mede te financieren. Ik heb er geen bezwaar tegen om ook mee te betalen, maar we zitten natuurlijk allemaal in zwaar weer. De gemeente moet bezuinigen, wij moeten bezuinigen. Om maar heel even te schetsen hoe het financiële gedeelte binnen een school als deze gaat. Wij krijgen 5000 euro per leerling per jaar en 8500 voor een leerling met LWO. Dat geld komt binnen, daar wordt 10% afgeroomd voor ROC west brabant, 5% voor de entiteit VMBO, nog eens 5% voor ziektevervanging. Dus in totaal wordt er 20% afgeroomd. Dan houden we een bedrag over. Bij dat bedrag wordt bekeken ‘wat is jouw gemiddelde personeelslast’, dat delen we en zoveel personeel kun je aannemen. Naarmate het bedrag van we ontvangen niet stijgt en wél mijn personeel ouder wordt en langer blijft doorwerken, stijgt die personeelslast. Wij zitten op een gemiddelde personeelslast van 67.000 euro op jaarbasis, dat kost 1 FTE het ROC. Althans bij mijn school.

Dat betekent nogal wat. Dat betekent dat je dus met minder mensen die school draaiende moet houden. Ouders hoeven steeds minder mee te betalen, we mogen geen rekeningen mee sturen. Het geld wordt dus minder. De gemeentes hebben ook terugtrekkende reacties als het gaat over ‘nog eens wat mee financieren’. Op dit moment wordt er in de Oosterhoutse gemeenteraad gesproken over de cofinanciering van een aantal projecten, waar M@ZL ook bij zit, waar wij dus ook nog geld van krijgen en waar we M@ZL ook mee kunnen betalen. Dat loopt al van het GGDproject van lang geleden. Wij krijgen 17.000 euro op jaarbasis om dat mee te financieren, maar daar zitten ook nog andere projecten in. Daar zit ook een project in waarbij we binnen de school proberen om leerlingen die uit de achterstandsmilieus komen huiswerk te laten maken en daar ook wat controle op uitoefenen en ze te helpen. Dat bedrag wordt aan meerdere dingen besteedt, maar als het een beetje meezit, zal de gemeenteraad ook voor 2012 zeggen dat we dat bedrag nog krijgen.

**Dat is mooi.**

Dat is even afwachten, maar dat betekent ook weer dat we daar een stukje financiering vinden. Maar ook als we dat niet krijgen, blijf ik M@ZL nog zó interessant vinden dat ik dat wil mee blijven betalen. Maar als ik zie wat het werkelijk kost en wat nu door projectsubsidies en door bijv het VSV verhaal wat er nu in zit.. de gemeente draagt geloof ik niet rechtstreeks bij aan M@ZL. Dus daarin zou ik ook zeggen van ‘de gemeentes moeten wel iets bijdragen’.

**Waarom moet de gemeente dat?**

Omdat als wij hier niets doen, zitten zij straks met de problematiek van mensen die uitvallen in het onderwijs. Ik geloof werkelijk dat als je vroegtijdig ingrijpt bij groot ziekteverzuim, dat kinderen binnen het onderwijs blijven en dat je ook een arbeidsethos ontwikkelt waar ze iets mee kunnen. Als iemand net zo veel ziek meldt als die 10% die zich het meeste ziek meldt hier binnen school, binnen een werkkring in het 1^ste^ jaar, dan wordt hij na een jaar ontslagen en staat hij op straat! Dan hebben gemeentes een probleem. Dus op het moment dat wij hier een arbeidsethos ontwikkelen door mensen zich minder makkelijk ziek te laten melden, zullen ze dat ook later in hun werk op een andere manier doen.

**En daar heeft de gemeente voordeel van.**

Ja, daar heeft de gemeente voordeel van. Dan heb je minder daklozen.. Je kan het heel erg doortrekken en je kunt het nooit bewijzen, dat is het nadeel. Je kunt nooit bewijzen dat er door de werkwijze die wij nu hebben er uiteindelijk minder werklozen komen. Die relatie kun je niet rechtstreeks leggen.

**Nou, er is wel een relatie tussen opleidingsniveau en gezondheid.**

Dat zonder meer.

Maar waar het om gaat is dat het ook een verantwoordelijkheid van de gemeente is. En ik ben ervan overtuigd dat we aan de ene kant in de JGZ..

Nou… Voor de basisschool vind ik het overigens wel een goede zaak, dat ik met mijn kinderen van 4 jaar bij de schoolarts ben geweest en in groep 7 nog een keer. Alleen daar gaat het om een aantal dingen, zoals gehoor en gezicht en allerlei gezondheidsaspecten. Die zijn al ontdekt op het moment dat kinderen naar het voortgezet onderwijs gaan. Op dat voortgezet onderwijs, als het dan gaat over iets wat nog ontdekt moet worden door de jeugdarts, wordt dat ook ontdekt door het veelvuldig ziekteverzuim. Want dat heeft dan rechtstreeks met elkaar te maken. Dus ik zou zeggen ‘gemeente, alsjeblieft, besteed geen geld aan dat periodiek onderzoek voor kinderen op het voortgezet onderwijs, maar besteed dat geld nu aan alle kinderen’. Het periodiek onderzoek heeft betrekking op één leeftijdsgroep. Het grote voordeel van M@ZL is dat alle kinderen van 12 – 16 / 18 jaar op deze school door de gaten worden gehouden. Op het moment dat er een probleem is dat zich openbaart in de 3^e^ klas van het voortgezet onderwijs, dan krijgen we dat nog in beeld. Terwijl dat kind al naar het periodiek onderzoek is geweest en het daar nog niet in beeld gebracht is. Wat dat betreft zeg ik dan dat M@ZL integraal invoeren in Nederland veel beter is dan het periodiek onderzoek in de 2^e^ klas.

**Ook om de gezondheid beter te monitoren zeg je eigenlijk.**

Juist, ja. Het monitoren om dingen te ontdekken die ook op latere leeftijd optreden, om gezinssituaties die veranderen.. en er gebeuren een hele hoop dingen waardoor er weer gezondheids- of psychische klachten ontstaan bij kinderen. Om die te kunnen monitoren hebben we veel meer M@ZL nodig dan dat periodieke onderzoek. En ik denk dat de kosten van dat periodieke onderzoek én dat van M@ZL, dat die vrij overeenkomen. En hetgeen wat dan niet overeenkomt, moeten wij als scholen dan maar bijbetalen.

**Je hebt ontzettend veel vertelt, dankjewel. Ik wil afsluiten gezien de tijd.**

**Wat ik jou nog wil vragen is: wat wil je ons als onderzoekers nog meegeven nu?**

Ik zou jullie mee willen geven: richt je onderzoek in ieder geval ook op de vergelijking van dat periodiek onderzoek en de resultaten van M@ZL. Dat is iets wat ik heel belangrijk vind. Bewijs alsjeblieft dat de zaken die uit M@ZL komen, relevanter zijn dan de zaken die uit dat periodieke onderzoek komen.

Want als je dat bewijst, heb je een stok om mee te slaan als je zegt ‘laten we dat periodieke onderzoek afschaffen en laten we gelden vinden om M@ZL te laten bestaan’. *Ik ben ervan overtuigd dat een leerling die in een gezinssituatie zit waar bijvoorbeeld ouders gaan scheiden of er sprake is van een overlijden van een van de ouders, dat een kind klachten kan ontwikkelen, die we met M@ZL vroeg ontdekken* *P_2b_* en door M@ZL kunnen doorsturen voor verdere behandeling, waardoor zo’n kind niet in psychische nood komt. Dit kind in de 4^e^ klas krijgt van ons wel ondersteuning, maar niet de medische ondersteuning die hij misschien nodig heeft. En het periodiek onderzoek is dan dus al voorbij. Dat is iets waar ik van zeg ‘dat soort dingen zouden we naast elkaar moeten zetten’. Ik zou jullie als onderzoekers adviseren dat naast elkaar te zetten, om te bewijzen dat M@ZL in die zin gewoon een hele grote meerwaarde heeft.

Plus het feit, en natuurlijk zeggen mensen ‘ja, er komt maar een kleine groep en die grote groep dan?’. De grote groep die mee hobbelt en het gewoon goed doet, daar hoeven we niet in te investeren. Ik weet wel dat er vaak gezegd wordt ‘we besteden zoveel geld aan een kleine groep mensen’.. dat zal altijd zo blijven. Want gelukkig besteden we een hoop van het medische geld ook maar aan een kleine groep mensen, want anders waren we allemaal ziek.

En we zijn natuurlijk niet allemaal ziek, gelukkig! Daarom zeg ik, dat is voor mij eenzelfde verhaal. Want iemand die die hulp niet nodig heeft, moet er ook niet mee vermoeid vinden vind ik.

**Als ik terug mag komen op het woord ‘relevanter’, wat je direct na het stellen van mijn vraag zei, dan associeer ik dat met het feit dat je ‘andere problematiek, relevantere problematiek’ signaleert. Dat hoor ik daarna niet meer terug.**

Nou, ehm..

Zo bedoel ik het wel. Kijk, op het moment dat iemand zich ziek meldt, is er iets aan de hand. Dan kan het zijn dat het spijbelgedrag is, maar ook dat er psychische nood is (“psychisch is ook écht”) of dat er ‘echte’ / lichamelijke gezondheidsklachten zijn. Op het moment dat een kind van 14 jaar bij de GGD-arts komt, dan kan een gezin waar het niet goed mee gaat, kan ‘faken’ dat het wel goed gaat. Dat bedoel ik eigenlijk te zeggen. Bijvoorbeeld: Ik doe vreselijk mijn best als ik met mijn dochter bij de GGD kom, dat het allemaal goed gaat, maar ondertussen ram ik ze iedere dag in elkaar’.

**Dat is denk ik inderdaad zo. Je benadert ze op een ander moment.**

Op het moment dat ik iedere dag mijn kind zou slaan, om het maar even in het ridicule voort te zetten, dan zal mijn kind verzuimen van school, omdat het daardoor verwondingen of blauwe plekken heeft die ze niet wil laten zien. Op het moment dat ik een afspraak heb met de GGD voor het periodieke onderzoek, kan ik zorgen dat ik daar met mijn kind naartoe ga en dat de GGD-arts dat niet merkt. Terwijl dat als een kind regelmatig met fysiek geweld te maken heeft en daardoor verzuimt, komt het wél aan de orde.

**Het kan het ook aan de orde laten komen, juist dóór te gaan verzuimen.**

Ook dat.. het kind kan dan zelfs op dat moment zelf actie ondernemen! Zelfs dat is waar, ja. Daar had ik niet eens over nagedacht.

**Je kan zelfs als kind dus een gesprek ‘afdwingen’, door jezelf ziek te gaan melden.**

Ja, en zeker wanneer het dan gaat over de 15/16/17 jarigen binnen het voortgezet onderwijs, die zouden ook op dat idee komen, die zijn daar oud genoeg voor. Een kind van 12/13/14 niet.

*“Off the record”*

Nog een voordeel van M@ZL:

Vóór de start van het project was het moeilijk aan ouders uit te leggen als je acties wilde ondernemen ivm het ziekteverzuim. Nu kun je heel duidelijk aangeven dat er een onderzoek/project als M@ZL loopt en zeggen dat het kind voldoet aan de criteria en dus naar de jeugdarts moet.

**Interview 3. M@ZL onderzoek Datum: 16-12-2011**

**Aanwezig: Yvonne Vanneste (onderzoeker), Marlou van de Loo (semi-arts)**

**Directeur school 1 / P_1_**

**Algemene gegevens**

Op school, kamer directeur.

Eerste kennismaking

Verloopt erg goed, ontspannen sfeer.

Idealistisch, enthousiast

Je merkt dat het interview op het begin al wat makkelijker verloopt, omdat dit (al) het 3^e^ interview is.

De directeur heeft een erg sterke mening over bepaalde zaken.

Wat muziek op de achtergrond (die soms wat storend is naar mijn idee)

Tijdens het laatste gedeelte ontstaat er bijna een discussie over de rol van de jeugdarts en het aanwezig zijn van de medische expertise. Het is moeilijk om je daar buiten te houden, omdat je zelf natuurlijk ook zo lang in die functie hebt gezeten en wordt ‘aangevallen’.

**Verslag interview**

**Vragen/opmerkingen door interviewster dikgedrukt**

Antwoorden/opmerkingen door geïnterviewde in normale opmaak

**Kunt u zich voorstellen?**

Uiteraard kan ik dat. Ik ben A.v.V., ik ben interim directeur hier op Prinsentuin. Ik zit hier vanaf mei 2010, nu zo’n anderhalf jaar. Daarvoor heb ik Van Kooten gedaan, daarvoor de middelbare Prinsentuin en daarvoor heb ik Andel gedaan, VMBO. Dus ik hop van de een naar de ander. Na de middelbare hebben we binnen de ROC een afspraak gemaakt dat ik “interim-werk” zou gaan doen, maar eigenlijk is anderhalf – twee jaar best lang voor een interim directeur. Maar wil je wat aan de kwaliteit doen binnen een school, dan moet je er net iets langer zijn dan gewoon een seizoen. De meeste dingen zijn natuurlijk al ingezet tijdens een seizoen en tijdens de rit maken we de verbeteractie voor volgend jaar.

Nou, dat is aardig gelukt tot nu toe hier. De kwaliteitskaart is weer op orde, het leerlingenaantal is weer op orde.

**Er zijn weer meerdere leerlingen aangemeld, want jullie liepen terug?**

Ja, we liepen fors terug. We hadden toen ik hier kwam 92 aanmeldingen en nu hebben we er 150. En ik denk dat we dit jaar de 170 wel halen.

Er zitten intern natuurlijk ook een hoop dingen waar we aan moeten sleutelen.

**Werk genoeg.**

Meer dan werk genoeg, maar wel leuk.

**Ik wil beginnen met een algemene vraag.**

**Welke redenen zou een school hebben om iets met ziekteverzuim van leerlingen te doen?**

Primair natuurlijk om de leerlingen bij het onderwijsproces te houden. Als ze er maar even tussenuit zijn, kan dat, iedereen is wel eens een dag ziek. Geen probleem. Maar degenen die iets langer ziek of afwezig zijn; ziek en afwezig zijn natuurlijk twee verschillende zaken. Maar het is hetzelfde als je zelf werkt en je zou niets horen van je werkgever als je een week ziek bent, dan heb je ook zoiets van “nou, ze missen me niet, ik blijf nog maar een paar dagen weg”. Het is zaak om er kort op te zitten en gelijk aan te geven van “joh, we hebben je gemist! Waar was je? We hebben je nodig. Kom.”. En om zo snel mogelijk leerlingen weer op school te hebben en als het langer is, dat je ze van thuis uit bereikt, dat als ze weer terug komen de achterstand niet te groot is, waardoor ze weer snel in het proces opgenomen kunnen worden. Dat zou mijn reden zijn.

**Dat zijn eigenlijk twee redenen, voel ik een beetje. Sowieso persoonlijke aandacht voor een leerling, maar ook zo snel mogelijk weer terug komen, zodat ze zo min mogelijk missen.**

**Zijn er nog meer redenen om aandacht aan ziekteverzuim te willen besteden?**

Nou, ik kan natuurlijk allerlei bijkomende oorzaken noemen. Als ze te lang weg blijven, verandert er financieel ook iets, maar dat vind ik niet zo belangrijk. Je bent met die leerlingen het avontuur aangegaan en ouders hebben het vertrouwen gegeven aan de school. Dan vind ik ook dat je daar ook voor moet staan en moet zorgen dat je alles in het werk stelt om ze weer zo plezierig mogelijk, maar ook kwalitatief goede periode te geven en te zorgen dat het afgerond wordt hier.

**Hoe kijkt u tegen het project M@ZL aan?**

Ik denk daar wel een beetje wisselend over. Enerzijds vind ik het natuurlijk goed dat we met elkaar afspraken gemaakt hebben. Dat klinkt zwart-wit, maar dat ben ik nu een beetje. Lik-op-stuk, dus bij zoveel keer te laat of zoveel keer afwezig in een voortschrijdende periode, geven we dat door. Vervolgens komt de actie van de GGD of van de jeugdarts of van de leerplichtambtenaar. Ik denk dat dat een prima uitgangspunt is, zo moet het zijn. Daar ben ik heel tevreden over, over die afspraak. Ik vind het nog wel een veel te korte schakel. Want er zitten natuurlijk veel meer instanties rond die kinderen, waarvan ik denk ‘hoe is het toch mogelijk dat we allemaal op die eilandjes werken’. Daar wil ik straks nog wel wat over vertellen.

Het staat of valt natuurlijk in eerste instantie bij de registratie van de collega’s. Als ik eerlijk ben, kost dat nogal wat moeite hier om dat goed voor elkaar te krijgen. Alle collega’s hebben een computer en kunnen live de absentie invullen – dat is allemaal het probleem niet en dat gebeurt ook wel. We zetten het ook elke keer in de info en zeggen dan “M@ZL moet, M@ZL moet”. Eens in de week geven we zo’n info en als we zien dat het project weer afzwakt, staat het iedere keer weer in die wekelijkse info. We noemen het dan M@ZL moet en dan weet iedereen weer wat we bedoelen. Groot probleem is het natuurlijk bij vakken als bewegingsonderwijs of praktijkonderwijs, die met groepen zitten of samengestelde groepen of keuzevakken enzovoorts. Wil je het dan sluitend hebben, dan is het natuurlijk noodzaak dat die collega’s die absenten goed doorgeven. Die komen met een briefje bij de administratie, moeten ze het inkloppen. Eigenlijk moeten ze er dan al onder de naam van een collega in, want anders krijgen ze geen totaaloverzicht. Want mijn management rapportage uit M@ZL wil ik gewoon sluitend hebben. Dat is een hele job, dat krijgen we op de een of andere manier niet goed voor elkaar. Dat is echt lastig.

**Hoe komt dat dan?**

Omdat de administratie al die dagstaten of uurstaten… de collega’s moeten ten eerste heel consequent zijn in het inleveren van die briefjes en dat lukt niet altijd, laten we het maar voorzichtig zeggen. Anderzijds, als je een totaaloverzicht wil hebben van zo’n collega – het uitgangspunt is het verzuim van die individuele leerling, dat is duidelijk – maar als een praktijkdocent 2 uur niet ingevuld heeft en vervolgens maak je een uitdraai van zo’n leerling. Als hij de eerste twee uur afwezig/aanwezig was en je gaat dan rapporteren en je geeft door ‘het is 16 uur geweest, terwijl het er maar 8 waren of andersom’, dat is natuurlijk een drama. *Dat moet natuurlijk sluitend zijn. Zeker op het eind van het traject. Als de leerplichtambtenaar een keer met de cijfers komt en het zou zo ver komen dat het bij de rechter komt en je gegevens zijn niet goed, dan zegt zo’n rechter ‘hup, wegwezen, zorg eerst dat je je huiswerk goed hebt gedaan’ en dan kijken we weer verder. Als je maar even kan weerleggen dat zo’n leerling wel op school is geweest, dan voelen leerling en ouders zich overwinnaar. P_1b_* En denken ze ‘zie je nu wel, niets aan de hand’. Dan is het klaar met de maatregel. Nu is het niet de doelstelling om de leerlingen voor de rechter te brengen, dat is niet wat ik zeg, maar het moet gewoon kloppen.

Om dat kloppend te krijgen, daar hebben we best moeite mee. We zullen wel de enige zijn.

**Nee hoor, jullie zijn zeker niet de enige. Jullie zijn daar wel heel eerlijk in. Ik zie het overal, het is een heel groot probleem om dat goed te krijgen.**

Daar zouden we natuurlijk met een paar mensen wat beter naar moeten kijken. Dat systeem mag natuurlijk niet het doel op zich zijn. Ik denk dat daar wel wat aan veranderd kan worden, waardoor je de registratie wat anders kan doen. Dat het wat simpeler gaat en dat die management rapportage uiteraard ook wat effectiever is. dat moet sluitender kunnen dan dat het nu gebeurt. Dat is in de oplossingsgerichte zin in het vervolgtraject. Daar zou ik wel eens met iemand naar willen kijken.

**Wij hebben in er in ieder geval voor gekozen om de ziekteverzuim omvang niet op uurniveau te gaan meten, omdat dat niet betrouwbaar is. Binnen de arbeidsgeneeskunde is het ook zo dat ‘een ziektemelding op 1 dag, is 1 dag ziek’. Als je zo gaat rekenen ben je heel eerlijk. Of je nu het 4^e^ uur naar huis gaat of gewoon helemaal niet komt, je bent gewoon ziek gemeld.**

Dat systeem geeft nu anders aan. Maar daar moeten we een andere keer naar kijken.

**(Uitleg over nieuw systeem, met 1 druk op knop zie je zo leerlingen die aan criteria voldoen. Advies om intern te kijken of het aangepast kan worden. Daarmee hebben we nog niet opgelost dat de docenten goed registreren.)**

**We zijn begonnen met de redenen die de school kan hebben om ziekteverzuim aan te pakken, daarna hoe u tegen M@ZL aankijkt. Toen begon u eigenlijk met iets positiefs te zeggen, maar.. ?**

Het positieve is natuurlijk dat je eenieder bewust hebt van de noodzaak. Dat is een. Twee is natuurlijk de link naar de jeugdarts en de leerplichtambtenaar is kort. Je hebt een gezamenlijke afspraak over het melden. Voorheen was het vaak zo ‘goh, een leerling is al zo lang aanwezig, laten we hem maar eens gaan melden’. Nu heb je natuurlijk een criterium afgesproken met elkaar over het moment dat je gaat melden. Dat is natuurlijk een grote winst die we samen geboekt hebben, dat zonder meer. Het is wel even wennen geweest in het traject, bijvoorbeeld als er weer iemand werd opgeroepen waarvan we zeiden ‘tja.. ‘. Maar nu geven we ook naar de ouders aan, bij de informatieavond voor de 1^e^ klas, ‘luister, zo doen we dat, dit zijn onze spelregels’. Dan is er ook geen misverstand meer over. We hebben ook heel duidelijk aangegeven dat de communicatie naar ouders hierbij heel belangrijk is. Als je dat nalaat, dan ….. En in het begin was het ook zo dat er ouders een brief kregen van MP dat ze werden uitgenodigd op het spreekuur. Als wij op school dat contact nog niet hadden via mentor met de ouders. Dat moet natuurlijk eerst, al halverwege, voordat ze aan de criteria voldoen, moeten wij natuurlijk onze zorgplicht hebben.

**Dat zou wel netjes zijn.**

Dat lijkt me wel. Dat is even wennen geweest en nu doen we dat anders. Ik bedoel, nu geven we de eerste paar keer telefonisch aan, vervolgens schrijven we een brief dat ‘als het zo doorgaat, we moeten melden’. Dan weet iedereen waar je aan toe bent. Dat loopt nu wel. Ik krijg er nu geen telefoontjes meer over.

**Kreeg je er telefoontjes over?**

Ja, van ouders die echt zeiden ‘ben je nu helemaal gek geworden’. ‘Ik krijg hier een uitnodiging, wat een ding, wat is dit nu toch voor flauwekul, u weet toch dat ze gewoon ziek is. Ik ben toch zo vaak met mijn dochter voor onderzoek weg geweest. U weet toch dat ik naar die arts moet.. waarom moet ik nu in een keer komen opdraven’. Daar waren ze echt boos over. *Dan denk ik dat zij vinden dat er in hun privésfeer een beetje gerommeld wordt. En dat ze als ouder een beetje een ‘brevet van onvermogen’ krijgen. P_1a_* Nu is dat gewoon geaccepteerd.

**Kon u dat op dat moment uitleggen?**

Ja, natuurlijk krijg ik dat wel uitgelegd. Maar op het moment dat je als school niet uitlegt wat je gaat doen en wat de afspraken zijn, dan gaat dat natuurlijk fout.

**Die communicatie is ontzettend belangrijk, hè?**

Ja, precies. Dat moet gewoon goed. Nu weet iedereen dat. Ik sprak deze week nog een meisje en ik vroeg aan haar ‘geef mij je rapport is’. Dat was niet zo denderend en ik vroeg hoe het kwam. Ze zei ‘ik ben vaak afwezig geweest’. Ik vroeg ‘ooh, hoe komt dat dan’. Ze zei ‘ja, een beetje gespijbeld’. Dus ik zei ‘is dat nu afgelopen’. Ze reageerde ‘ik heb mijn lesje nu wel geleerd, want ik ben bij de leerplichtambtenaar geweest enzovoorts en ik ze hebben me goed verteld hoe het zit’. Dat was eergisteren, dus dat is natuurlijk heel effectief. Zo moet het zijn.

**Communicatie met de ouders is ontzettend belangrijk. U zegt ‘ik hoor er niet meer van’. Als ik kijk naar de opkomst van ouders op de GGD, loopt die achteruit. Op dit moment komt maar de helft van de ouders komt op de oproep. Hoe kijkt u daar tegenaan?**

Ik moet dat intern eens bespreken. Ik weet niet of die terugkoppeling naar ons ook komt dat die ouders niet zijn verschenen. Wij zullen dat vanuit ons moet doen en die afspraak moeten maken dat de leerplichtambtenaar dan natuurlijk in actie komt. Van ‘mevrouw of meneer, u heeft daar maar te verschijnen’. We zullen de gelederen moeten sluiten, want anders heeft het geen nut.

**De cirkel moet rond zijn.**

Ja.

**U heeft reacties van ouders, heeft u ook reacties van kinderen? Hoort u daar iets van?**

Ja, de kinderen weten wel dat ze goed gemonitord worden enzovoorts en die kinderen spreken dat ook wel met elkaar door. Van als je teveel mist ‘dan wordt je opgeroepen hoor!’, ‘dus kijk maar uit’.

**U hoort dat wel?**

Ja, dat weten ze wel.

**Wat denkt u dat de redenen die achter ziekmeldingen kunnen zitten?**

Dat is natuurlijk heel divers.

**Bij deze school, deze ouders, deze kinderen. Wat denkt u dan in eerste instantie?**

Nou, wat je heel vaak ziet is dat de thuissituatie dusdanig is, dat er geen controle is op de kinderen, of ze er nu wel of niet zijn. De ouders hebben hun handjes vol met zichzelf en weten maar amper, en dan zeg ik het heel zwart-wit, of de kinderen wel op tijd naar school gaan en of ze er wel of niet zijn.

Kijk, je kan ook de school verwijten dat je het nooit doorgegeven hebt, als die enkeling die aan het spijbelen is, ‘waarom weten wij dat niet als ouders’. ‘Het zal toch niet waar zijn dat een van mijn kinderen 3 weken over straat zwalkt, dat ik dat niet weet’. Het is maar net welke invalshoek je kiest, natuurlijk. Ze kunnen het heel geraffineerd doen en dat zal allemaal waar zijn, maar er zijn natuurlijk veel kinderen… Daar ben ik van geschrokken, dat is hier het goede woord. Dat er hier toch een hoop kinderen zitten met sociale problematiek van thuis uit. Er zitten er echt veel meer dan ik verwacht had. Het is allemaal wel een leuk, rustig schooltje, zo het oogt, maar er zit toch een hoop problematiek hoor. Van ouders na een scheiding, ‘schering en inslag’, die woont bij die en dan zitten ze weer daar.. die heeft weer geen contact met zijn kind. Dan mag je als intermediair optreden, wat wij natuurlijk niet kunnen, maar wat ze wel aan ons vragen. Die weten gewoon niet waar hun zoon of dochter uit hangt. Dat is natuurlijk wel dramatisch als je in zo’n onveilig nest opgroeit.

**Dan zegt u, als ik het kort mag samenvatten, ‘de controle mist’. We weten eigenlijk niet waar de kinderen zijn. Maar ziekmelding moet toch door de ouders gebeuren?**

Ja, maar die zullen wel een keer ziek melden, maar als je na 2 dagen beter bent, dan moet je de 3^e^ dag weer naar school, maar dan kun je ook niet gaan… Maar ook doordat de kinderen op verschillende adressen zitten en ouders stoeien over ‘wie is nu het aanspreekpunt’, dan bel je naar de gezaghebbende. Vervolgens zeggen ze, ‘nou, hij zit bij m’n ex’ of ‘niet te traceren, ik weet het gewoon niet’. Dan moet je weer gaan kijken bij die ex, die dan zegt dan weer ‘ik heb m’n handjes vol met die’.. Het contact met de ouders..

**Dus van de thuissituatie en contact met de ouders zegt u, dat heeft zeker iets te maken wat achter ziekmelding kan zitten.**

Dat is volgens mij het grootste probleem.

**Wat zou er nog meer achter kunnen zitten?**

Ons onderwijssysteem leent zich natuurlijk gewoon voor een aantal kinderen niet. We zijn dan voor hun saai. Als het niet aantrekkelijk genoeg is, blijven de kinderen natuurlijk weg, dat is heel simpel.

**Aantrekkelijk genoeg? Zouden er andere zaken op school kunnen spelen bij ziekmelding?**

Pesten, niet fijn voelen, niet veilig voelen. Noem de factoren allemaal maar op. Die zitten er allemaal wel in. Als je elke dag gepest wordt, dan blijf je vanzelf wel thuis. Dan zou ik het ook ontlopen. Dan zijn wij nog relatief een veilig schooltje, denk ik te kunnen zeggen met alle ervaringen die ik heb op andere scholen waar ik heb rondgelopen. Ja, ik denk dat dit een relatief veilige school is voor de kinderen.

**Hoe komt dat?**

De onderlinge band, de saamhorigheid, de sfeer in de klas en op school zelf. Natuurlijk wordt er gepest, natuurlijk worden er discussies gevoerd buiten. Maar als ik zo de sfeer, en hoe de kinderen zijn en hoe de betrokkenheid van mijn collega’s is naar de leerlingen. De afwerking en de doorstap is een ander vraagstuk, maar er zijn natuurlijk veel scholen waar ik binnen geweest ben, waar collega’s buiten de lessen, ‘hup naar de koffiekamer lopen’, langs de leerlingen heen lopen. Als er hier eentje sip kijkt, is hij al drie keer aangesproken met de vraag wat er aan de hand is. Dan is de afwerking onvoldoende, daar hebben we nog een hele slag te maken, maar de zorg daaromheen, de betrokkenheid en de interesse is er wel zeker.

**En dat maakt de school veilig en prettig.**

Dat is wel de eerste stap denk ik. Dat ze die periode dat ze hier zijn, zich in ieder geval geborgen voelen. Als er eentje zegt dat hij gepest wordt, dan loopt er allicht een collega mee die zegt ‘we gaan samen kijken en ik zorg ervoor dat je in ieder geval veilig op weg bent naar huis. Die zorg is er allemaal.

**Wat maakt dat nu dat deze school zo veilig is? En zo veel betrokken docenten heeft?**

Dat is wel een container, maar ik denk dat er ten eerste een zelfde soort publiek op deze school afkomt. Ik bedoel, we kunnen dat verzwijgen of noemen, we zijn natuurlijk een ‘witte school’. Waardoor je niet het plezier hebt van alle inbreng van allerlei culturen, maar je hebt ook de nadelen er niet van. Dat brengt dat toch met zich mee. ‘Onbekend maakt onbemind’, dat hele verhaal.

**Het geeft wat rust.**

Dat is heel duidelijk. Daarnaast komen er natuurlijk leerlingen van veraf en die brengen de problemen van het weekend niet mee de school in. Als je een school hebt, waar leerlingen allemaal uit dezelfde wijk komen en elkaar op straat of bij de discotheek zien of wat dan ook. Hier moeten ze allemaal nog een uur op de fiets, in ieder geval een groot deel 30 – 45 minuten. Die stappen op de fiets, gaan naar huis, hebben daar hun privé-ding en ze komen maandag weer in de klas. Je hebt dan niet een massa leerlingen die als vriendenclub daar zitten. Dat is van de andere kant wel een nadeel voor die leerlingen, omdat het geen echte vriendenclub is. Maar omdat ze van veraf komen, komt het stoeipartijtje uit de disco hier niet mee naar binnen op maandag.

Dat is een heel ander stuk. Een ander deel daarvan is dat ze zich ook alleen voelen, want ze hebben natuurlijk weinig sociale dingen. Ze moeten de contacten met leerlingen van de basisschool aanhouden, want van elke basisschool komen er maar 1 of 2 hier. Als je met 8 of 9 leerlingen van één basisschool komen, dan heb je al herkenning en dan zitten ze hier met elkaar van ‘yes, hup, wij zijn de grootste club’. Dat hele sociale stuk ligt heel anders in deze school. Met alle voors en tegens. Maar dat heeft natuurlijk een andere input.

**Het is een agrarische opleiding. Maakt dat nog wat uit?**

Nou, kijk, 30% van onze leerlingen gaan maar door in die agrarische sector, dus het is meer de omvang en veiligheid en het ‘wit zijn’. Ik zeg wel eens gekscherend dat die meiden hier op school komen omdat ze een ‘verzorgpony’ hebben en op het moment dat ze verkering hebben, gaat de pony eruit en gaan ze wat anders doen. Zo simpel is het ook.

**Terug naar M@ZL. Waarom bent u niet zo enthousiast over M@ZL?**

Nou, dat is wat ik net aangegeven heb. Het registreren, de management rapportage. Maar we gaven net aan dat dat nu al op ziekmelding is en dat stelt mij al wat gerust, dat had ik nog niet meegekregen.

Maar het is natuurlijk nog maar een klein stuk wat we daaraan doen. Kijk, de hele zorg rond de leerling dat vind ik een heel ander vraagstuk. Daar heb ik met ‘jan en alleman’ al rond de tafel gezeten, maar we zitten met de zorg rond de leerling, op allemaal eilandjes. Dat maakt het lastig. Ik bedoel, als collega’s die zeggen ‘het loopt allemaal niet zo lekker, ga jij maar naar schoolmaatschappelijk werk (SMW)’. Daar vindt natuurlijk op dit moment ook van alles plaats. De een komt en de ander gaat, dus er is geen continuïteit. Het zijn allemaal goede mensen, maar als je een relatie opgebouwd hebt met iemand als leerling en vervolgens heb je dan bij SMW binnen een paar maanden weer een ander.. dat geeft geen vertrouwen in de volwassenen. Heb je net je verhaal in vertrouwen verteld en het gevoel dat iemand je gaat helpen. Vervolgens heeft die weer een andere baan en moet je je hele verhaal weer opnieuw vertellen. Die gaan natuurlijk niet weer naar de SMW. Dan heb je nog Centrum voor Jeugd en Gezin (CJG). Daar heb ik vorig jaar over afgesproken met MP vorig jaar afgesproken dat het goed zou zijn, omdat wij de vindplaats zijn van hetgeen waar zij ‘hun core-business in hebben’, kom nu eens op ouderavonden en zorg dat je hier je loket hebt. Dan is het een kleine stap voor ouders om na elk tien-minuten gesprek binnen te lopen, als je anoniem in een lokaal zit. Ouders lopen dan binnen met hun opvoedingsvraagstuk. Aan de ene kant kunnen zij zeggen dat ze iemand kunnen doorverwijzen, of ze zeggen ‘nou, daar zou ik me niet al te druk over maken, dat hoort bij de puberteit’. Net wat. Maar wij zijn natuurlijk ook maar ‘kruideniers’. De individuele wiskundedocent of directeur. De hulpverlening is mijn vak ook niet. Ik weet er wel iets van, maar dan houdt het wel op. Dus daarin hebben we nog wel een hele weg te gaan, voordat we ook met elkaar de neuzen dezelfde kant op krijgen. Met dat passend onderwijs zullen we wel een stapje verder komen, maar al die instanties, ik word er soms helemaal gek van. Dat werkt gewoon niet samen.

**Nu hebben we bij ziekteverzuim samenwerking met de GGD. Hoe gaat dat? Wat hoort u daarvan?**

Daar ben ik niet zo enthousiast over, nee.

**Hoe komt dat?**

Dat is wat ik zeg. Kijk, de terugkoppeling of de tijd die de collega’s krijgen/hebben of de interesse van enzovoorts.. op een gegeven moment geven collega’s het over. Die zijn bezig met hun klassenmanagement. Een hele goede mentor blijft zo’n leerling ook wel volgen naast datgene wat hij binnen de school doet, maar op een gegeven moment raak je ook de weg kwijt.

Als je niet meer teruggekoppeld krijgt. We hebben alles zo dichtgetimmerd met privacy-gegevens enzovoorts, dat niemand meer iets teruggeeft van wat hij doet met zo’n leerling. We raken, zodra ze in de hulpverlening zitten, het spoor bijster. Dan weten we het ook niet meer. Dan kan hij wel bij een psycholoog lopen, die kan natuurlijk een heel ander traject met zo’n leerling uitzetten als wij. Als wij misschien het idee hebben ‘dat hij gewoon achter zijn broek aan gezeten moet worden’, terwijl de oorzaak heel ergens anders ligt en die psycholoog misschien het idee heeft ‘nou, het zou juist goed zijn als de druk er eens af gaat’. Maar als we tegenstrijdige belangen of adviezen / inzichten hebben, dan komen we niet verder.

**En u heeft het gevoel dat dat niet goed wordt teruggekoppeld?**

Nou, dat is geen gevoel, dat is volgens mij wel een feit. De wet op privacy geeft ook aan dat we niet alle gegevens van een behandeling mogen krijgen. Kijk, ik heb wel eens vaker gezegd ‘het is toch van de gekke dat wij trainingen hebben over sociale vaardigheden of wat dan ook, waarbij we goedbedoelde collega’s, betaald in schaal LC, 3 dagen naar een cursus sturen en die dan denken ze dat ze sociale vaarhigheidstraining of faalangst-reductie training kunnen geven. Ja, ik bedoel, een psycholoog doet er ‘ik weet niet hoe lang’ over om daar te komen en om dat te doen met vallen en opstaan. Vervolgens zijn wij zo georganiseerd, dat hebben we nu al hier, waar ik leiding probeer te geven heb ik dat wel afgeschaft. Dan staan er 2 collega’s die een jaartaak krijgen en 2 uur krijgen om sociale vaardigheidstraining te doen en vervolgens gaat er een briefje rond om ‘jongens, hebben we nog leerlingen voor de sociale vaardigheidstraining’. Nou… ik heb er nog wel een, ja, ik heb er ook nog wel een die niet zo lekker in z’n vel zit. Maar er is natuurlijk geen enkel onderzoek of gesprek geweest of die leerling daar wel baat voor heeft of aan de doelstelling / criteria voldoet. Het is volgens mij een beetje kruidenieren wat wij dan doen natuurlijk.

Het is natuurlijk al te gek dat je een briefje rondstuurt met de vraag ‘hebben we ze nog’. Het zou toch andersom moeten zijn, dat je zegt ‘luister, we hebben zoveel leerlingen en we hebben die besproken’.

**Meer, ‘het aanbod schept de vraag’.**

Ja, die collega’s willen wel. Die sturen een briefje rond. Ik heb nu die opleiding gehad… en anders moet ik weer voor de klas. Dus geef me maar een paar leerlingen.

Dan denk ik ‘het zou toch veel beter zijn dat je met een psycholoog, of voor mijn part een vrijgevestigde, waar die markt toch heel in beweging is. Dat je gewoon met een vrijgevestigde psycholoog, waarvan er dan minimaal één NIP-geregistreerd is (??) en je zegt ‘jongens, we hebben nu 7 of 8 leerlingen, komen jullie eens deze kant op’. Het wordt dan betaald via de verzekering, want het zit meestal in het basispakket bij de meesten. Ja, regel het eens lekker via de verzekering, want dat is klaar. Dat zou toch veel beter zijn. Datzelfde geldt voor docenten die bewegingsonderwijs geven, die zien natuurlijk dat er leerlingen last hebben met bewegen, noem het hele verhaal maar op. Dan zou er toch een ‘remedial teaching’ op bewegingsonderwijs moeten zijn. Hier zit een hele fysiotherapeuten praktijk. Laat die gasten komen tegen het verhaal en laten wij nu niet lopen tobben met ons kruideniersgedrag.

**Ik hoor een beetje ‘nou, het signaleren dat doen en kunnen we wel’, maar vervolgens..**

Vervolgens gaan we zelf lopen tobben en als ze dan eenmaal in het circuit gaan, dan weten wij het niet meer.

**Bij M@ZL is wel afgesproken dat er altijd een terugkoppeling is van de jeugdarts aan school.**

Ja, maar de jeugdarts. Maar dan. Met alle respect. Daar komen ze natuurlijk ook maar voor 1 gesprekje, misschien 2. Het is bijna een vervangend leerplicht gesprek, als ik dat heel flauw zeg. Je gaat natuurlijk alleen maar zeggen ‘hee, je moet naar school, want je bent wel in staat om naar school te gaan, dus ga maar’. Nu zeg ik het heel zwart-wit en doe ik iedereen tekort, maar dat heeft natuurlijk niets te maken met een artsenverhaal. Dat is gewoon signaleren en een stok achter de deur van ‘je moet terug’. In mijn beleving heeft dat niets te maken met het arts zijn.

**Ja?**

Het is meer van ‘je moet naar de jeugdarts’ en die gaat zeggen van ‘je bent gezond genoeg en je moet naar school’. Het is eigenlijk een tussenstap, je kan ze net zo goed gelijk naar de leerplichtambtenaar sturen. Waarom zit de arts ertussen? Die doet er niets mee, met alle respect.

**Waarom zit die arts er tussen?**

Die zit er natuurlijk tussen om te kijken of het gerechtvaardigd is en of er een medisch stuk is, waardoor je kan zeggen ‘oké, we snappen en weten het’. Maar als die mevrouw een verklaring heeft van moeder dat dochterlief zoveel keer bij de controlerend geneesheer of bij een psycholoog of weet ik wat moet zijn, waardoor ze veel afwezig is, ja, dan kan je al die papieren laten zien en vervolgens zeg je dan ‘oké, het is gerechtvaardigd’. Dan denk ik, ja, dat kan ik hiervandaan ook wel beoordelen. Daar heb je geen jeugdarts voor nodig.

**Ja, ik denk..**

U wilde een mening, hier is die.

**Nou, ik denk juist dat het heel belangrijk is om dit te horen.**

Die schakel, ik zie de meerwaarde van die schakel niet.

**Hoe zou het anders kunnen dan?**

Ik denk echt dat je als het gaat over.. Ehm.. ik wil er nog één stap tussenzetten en dat is ZAT, het zorgadviesteam. Daar komt de hele wereld aangevlogen. Een leerling wordt besproken. De wijkagent zegt ‘ja, dat ventje ken ik wel’, maatschappelijk werk zegt ‘ja, dat gezin kennen wij’. De school zegt ‘ja, wij kennen hem ook want we hebben hem aangedragen’. Nou, zo zijn er nog wel een aantal die zeggen dat ze hem kennen, we zitten daar met z’n allen gezellig. Maar dan, wie is de probleemeigenaar. Wie zorgt er nu dat er actie ondernomen wordt en hoe wordt het gecontinueerd. Wie zegt nu wat we doen, wie is de koppeling. Nou verbetert dat de laatste jaren wel, maar ik vind het nog echt amateuristisch gedoe.

Dat komt natuurlijk omdat al die belangen er zijn. Dus ik denk echt dat wij, als ik moet reageren op de vraag ‘hoe zou het moeten’, dat je zegt ‘de opzet van het CJG, dat vind ik een goede’. Alleen moeten die niet ergens achter in een nieuwbouwwijk zitten waar niemand ze gevonden krijgt, want die moeten natuurlijk op een plek zitten waar je ook makkelijk naartoe gaat, waar het laagdrempelig is. Het zou toch zo moeten zijn, dat je vanuit de opzet van het CJG een leerling gemeld wordt en van daaruit gezegd wordt ‘hup, zo gaan we dat met dat ventje of meisje aanpakken’. En die en die spelers betrekken we daarbij. Maar het kan toch niet zo zijn dat we daar met zijn allen onvoorbereid, met alle respect, want iedereen vliegt dat ZAT in en we gaan op dat moment kijken wat we hebben. Het zou veel meer handelingsgericht moeten zijn, ‘jongens, hup, dit hebben we, deze gegevens, we hebben het goed voorbereid en dit is ons voorstel’. Maar het is allemaal andersom. We gaan daar kijken en iedereen zegt ‘ja, we kennen dat ventje wel’. Dat weet ik, daar zitten we hiervoor! Ik bedoel, dan sturen we al van tevoren de te bespreken leerlingen door, maar we gaan pas terplekke bedenken wat er moet gebeuren. Je schaamt je toch en durft in de buitenwereld toch niet te vertellen dat het zo werkt, hoeveel geld dat kost en hoe ineffectief het is, daar wordt je toch helemaal verdrietig van. Ik word daar verdrietig van en denk dan ‘schiet toch op’.

**Ik hoor iets van ‘beter voorbereid, handelingsgericht’ en iemand moet het coördineren.**

Ja, en de expertise moet gewoon in huis zijn, van ‘jongens, zo gaan we dit handelen’. En tegen de wijkagent ‘jij gaat met de ouders afspreken dat dat ventje niet meer op straat komt na achten’ en ‘jij zorgt dat hij dit doet’ en ‘jij zorgt dat hij dat doet’. Kortsluiten dat stuk. Dan hebben we ze natuurlijk zo in de tang. Maar op het moment dat dat natuurlijk is van ‘voordat dat dan helemaal doorgehobbeld is en we gaan vaak met elkaar praten, maar dan worden er niet eens afspraken gemaakt’. Dan zeggen we ‘ja, het is zo’ en de volgende keer staat het ventje weer op de lijst.

**Helder.**

Ik vind het een beetje tobben hoor.

**Toch weer terug naar ziekteverzuim. Deze school had M@ZL compleet gekozen (criteria, jeugdarts houdt verzuimgesprek en koppelt terug). Er is ook M@ZL smal ontwikkeld (school voert zelf verzuimgesprek en bepaalt zelf of er doorgezet moet worden, zonder strikte criteria).**

**Als u het nu voor het kiezen had, welke variant zou u dan nu kiezen en waarom?**

Ik blijf bij het complete pakket, omdat ik ervan overtuigd ben dat we met elkaar naar de eindstreep moeten, al is die nog ver weg. We hebben nog een heel eind met elkaar te gaan. Wij hebben het ook niet goed op orde intern. De dwang om, de druk van het melden en het ondernemen van actie is voor mij een beetje geruststellend dat die gasten in ieder geval in de gaten gehouden worden. En dat er goed naar gekeken wordt. Ik heb mijn zorg op deze school nog niet zo op orde, zodat ik het over kan laten, met alle respect, aan collega’s. Dat ook die verzuimgesprekken zouden gaan gebeuren enzovoorts.

Het zou natuurlijk heel logisch zijn vind ik, dat zo’n school ook afgerekend wordt op verzuim van leerlingen. Als een leerling verzuimt, nou, goed daar kan een school niets aan doen. Nu zeggen we ‘ja, die leerling loopt op straat en zo lang..’. De accountant zegt ook nog ‘hij is zo lang afwezig geweest, dus je krijgt niet eens bekostigd’. Dat is een absurde regeling natuurlijk. Als je kan aantonen dat je alles gedaan hebt, zoals het voeren van verzuimgesprekken..

Af en toe stap ik in de auto en als er eentje niet is, dan haal ik hem. Denk ik, is dat ‘rotjong’ er nu weer niet, dan stap ik in de auto en ga ik hem halen. Heb ik al een paar keer gedaan. Staan de ouders daar. Dan kijken mijn collega’s me aan, van ‘goh, ben jij gek’. Dan heb ik ze wel op school! Klaar, hup in de auto en halen. Het zal toch niet waar zijn dat hij er weer niet is. ‘Ik heb gisteren afgesproken dat hij er zou zijn’.

**En dan komt hij mee?**

Ja, natuurlijk. Dat zal waar zijn, dat hij niet meekomt.

Maar als je kan aantonen dat je er heel veel begeleiding opzet om die leerling te helpen, dan is het van de zotte dat je gekort wordt op je financiën. Want juist die leerlingen vragen alles. Op het moment dat het zo’n verhaal is, dan geeft het veel makkelijker over. Het financiële stuk, dat is natuurlijk een absurde regeling, helemaal debiel. Terwijl als je zegt ‘luister, wij hebben de verantwoording voor die leerling genomen, het wordt bekostigd, dus je pleegt ook de inzet van de leerlingen die afwezig zijn en je zorgt dat ze er weer bij blijven’. Dan hou je ze gewoon ingeschreven en dan is er niets aan de hand. Maar als een leerling er een tijd niet is om welke reden dan ook en je hebt er alles aan gedaan en vervolgens zeggen ze ‘ja, je mist de bekostiging’, denk ik ‘schiet toch op man’.

**Dus de complete versie helpt u eigenlijk als school om de zorgstructuur ‘dwingend erin te brengen’, want op dit moment is de school er nog niet klaar voor om zelf verzuimgesprekken te voeren. Als dat moment er wel komt, wie zou dat dan moeten doen? Hoe ziet u dat voor zich?**

Nou, goed, ik heb hier ingevoerd dat de mentor elke veertien dagen een gesprek met de coördinator voert over de voortgang van zijn klas. Dan bespreek je de cijfers.

**De zorgcoördinator?**

Nee, de algemeen coördinator. HM en JvdH, daarvan heb ik gezegd van ‘luister, we doen niet meer onderbouw of bovenbouw, we mixen het door elkaar’. Want het verschil tussen onderbouw en bovenbouw is erg groot. De overgang van de basisschool naar het voortgezet onderwijs is gemakkelijker dan die van de 2^e^ naar 3^e^ klas binnen de eigen school, zeg ik wel eens. Dus ik heb dat gemixt. Vervolgens zeg ik ‘ik wil dat die mentor elke 14 dagen zijn vorderingen van de klas bespreekt’. Daarin bespreekt hij het gedrag, de voortgang, presentie, absentie en contact met de ouders. Van daaruit worden afspraken gemaakt zoals ‘jij doet dit, ik doe dat, ik meld het bij de ambtenaar leerplicht enzovoorts’. De meeste coördinatoren zetten dat in werking. En dan de volgende keer komen ze na 14 dagen weer bij elkaar en wordt er besproken wat er gedaan is, zoals het bijvoorbeeld een gesprek met de ouders. Dat wordt allemaal in het systeem gezet, dat gebeurt echt strak. En ik krijg cc een verslag van dat gesprek wat heeft plaatsgevonden tussen de mentor en coördinator. Daar zijn we net mee begonnen dit schooljaar. De collega’s zijn daar nu aan gewend. Eerst was dat ‘wat een gedoe, we moeten veel meer vergaderen over de leerlingen enzovoorts’. Ik zeg ‘ja, om te constateren dat er weer niets gebeurd is’, weet je wat dat kost als er 30 man zitten van ‘ja, Pietje is wel vervelend, ja dat was hij 14 dagen geleden ook, maar wat doen we eraan’. Daarvan heb ik gezegd ‘met dat gedoe stoppen we’. Op de vergadering moet een voorstel komen….

**En de vergadering is dan..**

Als we 3x per jaar over die leerlingen praten, heb ik gezegd, hupsakee, vanuit de mentor én coördinator: ‘dit is ons voorstel’.

**Dat zijn de vergaderingen naar aanleiding van het rapport?**

We hebben leerling-bespreking en we hebben een cijferbespreking. De leerling-bespreking zegt iets over het gedrag en over de presentie en welke maatregelen/ wat er is afgesproken met jan en alleman. En de cijfers zijn echt van ‘op welk niveau functioneert die’, kan die over of niet.

**Ik hoorde twee namen, daar zit de zorgcoördinator dan niet bij. Waar zit die dan?**

We hebben gezegd dat het anoniem is.

**Ja hoor.**

Kijk, die algemeen coördinatoren die moeten natuurlijk signaleren wat er in de klas mis is en die moeten met de zorgmensen, in dit geval R., zeggen van nou luister, ik heb ‘die en die en die’, dat gaat niet goed in die klas. Daar moet extra aandacht aan besteed worden in de zin van ‘dit zijn de gegevens, wat is jouw advies om met die leerlingen verder te kunnen’. Want wij hebben dat wel geconstateerd, met H. en de mentoren doen niet anders als constateren / bellen en praten enzovoorts, maar het als buiten de school moet, in de 2^e^ lijn, dan moet er natuurlijk iemand zijn die weet waar het over gaat. Dan zijn die mentoren en die algemeen coördinatoren uitgekruidenierd. Dan moet er wat anders gebeuren. Dat zou natuurlijk dan in een overleg met die algemeen coördinatoren, die de boodschappen van de mentor doorkrijgen, naar de zorgcoördinator gaan. Die zegt nou, die melden we bij M@ZL of ‘laat dat nog maar even liggen, want daarvan weten we dat het even niet anders is, maar die krijgen we wel weer op de rit, met die ouders hebben we net goede afspraken gemaakt, laten we nog maar even wachten. Of die komt met die gegevens naar het ZAT, die dan het voorbereidende werk al gedaan heeft en zeggen ‘jongens, dit is de casus, hier willen we wat aan gedaan hebben’, in het voorbeeld wat ik net aangaf. Waarvan ik dus denk dat het anders kan. Dus je hebt daar echt die gelaagdheid in.

**Ik zou jullie mee willen geven, dat gaat eigenlijk buiten het interview om, om de aanmeldingen voor M@ZL op een lager niveau te doen (ipv de zorgcoördinator). Er kan verbetering plaatsvinden, ook met de terugkoppeling. De aanmelding zou misschien effectiever kunnen.**

Ik snap wel wat u zegt, maar R. zit ook niet op de goede plek. R. is niet de goede persoon die dat moet doen.

**Daar zijn we het over eens.**

Dus dat moet even zijn tijd hebben. Ik denk dat je daar iemand neer moet zetten die weet hoe de wereld in elkaar zit. Er moet iemand zitten die tegen die ouders kan zeggen ‘nu is het afgelopen’. En op het moment dat je die confrontatie niet aangaat. Of iemand die proactief is. Die moet natuurlijk tegen de jeugdarts of wie dan ook zeggen van ‘ik heb dit aangeleverd, hoe staat het ermee’, als dat zo zou zijn. Maar op die plek moet echt iemand anders komen.

**Ik geef je mee dat een heel belangrijke succesfactor voor M@ZL is, dat je het zo laag mogelijk in de organisatie laat. Er moet eigenlijk heel snel een reactie zijn van een mentor op dat niveau met de jeugdarts. Ik wil jullie aanbevelen om hier eens over na te denken, omdat er nog een verbetering kan zijn.**

Ik hoor goed wat u zegt, maar ik heb daar een hele duidelijke mening over. Ik vind dat de individuele mentor daar afzijdig van moet blijven. Die snappen dat niet, die moeten bij de core-business blijven, die moeten lesgeven.

**Maar op dat iets hogere niveau dan.**

De coördinatoren doen dat nu, die relatie zit daar gewoon niet goed. Maar dat is in maart opgelost.

**Dat gezegd hebbende gaan we terug naar het interview.**

**Mag ik met u naar de jeugdgezondheidszorg, wat weet u daarvan?**

Wat weet ik daarvan? Ik bedoel, ik weet niet op detail wie wat doet.

**Het is geen proefwerk hoor, maar ik ben benieuwd. Wat komt er in u op? De rol van de JGZ, verantwoordelijkheden, positie in het veld, wat dan ook.. ?**

Kijk, de JGZ als fenomeen ken ik niet goed. Ik ken alle pionnetjes. Ehm..

Als het gaat over de maatschappelijk werk of de schoolarts of … enzovoorts, die pionnetjes ken ik wel, maar ik heb geen samenhang ontdekt in JGZ. Maar dat heb ik zojuist al aangegeven.

**Dat is ook niet erg. De GGD wordt door de gemeente betaald om de zorg uit te voeren en daarbij hebben we een basistakenpakket dat we moeten uitvoeren. Dat is redelijk vastgetimmerd de laatste 10 jaar. Voorheen deed elke GGD naar eigen inzicht JGZ bedrijven, maar toen hebben we gezegd dat elk kind recht heeft op dezelfde JGZ, basaal. Dat heeft zijn voordelen en nadelen. De JGZ beseft heel goed dat we daarvoor eigenlijk heel intensief moeten samenwerken met de signaleerders in het veld, zoals de scholen. Nu zegt de GGD ‘ziekteverzuim is eigenlijk een heel belangrijk signaal’. De ziekteverzuimbegeleiding zit niet in het basistakenpakket, hoe kijkt u daar tegenaan? Daardoor moet er nu ook extra betaald worden door scholen. Ik zou het heel graag met u over die financiën hebben.**

Geld maakt me niets uit. Er is geld zat in Nederland. Waar hebben we het over, we zijn ongelofelijk rijk. We krijgen elkaar niet gevonden. Wie dat nu betaalt. Ik denk dat we het er eerst eens over eens moeten zijn over ‘wat willen we nu’. Hoe gaan we het doen?

Kijk, er zijn allerlei initiatieven. Ik heb hier de jeugdtafel gehad van CJG, we werken samen met de Universiteit Utrecht, waar we ‘gezond maakt slim’ meedoen. Die bevragen de leerlingen 2 keer op een jaar naar hun 7 leefstijlen (gamen, roken, drinken, sex, bewegen etc), daarvan hebben wij gezegd ‘oké, die cijfers worden weggezet tegen de landelijke cijfers van de GGD en waar wijken wij positief of negatief op af. Daar moet dan natuurlijk actie op ondernomen worden. Ik bedoel, wij kregen door van de laatste meting dat die leerlingen verhoudingsgewijs bij ons veel drinken en wij kregen door dat de sociale context van die leerlingen negatief afwijkt van de landelijke cijfers. Dan weet je al een paar dingen en met die cijfers moet je natuurlijk ook wat doen. Wij doen met de GGD het verhaal van het BMI, waarbij we de leerlingen allemaal meten en wegen enzovoorts.

Waar ik naartoe wil, is dat wij samen zeggen: naast een rapport, moet de leerling ook een ‘gezondheidsrapport’ mee krijgen vind ik, eens in een jaar. Daar moet het BMI in staan, waarin staat hoe vaak ze te laat zijn en hoe vaak ze… dit…, daar moet ook de coopertest en een conditiecijfer in staan. Nou, daar heb je al een aantal gegevens waar die ouders wat mee kunnen. We geven dat nu mee en vervolgens krijg ik discussies met ouders van ‘mijn kind is nou een keer te dik, waarom ga je dat nog eens op papier zetten’. Lastig vraagstuk. Maar wij zijn natuurlijk wel degenen die signaleren. Wij moeten dat ook aangeven vind ik en we moeten dat gesprek ook aangaan met ouders. Maar we moeten dit natuurlijk ook met de instanties kunnen bespreken. Maar nu zeggen we ‘ja, oké, jullie hebben die gegevens, maar daar moeten we dan weer een of ander subsidieproject voor aanboren, want niemand heeft daar nu tijd voor’.

**De Universiteit Utrecht heeft dus wel die monitoring en houdt het dan verder op? Krijg je vooral de cijfers terug?**

We krijgen de cijfers terug, maar we spreken ook met hun de acties door en zij doen ons ook aanbevelingen. Als wij in die voeding of wat dan ook afwijken. Dan vind ik dat we met elkaar zo ver moeten gaan dat je als school zegt ‘bijvoorbeeld op 1 januari zijn we rookvrij, klaar’. We zitten nog aan contracten van de cateraar vast, maar al die rommel moet eruit en we moeten een gezonde kantine hebben. Uiteindelijk moet dat rapport komen en dat moeten we met elkaar kunnen bespreken. Even goed, dat we hopelijk in een paar jaar tijd kunnen bereiken dat het ZAT ook actiegericht is. Klaar. Dat hele stuk heb ik wel voor ogen. Daar zullen wij elkaar natuurlijk, buiten onze eigen eilandjes, bereid moeten zijn om allemaal te investeren. En te zeggen ‘waar vinden we elkaar’. Nu is het zo, ‘dat behoort niet tot ons takenpakket’. Dan zeg ik ‘ja, rot op, dan veranderen we nooit’. Wat moet ik daar nu mee, met zo’n opmerking. Ik investeer, wat ik heel logisch vind, in het meten van die BMI, dat hoort in de les (gezondheidsonderwijs). Ik vind dat je een gezonde kantine moet hebben, maar ik kan ook zeggen ‘ik verkoop kroketten’, krijg ik misschien nog wel meer leerlingen door ook. Want dan hebben ze alles, dat is leuk. ‘We maken een leuke disco, geven grote schoolfeesten’. Ik heb ook gezegd dat er wat mij betreft geen drank meer wordt geschonken op schoolfeesten, klaar. Nou, het hele spul op zijn kop hier. Ik wil het gewoon.

**U bent heel gedreven om die gezondheid goed weg te zetten, hoe komt dat?**

Omdat ik me ongelofelijk zorgen maak over al die eilandjes. Ik word er echt gek van. Dan denk ik, nou dan doe ik het zelf maar. Dan zie ik wel wie er wel of niet mee doet. Ik heb altijd initiatieven genomen naar het CJG. Ik snap dat allemaal wel, die zitten natuurlijk ook te zoeken en die hebben moeite zat om ‘alle kikkers in de kruiwagen te houden’. Dat snap ik allemaal wel. En iedereen voelt zich bedreigd, vraagt zich af hoe het moet. Als ik kijk wat er bij Driespan gebeurt, de ambulant begeleiders, alles staat op z’n kop, iedereen is onrustig. Dan kan ik natuurlijk nu samenwerking gaan zoeken met de Driespan, dat doe ik ook wel, dat ik zeg ‘hupsakee, jongens, geef mijn collega’s eens training en begeleidt ze’. Ik zorg best dat ik een paar ‘goede’ van de Driespan bij mij in dienst heb volgend jaar, want die snappen het wel.

**Het geeft ook kansen, hè. Chaos geeft kansen, zeggen ze altijd.**

Precies. Met die mensen die die expertise hebben, die haal ik wel naar binnen en die moeten dat hier gewoon beter gaan opzetten en gewoon ook eisen stellen aan de partijen. Ik denk, wij zijn de vindplaats, wij vertegenwoordigen tot op zekere hoogte die leerlingen, wij zijn mede bepalend wat er gebeurt. Ik wil niet afhankelijk zijn van allerlei bureaus die hun eigen taken moeten halen en al die dingen. Ik snap het, dat is allemaal uit de historie zo gegroeid, maar we gaan het echt anders doen.

**Maar die ontwikkeling van zorg in en om de school, die juicht u ook toe dus?**

Ja, die juich ik ook toe. Maar dan moet je dat ook met elkaar doen. En niet kunnen zeggen van ‘ja, dat hoort niet tot mijn taken’. Daar word ik echt, als ik zo’n antwoord krijg, zeg ik ‘we stoppen met praten’. Ik heb hier al zoveel mensen aan tafel gehad die zeggen ‘dat kunnen we niet of dat past niet in mijn pakket’. Daar ga ik niet eens mee samenwerken. Daar stop ik gewoon geen energie meer in. Ik heb nu een aantal spelers waarvan ik denk, nou ‘die krijgen we best bij elkaar’. Ik heb een psychologenpraktijk die daarmee in wil stappen. Ik heb een fysiotherapeutenpraktijk die daarmee wil doen. Ik heb een paar mensen bij de Driesprong die eruit moeten, ‘kom dan maar bij mij’. Dan gaan we gewoon eens een expertise weg zetten. Dan kan iedereen hoog of laag springen, maar ik heb de cijfers, ik heb met een aantal collega’s een actiecluster gemaakt ‘de gezonde school’. Dat zijn 5 collega’s die naast hun normale lesgeven helemaal bezig zijn om dat project weg te zetten. Geen flauwekul, er moet binnen een half jaar na nu, moet het er staan.

**Waarom vindt u dat zo belangrijk, een gezonde school?**

Ja, omdat ik vind dat leerlingen vandaag de dag met zoveel rotzooi geconfronteerd worden dat ze hun eigen keuze niet meer kunnen maken. Dat moeten wij voor ze doen. Wij moeten die leerlingen beschermen tegen al die rotzooi die aangeboden wordt. Ik bedoel, we hoeven niet ‘rooms te zijn als de paus’. Van mij mogen ze best chips eten of een keer een frietje halen, wat maakt het allemaal uit. Maar daarnaast moeten ze natuurlijk ook andere dingen eten, klaar. Ik bedoel, we hebben hier een centrum van duurzaamheid gemaakt binnen de school. Ik vind dat die leerlingen die van Prinsentuin afkomen, in hun DNA en genen moeten hebben, dat je netjes met de natuur en jezelf omgaat. Ik doe het ook niet altijd, maar ik probeer wel een beetje het evenwicht te houden. Leerlingen moeten zich daar gewoon bewust van worden. Daar moet je eerst een infrastructuur voor wegzetten en je moet het er met je team eerst over hebben.

**Maar er zijn directeuren die zeggen, nou, gezondheid. Ik ben hier om kinderen iets bij te brengen en iets te leren. Gezondheid, daar bemoei ik me niet mee.**

Dat is de helft van het werk.

**Heel mooi, ik hoor dat graag natuurlijk.**

**Met welke argumenten kan ik een directeur die niet zo denkt overhalen? Om gezondheid wel boven op de agenda te zetten?**

Tja..

Die scholen zijn teveel door dat geld gestuurd. Die zitten allemaal te kijken van ‘wat past wel en niet in mijn begroting’. Die vinden dat er allemaal bij horen. Vervolgens doe je een project – we hebben hier het project van de donordag gedaan, met Lange Frans hier ’s morgens binnen, de kinderen op de tafel en rappen. Een aantal mensen die ook een donorlong, donorhart of donornier hadden, die kwamen hier vertellen. Die jongeren hebben meer geleerd in die dag, dan in 12 lessen biologie. Dat weet ik zeker. Dat mag ik zo niet zeggen tegen die collega, maar ik weet zeker dat ze toen veel geleerd hebben. ‘Ze zaten met een knijper op hun neus, waarbij iemand vertelde dat hij zich zo voelde toen hij nog geen nieuwe long had’. Nou, dat vergeten ze nooit meer. Daar kan je 3 theorie lessen aan geven en dan denken ze ‘het zal wel’ als ze de functie van de long gaan uitleggen.

**Helemaal misschien bij deze kinderen?**

Ja, die hebben dan ‘Lange Frans, weet je nog, toen was die er ook’.

Dan denk ik, als je het voelt dat het er bij is, dan gaan die collega’s in het verzet. Die gaan bij mij ook in het verzet, omdat ik internationalisering op de agenda zet en dat ze dan weer een week een klas of 5 leerlingen ‘kwijt zijn’. Zo zijn we vorige week naar Letland geweest. Dan zeggen ze ‘die leerling heeft een week geen les gehad’, maar ik weet zeker dat ze dan meer geleerd hebben als in de les. Dat weet ik zeker. Ze hebben daar Engels moeten spreken, kaartjes moeten kopen, eten moeten bestellen, in een hotel geslapen, gevlogen wat ze nog nooit gedaan hadden. Dan zeg ik ‘ze hebben meer geleerd dan bij jou’. Dus die collega’s zitten ook in het verzet voor al die veranderingen die ik doorvoer. Maar ik laat me niet door geld drijven, want dan denk ik, nou dan gaan we maar in het ‘negatieve’, het is niet anders.

**Wat zijn uw drijfveren dan?**

Die jongens beschermen en bewust maken hoe ze in het leven staan. Dat ze netjes met een meid moeten omgaan en respect voor hun naasten hebben. Ik vind dat de basis van, en dan gaat het leren volgens mij ook een stuk makkelijker. Als je geen energie hoeft te stoppen in pesten, dan heb je toch meer energie om te leren, denk ik. En als je met elkaar dingen doet, dan wordt het toch veel leuker om je heen! Zou ik denken.

We zijn nu bezig, en ik geloof er heilig in dat dat een ‘tool’ is, om te kijken dat je collega’s kan overtuigen. Nu gaan we op excursie hè, weet ik waar naartoe, en we doen een donordag, een duurzaamheiddag en we doen ‘dit en dat’, dan zeggen die collega’s: ‘al mijn lessen gaan eraan, wat is dat voor flauwekul’. ‘Jij altijd met die gekkigheid, het is onrustig in de school’. Maar je moet natuurlijk geen project doen om een ‘project te doen’, dat moet natuurlijk een doel hebben.

Dus als je zegt ‘luister, we gaan een donordag doen’, dan betekent dat dat je zegt ‘wat gaan we doen, hoe bieden we het aan’. Maar eerst moet het doel bepaald worden: ‘waarom doen we het’. Als je dat dan allemaal invult, rollen er vanzelf eindtermen of kerndoelen uit, waarin staat dat ze die dingen… dat is een tool, dat gaan we samen met de SLO ontwikkelen. Ik heb ze gelukkig zo ver dat ze daar subsidie voor uitgegeven hebben.

**SLO?**

Stichting Leer Ontwikkeling. Die zijn verantwoordelijk voor de eindtermen. Dus die omschrijven de leerdoelen van het VMBO, HAVO, atheneum. Ze zeggen ‘dat moet in het curriculum’.

Als je dan al die projecten invoert en je maakt een jaarplanning en zegt ‘in maart doen we dit, in januari dit, enzovoort’. Vervolgens laat je dan goed invullen wat je gaat doen en wat de doelen zijn, dan komen daar aan het eind kerndoelen of eindtermen uitgerold. Als je dán tegen een collega zegt, ‘luister, we hebben donordag gedaan, we hebben dit onderwerp en dat dekt die hele eindterm, dat hoef je alleen nog maar te toetsen, want we hebben dat behandeld op die dag’. Maar nu kan het goed zijn dat wij een donordag organiseren… en de leerling heeft het dan of al gehad, of ze krijgen het 3 maanden later weer helemaal. Want ja ‘het boek zegt, we zijn nu bij hoofdstuk 8, dus dat moeten we behandelen’.

**Je moet het integreren in je onderwijsprogramma.**

Nou, als je dat naar collega’s kan aantonen…

Maar dat is met al die zaken. Als je natuurlijk kan aantonen dat je, omdat je iets nastreeft, ook leerdoelen nastreeft, maar niet ervaart dat het erbij ‘moet’.. het moet natuurlijk geïntegreerd worden. Dan moet het eigenlijk eerst een ‘klerezooi’ in de school worden, dat kan niet anders. Eerst moet iedereen helemaal ‘van het padje’ en iedereen moet helemaal ontdaan zijn van alles wat er gebeurt.

**Chaos?**

Eerst moet er chaos komen en van daaruit komen we ergens. Dan zeggen ze ‘ja, we willen eigenlijk al die leuke dingen wel behalen, maar er moet meer structuur in komen’. Nou, als ze dat gaan vragen, is het zo geregeld.

**Dan doet u dat.**

Nee, ik niet. Dan gaan ze het zelf doen.

**Dan komt dat wel. Ook positieve ervaringen opdoen met dit soort zaken is belangrijk natuurlijk. Hoe ervaren de kinderen dat?**

Kinderen vinden het natuurlijk helemaal geweldig. Collega’s waren eerst heel erg tegen de internationalisering. We zijn nu in het afgelopen jaar met 1 groep naar Italië, met 2 groepen naar Frankrijk en ik ben zelf met die 5 jongens naar Letland geweest. En dat hebben we aan die collega’s laten presenteren door de leerlingen aan de lerarenvergadering. Ik doe veel aan presentaties, en dan komt er vanzelf wel een vraag van ‘hoe moet het’. Die leerlingen staan er natuurlijk met zoveel enthousiasme! Je hoeft ze ook niet te vertellen van ‘je moet gaan vertellen wat je geleerd hebt’, want dat vertellen ze vanzelf wel. Als je zegt ‘joh, presenteer eens wat je gedaan hebt en wat je allemaal gezien hebt enzovoorts’, dan komt het goed. Een jongen zei gisteren ‘ik heb dat geleerd, terwijl ik er in de klas niets van snapte. Ik zag het daar en nu weet ik hoe het zit’. Dat zei die zelf.

**Je hoeft cultuurshock niet meer uit te leggen daarna.**

Eitje.

**Ik wil nog even terug naar JGZ. We hadden het net over het basistakenpakket. Wij screenen kinderen in klas 2; dat is eigenlijk op een wat lager pitje gekomen met vragenlijsten, nu komt er een verpleegkundige. Hoe kijkt u daar tegenaan?**

Ik weet dat het gebeurt. Ik volg het eerlijk gezegd niet zo. Ik krijg dan te horen ‘de jeugdarts komt’ en dat is ongeveer het enige wat ik ervan mee krijg. Dat is het antwoord.

**Oké. Misschien is er een reden dat u dat niet volgt..**

Het zou natuurlijk zo moeten zijn dat wij de jeugdarts van tevoren al gegevens aan kunnen leveren, want je kan natuurlijk in 10 minuten die leerling zien, met alle respect… ‘ga eens recht staan, hoe zit het met je rug en weet ik veel wat jullie daar allemaal doen’… Ik ben wat zwart-wit. Maar dat ventje van mij (die mijn telefoon ingesproken had), is ook al 3 keer bij de jeugdarts op de basisschool geweest, een pleegkindje, maar die is al 3 keer langs geweest, maar die arts heeft nog niet gezien dat hij platvoeten heeft. Staat niets over in het rapport. Ik bedoel, klaar, dan denk ik ‘ja, ik ga elk half jaar nieuwe steunzolen halen, dat is best, maar er staat niets in het rapport’. Het zou toch goed zijn als jullie van te voren weten ‘zo staat hij erin, enzovoorts en z’n BMI is dit’, dan hebben jullie natuurlijk al een paar gegevens. Dat is makkelijker is als dat hij binnenkomt van ‘oh, hangt ie er zo bij’, dan kan je natuurlijk zeggen dat het toch wel zorgwekkend is. Die dingen die wij niet zien of waar geen actie op ondernomen is. Dus ook dat is een stuk waar wij niet zien, maar dan spreek ik voor mezelf of misschien doe ik u of mijn collega’s tekort, maar ik zie gewoon niet wat ermee gebeurt. Het enige wat ik doe is een kamer beschikbaar stellen en voor de rest niets. Dan denk ik ‘ja, wat schiet ik ermee op’. Die kinderen wel, als er iets zorgwekkends geconstateerd is, zal er wel een verwijzing komen van ‘hee, ga daar eens heen, daar moet naar gekeken worden’. Die hele signalering en dat hele stuk moet natuurlijk op elkaar aansluiten. Het moet natuurlijk zo zijn dat het weten. Dit is de vindplaats, die leerlingen zitten natuurlijk, naja, die zorgwekkende kinderen – we hebben het over 12%, want de rest hobbelt er wel tussen door, die grijze massa, die komen er wel. Maar we hebben het natuurlijk over 12%, een willekeurig getal, maar het zal er niet ver vanaf zitten.

**Het zal niet zo willekeurig zijn vaak..**

Maar ik bedoel, dan zou je natuurlijk zo ver moeten gaan, dat je ook weet of die leerling halfjaarlijks bij de tandarts is enzovoorts. Als je bevestigingen hebt, als we dat samen voor elkaar krijgen, dat je weet dat zo’n kind halfjaarlijks naar de tandarts is, dan hoef je daar natuurlijk niet naar te kijken. Dan weet je dat, dat zou gekoppeld moeten worden.

**Veel meer geïntegreerd.**

Dan weet je van elkaar hoe de vlag erbij hangt. Als ik een te dik kind heb, en als mentor ben je zo geschoold dat je het gesprek met de ouders aankan, van ‘goh, hoe is nu het eetgedrag van uw zoon of dochter, want we zien in het boekje dat hij maar amper ontbijt of we maken ons daar zorgen over’. Nou, als die ouders dan zeggen ‘waar bemoei je je mee’, dan is dat straks een kind wat in het ZAT besproken wordt. Daar kan je natuurlijk de klok op gelijk zetten. Dat moeten we natuurlijk gaan monitoren. Daar moeten we naar toe. Dan hebben we nog wel een weg te gaan, maar zo zou het toch moeten. Lijkt mij.

**Dankjewel.**

**Als ze onderzoek doen naar effectiviteit van M@ZL, wanneer zou u het als schooldirecteur effectief vinden? Wat verstaat u onder de term effectiviteit?**

Het ziekteverzuim terugbrengen, dat moeten we natuurlijk in een percentage kunnen uitdrukken. Daar moeten we streefgetallen voor hebben. We kunnen als school natuurlijk niet afwijken van het landelijk gemiddelde. Dat is één.

Anderzijds, de effectiviteit, als je natuurlijk inderdaad kijkt dat er effecten gesorteerd zijn op signalering en het ‘behandelen’, dan moet je ook kunnen meten. De effectiviteit is nu allemaal op gevoelswaarde. Ik bedoel, als R. het niet goed doet, dan heb ik zoiets van ‘dat rot-M@ZL, elke keer dat gezeur en MP koppelt niet terug of die doet dit niet’. Dan denk ik R., regel het is lekker zelf. Ik ga daar niet achteraan, ik heb wel wat anders te doen. We hebben het zo afgesproken, dan verwacht ik gewoon dat dat gebeurt. Als mensen dan niet meegaan met die schakel, dan ben ik vrij gemakkelijk en zeg ik ‘joh, dan moet je wat anders gaan doen’, ik heb geen zin en tijd om elk individu mee te krijgen. Dat ga ik niet doen. Daar wordt R. ongelukkig van, maar ik kan het niet helpen, want ik geloof er heilig in dat het zo moet. Veel scholen blijven natuurlijk hangen in die functionarissen en die blijven jarenlang hetzelfde doen, want ‘ja, we hebben er toch verplichtingen aan’. Dat is zo, maar wil je stappen maken, dan zul je soms ook een beslissing moeten nemen, want anders kom je er ook niet doorheen. Dus ik moet gewoon meer getallen hebben. Ik ben een mens die op getallen wil sturen. Als ik geen getallen heb, denk ik, tja, mijn gevoelswaarde, of het nu -3 of -10 is, wat heb ik daaraan, het zal wel koud zijn buiten. Het is allemaal gevoel en daar moeten we echt vanaf.

Ik wil bijvoorbeeld ook weten hoeveel leerlingen bij mij drugs gebruiken. Als het er 3 zijn, krijg ik die wel opgespoord. Maar als het er 30 zijn, moet ik toch eens achter mijn oren gaan krabben wat er aan de hand is, dus ik wil weten hoeveel leerlingen er drugs gebruiken. Klaar.

En als er cijfers zijn dat er bij mij geen drugs gebruikt worden, hoef ik mijn kop niet in het zand te steken, want dat zijn natuurlijk ook sociaal wenselijke antwoorden. Dat snap ik ook allemaal.

Maar ik ga natuurlijk geen project opzetten van ‘gezond eten’ als de leerlingen uitwijzen dat ze gezond eten. Dan denk ik ‘ik moet weten waarop ik me inzet en waar ik me zorgen over moet maken’.

**En dat het ook tot verbeteringen leidt.**

**De effectiviteit van M@ZL is afname van ziekteverzuim omvang. Signaleren en doorzetten.**

Ja.

**Nog meer?**

Nou, en het effect van het doorverwijzen of daarna verbetering ingetreden is.

**Voor wie?**

Voor de leerling. Als we niet verder komen als ‘we hebben het gesignaleerd en doorverwezen’.. Je wil natuurlijk ook volgen of zo’n leerling die daar geweest is, of dat tot verbetering heeft geleid.

**Je meet het niet alleen of het verzuim is afgelopen, maar ook of het beter gaat met de leerling.**

Ja.

Nou, een tweedeling hè. Je hebt wel gelijk, ik herken wat je zegt. Het zou natuurlijk op de een of andere manier zo moeten zijn dat de leerlingen die gemeld zijn bij de jeugdarts, dat die gevolgd worden en dat die apart gemonitord worden om te kijken of dat effect heeft. Want het zegt niets als je…Want ook andere acties kunnen invloed hebben, dat je minder ziekteverzuim hebt.

Maar ik wil weten of de actie van het – want ik heb mijn twijfels erbij, maar die heb ik al uitgesproken over het verwijzen naar de jeugdarts – want volgens mij kan je gelijk de boeman erop zetten en die doet hetzelfde als de jeugdarts. Maar dan ben ik heel zwart-wit.

Die leerlingen die daar gemeld zijn, moeten gevolgd worden en kijken of daar een verbetering in zit. Met een gemiddeld cijfer kan ik dan niets, want dan weet ik het nog niet.

**U haalt net aan dat de leerplichtambtenaar het ‘eigenlijk ook wel kan’. Klopt dat? Is dat zo?**

Ja, alles staat of valt bij welke leerplichtambtenaar je komt. Als je P. hebt, die zit er wel bovenop, die regelt het wel. Daar heb je de tussenstap bij de jeugdarts niet voor nodig. Maar als ik die andere meneer heb, denk ik ‘ja, daar kan ik hem 6 keer naar toesturen om nog een keer te praten en nog eens een briefje te sturen’, maar daar gebeurt geen donder. Dat is natuurlijk maar net hoe je met elkaar…

Dan moet je wel een paar goede mensen om je heen hebben. Het is altijd mensenwerk, dus daar ben je afhankelijk van. MP is er wel een die het wat dat betreft goed doet, die is de boeman en laat de kaas niet van haar brood eten, dus die zegt wel waar het op staat. Maar als ik de volgende keer een andere jeugdarts heb die wat milder is, dan zit ik weer met een probleem. Dus volgens mij kan een persoon het ook doen.

**Ik wil ergens naartoe…**

Mag ik u een vraag stellen? De expertise van de jeugdarts op die plek….

**Ja, daar wil ik het eigenlijk over hebben. Er is een verschil tussen een jeugdarts daar zetten of een leerplichtambtenaar.**

Ja, maar wat is dan de expertise van de jeugdarts die je niet zo kunnen overbrengen, met een week nascholing, bij die leerplichtambtenaar?

**Dat wil ik eigenlijk dat u dat zegt. Ik weet het wel.**

Ja.. Ik zie de medische kant met die duur opgeleide mevrouw of meneer, zie ik niet terugkomen in het verslag. Het is een verwijzingsmachine. En dat medische stuk zie ik daar helemaal niet in terug. En daar maak ik me zorgen over. Want dat signaleren hebben wij gedaan en vervolgens gaat die de druk opvoeren dat ze weer naar school moeten en zegt ‘als je nu niet gaat, want ik zie dat je medisch gezond bent…’. Maar in dat gesprek kan die arts ook niet zien of dat kind inderdaad een kleiner hart heeft of weet ik wat..

**Maar zit daar niet een stukje sociaal-medische expertise dan? Dat je die uitspraak op een gegeven moment kan doen?**

Nou, ik denk dat ik duidelijk heb gemaakt dat ik daar mijn twijfels over heb. Ik vind dat zo’n kort tijdsbestek. Ik twijfel niet aan de expertise van die mevrouw of meneer, maar ik twijfel of je in die setting, dat kan gebruiken en dat je er tijd genoeg voor hebt om daar iets zinnigs over te zeggen.

**Het is natuurlijk niet alleen die setting..**

Ze zijn er binnen 20 minuten weg.

Hoe kan je nu in 20 minuten medisch iets zeggen. Over die problematiek. De ouders komen al in het verzet daar naartoe van ‘ik heb vrij moeten nemen, dat gezeik allemaal’, dus de eerste 5 minuten is de weerstand overwinnen, als het geen 10 minuten is.

**1 minuut..**

Nou, daar geloof ik niets van. Dus dan ben je daar al een tijdje mee aan het tobben. Nou, vervolgens gaan ze dan vertellen wat er allemaal aan de hand is. Bijvoorbeeld dat zo’n kind allemaal migraine, hoofdpijn heeft. Dan zegt die mevrouw ‘nou, zou je dan eens niet naar de huisarts gaan als je altijd hoofdpijn hebt’. Die kan met dat medische vraagstuk toch ook niets doen daar in die 20 minuten, daar geloof ik niets van.

Maar ik zeg het altijd nogal scherp. Want ik wil wel weten hoe het zit.

**Nou, ik vind het heel belangrijk om te horen wat er in uw hoofd zit en hoe u er tegenaan kijkt. Het is niet zo interessant hoe ik er nu over denk.**

Ja, ik snap de doelstelling van het gesprek.

**We hebben heel veel besproken.**

**Na dit jaar willen we door met M@ZL of tenminste, dat aanbod ligt er. Wat is er nodig om deze vorm van ziekteverzuimbegeleiding in samenwerking met JGZ goed te implementeren op deze school, zodat het echt blijft?**

Ik heb al gezegd, ik moet intern nog wel een paar dingen / hobbeltjes nemen voordat ik zover ben. Dat zal ik eerst zelf moeten doen. De bewustwording, dat zijn we wel aan het doen met allerlei projecten. Maar de noodzaak en de samenwerking tussen die algemeen coördinatoren, de zorgcoördinator enzovoorts, daar moet ik nog wel een paar hobbeltjes nemen. Ik zeg altijd ‘jongens, die kant moet het op, ontwikkel het zelf’. Ga niet ergens een school zoeken. ‘Hup, gaandeweg en dan schrijven we er wel een keer beleid op. Maar we gaan geen beleid maken en zeggen zo wil ik het uitgevoerd hebben’. Ik geef altijd aan welke kant het ongeveer op moet en dat ze wat moeten ontwikkelen, dan komen ze vanzelf wel met een ding. Als dat dan niet gebeurt, denk ik ‘verkeerde mensen op de verkeerde plaats’. Zo zit ik erin, ik ga niets voorkauwen. Ik kan alleen maar aangeven welke kant ik op wil. Dat heeft zijn tijd nodig.

**Waarom kauwt u niet voor?**

Omdat, als ik het voorkauw, niet de bevlogen mensen op de goede plek heb. Dan gaat er eentje solliciteren die denkt ‘oh, nu hoef ik geen les meer te geven’. Want het enige promotiecriterium in het onderwijs is geen les geven. Nou, heb je ooit zo’n beroepstak gezien?

**Jammer hè?**

Ja, dat is zo. Ze willen allemaal coördinator of directeur worden, want dan hoef je geen les meer te geven. Dan denk ik ‘schiet toch op, dat is toch geen sector’. Er zal toch geen arts zijn die zegt ‘ik ga in landelijke werkgroepen, want dan hoef ik geen patiënten meer te zien’. Dat is toch te gek voor woorden, waar hebben we het over. Ik bedoel, dat is klaar. Je moet op een gegeven moment de ervaring opdoen, dat je zegt ‘nou, zoveel ervaring heb ik in het onderwijs, wil ik graag verbetering in zijn algemeen, dan denk ik dat ik nu zoveel expertise opgebouwd heb dat ik iets kan betekenen’. Maar nu zeggen ze ‘ja, ik wil de schoolkrant wel schrijven, hoeveel uur krijg ik daar voor’.

Ik ga dat niet voorkauwen, mensen moeten dat ontdekken, die moeten geïnspireerd raken door de omgeving, door leerlingen, door de ouders. En zeggen ‘hup’. Als ze dat niet pakken, zitten ze alleen maar formuliertjes in te vullen omdat ik zeg dat het moet. Daar heb ik er zat van.

**Intern dus nog een aantal dingen, extern misschien ook nog wat verbeterpunten?**

Nou, dat is wat ik aangeef.. Ik vind dat ik geen recht van spreken heb als ik mijn eigen spullen niet op orde heb.

**Nou, u mag hier wel suggesties doen.**

Die suggesties heb ik in het gesprek al wel gedaan, denk ik. Ik heb daar wel een beeld bij en of dat helemaal zo uitgewerkt wordt, zoals ik dat voor ogen heb… Je moet wat te wensen hebben. Je moet ergens een doel hebben waarvan je zegt ‘het zou toch mooi zijn als we over een jaar of 4 alle partijen, dat zo’n leerling gewoon een gezondheidsboekje bij zich heeft waarin staat ‘dit ben ik’, dat zou ik helemaal leuk vinden’. Als ik dat voor elkaar heb.. Waarin staat ‘ik loop bij een maatschappelijk werker’, het schaamtegevoel eraf, dat dat gewoon in dat boekje staat. Zodat iedereen kan zeggen ‘god, ventje, daar ben je al mee bezig, wat levert het op’. Dat de maatschappelijk werker die het boekje open doet ook kan zien hoe het zit en hoe het werkt en daar gewoon open over kan praten.

**Krijgt u daar de ouders ook in mee denkt u?**

Ja, daar ben ik van overtuigd. Ouders vinden dit helemaal te gek. Als je hier met ouders over praat, over wat je aan het doen bent en welke visie je daarop hebt, die worden helemaal enthousiast. Die ouderraad zegt alleen ‘hup, gas erop, tempo erin’. Dan zeg ik dat ik tijd moet hebben, maar dan beginnen ze zelf te drukken van ‘hee, we zien nog steeds die automaten staan en waarom worden er geen appels verkocht’. Nog wel leuk om te vertellen, is dat we bij de nieuwe Jumbo die hier geopend wordt, daar hebben we een gesprek met de directeur gehad en die zegt ‘ik zorg dat er 2 jaar lang elke dag fruit voor die kinderen is, voor niets’.

**Nou geweldig.**

Daar word ik helemaal enthousiast van natuurlijk!

Vanuit het duurzaamheidproject willen we gewoon dat het in het DNA komt van die leerlingen. Ik zal jullie nog wat meegeven.

**Dan sluiten we het interview af.**

**Interview 4. School 6 / P_6_ en S_6_  Datum 16-12-2011**

**Aanwezig: Directeur, Zorgcoördinator, Yvonne Vanneste (interviewer), Marlou van de Loo (onderzoeker).**

Algemeen**:**

**Het interview verliep aanvankelijk wat aarzelend. De zorgcoördinator had tevoren duidelijk aangegeven er de voorkeur aan te geven samen met de directeur geïnterviewd te worden. De tweede zorgcoördinator zou er ook bij aanwezig zijn, maar bleek uiteindelijk verhinderd. Vorig schooljaar heeft er, in aanloop van de besluitvorming over het al dan niet meedoen aan het onderzoek, wat inhield het implementeren van M@ZL-smal, eerst een tweetal gesprekken met de zorgcoördinator en vervolgens eenmalig een gesprek met de zorgcoördinator en de directeur samen plaatsgevonden. Ik heb met de zorgcoördinator tussendoor meerdere keren telefonisch contact gehad ter ondersteuning. Dit interview was de tweede keer dat ik de directeur weer sprak. Bij de start van dit interview had de directeur erg de neiging zelf de vragen te stellen, zoals hij gewoon is om in overleggen het voortouw te nemen, verontschuldigde hij. Na uitleg over het doel en de bedoeling liet hij het over aan mij als interviewer om de vragen te stellen. Ook bleek al snel dat hij ‘schrok’ van de investeringen door de zorgcoördinator en bemerkte ik bij mijzelf dat ik bang werd dat hij de voortgang van de implementatie en deelname aan het onderzoek zou gaan stopzetten. Dit was er denk ik de oorzaak van dat ik erg veel vertelde in aanvang. In de loop van het interview nam deze angst af en kon ik het meer loslaten en het interview zijn beloop laten. Het interview had ook een duidelijk oriënterend karakter, oriënterend op het jeugdbeleid en de organisatie en taken van de JGZ. De directeur moest meegenomen worden in de interventie. De sfeer was erg goed, er werd regelmatig veel gelachen.**

Interviewer: dik gedrukt

**Roberto: Z, regulier**

**Directeur: D, regulier**

Hoe vinden jullie dat de implementatie van M@ZL-smal gaat?

**Z: Eigenlijk wel goed, moet ik zeggen. Het doel was dat het moest gaan leven binnen de school en dat gaat langzaam aan wel leven. Positieve gesprekken. Ouders zijn voor 90% positief daarover. In het begin was er weerstand. Het was nieuw natuurlijk. Daarnaast kost het heel erg veel werk, leuk om te doen natuurlijk maar wel heel veel. Het is wel nuttig.**

Ja. wat levert het op?

**Z: Ik denk toch wel minder ziekteverzuim bij sommigen, als ik zo kijk.**

Is dat het enige wat het oplevert?

**Z: Ja, je gaat altijd wel in gesprek met ouders, daar komen altijd wel wat dingetjes uit. Er kunnen ook andere dingen uit komen dan alleen maar medisch.**

Zoals?

**Z: Uhm…..Dat de werkdruk voor die leerling wel heel erg hoog is, problemen thuis komen ook wel naar voren, van alles. Ik pak dan het overzicht van het verzuim en de cijferlijst erbij, meestal is er dan een link als ze zo vaak afwezig zijn. Dus dat klopt dan ook wel.**

Je zei zelf in het begin waren ouders vaak wat huiverig daarvoor, nu heb je..?

**Z: Minder, minder. In het begin deed ik het wel zelf, de afspraken maken, nu doet iemand anders het voor mij, dat vind ik wel handig. Waarom, ik ga toch naar de huisarts, dat is de meest voor de hand liggende hoor, ik heb ook een traject bij het ziekenhuis. Dat is nog wel een lastige nog steeds om daar dan van te zeggen van ja, maar daar kan een schoolarts wel een meerwaarde van hebben. Dat is toch nog wel een lastige.**

Of jij zelf als zorgcoördinator misschien?

**Z: ja**

Zien ze dat? Los van een medisch traject dat je doorloopt, een diagnosebehandeltraject. Kunnen we een diagnose vinden en is daar een behandeling voor nodig? Kun je daarnaast ook mogelijk ervaren dat het gesprek op school zinvol is over het ziekteverzuim van het kind?

**Z: Ja, dat ervaren sommigen wel zo.**

Wat geven ze dan aan?

**Z: Blij dat het gesprek is geweest. Er was er eentje bij die ging dan een beetje een hak in het zand zetten door de telefoon en toen we het gesprek hebben gehad waren ze er toch blij mee.**

Oh, wat fijn.

**Z: Ja, dat er iets uit is gekomen.**

En daar kun je ook wat mee?

**Z: Ja**

Je hebt een aantal gesprekken doorgezet naar de jeugdarts, kun je daar iets over vertellen?

**Z: Ja, dat is gevarieerd. Als ik merk van dat ik toch vind dat er medisch gekeken moet worden, bv slapeloosheid. Toch ook naar het psychische toe, dan adviseer ik toch wel naar de schoolarts te gaan.**

Hoe reageren ze daarop?

**Z: Meestal wel goed. Want ik probeer daar ze in het gesprek in mee te krijgen. Ik probeer er niet meteen mee te beginnen. Ik ga het uitleggen, eerst, dan ga ik naar de reden van ziekteverzuim, neem de resultaten erbij. Zo werkt dat.**

En dan meld je ze aan bij de jeugdarts, hoe gaat dat?

**Z: Goed. Technische kant bedoel je?**

Ja alles, hoe vind je vervolgens dat het gevolgtraject gaat?

**Z: Goed, ik ben er tevreden over, je krijg advies. Alleen is het wel zo van als ze niet op komen dagen bij de GGD heb ik nu het advies gekregen van (naam) bij de volgende ziekmelding naar de leerplichtambtenaar te gaan. Soms is er ook een nieuwe afspraak gemaakt. Dus hoe moet dat? Moet je na 1 keer als ze bij jullie niet zijn komen opdagen moet je dan gelijk naar de leerplichtambtenaar of maak je een tweede afspraak?**

Nou onze ervaring is dat als iemand zomaar niet komt zonder afmelding opnieuw uitnodigen geen zin heeft, dan komt ie weer niet. Dat heeft alleen maar zin als er toch ergens een terugkoppeling door school komt van hé, je bent niet geweest. We zijn zeker bereid om daarna weer opnieuw op te roepen. Als (jeugdarts) adviseert leerplicht in te schakelen, dan kun je dat ook uitleggen als een advies om het in het ZAT te bespreken. Minstens een advies aan school het niet verschijnen te bespreken met ouders.

**Z: Dat staat er dan ook vaak bij.**

Want dat is denk ik de plek van waaruit een actie moet komen.

**Z: Meestal van onze kant even een belletje naar huis van uh …**

En waarom, dan kun je zeggen nou van bel zelf dan, hè, als jeugdgezondheidszorg, maar waar wij heel bang voor zijn is dat als de jeugdarts dan gaat bellen, dat ze zeggen je hoeft helemaal niet te komen want ze belt wel en dan doen we ons verhaal wel over de telefoon. Terwijl eigenlijk vanuit een bedrijfsgeneeskunde heel erg geadviseerd wordt, dat eerste contact moet je echt face to face hebben. Je moet ze zien, je moet ze in de ogen kijken en zeggen we hebben het hier over. En om te voorkomen dat het gesprek over de telefoon wordt afgehandeld, dat is onze ervaring, en als je gaat bellen dat ook steeds minder mensen komen die denken nou doe die moeite niet, ik word wel gebeld. Maar u mag wat zeggen hoor, daarover.

**D: Nou ik zou even wat, even.. ik ben bij het gesprek geweest toen we dat hier hebben aangegaan, ik hoor van binnen de expertisegroepen wel eens van zorg hoor ik even dat het loopt en dat we een paar signalen krijgen dan, en vervolgens zitten we nou weer aan tafel. Even gewoon de facts, zoals dat tegenwoordig heet. Over hoeveel leerlingen praten we eigenlijk met wie we een gesprek hebben gehad dan?**

**Z: …, die zijn er nou uitgekomen. De lijst is nog niet bijgewerkt.**

**D: Maar die worden dan uitgenodigd, bij jou?**

**Z: Ja, en de mentor**

**D: En daar volgt een gesprek mee…**

**Z: Ja, maximaal een halfuur. Dat zeg ik ook in het begin, ik leg het uit, ik zeg dat het geen vermanend gesprek is en dat werkt gewoon heel goed moet ik zeggen.**

**D: Wat me dan opvalt is het alleen vmbo?**

**Z: Ja.**

**D: Want het is niet de bedoeling dat natuurlijk hè het vmbo is.**

**Z: Nee.**

**D: Maar dat is een keus?**

**Z: Nee, (…) komt er niet aan toe op dit moment. Het is zoals ik het begrijp vmbo is het nu op dit moment dan 10%**

**D: De mentor zit erbij hè?**

**Z: Ja, zo nodig, of uh zo mogelijk, dat is wel het streven. Maar mocht de mentor niet kunnen dan gaat het wel gewoon door. Dus er vervallen geen lessen uit of dat niet, maar dat koppel ik terug naar de mentor.**

**D: - zucht- want waar het knelpunt zit, is wel de tijd hoor. Gigantisch veel tijd hoor. .. doet het**

Ongelooflijk ja.

**D: Op een bepaalde drive. Want het knelt dus in de andere zorgtaken, daar gaat het dus ten koste van. Want inderdaad 76, ik wist bij god niet dat het er zoveel waren. Want dat is, reken maar even uit, dat zijn weken hè?**

Nou jullie hebben ervoor gekozen om de criteria aan te houden.

**Z: Ja.**

En dat is natuurlijk een keuze, toch?

**Z: mm.**

En dan kom je inderdaad aan zoveel uren, dat klopt.

**Z: Want ik had ook op een gegeven moment, dit komt er dan nog bij. Dat moet ingepland worden. Elke vrijdag kijk ik van welke komen er bij. Afgelopen vrijdag was ik er niet aan toe gekomen.**

En nodig je ze dan allemaal uit of maak je toch een selectie van die wel die niet**?**

**Z: Dat zijn er maar een aantal, op een hand te tellen, dat ik ze niet uitnodig zegmaar. Iemand met leukemie bijvoorbeeld nodig ik niet uit. Omdat daar al heel veel zorg op is, ja.**

Hoe vaak heb je het dat idee van dat het terecht is en goed is dat je ze uitgenodigd hebt en dat gesprek hebt gehad?

**Z: Ja, eigenlijk altijd wel. Het is dat in gesprek gaan, en soms is het een kwartier maar soms loopt het uit tot een uur, het is wat er uit komt. Soms dan uh is het, gister had ik iemand die komt dan net vier keer uitrollen uit het systeem maar die is ziek geweest, dus 2 ziekmeldingen, is ziek geweest, toch geprobeerd. Dan zegt de moeder ik laat het haar toch proberen, ze gaat dan toch ziek naar huis maar er zijn dan wel twee ziekmeldingen, snap je?**

Ja.

**Z: En dan dat (secretaresse) want daar ben ik heel blij mee dat zij de afspraken maakt, ja zij kijkt technisch, vier keer eruit dus uitnodigen.**

Dat gaat dan niet eerst langs jou, zij zegt dan gewoon komt eruit, en nodigt uit.

**Z: Ja, anders kan ik het net zo goed zelf doen.**

Nee daar hadden we het over gehad, we hebben een paar keer telefonisch contact gehad.

**Z: Ja, daar ben ik wel blij mee geweest. En ik zit nou te denken van is het misschien verstandig om de vierdejaars even niet meer te doen. Want die gaan toch zo op examen, nog een paar maanden, om die dan te laten schieten.**

Ja want het kost heel veel tijd**.**

**Z: Het kost heel veel tijd**

Het verbaast me niks natuurlijk, maar goed, dan zet je toch die jeugdarts, volgens mij komen ze bijna allemaal ook.

**Ja, over het algemeen wel ja.**

Heb je wat aan.. is er een meerwaarde van die jeugdarts?

**Z: Ja, ik vind het fijn wat er gezegd wordt. Kan meedoen aan het volledige lesprogramma, daar hebben we wat aan natuurlijk. Overleg in het ZAT, daar hebben we wat aan. Jawel, en soms staat er ook wat tekst bij en dan is het vervolgens lastig, dus daar heb ik wat aan dat zet ik in het systeem, dat is allemaal prima. En dan moet de mentor het gaan bewaken van hé dat is de afspraak, bij de volgende ziekmelding of de volgende keer in het systeem, of bij de volgende keer moeten we naar de leerplichtambtenaar, om dat te bewaken. Dat krijg ik alleen niet voor elkaar, daar heb ik de mentor voor nodig en dan moet ik maar uitgaan van de mentor dat dat ook gebeurt.**

We hebben eerder gezegd, we hebben een paar keer overleg gehad van eigenlijk zou je dat inderdaad ook op dat mentorniveau moeten krijgen en mogelijk zelfs die verzuimgesprekken, want jij loopt drie keer in de rondte om dat voor elkaar te krijgen en dat is eigenlijk voor 1 iemand veel te veel.

**Z en D: ja**

Terwijl als je het op mentorniveau doet, die heeft maar 2 of 3 van dit soort gesprekken in een jaar, dan krijg je een veel minder zware workload. Maar jij zei zelf al dat durf ik eigenlijk niet om dat op mentorniveau te doen.

**Z: Ja, dat is heel wisselend, dan gebeurt het of niet, of niet goed of goed of heel goed. Ja..**

**D: Mentoren**

**Z: Dat weet ik wel dat dat altijd is met alles maar omdat wij zeggen van dat moet gaan gonzen en niet dat ik allemaal zo goed ben maar ik wil dan, dat is wel één beleid en dan gaat dat wellicht gonzen.**

Je zei in het begin nog toen we overleg hadden van ja ik moest ook even wennen, je doet dat niet zomaar die gesprekken.

**Z: nee…**

Het is toch..

**Z: Qua gesprek leer ik er heel veel van, dat is toch wel verantwoord.**

**D: Wat was het doel van oorsprong? ….Want dit is met een bepaalde opzet gestart hè?....**

Uhm, we doen nu een effectiviteitonderzoek gewoon, dus in die zin is in ieder geval een aantal scholen gevraagd om M@ZL-smal te gaan implementeren om te kijken hoe effectief alles is. Maar u bedoelt misschien het doel van ziekteverzuimbegeleiding?

**D: Ja ik snap het, je wilt beter grip krijgen dat het verzuim teruggedrongen wordt. Dat is een doel denk ik? Maar wat is het doel van jullie als organisatie?**

**- de directeur wil het interview liefst ‘overnemen’…**

Ik wil daar best wel over praten maar ik zou eigenlijk heel graag, mag ik eerst een vraag stellen? Ik zou eigenlijk liever willen weten, welke reden heeft u gehad toen om ja te zeggen hiertegen? Als school, waarom willen jullie dit als school?

**D: Om al het ziekteverzuim beter in beeld te krijgen. Ik schrik nu eerlijk gezegd van de aantallen hoor. En dat is, ik had dat niet verwacht.**

Nee?

**Z: En ook om de aantal ziektemeldingen naar beneden te krijgen, dus om de te snelle ziekmeldingen toch op school te krijgen, dat ze zich niet te snel gaan ziekmelden. Kijk als je ziek bent en je hangt boven de pot dan ben je ziek, klaar, maar een keer een hoofdpijntje dan kun je wel naar school. En als ze dan toch gaan zien van he`we worden op de een of andere manier toch gecontroleerd, weliswaar hopelijk toch op een positieve manier.**

**D: Om beter inzicht te krijgen in het geoorloofde ziekteverzuim, oké, dat is de eerste instantie geweest en als je dat eenmaal doorredeneert dan hoop je daarmee dat de resultaten van leerlingen die veel ziek zijn misschien daardoor iets te verbeteren. Dat is marginaal denk ik. En voor de rest..**

En waarom zouden de resultaten verbeteren**?**

**D: Omdat ze meer aanwezig zijn als ze langdurig vaak ziek zijn, de achterstanden lopen terug, dat inhalen en heel het gedoe dat erbij komt. Dat kan ik me voorstellen. Maar goed ik stel geen vragen. Lastig, meid! --- lachen---**

Lastig hè, ik mag hier de vragen stellen. Het doel hiervan is niet dat wij een discussie hebben, het gaat er meer om dat ik probeer om.. u zegt dan we hebben hiervoor gekozen want we willen meer zicht krijgen op het geoorloofde ziekteverzuim. En dat het ziekteverzuim terugneemt en dus het gemis op de school. En daardoor zouden marginaal ook de prestaties toenemen. En u zegt zelf we hebben nu wel inzicht in hoe omvangrijk het is.

**D: Ja…**

En daar schrikt u van?

**D: Ja. Nu praten we alleen maar over het vmbo gedeelte, ik steek mijn hand er niet voor in het vuur dat de havo en het vwo niet net zo is..uh ernstig is.**

Als we kijken naar het ziekteverzuim als reden, kinderen melden zich ziek of ouders melden hun kinderen ziek. U zei net ik ben dit project gestart om mee te doen om meer grip te krijgen op het geoorloofde ziekteverzuim. Als we kijken naar het ziekteverzuim wat voor redenen kunnen kinderen hebben? We hebben het er net al over gehad. Mag ik daaruit concluderen dat u denkt dat het meeste ziekteverzuim geoorloofd is?

**D: Ja dat ouders dat er aan hangen, ja. Dat wij wel ergens voelen dat daar een x percentage ouders is dat hun kind geoorloofd ziekmelden om allemaal andere redenen. En dat beeld zou ik wel eens willen weten. En vervolgens komen we toch wel even terug op wat ik net wilde vragen: wiens verantwoordelijkheid is dit nou?**

Voor het ziekmelden bedoelt u?

**D: Ja voor het feit dat kinderen zo vaak of formeel ziek gemeld zijn terwijl je je afvraagt of dat wel formeel ziek is.**

Dan stel ik toch de vraag opnieuw: wiens verantwoordelijkheid is dat?

**D: Van de ouders.**

En waar ligt de verantwoordelijkheid van een school? Of jeugdgezondheidszorg? Want de ouders melden ziek en het is hun verantwoordelijkheid, zij maken een keuzen om hun kind ziek te melden.

**Z: Dat wat je toen zei dat is altijd bij me gebleven van: een directeur heeft het recht om te vragen waarom heb je ziek gemeld? De directeur geeft toestemming om het kind geoorloofd thuis te hebben, dus mag hij ook weten waarom.**

Officieel, daarom, u vindt het misschien vervelend dat u de vragen stelt. Het gaat, het is uiteindelijk de directeur die toestemming geeft, en dat zijn we een beetje vergeten, maar de ouders melden een kind ziek dat is ook wel een complicerende factor he, het is niet kind maar de ouders melden hun kind ziek en in het beginsel, iedereen kan zich ziekmelden, geeft een directeur toestemming. De schooldirecteur geeft toestemming om het kind thuis te houden. Zo staat het in de wet. En daar kun je als school aan toevoegen: als het heel vaak of langdurig is, dan hebben we op deze school de regel dat er een gesprek volgt, en eventueel verwijzing naar de jeugdgezondheidszorg of leerplichtambtenaar. Dat is eigenlijk de mogelijkheid die wij gezien hebben om te zeggen oké dan gaan wij daar structureel een interventie opzetten, en aandacht aan besteden. Het blijft dat ouders bepalen of ze hun kind ziek melden, maar als ze te vaak of langdurig hun kind ziekmelden hebben we vanuit zorg een gesprek en zo wordt het ook gecommuniceerd met ouders.

**Z: Ja, absoluut.**

En vanuit de arbeidsgeneeskunde weten we dat maar 5 procent echt geen keuze is. Dan hang je boven de pot, zeg maar. 5 % van de ziekmeldingen is geen keuze. En bij 95 % van de ziekmeldingen is er sprake van een keuze, een balans die doorslaat, ga ik wel of niet naar school.

**D en Z: Dus 5 procent van deze kunnen echt niet.**

**Z: En 95 procent zouden eigenlijk wel kunnen komen als..**

En die maken een keuze en het is belangrijk om erachter te komen en dat is het doel van jouw gesprek, wat maakt dat die balans van jou zo vaak doorslaat naar ziekmelden?

**D: Maar u bedoelt van deze vmbo leerlingen dan is dit wel meer dan 10 procent van het aantal leerlingen dat op school zit.**

Dat verbaast mij niet, dat wist ik. De frequentie van ziekmelden van leerlingen ligt 4/5 keer zo hoog dan van werkend Nederland. Dus dat is wel een probleem.

**Z: Dat is wel vaak, die 2 procent die zich ziek meldt. Twee keer gemiddeld ziek melden per jaar**

Anderhalf zelfs**.**

**Z: Die anderhalf heb ik gebruikt en dan zitten ze mij aan te kijken… ik maak er wel twee van.**

Ervaart deze school ziekteverzuim van leerlingen als een probleem?

**Z: Ik vind van wel.**

Wat is het probleem?

**Z: Te snel ziekmelden.**

Wat voor last heeft de school daarvan?

**Z: Het niet bij kunnen zijn met de didactische stof, het achterblijven van cijfers en daardoor soms weleens niet over kunnen gaan of zelfs geen diploma kunnen halen, bij sommigen is het echt dramatisch.**

Is dat een probleem van de school.. of?

**D: Op directieniveau ervaren we het zo niet..**

Daar zie ik heel veel verschil tussen.

**Z: Tja, is het een probleem van de school….**

Wat vindt u als directeur?

**D: Elke school heeft zijn problemen en als ik een top 10 moet samenstellen van wat de school heeft komt dit niet voor.**

Wat komt er wel voor dan? Wat staat er in de top dan?

**D: prestaties op het ogenblik, resultaten halen, dus vandaar die link met he. Dat geeft wel een aardig raakvlak. De hele zorgontwikkeling die eraan komt met moeilijke kinderen. Ik noem er een paar maar zo kunnen er wel meerdere he… maar het wordt dan niet echt bestempeld als zijnde het probleem van verzuim.**

**Z: Ik zie het ook als een soort van opvoeding, ik zeg van als ik me 4 keer ziek meld in 12 weken dan moet ik ook bij de directeur komen, dan gaat hij ook vragen van je bent nu al 4 keer ziek geweest. Als je straks werkt dan kun je niet zomaar thuis blijven en ziek melden. Zo vergelijk ik het ook wel, als een arbo-arts, niet zo streng maar..**

**Dat was jaren geleden wel in de top 10, het personeelsverzuim.**

Daar is nou al op in gezet he

**D: Ja en dat zie je ook..**

**Z: Nogmaals he en dat zeg ik echt niet, het is heel leuk om te doen het kost heel veel werk maar het zou me zo tegenvallen als het niet het resultaat zou zijn.. al gaat het maar een beetje naar beneden. Dat zou ik wel heel erg vinden.**

Ik kan je geruststellen dat het vergeleken met vorig jaar naar beneden gaat, op individueel niveau. Maar de vraag of je het op schoolniveau kunt aantonen omdat het toch om een gering aantal om een percentage gaat, dat blijft natuurlijk laag. Maar de echte frequente verzuimers haal je eruit. Maar we zagen dat het toch de duur is, die hele omvang neemt toch wel af. We hopen het wel, afgelopen jaar zijn we vooral bezig geweest om uit te zoeken hoe we de afname van de verzuimomvang op schoolniveau kunnen meten. Gezien de mogelijkheden binnen het registratiesysteem. We zagen toen op individueel niveau dat het wel degelijk een afname was. Dus dat hoop ik ook echt… ik hoop het wel aan te kunnen tonen. Maar het ziekteverzuim onder leerlingen.. daar zitten wat raakvlakken denk ik, met de problemen die u zelf wel zegt.

**D: Maar ik zit ook maar een beetje bewust te worden van het hele gebeuren, want dat is wel wat nu, als je het vraagt, goeie vraag hoor. Is het een probleem geweest voor de school? Is er ooit zo, nu wordt die bewustwording wel op gang gebracht dat het wel flinke raakvlakken kan hebben. Want ik praat namens mijn collega’s ook hoor, dat dat ziekteverzuim, dat is op directie echt niet als groot probleem gezien wordt.**

Hoe komt dat? Dat het niet gezien wordt?

**D: Ja ik denk omdat het gedekt is, en als het gedekt is hebben wij geen sorus met de leerplicht.**

**Ik maak het simpel he.**

Ja alsjeblieft..

**D: en op het ogenblik is dit wel heel erg hot en heel erg actueel en vandaar kom ik toch wel telkens terug op datgene wat ik net wilde zeggen, de verantwoordelijkheid.**

Ja dat mag.

**D:** *Over die verantwoordelijkheid, aandacht voor ziekteverzuim staat niet op onze agenda. Maar ik er zo over nadenk, het vergroten van de ouderbetrokkenheid bij de school staat wel op de agenda en heeft een hoge prioriteit. Op dit ogenblik is de ouderbetrokkenheid ook een van de 10 punten waar wij ons druk over maken. P_6a_* **Er lopen wel een aantal van die trajecten, hoe we dat nou vorm kunnen geven. Heel lastig he. Heel moeilijk he. Maar dat gaat wel heel erg over tot wiens verantwoordelijkheid hoort nou wat he. En dit, ik zeg het heel duidelijk, dit is de verantwoordelijkheid van de ouders he.**

Ja.

**D: En in hoeverre helpt dit bij die verantwoordelijkheid bij de ouders neer te leggen, en die betrokkenheid toch te krijgen. In dat hele complexe gedoe wat speelt er dan voor rol? Dat heeft ook heel erg te maken met grenzen die we als school willen gaan stellen. Het heeft heel erg te maken met wat is onze basistaak als school die we leggen. Wat is wat de inspectie ons op dit ogenblik constant in de nek hijgt.**

Wat is de basistaak van de school?

**D: Onderwijs geven, zorgen dat de leerling met een bepaald capaciteitniveau binnen de gestelde termijn eraf gaat. Komt er snel uit. Maar..**

Voor wie doen we dit? Voor wie doen we dit verzuimbegeleiding?

**D: Ehm… (duurt lang) ja, voor de leerling. Voor het kind. Voor het kind. Nogal wiedes. Alleen langzaam maar zeker zijn die verantwoordelijkheden die de scholen hebben gekregen en hebben genomen, moet ik er ook bij zeggen, die koers die wordt verlegd om die verantwoordelijkheden daar neer te leggen waar ze horen. En dat dit, het schoolverzuim dat ziekteverzuim, dat daar niet op de volle manier mee wordt omgegaan, is de verantwoordelijkheid niet zozeer van de school maar van de ouders.**

Daar blijft het denk ik, het ziekmelden blijft bij de ouders. dat blijft hun verantwoordelijkheid.

**Ik zou het zelfs heel fijn, wat ik eerder al zei ik zou het zelfs heel fijn vinden als dit proces waar we nou op i steken, dat ouders een bewustwording krijgen of dat ze zich daar heel erg bewust van worden ik kan mijn kind niet zomaar ziek melden voor elke keer als ie geen zin heeft om te komen, of het is maandagochtend of het is.. ja.. en dat is natuurlijk wel veel gevraagd. Dat krijg je zo één, twee drie niet... denk ik.**

**Z: Dat is een kwestie van doorzetten.**

Nou dat is denk ik op de lange termijn, hopen we. Ja…

**D: Nou oké publicaties kunnen.. ik kan me zo voorstellen in jullie hoop, wat jullie ermee willen bereiken, dat publicatie hier omtrent, dat dat ertoe kan bijdragen, dat ouders denk van, ja ik heb er toch voor te zorgen dat mijn kind naar school toe komt, en ik ben toch wel een keertje te makkelijk om mijn kind ziek te melden. Dat soort bewustwording toestanden dat ook bij ouders terecht gaan komen.**

Dus dat heeft er nu wel een heel belangrijk effect op denk ik. Niet alleen bij school maar ook bij de ouders denk ik, ook bij leerlingen toch een stukje bewustwording denk ik.

**D: Ja, van leerlingen vind ik nog wel lastig. Van het beeld als leerling. En als je enigszins… je moest stevig in je schoenen staan als leerlingen dat je.. je zelf aanpakt als je een rede vindt om niet naar school te gaan, dat je dan toch gaat.**

Als je googelt op schoolziekteverzuim dan vind je ook heel veel opmerkingen van leerlingen, nou ja als je toch geen zin hebt meld je je toch gewoon ziek. In die zin.. daar is ruimte waar het bij ongeoorloofd schoolverzuim eigenlijk niet de minister zich er enorm op heeft ingezet.

**Klopt**

Dat kan eigenlijk helemaal niet meer, en we hebben het idee dat de afgelopen jaren het ziekteverzuim enorm is toegenomen. Omdat dat nog een legitieme rede is om weg te blijven, en ouders daarin kennelijk meegaan, of niet mee gaan, het niet weten. Ik weet niet eh.. uit welke gezinnen worden de meeste kinderen ziek gemeld? Mag ik dat zo zeggen, hebben jullie enig idee?

**D en Z: Nee..**

Nee?

**Z: Nee.. allochtoon, autochtoon**

Als je naar de thuissituatie kijkt van deze kinderen, die zich vaker ziek melden**.**

**D: (..) in het verleden wist ik het wel, maar ik weet het echt niet meer.**

**Z: Nee nee, want er zitten hele sterke ouders tegenover je, en hele zwakke.**

Ja, nou dat kan.

**Z: Daar zie ik echt even geen lijn in.**

Nee

**Z: Ze zwaaien altijd wel, ik ga toch gewoon naar de huisarts. En dan ga ik altijd verder met, want het is geen vermanend gesprek he. Daar gaan ze wel heel vaak naar toe van..**

Vraag ik me af,

**Z: Sorry?**

Gaan ze echt?

**Z: Ja jeetje..**

**D: Wordt nog belangrijk met eigen bijdrages..**

**Z: Daar ga ik eigenlijk klakkeloos vanuit.**

Je gaat er vanuit dat het waar is wat ze zeggen.. De huisarts ziet de kinderen niet. Huisarts zegt, in het algemeen zegt de huisarts, wij zien kinderen van deze leeftijd nooit.

**D: Dat was vroeger met die briefjes, weet je wel, kon je om briefjes vragen.**

Dat mag nu niet meer**.**

**D: Dat is allang afgelopen.**

Briefjes vragen?

**D: Een briefje van de huisarts, neem een briefje van de huisarts mee dat je geweest ben. Ik heb dat vroeger toen ik Rotterdam werkte gedaan, een briefje van de huisarts dan? Toen kwam het op een gegeven moment dat het niet meer mocht.**

De laatste 10 jaar mag dat niet meer.

**D: Toen ik daarop kwam, thuis was het heel erg op wijsbaar, waren er allochtonen meisjes die het werk moesten toen he. Een hele toestand.**

In de literatuur en als je praat met huisartsen, zij zien kinderen niet van deze leeftijd. Deze kinderen met al hun klachten. We weten wel uit de literatuur dat 10 tot 15 % van de kinderen regelmatig klachten hebben. Ze zijn moe, hoofdpijn, wat dan ook allemaal. Maar die komen niet bij de huisarts. We hebben.. gaan we even terug naar ziekteverzuimbegeleiding. We hebben twee manieren om dat vorm te geven, jullie hebben nu M@ZL-smal, daar was ook geen keuze in. Want dat had ik in de aanbieding om mee te doen aan het onderzoek. Dat betekent dat jij de gesprekken voert, zelf bepaalt wat je omvangrijk ziekteverzuim vindt, je hebt onze criterium gehanteerd, hoeft niet maar dat kan. Je hebt zelf gesprekken die je kunt doorzetten. Maar M@ZL compleet, wat we eerst ontwikkeld hadden was dat aan de hand van die criteria de kinderen meteen doorgestuurd werden naar de jeugdarts. Wel met kennisgeving aan ouders en even een belangstellend gesprekje, en dan krijgen ze een bericht thuis dat ze worden uitgenodigd door de jeugdarts. Je kunt je voorstellen dat die 76 gesprekken die jij nu voert de jeugdarts voert bij M@ZL compleet. Die ziet al die kinderen. Als jullie mogen kiezen tussen deze twee opties waar zou je dan voor kiezen?

**Z: Mag ik heel eerlijk zijn?**

Ja je mag.. ja alsjeblieft!

**Z: Compleet.**

Ja?

**Z: Want ik vindt het wel hartstikke belangrijk maar wat jij zegt andere dingen komen een beetje in de verdrukking zeg maar.**

**D: formatief is het een probleem.**

Ja. Stel dat je die formatie had, die tijd is geen probleem.

**Z: Oh zo, dat.**

**Ja..**

Ja we hebben het nu even niet over… we kunnen er veel over zeggen want M@ZL-compleet zoekt steeds duurder. Want die arts moet betaald worden. Daar wil ik dadelijk nog uitgebreid over hebben met jullie, over financiën. Maar laten we het even hebben over.. dat is geen punt. Tijd zat. Wat zou jij dan toch..

**Z: M@ZL-smal.** *Want dat gesprek met die ouders, als het gelijk naar de jeugdarts zou gaan, dan zien de ouders niet de link met school, dat school dat.. dat school ze in de gaten heeft, dat er een gesprek komt. Dan kun je ook zeggen het is een gesprek uit zorg.* *S_6b_* **Daar komen ook nog, wat ik toen straks zei, ook andere dingen uit.** *Want de leerling kan niet lekker in zijn vel zitten, kan gepest worden, S_6a_* **dat een kind eh.. en als deze problemen blijken dan kan de docent er zelf mee aan de slag. En natuurlijk hebben die mentoren ook regelmatig gesprekken met die ouders, maar dit is weer een andere invalshoek.**

Mmm.

**Z: Er komen soms toch verassende, gekke dingen uit.**

Dus het levert op.

**Z: Het levert op. Ja. Ja.**

**D: Ik denk dus niet, ik denk dat als het niet gaat over formatie, maar goed dat is mijn petto. Ik snap het ook want dit gesprek heeft meerwaarde, als begeleider e.d. maar tegen de achtergrond wat ik net zei ieder zijn eigen taken, zijn eigen verantwoordelijkheden zou ik voor de andere vorm kiezen.**

Kunt u dat uitleggen?

**D: De school is zich aan het ontwikkelen als steeds minder een, nou moet ik dat goed zeggen, ja. Als steeds minder een zorgstelling, en moet zich steeds meer gaan beperken tot datgene waar ze voor bedoeld zijn. En dat zeg ik die zich juist héél erg met zorg is bezig geweest. Maar aan alle kanten wordt er aan getrokken en aangeknibbeld en gestuurd en… ik weet, ook omdat je allemaal andere verantwoordelijkheden meer gaat krijgen in de kader van prestaties, levering van personele problemen die er zijn e.d. moet je proberen daar de zorg weg te krijgen waar die het best in handen is. Dan zou ik in dit geval dus zeggen direct naar de jeugdarts. Maar dan moet je dus ook proberen sociale emotionele problematieke weg te leggen bij centra van jeugd en gezin. Zoals we ook proberen allerlei remedial-teaching-achtige en huiswerkbegeleidingconstructies weg te leggen bij instituten die daar voor zijn. Ik zeg dit heel zwart-wit voor de duidelijkheid he.**

Ja dat mag, doe maar gewoon.

**D: 10 jaar geleden zou er gezegd worden van doe dit allemaal zelf probeer het zoveel mogelijk zelf te doen. Dat hebben we ook heel veel gedaan maar daardoor wordt je wel heel veel verknocht met de zorg. Dat op een gegeven moment het verwijt ook wel eens kan gaan klinken van school je bent te veel een zorginstelling. Sterker nog, de ervaring die ik in het verleden heb meegemaakt, je kan zo goed zijn in zorg, dat je in mum van tijd een zorginstelling bent. Waarbij je de andere kinderen waar je voor bedoeld bent, afstoot. Even de achtergrond he..**

Ja oke.

**D: Waar het om gaat he.**

Je trekt de verkeerde leerlingen aan.

**D: Ja**

Ja ik zeg het ook maar zwart-wit even.

**D: Het gaat helemaal tegen mijn principes in.**

Ja ik voel je pijn.

**D: Ik eh..**

Hoe zit het dan met de ontwikkeling van zorg-in-en-om de school?

**D: Ja daarom.. dat is heel goed, daarom moet je die lijnen wel goed hebben en die contacten hebben. En daarvoor moet je die oplossingen als scholen wel hebben. En daarvoor heb je een goede zorgstructuur nodig en een goede zat, waarbij al die contacten heel erg aanwezig zijn en je dus heel snel bij signalering doorverwijzing.**

Dus signalering is een taak van de school?

**D: Ja.**

Oké

**D: Ja, zonder meer, dus signalering ga daar maar aan de slag. Maatschappelijk werk. Ga daar maar aan de slag. Zo is het he. In het ZAT komt alles bij elkaar… ik ehm.**

Dan hebben we het over, u zegt wel het is wel een taak van de jeugdgezondheid.. om hiermee aan de slag te gaan. Ik zie twijfel.

**D: Ja ik zit even.. dat is een goede vraag moet ik even over nadenken. Ja.. Ja. Oké. Ja dat denk ik wel. Ik moet even inbeelden hoe dat zit bij jeugdgezondheidszorg.**

Wat weet u van de jeugdgezondheidszorg?

**D: Ja ik weet niet… wat weet ik ervan..? ik weet dat.. we zitten hier met een jeugdbeleid en we zitten hier met een gezondheidsbeleid. En als de jeugd daar gaat zijn er een aantal speerpunten waar mee ingezet is. Want daar zijn signaleringen. Weet ik niet, dat dacht ik, die naar voren zijn gekomen. Is dat herkenbaar? Ja dat is herkenbaar. Wie heeft daar last van? Ik denk dat veel mensen hebben daar last van. Dan praat je over verslavingsvormen, je praat dan over te dik zijn. Even 2 speerpunten die naar boven komen. Daar moet nodig wat aan gedaan komen als het gaat over gezondheid, ja. Hebben de scholen daar last van? De school heeft daar last van. Hebben de ouders daar last van? Ja die hebben daar last van. Wie zijn degene van die daar het meest deskundig is, dan kom je wel bij de jeugdgezondheidszorg, denk ik. Ja.. zo ongeveer.**

Ja. En weet u.. het is geen examen of zo hè, maar ik ben zo benieuwd naar wat bekend is gewoon over de jeugdgezondheidszorg.. heeft u enigszins een idee van jeugdgezondheidszorg, hoe dat georganiseerd is, wie betaalt, waar opdrachten liggen waar..

**D: Nee nee nee.**

Heel even in het kort, de gemeente betaalt de GGD om jeugdgezondheidszorg uit te voeren, samen met de thuisinstellingen dan hè. En daar is een basistakenpakket voor afgesproken, dat is eigenlijk ontstaan ongeveer 10 jaar geleden toen men eigenlijk de policy had van nou ja iedere kind in Nederland heeft recht op dezelfde zorg en tot dan was het zo dat elke GGD zelf een beetje bekeek hoe jeugdgezondheidszorg werd uitgevoerd. Maar goed, met het decentraliseren en het feit dat de gemeenten geld kregen werd ook bij wet een basistakenpakket vastgesteld door de beroepsgroep zelf en door de inspectie. Dat is toch wel minimaal wat de jeugdgezondheidszorg moet doen. Voor het geld dat betaald wordt aan de GGD. En in dat basistakenpakket zit, dat met enige regelmaat kinderen worden uitgenodigd, al dan niet met hun ouders, voor een periodiek onderzoek, de bekende screening, en de bekende onderzoeken. En daar.. van daaruit kunnen nog een aantal acties komen, hè met het enige doel om te signaleren om zo snel mogelijk door te zetten naar hulpverlening. De jeugdgezondheidszorg heeft niet de taak om hulp te verlenen, hooguit een overbruggingszorg naar hulpverlening toe. Dus we hebben daardoor een signalerende taak. Dit is er aan de hand, een probleemanalyse en zo kan men doorzetten. En ook wel zorgen dat het inderdaad doorgezet wordt naar de GGZ, naar de huisarts of kinderarts en het monitoren daarna. Zijn ze aangekomen, wat is er aan de hand en gaat het uiteindelijk beter met het kind? Dat zijn de taken van de jeugdgezondheidszorg en daarvoor betaalt de gemeente. Wij beseffen als geen ander dat we daarvoor de school heel erg nodig hebben. We moeten heel erg samenwerken met de school want ook zij hebben een signalerende functie. En in dat samenwerken en samen signaleren en samen op de rit krijgen van kinderen kunnen we elkaar helpen en steunen, toch?

**Z: Hm-m**

Ik vertel dat eigenlijk ook wel omdat ziekteverzuimbegeleiding.. ja jammer genoeg niet in dat basistakenpakket zit nu. Er zijn al gelukkig geluiden dat dat weer wat losser gelaten mag worden omdat er vanuit de werkvloer eigenlijk heel veel bezwaar komt, steeds meer. Want we willen samen met scholen kijken hoe wij de middelen voor jeugdgezondheidszorg het beste in kunnen zetten. Maar u, was u bekend met het screeningen? Met de pgo’s, ik zag u toen wel even knikken.

**D: Die keuringen, ja. Die zijn voor de vmbo leerlingen hè.**

Nou, het is wel voor alle kinderen maar ook de GGD is aan het zoeken naar ruimte om een deel van ziekteverzuimbegeleiding wordt nu ook wel betaald doordat we iets minder aan die screeningen.. hè, hoger onderwijsniveaus worden kinderen nog wel gescreend maar niet allemaal gezien door de jeugdarts of jeugdverpleegkundige. Daar halen we wat tijd vandaan om.. maar dat is dat klas 2 leerlingen gescreend worden.

**D: Dat was, oké, daar zijn verschillende gemeenten verschillend mee omgegaan in het verleden en nog steeds. Dat monitor project, daar worden alle kinderen uit leerjaar 3 bevraagd en daar kwamen uit enquêtes speerpunten uit naar voren wat voor grote problemen er waren en daar werd dan op ingezet om dat te voorkomen. Van suïcidaal gedrag tot wapenbezit en dat soort…. Maar de gemeente betaalt, maar dat zijn ‘de gemeenten’.**

18 gemeenten

**D: De RMC-groep**

Ongeveer gelijk aan onze 18 gemeenten West-Brabant**.** Jullie zitten niet bij RMC-regio34? Bergen-op-Zoom niet, toch?

**D: Nee, dat klopt.**

Dat komt ook dat M@ZL-compleet betaald moet worden.

**D: En het jgz-beleid van de gemeente Halderberge? Is dat ook gebaseerd op datgene wat jullie….**

Die linken zouden beter kunnen. De screening houdt zich vooral bezig met het individuele kind, signaleren en doorgeleiden. Daaruit komt wel informatie om op groepsniveau jeugdbeleid te maken. We hebben ook vtv’s, jeugdmonitoren, die daarvoor ook input leveren. Alles heeft raakvlakken, komt allemaal bij elkaar. JGZ heeft in eerste instantie toch de focus op het individuele kind. Van daaruit wordt op collectief gemeenteniveau beleid geadviseerd.

**D: Nee, ik snap het verhaal. Met andere woorden, jij denkt… lacht…**

Wat denk ik?

**D: Oké, kaatsballetje 1 dat hoort niet bij de school, dat hoort dus bij jgz. De directe aanpak niet, want dat is eigenlijk wat je zegt. Want daar zijn we ook niet voor. Maar dan ondersteunend aan het vervolgtraject.**

En signalerend, he, we hebben een heel belangrijke signalerende taak, net zoals jullie dat hebben, en we hebben een heel belangrijke taak in het monitoren van de gezondheid en de hele ontwikkeling van het kind, niet alleen lichamelijk maar ook psychisch en sociaal. Een jeugdarts/verpleegkundige pakt het totale ontwikkeling van het kind en zijn omgeving. We hebben een taak in het monitoren en doorgeleiden. En we hebben een taak in het vertalen van individuele gegevens naar het collectief, en de gemeenten te adviseren met betrekking tot jeugdbeleid. Zo is het wel geregeld. Je hebt drie partijen, de gemeente, school en jgz. Tenminste zo zien wij het.

**Z en D: ja,ja…**

Ouders en leerlingen ook, maar om het inhoud en vorm te geven.. Stel nou dat we…

**D: Ja, want ik zit hardop te denken. We zitten in allerlei ontwikkelingen, de zorg komt steeds meer onder regie van de gemeenten.**

Ja, ook de jeugdzorg, hé? Ook voor ons.

**D: Ja, wij zitten met de worsteling van het passend onderwijs e.d.**

Het moet allemaal weer normaliseren, dus al die rugzakjes moeten weg en het moet weer passen in het reguliere onderwijs, van zorgstructuur naar begeleidingscultuur. Dat is bij ons ook. Jeugdzorg moet minder, we moeten veel meer preventief gaan werken. Ziekteverzuimbegeleiding, maar dat is natuurlijk mijn persoonlijke mening, kan daarin een heel belangrijke positie innemen, omdat je daarmee een groep kinderen signaleert. Je hebt dan ook recht van spreken en een reden om ze uit te nodigen en met ze in gesprek te gaan. Stel nou dat wij zeggen we stoppen met die screeningen en we gaan ziekteverzuimbegeleiding doen, hoe zouden jullie dat vinden?

**Z: Wil je dat verder uitleggen?**

Nu worden alle leerlingen van klas 2 uitgenodigd voor een onderzoek, daar stoppen we mee en in de plaats daarvan krijgen jullie ziekteverzuimbegeleiding.

**Z: ja doen.**

Waarom?

**Z: Ik denk dat het effect daarvan en weten dat daar ook mensen niet komen opdagen, dat dat effect minder is dan dit. Dat je echt gericht gaat kijken naar die leerlingen die uitvallen. Daar ben ik een grote voorstander van.**

Hoe ziet u dat?

**D: Ik denk dat het waar is wat Z zegt. Ik ben ook eerlijk gezegd een beetje het spoor bijster van wat er met de nabespreking van die periodieke keuringen gebeurt. In die 10 jaar dat ik nu hier werk, krijgen we het verslag ervan, krijgen we te horen wat er gaande was.**

**Z: Nu sporadisch. Als er iets is, de verpleegkundige (naam) koppelt dat terug.**

**D: Oh ja, …. (naam jvl). Maar ik denk dat dit wel misschien wat meer rendement heeft voor de school in ieder geval. Ik weet niet hoe het met de andere partijen zit. Ik heb echt niet het zicht wat die keuringen, ik kan me ook voorstellen dat daarmee dingen naar boven komen waarmee mensen heel erg blij zijn dat die ontdekt zijn.**

**Z: dat komt hiermee ook.**

Er is een overlap.

**D: Ja, daar is een overlap, maar niet dekkend, denk ik. Er zijn ook kinderen die gewoon op school komen en waarmee wel wat is, toch? Die komen dan niet in beeld.**

**Z: Maar die komen uiteindelijk wel in beeld. Als er iets is, en als ze het niet meer kunnen handelen. De ene met migraine zegt het hoort erbij en die gaat wel gewoon naar school en de ander zegt ik voel hem al aankomen, ik ga plat. Migraine is misschien niet het goede voorbeeld… En als zo iemand dan niet … trekt, dan valt zo iemand heus wel uit.**

**D: Ja, als je mag kiezen. Ik ben er te weinig van op de hoogte om direct antwoord te geven maar ik neig er naar te denken dat het ziekteverzuim meer rendement oplevert, voor school, voor leerlingen….. Ik denk het wel.**

Als de kinderen naar de jeugdarts zijn geweest, zie jij ze dan nog, zie je het dan ergens terug? Heb je dan nog een terugkoppeling met de leerling zelf, of via de mentor?

**Z: nee, de mentor volgt ze. Als het goed gaat hoor je meestal niets. Sommige mentoren praten er toch nog over, daar moet ik het van hebben. Ik zie ze zelf niet meer.**

Gaan de leerlingen zelf uiteindelijk ook inzien dat ze er iets aan hebben?

**Z: Heel wisselend. Soms zeggen ze zelf, vaak gaande het gesprek gaan we bespreken of we de schoolarts gaan inschakelen. Ik leg het nu nog niet op. En sommige leerlingen zeggen dan, ja, misschien is het wel verstandig om naar de schoolarts te gaan. En anderen zeggen, nee dat is niet nodig. En sommige ouders zeggen van misschien is het wel verstandig en andere zeggen nee het is niet nodig. En dan maak ik vervolgens de afspraak, of ik zeg oké voor mij hoeft het nu nog niet maar de volgende keer is het de standaardprocedure, en ik verkoop het dat is door de school zo afgesproken. Dat is de schoolregel. Even een belletje naar huis en dan… Ik zie de leerling zelf dan niet.**

De andere zorgcoördinator (naam) zit hier niet bij. Op havo-vwo loopt het nog niet, je zei ze het niet geregeld krijgt. Is dat de enige reden denk je?

**Z: Ja, dat weet ik zeker. Ze is wel een felle voorstander hiervan…. Ja, ik weet niet of dat dan een kwestie van prioriteiten stellen is, dat weet ik niet, dat kan ik voor haar niet vertellen, maar ze is wel een felle voorstander hiervan.**

Ze heeft in de wandelgangen niet aangegeven dat er nog andere hobbels genomen moeten worden?

**Z: Nee, het is puur tijd.**

Ik kan me er alles bij voorstellen… We hebben het er al eerder over de telefoon over gehad. Dit ga jij niet volhouden. Wat moet er veranderen, hoe kunnen wij je ondersteunen dat je het wel gaat volhouden?

**Z: Dat is een moeilijke vraag. Kijk, technisch gezien zou het voor mij een walhalla zijn als we nu konden overstappen op M@ZL-compleet, bij wijze van hè?**

Zodat jij niet meer al die gesprekken hoeft te doen.

**Z: Ja, en wat dat betreft ben ik wel eigenaardige. As ik me ergens in vast bijt wil ik het ook afmaken. Dat is mijn valkuil.**

Ook je sterke kant… Of zou er binnen school nog iets kunnen veranderen?

**Z: Dat het de mentoren gaat, dan. Maar dan lijkt de werkdruk bij mij weg. Maar dat is niet. Je moet het coördineren.**

**D: en het verwatert**

**Z: en dan is het fysiek wel gemakkelijker, dan heb ik die gesprekken niet, maar dan gaat het hier overlopen. Oh ja, daar moet ik nog een terugkoppeling van, heeft die het gesprek al gedaan, snap je?**

Als zorgcoördinator voel je je verantwoordelijk ervoor dat het goed gaat?

**Z: Ik vind het niet eerlijk, zeker ten opzichte van de leerling niet, want we doen het uiteindelijk om ze te helpen, dat mentor a zegt ik doe het niet, omdat hij het niet ziet zitten, mentor b kan het niet, mentor c doet het hartstikke goed.**

Maar we hebben het nu over ziekteverzuim. Er zit een groot verschil tussen mentoren, ook ten aanzien van heel veel andere taken. Ten aanzien van heel veel mentortaken is er heel veel verschil tussen mentoren, wordt het verschillend opgepakt. Daar maak je je misschien ook druk om?

**D en Z: oh ja, zeker. Dat is ook een punt van zorg.**

Als ik met landelijke partijen praat zeggen ze allemaal dat dit echt tot het mentoraat hoort om zorg te hebben voor kinderen en met hen en hun ouders in gesprek te gaan, ook over ziekteverzuim. Dat hoort ook weer bij passend onderwijs. Wat moet er nog gebeuren? Moet dat mentoraat anders georganiseerd worden? Wat kan ik de politiek meegeven?

**D: Ik denk dat het waar is dat het op mentorniveau hoort, ik geloof dat ook en ben er ook helemaal voor, maar ik snap wat Z bedoelt, ik ken ook verschillende mentoren, het totaalbeeld is hij dan kwijt, dan varieert het zo in aanpak. Dat is op alle scholen zo.**

Dat weet ik. We zijn nu nog aan het piloten. Stel nu dat je het wilt implementeren, dan moet het echt in de begeleidingscultuur van de school verankerd zijn.

**D: Dan is dit geen ander onderwerp als alle andere onderwerpen waar het gaat over mentoren en begeleiding. Wat jij ook zegt, ik geloof dat het helemaal waar is.**

**Z: Over het algemeen voelen de mentoren ook de druk. Dat is heel gevarieerd.**

Welke druk?

**Z: De werkdruk. Ze moeten behandelplannen schrijven en daar is bij sommige al heel veel weerstand.**

Omdat?

**Z: Geen tijd voor.**

**D: Administratieve zaken worden sowieso heel lastig gevonden.**

**Z: Advies aan de politiek: bv. je hebt de transfercoach, die zorgt voor leerlingen, begeleidt ze naar het mbo. Misschien dat zulke mensen dit soort dingen gaan doen. Misschien een verzuimcoördinator.**

Ter ondersteuning van de mentor?

**D: ja, maar daar hangt dan een zak geld aan en die krijg je niet. Dat is wel waar, het ideale plaatje.**

Je mag hier fantaseren.

**D: Dat is waar, als het zo kan, dan graag. Maar dat kun je op je buik schrijven. Want dan wordt het doorgeschoven naar de scholen, jullie moeten het doen binnen jullie formatie, maar die scholen moeten ook bezuinigen.**

Maar goed de mentoren krijgen meer tijd, tijd is geen punt…

**D: Ik probeer wat anders te zeggen. Ik wil het graag hebben over de havo-vwo-mentoren.**

**Z: Ja, dat moet je doen.**

**D: Jij hebt het beeld van vmbo-mentoren. Hoe zeg ik dat?**

**Z: Die lopen een beetje achter.**

**D: Ja, die lopen sowieso achter, maar door de dagelijkse gang van zaken en de problemen waarbij de mentoren van de laagste streams in het vo merken van hé met alleen puur lesboer zijn komen we er niet is er een bewustwording ontstaan we moeten meer aan begeleiding doen en langzaam maar zeker zie je dat het van beroepsgerichte leerwegenniveau zich uitbreidt naar andere niveaus zoals mavo en zo moet dat binnen de havo en het vwo gebeuren. Neem een voorbeeld, neem de extremen, op het vwo, de mentor heeft een makkie tov de beroepsgerichte leerwegenmentoren. Daarbij komt, ik heb er heel wat aangenomen de afgelopen jaren, dat het type docent op het vmbo gaat voor de leerling, die was net zo goed bij de jeugdzorg terecht gekomen, ik zeg het even zwart-wit, en het type docent op het vwo gaat voor het vak, die voelt zich daartoe geroepen om dat over te brengen. Kom je in het eerstegraadsveld is het helemaal extreem, met drs-titel, we zijn er toch voor het vak om de leerlingen op zo hoog mogelijk niveau af te leveren, die hebben héél weinig affiniteit om leerlingen te begeleiden, terwijl de ontwikkelingen zoals deze en ook van passend onderwijs die vragen ook van hen een andere insteek als docent en ook al staat het in de categorieën / functieomschrijvingen (lb, lc en ld) daar staat tegenwoordig in beschreven (lc en ld) ‘kan omgaan met meervoudige problematiek’. Die lb docent moet kunnen omgaan met enkelvoudige problematiek, terwijl daar juist de meervoudige problematiek zit. Die ld-er zegt maar daar ben ik niet voor aangenomen. En in dat zelfde kader past dit verhaal ook. Daar is nog een hele lange weg te gaan, en dat is moeilijk hoor, vandaar die weerstanden tegen passend onderwijs en dergelijke, voordat die groep docenten, die havo-vwo-ers met name, zich ervan bewust gaan worden dat het tot hun takenpakket hoort. Ik ben niet alleen die leesboer Engels die tot Cambridge Engels allemaal afgeeft. Nee, ik moet ook zorgen dat die leerling, en dan komt het verhaal wat me het laatste halfjaar heel erg bezig houdt, je hebt als taak en als professionaliteit om die kinderen op dat niveau af te leveren wat hij in feite in zijn hoofd heeft zitten. En dat is niet alleen maar door lessen te geven maar ook een stukje begeleiding en zorg te geven. En dat is een ommezwaai van denken.**

Ja**.**

**D: En daar is nog het nodige in te gebeuren, en dan moet het ook afgezet worden tegen rendement. Dus die insteek die jij doet als mentor dat je niet alleen zegt van hé dat zijn Engelse woordjes, maar je bent een aardige jongen hoor en je kan het heel goed en het is leuk zoals je dat doet. Alleen dat positieve kan het rendement heel erg verhogen. En, dus dat is de begeleiding die hij moet gaan doen. Die omslag, met vwo zijn we daar dan mee bezig want het vwo heeft te laag gescoord dus de inspectie zit daarop te sturen, en die heeft ons de opdracht gegeven, daar hebben we het vaker over gehad van wat bedoelen ze nou? Het rendement van de zorg moeten we aantonen. We moeten aantonen van wat doe je nou? Wat is nou je voortgang met alle zorgmiddelen, we besteden ruim 2 ton per jaar aan de zorg, maar waar blijkt dat nou uit? Ik worstel me eigen dood daarmee, we hebben een zorgteam, we hebben dit en we hebben dat. Dat is allemaal leuk en aardig maar voor de rendement van de zorg levert het niet veel op. Nee, ze komen in klas 1, krijgt toetsen, scoort die en die formules, en gaan naar klas 2. Wat haalt hij dan voor die toetsen? En als hij daar slecht voor scoort, wat doe je dan als school om hem hoger te laten scoren? Die zorg moet je hem bieden.**

Dat zou mooi zijn.

**D: Dat is wat de inspecties ons tegenwoordig vragen, en we zijn te veel, dat bedoel ik ook te zeggen met de worsteling waarmee ik zit, we zijn te veel, en vooral op het vmbo gebied, te veel naar de sociaal emotionele zorg doorgeschoven en met kinderen die in de ellende zitten en waar je je eigen als een.. waar je op je eigen gevoel denkt God die moet er toch doorheen, die zal toch ook goed door de middelbare schoolleeftijd heen moeten komen en wat een ellende heeft hij thuis en wat kunnen we daar nou voor doen? Daar zijn we heel erg in doorgeschoten, misschien te lelijk gezegd, maar we zijn er heel goed in geworden. In de loop der jaren, waardoor we dat als heel vanzelfsprekend zijn gaan vinden dat we dat doen in bepaalde takken van het onderwijs en daarmee zijn ze uit elkaar gedreven, het vmbo en het havo-vwo om in die term te blijven. En nu worden we eigenlijk weer als het ware gedwongen van hé jij terug en jij terug en dat moet de zorg zijn. Een moeilijk verhaal hoor wat ik vertel, maar je begrijpt een beetje wat ik bedoel?**

Ja, maar dat is helemaal maatschappij breed**.** Wat ze in de arbeidsgeneeskunde zeggen: ‘praten mag werken moet’. Beetje hetzelfde hè, jullie mogen heel veel begrip hebben, en het is heel goed en we kunnen daar van alles voor regelen, maar uiteindelijk moeten jullie presteren en naar school gaan en ook die eisen stellen. Dus dat is een beetje terug naar.. wat ik ook gemerkt heb toen ik heel veel op scholen nog in de uitvoering zat, dat allemaal maar begrijpen waarom ze hij het zo moeilijk heeft en niet zeggen maar ja toch ook eisen stellen en verder gaan want daar ligt je toekomst, daar moet je naartoe. Ik weet niet of u dat ook bedoelt, daar zijn we in doorgeschoten, in het begrip en begeleiden maar we moeten ook presteren. Anderzijds is het ook op havo-vwo, presteren.. je presteert beter als je gezond bent, als je goed in je vel zit. Dat heeft natuurlijk alles met elkaar van doen.

**D: Diezelfde discussie hebben wij ook gehad bij de oprichting van die Haldenberg, was een van de eerste bij CJG hè. K2 begeleidde dat toen en toen hadden we ook eh.. daar zijn we erg bij betrokken geweest, toen hadden we ook interviews gehouden toen kwam het ook naar boven. Wat zijn nou de taken van de scholen. In hoeverre vind je dat je allerlei dingen moet doen, toen kwam dat ook naar boven. Dat is al enkele jaren geleden. En het zijn langdurige processen hoor.**

Ja..

**D: Want als het zo is als het zo zou moeten zijn, nou ik denk dat je over zo’n hele school met 200 personeelsleden, dan ben je een end verder. Dat duurt nog wel even hè**

Ja dan is er wel een tijd overheen.

**Z: En toch ontkom je er vooral bij de lagere streams niet aan om die zorg te bieden. Dan zit je wel op school met die..**

Ja maar dan is de vraag wie die zorg biedt, hè. En tegelijkertijd toch eisen stellen en verdergaan.

**D: Wat wij hier proberen te doen, daar zijn we een beetje mee bezig, om al die partners die die zorg als professionaliteit hebben, haal die in en rondom de school en dan kom je bij zorg.. houd die lijnen zo kort mogelijk. Je zou eigenlijk een constante club mensen, zo’n plusvoorziening b.v. bij school moeten hebben, van hè die ontspoort en zet dan gelijk boem zo’n club erop. En zo’n team is wel heel gerelateerd aan de school maar het is niet ‘de school’. En dan houd je het heel erg..**

Maar dat zou eigenlijk het ZAT toch moeten kunnen zijn, zulk adviesteam waar mensen van extern uit de zorg bij elkaar komen om... leerlingen te bespreken.

**D: Maar in de uitvoer daar wordt weer besproken hè.**

Maar ja.. je mag er wel acties en plannen over maken hè.

**D: Dat klopt.**

**Z: Waar de zorgcoördinator verantwoordelijk is voor de lijnen die uitgezet worden, en wat jij bedoelt, zo’n plusvoorziening, wat jij bedoelt, die nemen het over.**

Die nemen het over ja, de coördinatie. Dat heb ik eerder gehoord, dat verhaal. Positief bedoelt dan.

**Z: Zo kwam het ook over**

Heel goed ik vind het wel fijn om te horen. Dan kom ik toch terug op (naam andere zorgcoördinator). Want je zegt (…) heeft geen tijd. Nou hoor ik een ander verhaal, van havo-vwo. Wat een andere cultuur is. Zou dat niet, ja dan ben ik wel heel flauw, maar dan denk ik, (…) geeft aan ik heb geen tijd voor die gesprekken. (…) zit er nu niet dus ik kan haar er niet bevragen erop, maar zou er toch niet ook wat anders zijn?

**D: Ja.. (…) doet, ten eerste is de constructie wat we nu zouden hebben met 2 zorgcoördinatoren is nieuw, want in de loop van dit jaar waren er wisselingen in want er zat een andere zorgcoördinator op de bovenbouw havo-vwo die dat als zijn eigen praktijk min of meer daar de zorg verrichte. Waarbij de docent van de zorgleerlingen zei..**

Hier heb je hem.

**D: Hè, ongeveer zo, zo, zo gaat dat. Zo gaat dat wel. Nou heeft (…) die club gekregen plus nog een nieuw iemand naast zich gekregen dus die moet met de insteek, want die heeft zelf insteek als wij, maar die heeft dus op dat terrein nog heel wat mensen te..**

Mobiliseren.

**D: Te mobiliseren. En dus daarom heeft zij ook, vergt dat heel veel tijd en energie van haar. Dat is.. ja dat is nogal...**

Want stel dat Inge wel.. 10 uur in een werkdag had, en wel tijd had om die verzuimgesprekken te voeren, zou dat dan wel van de grond komen?

**D: Van haar persoonlijk zeker.**

Nee, ik twijfel niet aan (…), daar gaat het hier niet om. Maar Z zegt zelf ik probeer zoveel mogelijk mentoren erbij te betrekken, ik koppel dat terug. Dat leeft ook onder de mentoren. Zou diezelfde ervaring (…) ook hebben? Ik hoor eigenlijk al zeggen toenet van nou….

**D: Varianten.. ik weet in de onderbouw havo-vwo, daar zitten best wel mentoren waar zij zich heel erg goed mee voor elkaar krijgt.**

Ja**.**

**D: Ik moet ook een beetje nuanceren wat ik zeg. Ten eerste is hier de campus, daar gaan die ontwikkelen weer wat beter en wat sneller om dat fysiek bij elkaar te brengen. Op de andere locatie, die zit een eindje verderop. Daar zit nog steeds een status aparte. Daar zitten nogal wat oudere, daar zitten alle eerste grades om dat zo eens te zeggen, en daar zitten ook wat oudere docenten. Er zitten acht of zo die in hun laatste jaar zitten. En die krijg je niet gek met dit verhaal om dat zo maar eens te zeggen. En daar zit je nu op te hendelen om dat zo maar eens te zeggen. Maar.. ik betwijfel echt niet erover dat (…) dit project ook wel zou willen.**

Nee daar twijfel ik geen seconde over.. nee.

**D: Maar bij mij is het wel heel erg.. maar goed dat het ik ook wel aangegeven, de afweging de tijd die er nu aan besteed wordt in relatie tot het rendement.**

Ja, dat begrijp ik ook. Ja. Ik hoop daar wel wat over te kunnen zeggen na dit jaar, dat doen we wel.. ik hoop heel er dat je nog wat vol houdt totdat wij eh.. het rendement kunnen aantonen.

**D: Want ik had het al helemaal in het begin gezegd, we verbreden dit traject hè. Je kan ook denken bij eh.. niet alleen bij jou maar bij een aantal mensen om ze erbij te betrekken, en niet alle mentoren maar je hebt ook mensen op het netvlies van oh God dat wordt moeilijk als die een gesprek daarover moet voeren. Maar er zijn wel een aantal waar het wel bij kan. Of bij de leerling-coördinatoren of bij de counselors, dat kan natuurlijk ook een mogelijkheid zijn hè.**

Ja. Ergens wil jij graag die gesprekken hoor.

**Z: Nee nee, jawel ik vind het heel leuk.**

We stoppen erbij.

**Z: Maar nee die geven ook al aan, die zitten ook al aan hun taks. Als je die met dit aankomt…**

Maar ik denk dat wil je, wil je dit echt.. dat weet ik niet of dat werkt maar ik denk wel dat het belangrijk is om daar goed over na te denken**.**

**D: Want dit gaat door?**

Wij willen het ontzettend graag, geloof me dat. Wij zullen het in het voorjaar aan alle gemeenten presenteren. Wij willen heel graag dat wij de ruimte krijgen om de scholen dit aan te bieden. Al is het maar dat ze mogen kiezen tussen de screeningen, en tussen dit bijvoorbeeld. En dan wil ik ook heel graag proberen aan te tonen met het onderzoek, wat is nou de meest effectieve vorm, wat zijn de randvoorwaarden, succesfactoren bijvoorbeeld, dat is ook het belangrijkst voor dit gesprek. En ik weet bijvoorbeeld dat de gemeente Oosterhout, dat hoorden wij woensdagochtend bij het interview, die gaan waarschijnlijk mee financieren, die vinden het zo’n goed project dat ze zeggen, wij zien dit wel. Want ook de gemeente heeft veel aan de jongeren die goed gekwalificeerd afgegeven worden en die goed in hun vel zitten.

**D: Financieel? Want gemeenten zijn op dit ogenblik flink aan de kraan aan het dichtdraaien.**

Ja.. toch is die gemeente wel bereid. Ik hoop dat ik door goed onderzoek nu te doen en rendementen aan te tonen dat de gemeentes ja zeggen. Want nu zeggen de wethouders ja.. we hopen dat op alle scholen ziekteverzuimbegeleiders komen. Zij zitten met de handen en de voeten gebonden aan onze basistakenpakket op dit moment. Daar komt wel wat versoepeling dus we willen heel graag daar in ieder geval de vrijheid voelen om.. het minste wat we kunnen bereiken om de scholen de keuze te kunnen laten maken.

**Z: Maar vervolgens als je daar bij de gemeente voor elkaar krijgt, moeten wij 5 gemeentes om krijgen.**

**D: Dan heeft het enigszins wel zin..**

**Z: Ja maar ik bedoel voor deze school dat je ze niet allemaal om krijgt. Als het alleen maar geldt voor de leerling uit een bepaalde gemeente,**

**(iedereen praat door elkaar)**

**Z: Dat werkt dus niet..**

**D: Dat is een lastige hoor. Op maatschappelijk werk wordt ook al bezuinigd.**

Wij werken met 18 gemeentes samen en wij hebben afgesproken als GGD dat wij met 18 gemeente één plan maken. We gaan dat niet voor elke gemeente apart doen … dat gaat niet

**D: En gaat het ook nog eens in afstemming met leerplicht..?**

Ja ja.. nou leerplichtzaken.. die is alleen maar blij met dit soort interventies.. we zeggen ook: gemeente jullie hebben daar heel veel voordeel van. Dat zeggen de leerplichtambtenaren ook. Kinderen die nu in beeld komen naar aanleiding van langdurig of frequent ziekteverzuim, die zijn goed in beeld. Want ze zijn al bij de jeugdarts geweest, of niet, er is al dossiervorming, dat is voor ons bijna een inkoppertje. Dat geven ze ook duidelijk aan. Dat geven wij ook terug aan gemeenten van ja jullie hebben daar ook veel aan. De leerplicht in de uitvoering ook. Dat is wat de gemeente Oosterhout ook zegt, dan gaan we daar ook wel aan meebetalen, daar zijn we ook heel erg blij mee. Dus in die zin is er al beweging. Dus.. nou ja goed.. maar we merken ook, zoveel scholen, zoveel meningen ook over hoe je dat moet gaan doen. Dus dat wordt ook nog wel.. die grote hoed waar we alles in gaan stoppen en hopelijk wat moois uit gaan krijgen. Zijn er nog.. wil jij nog een vraag stellen? Willen jullie nog wat zeggen? Nog wat mee geven?

**Z: Stel je voor dat je voor, dat geldt volgend jaar natuurlijk pas dan, zo’n M@ZL-compleet. Wat zijn de kosten dan?**

Op dit moment kost dat 13 euro per zittende leerling. Dus op een school met 1000 leerlingen kost dat 13000 euro… Ja…

**Z: En hoe.. zulke bedragen, zeg je dan van ooooh of zeg je van..**

**D: Dat gaat dus niet gebeuren.. Nee nee.. Nee ik bedoel maar, ze zeggen oh oh ja.. dat gaat over ons.. maal 1700.. daar ga je dan naar kijken.. wat wat.. Ik zie je al met bezuinigingen maatschappelijk werk betalen, daar komen wij ook niet meer bij.**

**Z: Via het CJG?**

**D: Dus de gemeente.**

Maar weet je, wij zijn absoluut aan het zoeken naar mogelijkheden.

**Interview 5. M@ZL onderzoek Datum: 19-12-2011**

**Aanwezig: Yvonne Vanneste (onderzoeker), Marlou van de Loo (semi-arts)**

**Coördinator leerlingzaken school 7 / S_7_**

**Variant: second route**

**Algemene gegevens**

Het gesprek vindt plaats op school, in de kamer van de coördinator leerlingzaken. Later komt ook de zorgcoördinator binnen, die zich op het einde nog even mengt in het gesprek. Het is wat hectisch op school bij binnenkomst, de politie is er en er moet nog even wat afgehandeld worden met betrekking tot een leerling. Er wordt genoeg tijd genomen voor het interview. Het is soms wel wat onrustig, omdat er soms iemand binnenkomt om wat te vragen, maar het interview wordt dan weer goed opgepakt. De coördinator is rustig, spreekt ook rustig, met veel pauzes, is vriendelijk en open. Er is veel non-verbaal contact en er heerst een goede sfeer. Het gesprek moet ten opzichte van de andere interviews wat meer gestuurd worden, dat zie je ook in het uitgewerkte verslag. Soms breekt Yvonne wat te vroeg in met aanvullingen / conclusies, waardoor de geïnterviewde zijn zinnen niet af kan maken. Er worden veel sprekende voorbeelden gebruikt.

**Verslag interview**

**Vragen/opmerkingen door interviewster dikgedrukt**

Antwoorden/opmerkingen door geïnterviewde in normale opmaak

**Nogmaals, fijn dat je hier tijd voor vrij wil maken. Wij kennen elkaar niet zo goed, want de directeur van jullie school, die heeft vorig jaar uiteindelijk gekozen voor M@ZL-smal. Hoe is dat gegaan verder in de school? Want jij bent degene die het nu mag gaan doen.. Kun je daar iets over vertellen?**

Nou.. ik ben degene die het nu mag gaan doen.. Tja.. nou, we hebben het er wel, eh… De directeur zag het aanbod, M@ZL-smal, wel zitten en zag dat wel als een aanwinst voor de school om onze begeleiding van het ziekteverzuim, om het zo uit te drukken, om dat te, ehm…. te stroomlijnen, om daar beter grip op te krijgen. Ik zeg, met nadruk, de directeur, zag het wel zitten. Wij, dat betekent ik en de adjunct-directeur

**Want jij bent zorgcoördinator?**

Nee, ik ben coördinator leerling-zaken. Dus ik hou me bezig met alle problemen rondom leerlingen, maar zijnde niet de zorgproblemen. Dus niet als het gaat om extra ondersteuning of als het gaat om dyslexie of wat dan ook, dat doe ik allemaal niet. Maar gewoon net zo’n probleem als we daarnet hadden (*Marlou: jongere* *in aanraking met politie*), dat is voor mij. En ehm.. wij zagen dat niet zo zitten, tenminste in die zin.. ehm.. we hadden er geen prioriteit bij, omdat we eigenlijk nog steeds in het traject zaten om het verzuim anders te regelen, laat ik het zo zeggen. Maar goed, de directeur zag er wel voordeel in en zei ‘nou, ik denk toch dat we dat gaan doen’. En toen is er ingeschreven en toen ben ik, ja, naar de bijeenkomst gestuurd zal ik maar zeggen. En ik heb het op mijn bordje gekregen om dat verder uit te zetten en ehm.. want eigenlijk moet dit hele project ook echt bij de mentoren terecht komen. Dat mentoren met name dat ziekteverzuim bij hun leerlingen zien en daar vervolgens ook stappen op ondernemen en vervolgens ook die gesprekken hebben enzovoorts. Dat zou niet alleen bij mij moeten liggen.

**Waarom vind je dat?**

Nou, kijk, als ik van 462 leerlingen het verzuim moet bijhouden, dat doe ik al. Maar als ik dan ook alle gesprekken die daar bij horen voeren, ja, dan mag ik er nog wel een weektaak bij krijgen denk ik. Ik bedoel, dat gaat niet. Dus ik ben er nu zelf mee gestart, zo van ‘nou, laat ik er eerst eens even mee beginnen, zodat ik het dan vervolgens kan overdragen aan mentoren van jongens ‘zo werkt het’’. Maar in dat stadium zitten we nu en zo ben ik er dus ook aan vast komen te zitten.

**Je zei net dat jullie al bezig zijn het verzuim op orde te krijgen, wat bedoel je daarmee?**

Nou, ehm.. de hele registratie van het verzuim liep hier nogal gebrekkig. Vorig jaar zijn we gaan werken met het systeem PARS, of anderhalf jaar geleden.. dat heeft al wat voordelen opgeleverd. Dus dat we nu veel meer zicht hebben op hoeveel leerlingen verzuimen en hoe lang en waarom ze verzuimen. En daar liep de doorkoppeling naar de leerplicht nog niet zo goed van, dat loopt nu ook. En we hebben veel striktere maatregelen op dat verzuim gezet.

**En heb je het dan over ongeoorloofd verzuim?**

Dan hebben we het over ongeoorloofd verzuim en ook over het signaleren van lang ziekteverzuim. Dat we zien van ‘hee, deze is wel erg lang ziek voor een ‘kleinigheidje’’.

**En hoe zie je dat dan?**

Nou, omdat bij de administratie de ziekmeldingen worden gedaan en daar wordt er altijd een vraag gesteld van ‘wat heeft hij, wanneer verwacht u dat hij weer terug is op school enzovoorts’. En als er dan wordt ingevuld ‘hoofdpijn of buikpijn’, maar ze zijn vervolgens een week niet op school, dan gaat er bij de administratie weer een belletje rinkelen van ‘hee, dit klopt niet’.

**Dus het is de administratie die dat in de gaten houdt?**

Ja, die dat in de gaten houdt. Ja, samen met de mentoren, want die krijgen vanuit PARS elke week keurig netjes een overzicht van ‘die leerlingen van jou zijn zo vaak ziek geweest om die en die reden’.

Dus mentoren gaan ook beter kijken. Terwijl voorheen…

**Is dat nieuw, dat ze dat nu krijgen?**

Nee, dat is nu een jaar. Dus door PARS hebben we wel gewoon door cijfers meer zicht op hoe loopt het nou met dat ziekteverzuim en daardoor al zien we dat het langdurig ziekteverzuim wel wat vermindert. Omdat we er bovenop zitten. Maar..

**Wat is ‘er bovenop zitten’?**

Nou, dus dat we op het moment dat ik of de mentor van de administratie zo’n melding krijgt van ‘deze leerling is nu al 6 dagen ziek, maar de oorzaak was hoofdpijn’, dan hebben we zoiets van ‘daar gaan we eens een telefoontje aan wagen’. En dan blijkt al vaak dat een telefoontje voor ouders al zoiets is van ´hee, ooh, hij moet maar eens naar school´. Wat dat betreft zijn er toch best veel ouders die hun kinderen wel erg gemakkelijk thuis houden. Tenminste, ik vind van wel.

Kijk, als je dat verhaal van net, die jongen die zich bedreigd voelt op school… Nouja, goed, er is wel wat voorgevallen, maar om hem dan in afwachting van aangifte bij de politie gewoon een week thuis te houden, heb ik zoiets van ´ja, volgens mij maak je het alleen maar erger´. De stap om naar school te gaan…

**Wat is het nadeel dat ouders steeds makkelijker met dat ziekmelden omgaan? Welke last heeft de school daarvan?**

Nou, de last die de school ervan heeft is dat we met lesprogramma’s natuurlijk voor zo’n leerling achterkomen en dat het zeker bij ons soort leerlingen heel moeilijk is om ze te bewegen om op die achterstand in te lopen. Hè, dus, als er toetsen of overhoringen worden gedaan, die moeten altijd – en zeker in het 3^e^ en 4^e^ jaar, omdat dat altijd examenwerk is – worden ingehaald. Nou, en als een leerling ziek is en hij mist 3 of 4 toetsen, ja, een VMBO-leerling heeft daar gewoon grote moeite mee om te plannen van ‘oh, dan moet ik naar die leerkracht, naar die leerkracht en die leerkracht en dan moet ik eens gaan kijken wanneer ik die toets kan inhalen’. Onze leerlingen hebben dat uit zichzelf niet zo. Zo van ‘nou ja, als er niets van gezegd wordt, dan eh…’. Dus daar moeten we achteraan. Dus heeft ziek-zijn en afwezig zijn voor de leerling, maar ook voor de school, altijd een lange nasleep. En als leerlingen dus regelmatig ziek zijn, dan gaat dat steeds langer na-ijlen, dat is heel lastig. We hebben nu een paar leerlingen met echt exorbitant ziekteverzuim en eentje waar ik nu mee bezig ben, die heeft vorig jaar en dit jaar al zoveel verzuimd, dat dit jaar gewoon puur op basis van prestaties nú al mislukt is. Die gaat het nu al niet meer halen, kan het niet meer ingehaald krijgen.

Ik bedoel, als jij in de eerste 3 maanden van het schooljaar anderhalve maand ziek bent geweest en al die toetsen nog moet inhalen, dat krijgen ze nooit voor elkaar. Dat krijgen ze echt niet voor elkaar.

**Bij de aanpak van ziekteverzuim, kun je met de ouders bellen, zie je daar ook een rol weggelegd voor de jeugdgezondheidszorg?**

Ja, zeker als je.. ehm.. als het ziekteverzuim niet ‘echt aanwijsbaar een puur lichamelijke oorzaak heeft’. Ik bedoel, klachten als hoofdpijn, buikpijn, noem maar op, die elke keer weer opnieuw optreden, wijzen vaak op iets anders dan puur een lichamelijke klacht. En als dan, bijvoorbeeld de schoolarts daar ook eens naar zou kijken. Hè, *want van een huisarts worden we meestal niet wijzer, als we er al informatie van krijgen. S_7c_ En via de ouders is de informatie toch al vaak heel erg gekleurd en niet erg betrouwbaar. S_7b_* Dan kun je de schoolarts inschakelen en zeggen ‘nou, kijk jij er eens naar en geef mij eens een advies’. Dat zie ik wel, alleen de weg daar naartoe is vaak nog wel, voor ons moeizaam te nemen.

**Wat bedoel je daarmee?**

Nou, puur het werk wat eraan zit. Signaleren, vervolgens doorzetten, het gesprek dat blijft… dat moet je echt heel structureel kunnen inplannen. Op dit moment krijg je dat de mentoren, of ze het zijn weet ik niet maar, laat ik dat even in het midden houden, ze voelen zich in elk geval toch al steeds meer overbelast. We moeten dit doen, zus doen, zo doen. Dat als ik nu kom van ‘jongens, dit gaan we ook nog doen’. Vandaar dat ik er dus nu eerst zelf mee aan de slag ben, zo van ‘nou, zo werkt het’, zodat ik het als een kant-en-klaar pakketje kan neerleggen.

**Want je bent er wel mee begonnen nu?**

Ja, ik ben er nu wel mee begonnen.

**Hoe gaat dat, hoe bevalt dat?**

Nou, dat weet ik nog niet, want ik moet voor die jongen die ik net noemde, ga ik naar M@ZL de melding doen, want daar heb ik namelijk al een beeld bij wat daarmee zou moeten gebeuren. Dat is een jongen met waarschijnlijk, met mijn deskundigheid van de koude grond overigens, zich ontwikkelende psychose. Die jongen is.. en.. maar.. Ouders willen, ja, willen daar wel aan, maar die willen niet richting een behandeling. Maar nu heb ik via kanalen gehoord dat C., de jeugdarts, daar dan echt iets in kan betekenen.

**Zouden ze naar de jeugdarts willen gaan?**

Ja.

**Hoe breng je dat?**

Nou, dat heb ik ze al verteld in het gesprek wat ik met ouders heb gehad. Eh…

**Wat is de meerwaarde van de jeugdarts dan voor jou, hoe breng je dat naar ouders?**

Ehm… Die leerling die hebben we ook al in het ZAT besproken en ik heb met moeder ook al besproken dat wij de stap naar de schoolarts ook maken en moeder die wil best alle hulp die ze krijgen kan. Absoluut. Die zit thuis ook al…

Maar wat dus wel nu nog aan de hand is, dat ik dus nu alleen echt met de ernstigste gevallen begonnen ben. Ik bedoel, eh.. Terwijl het zo zou moeten zijn dat het echt gewoon al een vroegtijdige signalering is van ‘hee, wacht even, deze leerling is 6 keer of 6 dagen ziek, moet ik daar niet een klein telefoontje aan wagen, moeten we daar niet even een gesprekje over hebben, hoe komt dat’. En dan zijn we er eerder bij denk ik. Maar..

**Want, wat levert het op als je er eerder bij bent?**

Nou, dat ze waarschijnlijk een volgende keer hun kind weer eerder naar school toe sturen. Zo van ‘6 dagen ziek is toch wel een beetje veel, zou je niet al wat eerder moeten’.

**Wat is voor jou nu omvangrijk? Wat vind je omvangrijk?**

Omvangrijke ziekte?

**Ja, wanneer zeg je nu ‘ik ga eens een gesprek voeren’?**

Nou, meestal komt het signaal binnen als er echt al langer als een dag of 10 een ziekmelding is. Dus eh.. en dan gaan er bellen rinkelen, zo van ‘jeetje’. Grappig is dan wel dat die bellen weliswaar via de administratie rinkelen, maar dan ook via de docenten beginnen te rinkelen, zo van ‘hee, die heb ik nu al drie lessen niet gezien, hoe zit dat?’. Dan begint er wat leven in de brouwerij te komen wat dat betreft.

**Dat is dus langdurig verzuim. Gaan er ook wel eens bellen rinkelen als je zegt ‘die is nu voor de zoveelste keer ziek’.**

Ja, toch wel.

**Wat zie je dan? Wanneer is dat dan?**

Nou, dat zie je ook wanneer er met name leerlingen in bepaalde lessen steeds uitvallen. Bijvoorbeeld soms ook wel eens in lessen van dingen die ze moeilijk vinden, bijvoorbeeld. Of een docent waar ze het niet zo goed mee kunnen vinden. Dan zijn ze bijvoorbeeld elke keer op de woensdag ziek. Ik bedoel, ik heb ook wel eens een leerling gehad die zich door mij heel erg gecontroleerd voelde – ik geef ook les, niet zo veel, nu maatschappijleer, maar ik gaf mens&maatschappij (aardrijkskunde, geschiedenis en economie – en die jongen die voelde zich erg gecontroleerd door mij en was er dus steevast op woensdag niet. Omdat hij bij mij in de les zat. En dat valt je dan op.

*En ook leerlingen die bijvoorbeeld stelselmatig op maandagochtend ziek zijn hè, daar hebben we ook veel last van hier. Leerlingen die dus gewoon in het weekend, in het weekend zo de bloemetjes buiten zetten dat ze echt te gammel zijn om naar school te komen. S_7a_* Ja, daar ben ik.. Ik heb voorheen in Roosendaal op een VMBO gewerkt en daar was dat niet zo erg als hier.

**Plattelandsjongeren?**

Ja, niet alleen maar, maar 60 a 70% zijn plattelandsjongeren.

**Kun je iets vertellen over de leerlingen die hier zitten? Wat zijn het voor leerlingen?**

Heel veel leerlingen uit de dorpen hier rondom Halderberge. 30% van de leerlingen komt ongeveer uit Roosendaal, dat is dus gewoon ‘Roosendaalse stadsbevolking’ zal ik maar zeggen. En de rest komt allemaal uit de dorpen en zij hebben wel een link met ‘groen’, want we zijn natuurlijk een landbouw-VMBO, maar ze kiezen ook heel veel voor deze school, omdat het een kleine school is.
De Roosendaalse jongeren ook omdat het, ik schaam me bijna om het te moeten zeggen, maar ook omdat het ‘een witte school is’.

**Daar hoef je je niet voor te schamen..**

Nee, nee, ik hoef me daar niet voor te schamen. Ik bedoel, dat andere mensen daar niet voor kiezen, maar het is wel opvallend.

**Is agrarisch onderwijs zo?**

Nee, door allochtonen wordt dat niet gekozen. Daar is ook wel een verklaring voor, volgens mij dan. Kijk, in Marokko of in Turkije, als je boer bent sta je daar helemaal onderaan de maatschappelijke ladder.

**Ze komen niet naar Nederland om hier weer boer te worden?**

Nee, ze komen niet naar Nederland om hier weer boer te worden. En de enkeling die hier iets beter naars school kijkt, ziet dat hier natuurlijk echt heel veel leuke dingen te doen zijn. Dus we hebben wel, we hebben op dit moment 2 Turkse meisjes hier op school en dan hebben we nog eentje uit Colombia…

**U kunt ze op een hand tellen?**

Ja, ik denk dat wij op 462 leerlingen, dat we maximaal 10 hebben waar ik een enigszins allochtoon plaatje op kan plakken. Dat is echt heel weinig. En dan vind ik wel eens jammer, want het vernauwt de blik van onze leerlingen wel heel erg. Dus ehm.. De meningen die er over allochtonen heersen hier op school, dat zijn natuurlijk hele platvloerse en hele ondoordachte meningen waar de leerlingen in principe zelf ook wat aan kunnen doen, maar goed, ze hebben zo’n beperkte blik. Je wil het soms niet horen hoor, wat er hier gezegd wordt. En dat komt alleen maar omdat ze er nooit mee in aanraking komen.

**Er zijn dus leerlingen die op maandagochtend steevast in bed blijven liggen, wat gebeurt daarmee? Zijn er dan ook gesprekken?**

Nee, diegenen van wie we dat echt hebben gesignaleerd, daar zijn wel gesprekken met de leerlingen geweest. Maar ja, dat wordt natuurlijk altijd door ouders ook ontkend hè. Ik bedoel, wat bij ziek zijn sowieso wel een rol speelt is dat als er iets aan de hand is, er een lange tijd van ontkenning is door de ouders. Als er een probleem op school speelt, dan zeggen ouders daar heel lang niets over en op een bepaald moment komt het eruit. Op dit moment hebben we een leerling die langdurig ziek thuis zit vanwege last van hypermobiliteit. Daar zijn de ouders pas op een heel laat moment mee naar buiten gekomen en vervolgens zeggen ze van ‘ja, de school is er heel slecht mee omgegaan’. Want ‘wij hadden het niet op tijd onderkend’. Zij hebben dat zelf niet op tijd aangegeven of misschien wel aangegeven, maar niet de ernst ervan aangegeven. Vervolgens houden ze zo’n jongen thuis en dan moeten wij erachter komen, nadat we die jongen eens gevraagd hebben van ‘hee, hoe zit het nou’. Dan komt er in een keer alles uit en dan blijkt er naast het lichamelijk probleem, plotseling ook een psychisch probleem te spelen. Vervolgens is de school dan wel de schuldige. ‘Want het is op school gebeurd enzovoorts’. En als er een, kijk, als jongeren zich thuis, met name in het weekend flink te buiten gaan aan bijvoorbeeld drank ofzo, ja, dan zul je de ouders daar niet over horen. Die gaan dat niet vertellen.

**Zijn er nu ook bepaalde gezinnen of ouders die bij die leerlingen horen?**

Nou, ja, ik weet niet of het nou zo direct milieubepaald is. Wat wel is, natuurlijk ook in de dorpen heb je groepen jongeren die in het weekend natuurlijk met elkaar optrekken in de vrije tijd en met name de ‘schuurfeesten’ en de ‘zuipketen’ enzovoorts, die spelen daarbij wel een rol. Ik bedoel, de KPJ is natuurlijk een prachtige organisatie, maar het is voor veel jongeren ook dé plek / omgeving waarin je uitgaat. En ja, ik heb wel het idee dat er veel meer jongeren dan ik goed vind, in het weekend drank gebruiken. Excessief.

**En wordt daar op school in het algemeen wat aan gedaan?**

Wij hebben op school onze projecten en we hebben een vast item op de ouderavonden dat we hier Novadic / Kentron uitnodigen, dat we het CJG uitnodigen, om dat soort dingen bespreekbaar te maken. Dat doen we elk jaar weer. Maar dat gebeurt ehm.. Ik denk dat hier, nou goed, ik denk dat het in het kader van dit gesprek past… Ik denk dat hier zeker 50% van onze leerlingen te vroeg drank gebruikt.

**En dat hangt samen met ziekteverzuim?**

Ehm… niet altijd. Niet generaliserend dat dat ermee samen hangt. Maar er zijn wel kinderen die daar op maandagmorgen last van hebben. En dus niet op school zijn. En leerprestaties, ja, ik weet niet of ze eronder lijden. Ik heb er geen bewijzen voor, maar als ik de deskundigen moet geloven heeft dat ook zijn gevolgen.

**Hoe ziet u de taken en verantwoordelijkheden van de school met betrekking tot ziekteverzuim van leerlingen?**

Ja, wij moeten het natuurlijk zoveel mogelijk proberen te voorkomen door goed voor te lichten en er bovenop te zitten als leerlingen er niet zijn. Maar ik vind ook dat er wel een flink stuk van de verantwoordelijkheid voor het ziekteverzuim bij ouders ligt. Maar wij kunnen natuurlijk niet veel meer doen dan het signaleren, registreren en als we iets gesignaleerd hebben, daar melding van maken bij ouders, bij leerplicht en eventueel nog andere ondersteunende instanties. En daar waar nodig natuurlijk gesprekken hebben met de ouders en leerlingen om te zorgen dat ze inderdaad weer vaker die school bezoeken of niet zo vaak verzuimen. Maar ja, daar houdt het zo ongeveer op. Ik bedoel, ja, je hebt ook niet zoveel mogelijkheden meer dan ouders te spreken en ouders te waarschuwen voor gevolgen enzovoorts. Wat we wel merken is dat met name gesprekken bij bijvoorbeeld leerplicht, die we dan wel proberen te organiseren.. Want als wij zien dat een leerling heel vaak ziek is, hebben we soms ook wel overleg met leerplicht, zo van ‘nou, zou je die nou eens niet oproepen’. Dat vervolgens die gesprekken niet zoveel uithalen. Leerlingen zijn daar niet zo van onder de indruk.

**Hoe komt dat? Niet onder de indruk? Ziet u wel een taak of rol voor de leerplichtambtenaar bij ziekteverzuim?**

Jawel, absoluut. En die taak of rol die nemen ze ook. Ik bedoel, in een paar voorkomende gevallen die ik de laatste tijd heb gehad heb ik bijvoorbeeld een hele goede samenwerking met de leerplicht Roosendaal, maar je ziet daar ook wel heel veel ‘onmogelijkheden’. Als ik zie, bijvoorbeeld, ik heb hier een jongen die we nou uiteindelijk op de ‘reboundvoorziening’ hebben geplaatst in afwachting van een vervolg. Die is alles bij elkaar, vorig jaar 50% van de tijd niet geweest en daar hebben we dus constant contact over gehad met leerplicht. En hij verzuimde niet alleen veel, maar hij werd ook gewoon echt ziek gemeld, als zijnde ‘ziek’. ‘Ja, hij heeft nu weer last van eczeem en hij voelt zich nu niet lekker of hij kan zijn bed niet uit van de …’. Nou ja, noem het maar op, alle redenen zijn door moeder al aangedragen. En die heeft zelfs van de rechter een taakstraf gekregen en nóg zit leerplicht in samenwerking met jeugdreclassering met handen gebonden voor die jongen, we krijgen hem nog niet naar school.

**Is die jongen dan ook door een arts gezien? De huisarts?**

Daar zit begeleiding in de thuissituatie, daar is begeleiding via de leerplichtambtenaar, die zit er bovenop, de jeugdreclassering heeft daar een rol in en door een arts is hij door een huisarts gezien, maar.. En ja, die jongen die ‘hebben we opgegeven’. Tenminste, hij gaat nu naar de rebound en nu proberen we eindelijk of we hem niet uit huis geplaatst kunnen krijgen. Zo ver gaat het dan!

Want als je vraagt, ‘hoe ver gaat dat dan’, dat de school zich uiteindelijk nog probeert te bemoeien dat die jongen thuis niet op zijn plek is. Dan zeg ik wel eens ‘ja, ik ga wel heel erg ver hè?’.

**Wat zou de meerwaarde zijn van een arts in dat hele traject rondom ziekteverzuim?**

Nou, een arts als derde objectieve persoon, zou wel kunnen werken, maar ouders voelen het, als ze door de schoolarts / jeugdarts worden opgeroepen, vaak wel als een bedreiging. Ze komen dan vaak ook niet opdagen.

**En hoe komt dat?**

Ik denk omdat ze zich gecontroleerd voelen en ik denk omdat ze ‘ter verantwoording’ geroepen worden.

**En hoe zou dat komen?**

Ja, omdat het al vaak via, dat ze ook al eens met leerplicht in aanraking zijn gekomen en dan volgt er vervolgens nog een oproep van de schoolarts. Dus het zit vaak al in het ‘beschuldigende’. En niet in het ‘helpende’.

**En hoe zou dat anders kunnen?**

Ja, dat weet ik niet. Hoe anders.. ik bedoel, trajecten lopen zoals ze lopen. En je begint eerst altijd met melden bij leerplicht en als ze dan vervolgens ook nog denken van ‘nou, wat is er nog meer mogelijk’, dan komt de schoolarts in beeld. Maar die wordt dus ook door school en door leerplicht ‘gebruikt’ als middel.

**Als controleur?**

Ja, als controleur.

**Nu proberen we bij M@ZL-smal die weg om te draaien, dat is eigenlijk M@ZL. Dat je zegt ‘we willen eigenlijk voordat leerplicht zich erover buigt, dat er door school en zo nodig door de jeugdarts gesprekken zijn gevoerd vanuit de zorg en niet vanuit de controle’. Zou dat een oplossing zijn?**

Dat is wel zo, maar ik denk dat ouders het heel vaak als ‘bemoeizucht’ zullen ervaren. ‘Ik weet wel wat er goed is voor mijn kind’, dat horen wij hier natuurlijk talloze malen. Dat ouders ook over ons werk hier heel erg precies weten wat wij zouden moeten doen om hun kind te helpen. En misschien is dat wel een teken van deze tijd, maar dat is..

Ouders delen hun zorgen niet graag. En dat heeft ook wel een klein beetje met wat ik daarnet vertelde te maken. Als er iets aan de hand is, gaan ze in eerste instantie niet een ‘bondgenootschap’ aan met de school of met een derde. Nee, ze gaan ertegenover staan. Ook als het met een leerling niet goed gaat in prestaties bijvoorbeeld, dan is het niet vaak zo dat ouders zeggen van ‘ja, goh, hoe komt dat nou en wat kunnen wij doen, wat kan de leerling doen?’. Nee, er ligt een slecht rapport op tafel, ‘wat gaat de school eraan doen’. Dan denk ik van ‘ja, ho, stop, wat gaat school eraan doen?’, volgens mij gaan we er op zijn minst samen wat aan doen!

**Is dat een verandering in de tijd ofzo?**

Ja, ik vind het wel een tijdsbeeld. Ik zit nu 35 jaar in het onderwijs en ik heb dat wel zien veranderen ja. Dat ouders, ook bij conflicten met de school, die gaan meteen achter hun kind staan, tegenover de school. En tegenover instanties en tegenover…

En er wordt niet, er wordt steeds minder gekeken van ‘hee, we constateren een probleem bij mijn zoon of dochter, hoe kunnen we dat nou oplossen?’. Of, en dat zou ook al een oplossing zijn, van ‘school, lossen jullie dat voor mij eens op’, maar heel vaak gaan ze in de aanval. Ze gaan tegenover je staan. En dat vind ik wel steeds moeilijker worden.

**En de jeugdarts lijkt dan een verlengde van de school en dus tegenover ouders te staan?**

Ja, dat denk ik wel. Wordt niet zo, kijk.. Vroeger, ik als klein jongetje moest ook naar de schoolarts en dan kwam mijn moeder mee en dan werd daar, dat weet ik nog goed, gewoon gesproken over mijn gezondheid en of alles bij mij in orde was. Zo van ‘oké, nou, wat mooi!’. Die sfeer proef ik tegenwoordig niet meer. Dat er ook iets werd aangenomen van een deskundige. ‘Nee, ik weet wat het beste is voor het kind. Ik moet hier nu wel komen, maar wat ga jij mij nou vertellen wat ik nog niet weet’. Ik chargeer nu hoor, maar dat gevoel proef ik steeds minder. Ouders weten heel erg goed hoe het moet, wat er goed is voor hun kind.

**Hoe komt dat, die verandering?**

Dat weet ik niet, geen idee. Ik bedoel, ik heb zelf kinderen en ik heb die houding zelf niet. Maar ja, goed, dat komt omdat ik misschien zelf in het onderwijs zit.

Ouders weten ook al altijd exact wat er hier op school gebeurd is. Vandaag nog aan de hand gehad, een ouder die opbelt over een klein voorvalletje: een leerling die een docent uitgescholden heeft. En die docent heeft dat gehoord en de leerling zegt ‘ik heb dat niet gezegd’. En ouders zeggen dan ‘ze zegt dat ze het niet gezegd heeft, dus het is niet gebeurd. En dus vind ik niet dat ze er straf voor moet krijgen. Ik bepaal dat mijn dochter geen straf krijgt.’. Ja, dan denk ik, waar ben je, waar sta je dan nog. Ik bedoel.. En dan bepalen de ouders dus dat ze geen straf krijgt.

**Bepalen de ouders dat kinderen ziek gemeld thuis blijven of de kinderen?**

En daar zit een hele goede. Ik denk, en dat is wel een klein beetje een tijdsbeeld, dat tegenwoordig de kinderen thuis de boel besturen.

**Die krijgen de macht daardoor hè?**

Ouders zijn, lang niet allemaal hoor, maar er zijn er wel veel, er zijn heel veel, en dat is ook weer gechargeerd, maar er zijn heel veel ouders die gewoon de lakeien van hun kinderen zijn. Echt waar. Want ze willen maar aardig gevonden worden. ‘Ik moet vooral een goede band hebben met mijn kinderen’. Ja, dat kun je ook hebben door gewoon streng te zijn of duidelijk te zijn.

**Dus al die verhalen over ‘ouders moeten weer eens op gaan voeden’, dat klopt.**

Ja. Kijk, daar is in sommige gevallen echt wel wat van waar ja. Dat denk ik wel.

**U bent nu bezig met het invoeren van M@ZL, u gaat het inbrengen bij de mentoren. Hoe ziet u dat voor zich? Denkt u dat het gaat werken?**

Ja, het moet gaan werken. Gewoon omdat we het invoeringsplan volgen en het gewoon als taak wegleggen. ‘Dat hoort er gewoon bij’. Dus dat moet gewoon, het moet inslijpen, maar dat moet gewoon onderdeel worden van het dagelijks en wekelijks handelen. Zo van, ‘nou, even kijken welke leerlingen eh..’.

**Staat u daar achter?**

Ja, ik wel. Ik vind dit een… Kijk, de hele invoering dat zal nog een flinke dobber worden, daar gaat het niet om. En dan niet alleen omdat we de mentoren misschien niet zo ver krijgen, maar ook gewoon omdat het mezelf vaak aan tijd ontbreekt. Maar als er gewoon een stramien is, een helder stramien, waarmee je verzuim behandelt, waarmee je het verzuim aanpakt. Als iedereen dat gewoon in zijn vingers heeft, denk ik dat het op termijn weinig werk hoeft te zijn. Ik bedoel, als je ervan uit gaat dat het al wel weer ‘heel veel werk’ zal zijn, dan moet je er niet aan beginnen. Kijk, het is nu heel veel werk, het invoeren is veel werk, maar uiteindelijk moet het werk besparen.

**Ja, zeker.**

Want als ik achter alle kinderen aan moet blijven zitten zoals ik nu doe, dan kost mij dat gewoon veel te veel tijd. Zoals ik nu, zonder M@ZL, achter de kinderen aanzit hè.

**Dus u hoopt dan te voorkomen dat u daar achteraan moet zitten. Zijn er nog andere effecten die u verwacht van M@ZL?**

Ja, kijk, ik zou heel graag zien, maar dat is natuurlijk een heel prachtig doel... Ik zou heel graag zien, maar dat is op heel veel meer vlakken, dat ouders ons veel meer, ten aanzien van ziekte, als compagnon zouden zien. Dan als zijnde ‘oh, de school’.

**M@ZL staat voor medische advisering ziekgemelde leerling, door de JGZ, we hebben daar 2 varianten van. (Uitleg over smal en compleet, criteria en het ondernemen van actie.) U bent daarmee bekend. Als u nou vrij mocht kiezen voor de aanpak van ziekteverzuim, zou u dan überhaupt kiezen voor een samenwerking met de JGZ en zo ja, wat zou u kiezen?**

Nou, ik zou sowieso wel kiezen voor samenwerking, dat wel. En ik zou, maar dat is altijd een financiële kwestie, ik zou voor compleet gekozen hebben. Als ik de keuze had mogen maken, had ik meteen voor compleet gekozen.

**Waarom?**

Puur vanwege het werk. Omdat..

**Oké. Stel dat u gewoon een mannetje geleverd krijgt, die al die gesprekken gaat doen, en dan?**

Nou, dan, ten eerste denk ik dat diegene die de gesprekken dan voert, daar deskundiger in is. Hè, want kijk, ik kan natuurlijk hartstikke goed gesprekken voeren met ouders, maar als het gaat om gezondheid, om het zo maar te noemen, dan ben ik natuurlijk een leek. Vervolgens lijkt mij de directe link met of naar een deskundigheid veel beter dan dat wij daar eerst nog een tussenstap in moeten maken. Dat vind ik gewoon… En ik denk dat het mij ook wel werk zal.. Want dan krijg ik namelijk, tenminste, neem ik aan, gewoon een terugmelding van ‘nou, deze leerling is gesproken, dat en dat is er aan de hand’. Daar kan ik dan waarschijnlijk mee aan de slag. Dan weet ik wat ik moet doen. Terwijl, nu moet ik eerst zelf een gesprek aangaan, zelf gaan bedenken wat ik moet doen en dan is er weliswaar al een doormelding naar de jeugdarts, maar ik moet toch aardig wat meer in verzetten. Maar dat is puur..

**En u zegt zelf, het is niet alleen werk, maar ook de deskundigheid.**

Ja, ook de deskundigheid. Kijk, om maar terug te pakken op die jongen met zijn hypermobiliteit. Ja, ik heb het verhaal van die ouders wel aangehoord over hypermobiliteit en wat daar de consequenties van zijn. Dan moet ik aannemen van de ouders dat wat zij zeggen, dat het klopt. Ik kan dat in twijfel trekken, maar dan moet ik toch nog ergens gaan navragen van ‘hee, heeft die hypermobiliteit voor ons onderwijs aan hem zoveel gevolgen?’. Want ouders zeggen nu heel netjes, ‘moet je luisteren, door zijn hypermobiliteit kan hij bij jullie de opleiding niet meer volgen, want hij heeft veel te veel pijn als hij in de tuin moet werken’. Dat moet ik maar aannemen. Als een jeugdarts daarnaar gekeken heeft, meteen naar gekeken heeft, kan die A. misschien adviseren van wat kan hij nou wel en B. het is misschien helemaal niet zo, want hij kan best een heleboel, alleen moet hij misschien wat lichtere werkzaamheden doen. Óf ‘nee, het is beter dat hij naar een andere school gaat’. Dat soort vragen moet ik nou eerst allemaal zelf beantwoorden. Wat ik dan ook gedaan heb, maar het is wel lastig.

**Ja, op dat punt ziet u duidelijk de meerwaarde van een jeugdarts. Zouden de ouders dat ook zien? Hoe gaat u dat communiceren met ouders?**

Of de ouders dat ook zien? Bij duidelijke ziektebeelden denk ik wel dat ze het zien, van ‘nou, nog een extra hulp, nog een dokter die iets zegt’, dan wel. Bij de wat vagere klachten en ook klachten waarbij gedragsproblematiek of schoolproblemen een rol spelen, denk ik dat ouders nog wel wat gereserveerder zullen zijn ten aanzien van de jeugdarts en de adviezen daarvan.

**Jammer.**

Maar dan zit je ook weer in het plaatje van ‘het gaat al niet goed op school en ze zijn ziek’ en dan zit je toch in de aanval hè. In het tegenover elkaar staan.

**Wat jammer hè.**

Ja.

**Ik stel dan ‘het is toch een extra kans om een stukje ondersteuning te krijgen’.**

Ja, ik begrijp die instelling ook niet helemaal. Ik bedoel, ik heb zelf ook 2 kinderen.

**Stel dat uw dochter nu voor de 4^e^ keer ziek moest melden en u kreeg een bericht dat er een gesprek met de jeugdarts was. Hoe zou u dat vinden?**

Daar zou ik, persoonlijk, geen probleem mee hebben. Maar ja, goed, ik sta ook, behoudens een enkele docent die ik dan ken, ik sta ook niet tegenover de school. Ik bedoel, als er met mijn dochter op een bepaald terrein problemen waren, stapte ik naar de mentor van ‘goh, we hebben dit gesignaleerd, hoe kunnen we het oplossen?’ en die meneer heeft ons daar ook in geholpen. Mijn dochter wilde dat dan wel niet, maar goed, dat ligt aan mijn dochter, dat ligt niet aan mij (lacht).

Kijk, want het is natuurlijk wel zo, maar daar zit ook een klein beetje van dat bestuur in. Kijk, kinderen staan natuurlijk traditioneel gezien al tegenover de school. Ik bedoel, eh.. Kijk, alle kinderen gaan heel graag naar school, maar ze zullen het nooit zeggen. Ik bedoel ‘School, nee, daar ben je tegen’. ‘Je moet zoveel mogelijk vrij zijn’, totdat ze niet meer naar school mogen. Dan blijkt dat ze het toch eigenlijk wel heel erg leuk vinden op die school, want zo werkt het hè. Dus kinderen staan in principe gevoelsmatig tegenover de school, maar ouders zouden dat niet moeten zijn. Maar ze worden er door hun kinderen, denk ik, wel eens ingetrokken. ‘Want ik kan niet zo goed met die docent opschieten, die man mag mij niet’. Ja, oké. ‘Die mag jou niet’, ik zeg dan ‘moet je daar dan niet een modus in vinden dat het in ieder geval werkbaar is’. Maar zo denken heel veel ouders niet. Nee, ‘die man ziet alles van mijn dochter, ze krijgt geen kans, enzovoorts, enzovoorts’.

Ja, en dat vind ik wel lastig.

**Als de school voor M@ZL compleet kiest, dan zit daar natuurlijk ook een kostenplaatje aan.**

Ja, dat is een van de redenen waarom de school, in dit geval de directeur, gekozen heeft voor M@ZL smal. Want dat kostenplaatje is wel een.., kijk dat komt nog wel een keer terug en daar kun je dan op een gegeven moment ook niet meer zonder. Kijk, een school zal natuurlijk altijd proberen om de vaste kosten, zal ik maar zeggen, zo laag mogelijk te houden. En als M@ZL een terugkerende vaste kostenpost is, ja, dan kan ik me voorstellen dat dat budgettair natuurlijk problematisch is. En ja, wat scholen natuurlijk heel vaak denken, misschien wel te vaak denken, ‘dat kunnen we zelf wel of daar hebben we zelf wel mensen voor in huis’. Vergetende dat die mensen meestal ook al druk zijn.

Ja, dat is heel, ik weet niet of dat ergens anders ook zo is, ik heb nooit ergens anders gewerkt als in het onderwijs, ik zit mijn hele leven al op school. Taakbelasting is namelijk een hele lastige op school. Ik bedoel, omschrijf eens wat je doet en kun je zo’n taak er nog wel bij hebben. Het wordt bij iemand neergelegd, die zoiets moet gaan doen en dat is goedkoper dan werk inkopen.

**Dat lijkt het.**

Ja, dat lijkt het. Op termijn is het misschien wel niet zo. Maar bij ons wordt natuurlijk niet alles in uren uitgerekend. Er wordt ergens een taak neergelegd en daar merk je dan verder niets meer van.

**Kost ook niets..**

Het kost zo gezegd niets.

**Vind u dat de school begeleiding door de jeugdarts voor ziekteverzuim bij leerlingen moet betalen? Of zijn er andere partijen die dat zouden kunnen doen?**

Nou, eigenlijk vind ik niet dat de school het zou moeten betalen.

**Wie dan wel?**

Ja, ik vind dat eigenlijk een… Kijk, als het schoolverzuim bijvoorbeeld bijhouden en regelen, als dat een gemeentelijke taak is, zou ik dit ook een gemeentelijke taak willen noemen. De maatschappij in zijn breedte, vindt dat schoolverzuim zo minimaal mogelijk moet zijn, dus moet de maatschappij, in dit geval de gemeente dus of een andere maatschappelijke dienstverlening moet daarvoor opdraaien. En niet een school met zijn beperkte middelen. School verzorgt onderwijs en school heeft, kort door de bocht, weinig te maken met de gezondheid van kinderen.

**Klopt dat?**

Nee, ik zeg ‘kort door de bocht’. Natuurlijk hebben wij wel een belangrijke taak in de gezondheid van kinderen, want wij voeden ze mee op en we moeten zorgen dat ze een gezonde leefwijze hebben. Dus we hebben daar absoluut een taak in. Maar even zwart-wit gesteld, ‘wij zijn geen ziekenhuis’. Ik bedoel, ik mag hier, als er een kind iets overkomt, mag ik dat ook alleen maar melden en zorgen dat die zo gauw mogelijk in het ziekenhuis terecht komt. Dan mag ik ook niets doen, want dan heb ik daar ook geen taak in.

**Maar kijk naar ‘zorgen in en om de school’, er komt eigenlijk steeds meer richting school. Stel dat u nu als school een zak met geld krijgt om de zorg in en om de school te kopen. Zou u dan ook bedenken dat u ziekteverzuimbegeleiding daarvan moest betalen?**

Ja, als de school meer gedefinieerd wordt ook als een zorginstelling. En nu zijn wij school, maar we krijgen steeds meer taken binnen school. Ehm..

Ik vind dat je dat eens of inderdaad met meer geld moet afkomen en zeggen van ‘oké, je krijgt er een maatschappelijke taak bij en daar hoort dan dit geld bij’, maar dat gebeurt nu nooit. Ik bedoel, hoe vaak lees je niet in de krant van ‘op school moeten ze dit, op school moeten ze dat, het onderwijs kan dit ook nog wel eens doen’. Maar er komt nooit geld bij. Wij moeten bijvoorbeeld voorlichting geven over alcohol en drugs, daar moeten we lessen aan besteden. Dat gaat van onze ‘pure’ lestijd af, in die les zou je er natuurlijk aandacht aan kunnen besteden, maar daar gaat het even niet over. Wij worden geacht daar tijd aan te besteden, maar die tijd die we daaraan moeten besteden, wordt niet betaald, door niemand.

(zorgcoördinator komt binnen)

Dus dan moet er geld mee komen. Aan de andere kant zeg ik van ‘ja, moet je luisteren, als wij op het gebied van zorg meer moeten doen, moet er geld komen, maar ik zou nu zeggen: nee, pak er maar lekker een aparte organisatie voor’. Dat zou ik prefereren. Ik zou het dus buiten de school zien.

**Want school heeft wel een taak voor het signaleren, denk ik.**

Ja, signaleren wel, natuurlijk. Ik bedoel, wij zijn gehouden om aan onze ingeschreven leerlingen onderwijs aan te bieden. Als we dat onderwijs niet meer aan kunnen bieden, dan voldoen we niet aan onze taak. En dan moeten we dat signaleren. Dat is heel strikt genomen. De trend die ons staat dat wij steeds meer sociaal werker worden, om het zo maar te zeggen, ja, die zou ik wel gekeerd willen zien. Ik kan je nog een heel mooi staaltje vertellen. Op vrijdag ben ik vrij en afgelopen vrijdag loop ik in de Albert Heijn te winkelen, komt er een moeder langs van een leerling. Ik zeg ‘goedendag’, dus zij zegt ‘hallo, goedendag’. Ik loop door, vijf minuten later sta ik te kijken welk pak koffie ik moet kopen, krijg ik opeens een telefoontje van die mevrouw in mijn handen gedrukt. ‘Ja, er zijn problemen op school en mijn dochter die wordt buiten school bedreigd’, dat ging ook weer over een vechtpartijtje, ‘wilt u het even oplossen?’. En dan sta ik daar en denk ik ‘ja, waar trekken we eigenlijk de grens’. Dit is een waargebeurd verhaal, dat is echt. Dan denk ik ‘waar trek je de grens, wanneer is het nou voor de school en wanneer is het voor iemand anders’. De onderwijsinstituten van 30 à 40 jaar geleden, zijn we al lang niet meer, maar soms verlang ik daar wel eens naar terug.

**We hebben ontzettend veel besproken, zijn er nog dingen die u ons wil meegeven? Dingen over ziekteverzuim, de begeleiding, de samenwerking met JGZ, de rol van de school of gemeente?**

Nou, ik zou wel willen dat het rondom het verzuim en rondom met name ziekteverzuim, er duidelijkere en betere stappen, ook helderdere stappen voor ouders en leerlingen genomen werden dan nu het geval is. Want nu opereren wij altijd in een heel grijs gebied van ‘wie doet wat en waarom en bij wie moet ik terecht’. Met name ook met leerlingen waarbij de problematiek én gezondheid én geestelijke gezondheid is. Dat wij contacten hebben met ‘jan en alleman’, het CJG, JPP, noem ze maar op. En dat het uiteindelijk op ons bordje blijft liggen en er heel vaak toch nog weinig gebeurt. En dat we dan dus..

**En M@ZL smal is daar niet de oplossing voor?**

Nee, niet volledig. Het is een stap in de goede richting, maar het is niet de oplossing daarvoor. Ik zou veel meer pleiten voor een aan leerplicht of GGZ gelieerde organisatie die de signalen, want die moeten natuurlijk wel van de school komen, signalen oppikt, maar eigenstandig daarmee aan het werk gaat en de diverse kanalen / organisaties informeert. ‘Dit moet je met die leerling doen en dat moet je met die leerling doen’. Nu zijn wij altijd nog maar aan het ‘shoppen’.

**Dan komen we eigenlijk bij M@ZL compleet uit..**

Als ik nu kijk naar de ernstige gevallen waar ik nu mee bezig ben, dat gaat altijd om verzuim met een ‘gezondheidsrandje’ eraan. Het is vaak geen gedragsproblematiek hier op school, maar met een ‘gezondheidsrandje’ eraan. Dat het altijd hele slepende trajecten zijn, waarbij ik als coördinator hier allerlei mensen rondom de tafel moet roepen en beslissingen moet gaan nemen, terwijl wij het als school al neer hebben gelegd bij ‘die, die, bij die en die’, maar oplossingen komen er niet!

Niemand pakt het op. Ik bedoel, ik kan geen beslissing nemen.

**Het blijft eigenlijk op het bordje van de school liggen.**

Ja, het blijft op het bordje van de school liggen. En die blijft maar verzuim registreren, die blijft maar zeggen ‘ja, hij is weer ziek, zijn programma lukt niet’. En dan komt het zo ver dat je tegen de ouders moet zeggen ‘sorry, hij gaat het niet maken hier op school, want hij is te vaak ziek geweest’. In het geval waar ik over praatte, die nu op de rebound zit.. Wij hebben hem uiteindelijk op de rebound gekregen, dat kost de school heel veel geld, terwijl het geen probleem van de school is, maar een gezondheids- c.q. verzuimprobleem van die jongen. Maar de rebound kost ons veel geld. Wij hebben hem uiteindelijk op die rebound gekregen, terwijl eigenlijk had de kinderbescherming in dit geval al lang de beslissing moeten nemen om deze jongen uit huis te plaatsen. Die beslissing kan ik niet nemen, maar wij zijn al anderhalf jaar met die jongen aan het tobben. En als ik dan bekijk hoeveel tijd mij dat gekost heeft, ja, alleen mij dan, dan heb ik het nog niet eens over onze docenten en over alle anderen die erbij betrokken zijn. Dan denk ik van ‘ja..’. En dat draait in eerste instantie gewoon puur om ziekteverzuim, ‘er wordt ziek gemeld’. En ja, het blijft tobben. Dat is een lastige. Dus ik zou het wat dat betreft wel graag uitbesteden.

(Zorgcoördinator wordt erbij betrokken)

***Geïnterviewde tegen zorgcoördinator*:** Hè, zou jij het ziekteverzuim, de hele organisatie en hele, laten we zeggen ‘behandeling van problematiek rondom ziekte’ willen uitbesteden?

***Zorgcoördinator:*** Het zou erg fijn zijn als dat uitbesteed kan worden, ja. Ook omdat wij heel weinig machtsmiddelen hebben. We kunnen zeggen ‘je bent niet ziek’, maar verder gaat het niet. Als ouders het legitimeren altijd, dan houdt het op.

**Dat dilemma heeft leerplichtzaken ook.**

Ja, maar als je dan de ouders ook heel vaak tegenover je vindt in plaats van ‘naast je’.

Dus vooral bij dat soort ‘vage, niet te definiëren ziektes’. Wanneer is een leerling nu ziek?

**Maar zeg ik dan iets raars als ik denk dat je dat alleen maar onder controle krijgt door er ook snel bij te zijn.**

Nee, dat is helemaal niet raar.

**Want het is een stuk preventie.**

Ja, en daarom zou ik dus ook voor compleet kiezen. Als er ‘heel sec’ vanuit onze administratie blijkt ‘deze leerling is nu voor de 7^e^ dag of deze leerling is voor de 4^e^ keer in 12 weken ziek gemeld’, hier in de organisatie verdwijnt dat wel eens en wordt dat niet de 4^e^ keer in 12 weken, maar de 10^e^ keer in 12 weken voordat we dat in de gaten hebben. Maar als dat gewoon administratief afgehandeld wordt, dus automatisch gaat er een belletje rinkelen bij M@ZL-compleet en die leerling wordt gewoon automatisch opgeroepen voor een gesprek, dan denk ik dat je ontzettend veel winst hebt. Want dat betekent dat, in feite ook wij, als het ware, ‘gecontroleerd’ worden. Dat is op zich niet zo erg. Ik bedoel, nu verdwijnen er teveel dingen tussen ‘wal en schip’. Het kan gerust gebeuren dat een leerling 14 dagen ziek is, zonder dat ik het weet. Dat moet niet kunnen.

**Stel, toch nog één keer, u krijgt een zak met geld en daar zou u M@ZL compleet van kunnen kopen, maar u mag het ook anders besteden, zou u het dan aan ziekteverzuimbegeleiding geven of zou u zeggen ‘dan heb ik nog 10 andere belangrijkere dingen’?**

Ja, dan zijn er ook nog andere dingen. Maar ik weet niet wat die andere dingen dan zouden moeten zijn, dus ik kan daar wat dat betreft.. Want er zijn natuurlijk op school talloze dingen waar wij heel graag geld voor zouden willen hebben. Maar daarom vind ik ook dat M@ZL-compleet dus niet door de school.. Kijk, als je een zak met geld krijgt om M@ZL te betalen, zou ik zeggen ‘oké, ik heb geld gekregen om M@ZL te betalen’. Maar daarom zeg ik ‘nou, nee, leerplicht of weet ik veel wie, de gemeente, betalen jullie nu gewoon M@ZL en helpen jullie ons daarmee’.

**Moeten ze scholen dan verplichten?**

Ik zou het heel mooi vinden als dat inderdaad gewoon een door de overheid geregelde organisatie zou zijn.

**In wet- en regelgeving vastgelegd dat scholen aan ziekteverzuimbegeleiding moeten doen?**

Ja. En dan vind je denk ik ook heel vaak de ouders niet tegenover je, want dan zeg je ‘moet je luisteren, ouders, u wordt alleen maar opgeroepen, hier hebben wij niets mee van doen, dit is gewoon zo geregeld’. En ‘grijp uw kans’.

**Interview 6. M@ZL onderzoek Datum: 19-12-2011**

**Aanwezig: Yvonne Vanneste (onderzoeker), Marlou van de Loo (semi-arts)**

**Directeur school 7 / P_7_**

**Algemene gegevens**

Op school, in kamer directeur. De directeur neemt zelf wat afstand (grote tafel en gaat nog wat ver weg zitten), maar heeft wel een open houding. Het gesprek is wat formeler dan voorgaande interviews, correct taalgebruik. Het is op de achtergrond erg rommelig, er lopen steeds leerlingen langs en tijdens de pauze is het helemaal druk. Ik ervaar dit als storend, maar het lijkt het gesprek niet te beïnvloeden.

Antwoorden zijn helder en veel voorbeelden om standpunt duidelijk te maken.

**Verslag interview**

**Vragen/opmerkingen door interviewster dikgedrukt**

Antwoorden/opmerkingen door geïnterviewde in normale opmaak

**Je hebt vorig jaar ‘ja’ gezegd tegen M@ZL smal, waarom?**

Ehm…. Waarom heb ik dat gedaan? Ten eerste ben jij aardig in het vasthoudend zijn op bepaalde dingen waar je echt achter staat en dat maakt indruk. Maar dat is voor mij niet dé reden. Want ik ben ook naar jou toe heel duidelijk geweest wanneer het voor mij wel of niet zou afspringen. Het financieel aspect lag daaraan ten grondslag. Dus waarom heb ik nu uiteindelijk ‘ja’ gezegd.. Omdat ik er op vertrouw dat het financieel aspect geen rol speelt en ik vervolgens terug kan naar de inhoud en dat betekent dat op deze manier, volgens mij, weer een vinger achter onregelmatig verzuim kunnen krijgen. En dat ‘onregelmatig’ zit hem niet in de periodiciteit, maar zo van ‘hier is iets aparts aan de hand’ en op deze manier zouden we daar misschien wel weer een vinger achter kunnen krijgen. Met twee doelen: om de ‘slechte redenen’ om thuis te blijven ook aan te pakken, maar ook om de ‘goede redenen’ om thuis te blijven goed zichtbaar te maken zodat je daar iets mee kan. Dus het achterhalen: waarom zit een kind thuis. Nou, als er iets serieus aan de hand is, kun je die insteek gebruiken om het kind of het gezin weer verder te helpen, zodat de ontwikkeling van het kind niet stil hoeft te staan. Dat vind ik wel aardig.

**Hoe zie jij daar de rol of de taken van een school in?**

Daar zitten wij nog op het onderzoeksgebied. We hebben daar in dat hele convenant wat afspraken over gemaakt, dat er hier verzuimgesprekken zouden plaatsvinden enzovoorts en dat op enig moment de GGD om de hoek komt kijken, de schoolarts. Ik vind dat nog niet zo helder. Ik zou willen dat mijn collega’s – en welke dat dan precies zijn, docenten, mentoren of bijvoorbeeld zo iemand als Mark – dat die een hele goede benadering van zo’n verzuimgesprek of van dat hele proces zouden hebben. Ik zeg niet dat ze nu een hele slechte hebben, maar ik zou echt die ‘hele goede’ willen hebben. Dat je met onderzoeksvragen, doorvragen, achterhaalt ‘wat speelt hier’. Vanuit de bedoeling om het kind verder te helpen. Om het kind, als het van het spoor af dreigt af te raken, er terug op te krijgen. Of als er iets mee aan de hand is, boven water te krijgen wat we kunnen doen. Dus de school zou daar zeker een betere rol in kunnen spelen, maar dan zit ik niet alleen op verzuim wegens ziekte of wat voor verzuim dan ook, dan zit ik eigenlijk op de grondhouding van mijn collega’s naar kinderen toe.

Waarbij ze toch nog heel vaak, ja.. ik vind deze lastig hoor. Wat ik er lastig aan vind, is dat ik weet dat het gros van de mensen hier in principe de goede inborst heeft naar leerlingen toe, maar niet altijd de juiste/goede professionele benadering daarvoor hanteert. Of in staat is om dat te doen. Wat ik veel zie, is dat we veel ‘pampergedrag’ vertonen in de richting van leerlingen. Dat is op zich prachtig, want dat betekent dat mensen hun best willen doen voor kinderen. Maar tegelijkertijd weten jullie waarschijnlijk dat dat heel vaak een beetje het ‘zachte heelmeesters effect’ heeft, daar krijg je stinkende wonden van. Dat zien we dan ook af en toe. En dat slaat ook door naar de andere kant. Op het moment dat het corrigerend is, gaan ze ook vaak daar wat … als het dan op een gegeven moment allemaal niet lukt of een kind is dwars. Een kind is dwars, nee, een kind dóet dwars. Maar er wordt geroepen ‘het kind is dwars’ en ‘zijn ouders deugen ook niet’.. al dat soort termen komen er dan bij. En dat zijn voor mij niet de goede vragen. Dus ik weet dat ze hun best doen op die kinderen op het spoor te krijgen, maar ze gebruiken dan niet altijd de goede, professionele benadering.

**Wat is daar voor nodig?**Professionalisering. Daar zou een stuk scholing op moeten. Daar zijn we ook mee bezig, in de zin van dat het hele professionaliseringsbeleid opnieuw in kaart wordt gebracht en dat we ook naar dit soort dingen toe gaan om ervoor te zorgen dat onze tijd die we kunnen besteden aan deze kinderen, efficiënter wordt, maar vooral ook beter gaat voelen.

**Ziekteverzuim van leerlingen, heeft dat prioriteit hier op school?**

Het staat niet hoog in de prioriteiten.

**Hoe komt dat?**

Waarschijnlijk komt het omdat we op dit moment genoeg andere dingen hebben die onze aandacht opeisen, zoals bijvoorbeeld het werken aan kwaliteit en opbrengstgericht werken. Waar half Nederland van overloopt op dit moment en wij niet minder. Onze resultaten staan onder druk. We zijn dus ook volop bezig, ik met name, om vakgroepen te activeren om daarnaar te kijken. En resultaatgerichter te werken.

**Prestatiegericht?**

Nee, resultaatgericht. Dat betekent letterlijk wat ik zeg. Uiteindelijk komt er een keer een resultaat op papier, een cijfer, dat soort zaken. En dat levert uiteindelijk ook een oordeel op. Bijvoorbeeld bij de interne audit of bij de inspectie. Dat wil niet zeggen dat we constant prestatiegericht bezig zijn, dat niet. Dat heeft te maken met hoe je je pedagogisch-didactische invulling aan je lessen geeft. Die hoeft niet voortdurend met de zweep erover enzovoorts. Het gaat veel meer om bewustwording van onze collega’s van heel het team, dat er uiteindelijk weer iets uit moet komen. Dat zou je zomaar naast datzelfde verhaal kunnen leggen wat ik daarnet zei. Dat je dus ook daarin een professionelere houding kunt aannemen, zodat je rendement van onderwijs beter wordt. En dat is bepaald geen slechte keuze. Dus zo willen we het ook graag zien. Dat waar de inspectie met zijn vingertje staat, wij dat ombuigen in ons eigen voordeel om het onderwijs beter te ontwikkelen. Vanuit dat punt moeten we eigenlijk vertrekken. Terwijl eigenlijk de thermometer door de inspectie erin gestoken is, wil ik eigenlijk wel naar een gezonde leefstijl van de school.

**Van de leerlingen?**

Niet van de thermometer van de inspectie zit erin, dus nu nemen we maar weer een pilletje en kijken we of we het op kunnen lossen. Of we verzinnen iets en ‘foppen de thermometer’ ofzo. Dat niet. Dan moet het ook goed. En als wij dat goed doen, dan heeft dat voordeel voor de leerlingen. En dat móet het doel zijn en blijven.

**Waarom willen we met zijn allen die aandacht voor ziekteverzuim bij kinderen?**

Om te zorgen dat er niet zoveel schooluitval is. Want schooluitval door ziekte of door ‘vermeende ziekte vooral’, en vooral voor leerlingen die zich op de een of andere manier ook in verkeerde kringen gaan begeven of depressief raken, want die hebben we ook genoeg. Dat je dat boven water krijgt. Als een kind thuis komt te zitten, een beetje gaat hangen en vervolgens toch buitenshuis vertier zoekt enzovoorts. Ja, dat is ‘kat op het spek binden’, daar komt alleen maar rottigheid van. En dan krijgen we dus alleen maar, steeds meer, ‘drop-outs’ in onze samenleving. Dus zorgen dat ze gewoon in een bepaald stramien blijven en dat stramien is gewoon ’s avonds, ’s morgens en laat in de middag thuis en de rest van de dag gewoon op school. Dat is de manier om leerlingen regelmaat bij te brengen.

**Wat verwacht jij van M@ZL?**

Ik verwacht van M@ZL dat wij beter inzicht krijgen in wat leerlingen beweegt om de school niet te bezoeken. Dus op het moment dat dit traject een tijd loopt, dan zou het op moeten leveren dat we beter snappen waarom dat bepaalde kinderen zich ziek melden. Met wel of niet de goede redenen. Dat we wellicht beter leren observeren naar hetgeen er speelt met kinderen. Zodat er ook in onze eigen aanpak, daar iets in kunnen verbeteren. En dat kan op twee gebieden: zowel inhoudelijk als ook professioneel. Want het is hartstikke duidelijk dat als je ‘slap beleid’ voert, dat het verzuim of te laat komen ook gemakkelijker wordt gedaan. Nou, als je er een soort strafkamp van maakt, dan bereik je ook iets, maar dat wil je niet. Maar ik wil wel dat het voor iedereen duidelijk is dat we hier om half 9 beginnen, en niet ‘ongeveer om half 9’. Ik wil ook wel dat kinderen ook snappen dat als ze zich een klein beetje onwel voelen, dat dat niet perse de reden moet zijn om thuis te blijven. Dus dat zij ook een soort, daar moet ik precies het juiste woord voor zoeken.. dat die ‘drive’ om naar school te komen, dat die er in voldoende mate is. Dat ze het onderscheid kunnen maken van ‘nu ben ik ziek, nu kan het niet, nu zou het echt niet goed zijn’ of ‘nu heb ik een verkoudheid of oké ik heb mijn knie gestoten, maar het is niet zo erg, ik ga toch naar school’. Dus dat daar een stuk opgeschoven wordt. Ik heb geen idee overigens in hoeverre dat wij daar hier slecht inzitten. Je hebt destijds wel een stukje statistiek gebouwd, daar had je wel wat zorg in zitten. Dus ik ken de cijfers niet, maar ik weet nog wel dat jij die zorg hebt uitgesproken. Dat is voor mij ook een reden om hiermee aan de slag te gaan.

**Wat voor een rol spelen ouders bij deze ziekmeldingen bij jou?**

Wij hebben vrijwel altijd contact met ouders als het gaat over ziekmeldingen.

**Wie is wij? Wie heeft dat contact hier op school?**

In principe gebeurt dat veelal door de administratie. Dus het is niet zo dat kinderen zich gemakkelijk zelf ziek kunnen melden. Dat heeft meer met de procedure te maken, dan wel de inhoudelijke kant. Dus..

**Maar met betrekking tot inhoud?**

De inhoud, dat is lastig. Daar komen we weer op ‘wat voor contact heb je met de ouders over ziek zijn’. Welke rol heeft daar de mentor in. Of iemand vanuit de lijn, zeg maar. En daar heb ik geen zicht op. Dat varieert ook heel erg. Er zijn mentoren die heel erg betrokken zijn en op dit soort punten de communicatie altijd zoeken, maar er zijn er ook bij die zich daar gemakkelijker vanaf maken en zeggen ‘hij is toch ziekgemeld’.

**Dus er wel mentoren die ook met ziekteverzuim actief aan de slag gaan?**

Die zijn met van alles en nog wat actief, maar ook met ziekteverzuim ja.

**M@ZL smal, je hebt vorig jaar ‘ja’ gezegd, hoe heb je dat hier vervolgens op school gecommuniceerd?**

Nauwelijks.

**Omdat?**

Daar kan ik geen fatsoenlijk argument voor bedenken. Je wilde dat ik eerlijk was.

**Ja, fijn.**

Dat had ik ook al via de telefoon gezegd.

**Dat geeft ook niet. Waarom niet?**

Ik ben een tijdje geleden begonnen met op te schrijven waar ik allemaal mee bezig ben. Ik heb een ‘mindmap’ gemaakt van de taken van de vestigingsdirecteur van deze school. Dat heeft 10 dagen geduurd voordat ik het ongeveer in beeld had. En daar ben ik van geschrokken. Dat heeft me ook weer het een en ander opgebracht en geleerd. ‘Ik wil teveel dingen zelf in de hand houden’. En ik wil misschien ook wel teveel dingen doen, dat kan ook nog. Dat je voor de school teveel hooi op je vork wil nemen. Terwijl ik van bepaalde dingen overtuigd ben dat ze goed zijn, kun je even vrolijk nog niet alles doen. Dus het ‘waarom heb ik het niet gedaan’ heeft naar mijn idee te maken met het feit dat ik telkens weer mijn prioriteiten ergens moest gaan leggen. En ik ben erachter gekomen dat, als je wil dat ook jouw wonden die moeten genezen, dat die fatsoenlijk genezen. Dan moet je niet proberen om 86.000 wonden te verzorgen, want voordat je weer terug bij de eerste bent, moet je al aan amputeren gaan denken ofzo.

Dus, een beetje een omslachtige manier om uit te leggen dat ik probeer teveel dingen draaiende te houden. “En YV belt zo af en toe wel eens, maar niet zo heel dikwijls”.

**Het waarom is wel helder, maar nu de uitvoering nodig. De communicatie intern en met ouders is natuurlijk de allereerste stap. Ik begrijp het heel goed.**

**Kun je me iets vertellen over de rol en taken van de jeugdgezondheidszorg in samenwerking met de school?**

Ehm.. wat ik begrijp, is dat bijvoorbeeld het CJG, in de regio (dat zijn er verschillende). Oudenbosch was een van de eerste, er zijn er 2 in Roosendaal, in Hardenberg. Dat die allemaal nogal redelijk verschillend succesvol zijn en hun werkwijze niet overeenstemmen. Dat ook de gemeentes daar verschillend mee omgaan. Dat de deelnemende partijen er eigenlijk niet allemaal op dezelfde in staan. Maar dat ze wel allemaal willen dat het CJG een laagdrempelig gebeuren zou moeten zijn. CG ken ik dan vanuit het CJG, die is natuurlijk niet de eerste die hier de leiding heeft, dus dat maakt het ook niet gemakkelijk. En als je dan gaat nadenken over ‘hoe zou nu de zorg buiten de school het beste geregeld kunnen worden, zou je zeggen dat zo’n CJG daar erg goed en geschikt voor zou zijn’. En in de praktijk blijkt dat het heel weerbarstig is, om met hen in communicatie te blijven. We hebben natuurlijk wel, bijvoorbeeld de dienst van SMW, dat draait, want die hebben we hier binnen.

Marktland gaat daar nog wat anders mee om, die probeert het ook gewoon binnen te trekken, daar hebben ze zitting. Maarja, daar heeft de wijkagent ook spreekuur en dat zijn geen dingen die ik wil.

Ik wil niet dat de wijkagent hier spreekuur heeft, daar zou ik in mijn situatie, met deze school en deze populatie, het verkeerde signaal door afgeven.

Ik denk ook niet dat het zo zou moeten zijn, dat een school een soort verkapt zorginstituut moet zijn.

Een school is een school. En als je dan een SMW in huis hebt, die af en toe met leerlingen praat die het wat moeilijker hebben, dan kan ik daar goed mee uit de voeten. Op het moment dat dat anders zou zijn, zou ik niet willen.

Wél zou het zo moeten zijn, dat het CJG gemakkelijker toegankelijk wordt voor ouders bijvoorbeeld. Waar dan ook de spilfunctie zou liggen voor doorverwijzing naar vanalles en nog wat. In mijn beleving is dat ook de bedoeling. Dan zou het ook zo moeten zijn, dat die deur vaker open gaat van CJG. Ik bedoel niet te zeggen dat zij de deur dicht hebben, nee, hij zou vaker open moeten gaan doordat mensen die weg zoeken. Maar mensen zoeken die weg niet, dat blijkt.

Het is dus zo dat heel veel ouders, ik heb dan zo af en toe wel eens gesprekken met ouders, en als het daar dan over gaat, hebben ze het idee van ‘ja, zo werkt het nu ook weer niet’. Dan denk ik, nee, nog niet. Dus op tijd daar zijn dat zie je eigenlijk niet echt gebeuren. Dus hetzelfde verhaal als ik hier een informatieavond zou beleggen over het voorkomen van het gebruik van alcohol en drugs door je kind. Als ik een brief rondstuur en vraag mensen zich aan te melden, krijg ik geen zaal vol, dat bestaat niet.

Terwijl ik donders goed weet dat er best wel het een en ander over te vertellen valt. Dat je mensen ook heel aardig zou kunnen helpen bij ‘hoe ga ik om met mijn kind in die leeftijd als al die bedreigingen zich aandienen’. Dat vind ik dus een hele weerbarstige situatie.

En ik maak een hele lange route om aan te geven dat het eigenlijk het proactieve en preventieve werk, op dit moment helemaal niet uit de voeten komt. Vandaar dat ik ook eigenlijk aardig gecharmeerd was van het M@ZL project, omdat dat min of meer een beetje op die route gaat staan. Bij signalen die nog helemaal niet uit de hand zijn gelopen, dus vroegsignalering van problemen, kun je op individueel niveau aan de slag. En dan kun je effect bereiken. Nou, daar zit spanning op.

Dus als het gaat over ‘hoe kun je samenwerking zoeken met verschillende zorginstellingen, dan weet ik dat even niet’. Ik heb destijds ook mee gediscussieerd over de LEA, de ‘lokaal educatieve agenda’, waarbij ook Kentron aan tafel zat en daar werd voortdurend gezegd ‘nou, dan beleggen we een informatieavond, maar dan komt er niemand’. Dan denk ik ‘ja, daar moeten we iets aan doen!’.

Dan kun je wel zeggen dat we nog meer brieven rond moeten sturen, nee, dan moet je er eens over nadenken ‘hoe komt het nou dat je die mensen niet bereikt’.

**En hoe komt dat dan?**

Ja, dat is een goede. Kijk, ik ben zo vrij geweest om dat daar toen te zeggen. Als je alleen maar zegt ‘we hebben het toch georganiseerd en er komt niemand’, ‘als ze het niet willen houdt het op’.

**Dat is een beetje hetzelfde als bij het CJG, we hebben het mooi georganiseerd en er komt niemand.**

Ja, en CG heeft toen aangegeven dat ze bezig was met het bekendmaken van CJG. Dan denk ik ‘ja, bekend maken’, advertenties in de krant, posters. Ze mogen hier ook, dat doen ze ook en dat gaat goed, zich op informatieavonden presenteren. Dat gaat goed, dus ik denk wel dat ze wat dat betreft wat aan de weg timmeren, maar we zijn nog niet zo ver – en nogmaals M@ZL is daar wel een voorbeeldje van – dat er dus gewoon op casusniveau iets aan gedaan kan worden.

**Denk je dat mensen komen op gesprek naar aanleiding van ziekteverzuim? Als ze uitgenodigd worden?**

Ik denk dat de populatie die wij hier hebben wel. Die gaan gewoon allemaal. Gemiddeld genomen wel.

**Jullie hebben M@ZL smal (school doet zelf verzuimgesprek), er is ook M@ZL compleet (strikte criteria, altijd gesprek bij jeugdarts). Daar heb je toen niet voor mogen kiezen, want het geld voor M@ZL compleet was er niet. Stel dat je wel mocht kiezen, wat zou je dan kiezen en waarom?**

Ik denk dat ik compleet zou kiezen, om twee redenen. Het zou hier een stuk ontlasting geven – ik kom zo op de tegenargumenten – want je zou dus inderdaad bij wijze van spreken als ‘boekhouder’ aan de slag kunnen, waardoor je zeker weet dat er een goede opvolging komt. Een tweede voordeel zou in mijn ogen zijn, dat er een eenduidige aanpak te verwachten is van de organisatie van M@ZL. Waarbij je dus professionals die daar ‘van wanten’ weten, dat die daar mee aan de slag gaan. Dat zou voor mij heel belangrijk kunnen zijn. Een soort bedrijfsarts-systematiek.

*Nadeel zou zijn dat wij die expertise, waar ik het daar straks in het verhaal over had, bij mijn eigen mensen, niet in de school zou krijgen. Dus het verzuimcontact, dat kun je leren. Ik zie dat docenten het moeilijk vinden het advies van de jeugdarts te bespreken en uit te voeren. P_7b_*. Dus het verzuimcontact, zoals ik ‘ik hoor bij deze school en ik moet ook met mijn collega’s over hun verzuim praten etc.’. Dus dat zou er weer aan tegenwerken als je M@ZL compleet pakt. Hoewel dat niet betekent dat je dat ook niet met elkaar kan afspreken.

**Je ziet wel een meerwaarde van de professionalisering, ook op dat vlak.**

Ja, zeker weten.

**Wat zou het de school opleveren als mentoren, laat ik voor het gemak zeggen dat de mentoren die verzuimgesprekken gaan voeren, als zij in goed in staat zijn om die verzuimgesprekken te gaan voeren?**

*Ik denk verschillende dingen. Ten eerste denk ik dat er veel meer begrip zou komen, niet alleen emotioneel begrip, maar letterlijk begrip, over situaties. Kennis over wat er speelt bij kinderen en wat redenen kunnen zijn om op school te verzuimen. Het effect daarvan zou moeten zijn dat we daar adequater mee om kunnen gaan en het uiteindelijk effect zou meetbaar kunnen zijn.* *P_7a_* In de zin dat je verzuim afneemt.

**Nog meer dingen die meetbaar worden?**

Tevredenheidonderzoek, zou zomaar kunnen. Bij personeel. Ik denk echt dat als ze het gevoel hebben dat ze meer controle hebben over deze zaken, dan zou die tevredenheid toenemen. Maar ook bij ouders en wellicht bij leerlingen.

**Je zegt het effect is afname van het verzuim. Wat zou je allemaal onder effectiviteit willen verstaan bij M@ZL?**

Ja, ik denk ook dat… Het is altijd even lastig, want je praat natuurlijk niet over een volledige populatie, over die 460 leerlingen. Die 460 leerlingen gaan niet allemaal in een dergelijk soort traject terecht komen. Maar ik denk wel dat ook de ontwikkelingsresultaten van kinderen er beter van worden. En dat neem ik breed. Niet alleen maar leerresultaten, maar echt ontwikkelingsresultaten. Dat er veel adequater dingen boven water worden gehaald, waar je als instantie buiten de school of als school zelf, iets mee kunt. Waar je rekening mee kunt houden in de IAP’s (???) of waar je beter in de verwijzingen terecht kunt komen. Waar je meer informatie naar boven haalt, die in het ZAT eventueel aan de orde kan komen.

**Wat moet er nog gebeuren hier op school om het M@ZL project te laten lopen?**

Dat vind ik wel een probleem, want ik heb daarstraks in het interview al gezegd dat er best veel dingen zijn die onze aandacht opeisen. Er wordt veel druk op mij gelegd om opbrengstgericht te werken en het resultaat van school omhoog te werken. En dan praat men bijna letterlijk over ‘quick wins’. Dit is in de ogen van degenen die hier aan tafel zitten, misschien wel een quick win, maar niet in de ogen van anderen. Bijvoorbeeld ook niet in de ogen van docenten, mentoren. Die zien, als we hiermee aankomen, dit als een extra belasting

**Qua tijd?**

Ja, qua tijd, maar ook in de zin van ‘hebben we nu echt niet iets anders waar we ons mee bezig kunnen houden?’. Want we moeten al vakwerkplannen en een handboek voor ons vak maken, we moeten kijken naar de verschillen tussen de SE en de CE en dan moeten we direct ook nog als een kind ziek is gaan praten over ‘waarom ben je ziek’. Dus daar moet ik weerstand overwinnen en dat is eigenlijk nooit een goede insteek! Want dan ga je er al vanuit dat de weerstand er is, en dat doe ik dus ook.

**Weet je dat ook echt? Is dat ooit besproken?**

Nee, maar als je hier bijna 5 jaar zit, dan kun je dingen aardig inschatten. In vergelijkbare situaties zijn er natuurlijk ook wel eens ‘wat pijlen afgeschoten’, die ook allemaal ‘de ene wel, de andere niet’ wegslagen. Dus als ik hier echt goed mee door wil trekken, dan moet er inderdaad als het ware helder voor ogen staan dat we hier iets mee kunnen winnen. Dan moet ik zo ver zijn, dat mensen ook door hebben dat ze eigenlijk iets tekort komen om dit kind te helpen enzovoorts. Als dat gevoel er is, en dat zie ik wel in beeld hè, dus niet heel dichtbij maar wel wat verder weg, dat de juiste informatie er zou moeten zijn.

**(oogmigraine)**

Ehm.. je moet me even helpen.

**Maar elke verandering geeft weerstand toch?**

Ja, altijd, nou… Ik weet al wat ik nog wilde zeggen.

Binnen het verschiet ligt dat wij over de informatiestroom van leerlingen en leerling-besprekingen gaan praten. Dit is daar wel een aanvulling op, dat je dus zegt van nou, als je de gegevens voor een leerling-bespreking goed wil wegleggen, dan moet je het een en ander van de leerlingen weten. Natuurlijk zijn resultaten, maar ook hoe zijn gedragingen zijn, hoe het met zijn competenties zit. En dan kan dit daar heel goed een rol in spelen.

We moeten het ook niet vreselijk opblazen, want dat is het dus ook niet. Je moet het het liefste ook op proportie houden, zo van ‘nou, dit is een aanvulling, daar kun je wat mee’. Als een kind wat afwezig is..

**Je kunt het als signaal gebruiken..**

De feiten liggen er. Kinderen zijn afwezig. Als we dat goed in beeld kunnen brengen en we kunnen onze collega’s dan ook aanbieden dat we daar iets voor hebben, zodat je er op een goede manier mee om kunt gaan, dat je er wat aan kan doen. Nou, als dat gaat, dan kan het, maar dan moeten we dat wel zorgvuldig op de juiste plek en het juiste moment lanceren. En nu is het eigenlijk een soort…

Goed, YV en ik hebben een aantal goede gesprekken gehad, ze is heel vasthoudend zoals ik zei. Dus uiteindelijk heb ik gezegd ‘nou ik zie er ook wel brood in’, waarom zou je het dan niet doen. Je krijgt het nu voor niets, enzovoorts.. Toen heb ik gezegd, ik doe dat.

**Moet er binnen de jeugdgezondheidszorg ook nog iets veranderen? Voor de samenwerking voor dit project?**

Daar zou ik echt geen antwoord op weten.

**Het contact…**

Kijk YV en ik hebben volop contact en dat verloopt goed. Dus daar zit het niet in. Het is ehm… Nee, daar weet ik geen goed antwoord op. Ik vind het wel een goede vraag.

**En de samenwerking in de uitvoering?**

Nou je helpt me wel. Als het van een directeur af moet hangen, moet je zeggen ‘daar ben ik niet tevreden mee’. Zo hebben we het hier wel geregeld, dus als YV nog meer scholen moet werven, dan moet ze niet stoppen voordat ze de juiste figuur aan tafel heeft waar ze in de praktijk mee verder kan. Dat heeft hier te lang geduurd. En dat heeft een onnodige vertraging opgeleverd. En eigenlijk zou je er ook niet in moeten stappen als je niet zeker weet dat je een goede lancering naar het team kunt hebben. Want dan gaat er volgens mij veel energie verloren. Ook hier bij mij en op school.

**Nou het implementeren van iets nieuws is toch een hele klus. Het begint met ‘ja’ zeggen en dan ga je verder kijken hoe je dit weg kan zetten binnen school. Langzaam aan komt dat wel. Zonder dat je het beseft, is het wel iets waar je nu meer mee bezig bent.**

Het sluit in ieder geval wel aan bij andere dingen die we hier in ontwikkeling willen brengen.

**Kunnen we, als JGZ, daarin iets voor jullie betekenen?**

Ik vraag me af, kijk, communiceren is 1 ding, als het erom gaat dat mentoren dit zouden moeten kunnen, dan moeten ze ook iets leren. Ik weet niet of jullie op dat terrein iets kunnen betekenen. Dat je als het ware ook aan collega’s een soort cursus kan aanbieden. Kan dat?

**Ja, nu hebben we dat op andere scholen al gedaan, maar ze schrijven niet in… Gaan jouw mentoren zich inschrijven?**

Ehh… Ik zal je een leuke anekdote vertellen. We hebben een paar jaar geleden al met het team rond de tafel gezeten om eens te kijken van ‘waar zijn we goed in en willen we ook echt mee door gaan. Waar willen we vanaf, want dat is toch niets. En wat doen we, hoe zou het beter kunnen’. Nou, dan krijg je natuurlijk allerlei mooie plannen. Een van de dingen die het team toen aandroeg, was ‘ja, we zijn allemaal wel mentor, maar we zouden best een mentorentraining willen’. Dus wij organiseren een mentorentraining, samen met nog een paar scholen en daar mochten 18 mensen deelnemen en vanuit ons team mochten we daar de helft van vullen, want wij zaten er echt om verlegen. Dat zijn 9 mensen, dus wij vragen 9 mensen ‘je kunt je opgeven, wij willen graag dat je deelneemt’. Wat denk je?

**Je kreeg het niet gevuld..**

Een hoop gezeik. We hadden er gewoon 9 gevraagd en ze zeiden ‘dat wil ik niet, dat doe ik niet, daar begin ik niet aan’. Van de 9 waren er 5 die eigenlijk zeiden ‘ik liever niet’. Nou, toen hebben we daarmee gesproken en uiteindelijk hebben ze het allemaal wel gedaan. Dus ik herken het wel..

**Hoe komt dat?**

Hier ligt voor mij een ongelofelijk grote uitdaging. Om mensen duidelijk te maken dat ontwikkeling iets is waar je voordurend mee bezig bent en dat het dus ook niet mag en kan stoppen. Alleen al vanuit de CAO is het zo dat ze 10% van hun jaartaak kunnen / zouden moeten besteden aan professionalisering en deskundigheidbevordering. Dat is heel veel! Dat is in heel veel bedrijven en instellingen een klap minder. Hier betekent dat, dat je ongeveer 160 uur per jaar, als je daar weken van 40 van maakt, 4 weken lang zou kunnen / moeten studeren, cursussen of trainingen moet volgen, of vakbladen lezen. Nou, als je dat dan doet en je kan daar 1 uur per week mee vullen, ben je er 40 kwijt. Dus..

**Hoe wordt je mentor hier?**

Nou, als wij denken dat je het goed voor mekaar hebt, een beetje aanleg hebt en een buddy hebt waar je mee aan de slag kunt, dan kun je mentor worden.

**Dat is vrijwillig?**

Nee, wij hebben er 23 nodig, want we hebben 23 klassen.

**Van de hoeveel docenten?**

Ongeveer 45.. dus pak ´m beet de helft van de docenten moet mentor zijn. Maar er zijn er ook bij die maar 2 dagen hier werken, een hoop parttimers, dus uiteindelijk blijft er gewoon een clubje docenten over ´die het zijn´. ´Tikkie, jij bent hem´.

**En dat komt erbij of krijgen ze daar uren voor?**

Daar krijgen ze uren voor.

**En staat er duidelijk omschreven wat de taken zijn, wat ze in die uren moeten doen?**

Dat is ook duidelijk. En ze ervaren dat als behoorlijk belastend. Het is ook zo dat, in het kader van dossiervorming met de digitalisering, de druk op dossiervorming op de een of andere rare manier toeneemt. Waar je vroeger een soort doorschrijfsysteem had, met je aantekeningen, moet je nu allemaal inloggen, opzoeken waar de leerling zit. Ik heb er zelf ook last van. Ik doe even een uitstapje..

Ik heb hier wat uitzendkrachten zitten. In het verleden was het zo dat een uitzendkracht aan het eind van de week met zijn urenbriefje kwam, dan keek ik eroverheen, zette ik er handtekening op, liet hem 1 briefje hiernaast afgeven en dan was ik klaar. Kostte mij 1 minuut. Nu krijg ik voor al die mensen mailtjes, moet ik inloggen en dat lukt niet altijd thuis, dus dat moet hier, en dan moet ik goedkeuring geven…. Bij sommige moet ik nog ingewikkelde stappen op de website maken. Dus per geval ben ik daar nu tussen de 5 en 6 minuten mee kwijt. Dan zul je zeggen ‘dat valt wel mee’, nee dat is de ‘zegen’ van digitalisering. En mijn collega´s ervaren dat dus ook zo.

**Het is ook zo denk ik. Wij herkennen dat ook.**

Natuurlijk, dat kan niet anders. In de hele medische wereld is het dossier veranderd. Ook met alle standaard lijsten en toestanden. Ik ben de laatste paar keer even bij mijn arts geweest en als ik zie hoe hij door dat systeem moet werken, voordat ik weg ben, en dan ben ik er nog. En ik weet niet wat hij daarna nog moet doen. Dat was vroeger een kaartje, daar werd wat opgeschreven en de assistente duwde het weer terug op zijn plek.

**Digitalisering kost veel tijd.**

Jep. Dus dat is één. Nou met dat hele opbrengstgericht werken en alle resultaatmetingen die we doen.. ik heb pas een artikel gelezen dat ‘meten ook niet alles is’. Een van de beste landen ter wereld, Finland, die staat in vele onderzoeken op eenzame hoogte bijna, daar doen ze veel en veel minder aan metingen. Daar investeren ze wel heel veel in docenten, die geven minder les en worden beter betaald. Dat ‘minder les’ klinkt negatief, maar dat betekent alleen dat ze veel beter zijn toegerust om hun werk goed te kunnen doen! Ik herinner me nog dat, toen ik in het onderwijs begon, dat ze net een slag gemaakt hadden van 31 lessen per week naar 29, dat was toen een volledige betrekking. Dat staat inmiddels nu pak-‘m-beet op 26, bij OMO op 21. En toch vinden we het allemaal nog steeds enorm druk. En toen gaven we 29 lessen en hadden we niet echt het idee dat het vreselijk druk was. Er moesten op tijd cijfers komen en er werd een schriftje rondgestuurd als het rapportbespreking was.

**Ligt dat alleen maar aan de digitalisering?**

Nee, die hele systematiek, dat is juist wat ik bedoel. De hele systematiek van ‘volgen’ is veranderd. Dat heeft ook veel voordelen. Ik vergelijk het ook wel eens als volgt: ‘als je met een vliegtuig meegaat, dan worden al die mensen en al die koffers enorm zwaar gecontroleerd om die ene gek op de 700 vliegtuigen tegen te houden… Ik moet mijn riem en schoenen uit doen, alles, terwijl ik als eerzame burger gewoon met het vliegtuig mee wil. Om te voorkomen dat die halve zool, die er misschien wel ergens tussen zit, dat vliegtuig in komt’. Zo doen wij dat nu ook met onze systemen. Wij meten en houden álles in de gaten, proberen alles te volgen, neer te schrijven etcetera. En grote delen van die informatie, staat in de computer en komt er nooit van zijn leven meer uit. Of we printen hem uit, maar doordat we het al in de computer hebben gezet, leest ook niemand die prints meer volledig. We doen dus iets met zijn allen helemaal verkeerd!

Vanmorgen heb ik een filmpje gezien op TedX, ken je dat? Die heeft het daarover. Dat wij langzaam maar zeker niet meer met elkaar in contact komen. We doen het via de mail of proppen het in de computer of we hebben een netwerk. En hij stelt daar hele mooie vragen bij. Ook mensen die in zo’n netwerk zitten, vinden het toch nodig om 1 keer per jaar met 20.000 man een conferentie te houden. Dus schijnbaar kan zo’n conferentie niet per netwerk zegt hij dan heel treffend, schijnbaar moeten ze elkaar toch zien. Zo had jij mij ook een vragenlijst via de computer kunnen sturen, maar dat heb je niet gedaan…

**Nee.**

Dus dit zijn vragen die mij wel bezig houden. Ik weet niet of je dit bedoeld had, maar het zijn wel zaken die mij bezig houden. En deze school, mijn school, heeft als ankerwaarde / kernwaarde ‘aandacht’, relaties. Ik wil er voor waken dat we die niet gaan opofferen aan bepaalde systemen. ‘Kennen en gekend worden’. Dat moet hier blijven. Dat is ook de reden dat ik een paar vragen geleden heb gezegd dat het leuk zo zijn als we M@ZL compleet hadden, maar we zouden ook iets missen.

**Dat is denk ik ook waar. Door zelf die gesprekken te voeren, kom je ook veel te weten.**

**We hebben het er al eerder over gehad. We zitten natuurlijk met geld, de inzet van JGZ kost geld. Zoals je weet wordt de JGZ door de gemeente betaald en moeten we een basistakenpakket uitvoeren. Stel nu dat de gemeente zegt ‘we stoppen met de periodieke onderzoeken en van dat geld geven we ziekteverzuimbegeleiding volgens M@ZL compleet’. Wat zou je daarvan vinden?**

Ehm..

Ik weet niet of ik dat goed vind, dat weet ik niet. Destijds is er een work-around gesuggereerd dat wij zelf lijsten zouden gaan invullen over ziekteverzuim en de redenen waarom kinderen ziek thuis zouden zijn. Als we dan denken dat er met een kind iets is, zouden wij een papier kunnen invullen.

**Dat is breder dan ziekteverzuim denk ik?**

Ja, dat gaat breder dan ziekteverzuim. Als we denken dat er ‘iets’ is met een kind, konden wij die invullen er werd ernaar gekeken. Dat vind ik bloedlink. Ik zou ook absoluut weigeren om die verantwoordelijkheid om mijn personeel af te schuiven. Dat vertel ik ook tegen iedere beleidsvoerder van de gemeente en ook tegen jou heb ik het al een keer gezegd. Het kwalitatief onderzoek door de bevolking heen is voor mij van onschatbare waarde. Ondanks dat ik weet dat je als het ware met ‘een net vist naar dingen’… Dat je dus maar beperkt ‘vangt’. Dat is ook goed, want dat betekent dat je bevolking gezond is. Maar op die manier achterhaal je waar mankementen zitten bij kinderen en of die in grote aantallen zijn, dus ook statistisch, hoe die zich ontwikkelen enzovoorts. Als we dat kwijt zouden raken, dan zou, vind ik, onze gezondheidszorg een stap achteruit gaan. Dus omwille van de verantwoordelijkheid die ik niet bij mijn mensen wil leggen, zeg ik, ik weiger om het te doen, dat is een. Twee, ik zou het ook een gemiste kans vinden om de gezondheidszorg in de brede zin, om die op deze manier achteruit te laten gaan. Als we dat niet meer kunnen betalen als Nederland, dan houdt het op. Maar ik ga die rol liever niet overnemen.

**De rol van signaleerder?**

Correct. Niet zozeer dat wij niet willen signaleren als we denken dat er iets is met een kind. Maar als die verantwoordelijkheid op deze manier bij de school zou worden gelegd, zou ik dat een kwalijke zaak vinden. Je zou, als ik het erg overdrijf, zou je denk ik ook niet willen dat wij hier ‘spreekuren zouden gaan houden voor zweren en breuken’.

**Nee, maar je kunt je voorstellen dat ‘en – en’ wel werkt.**

Ja, dat kan. Als jullie de onderzoeken wel blijven doen. Als de gemeente die wel blijft betalen, dan kunnen wij nog steeds signaleren en vaststellen ‘hee, er is hier iets aan de hand, we sturen door naar de schoolarts’. Dat gebeurt nu eigenlijk al.

**Dat is ook jullie taak zeg maar.**

Ja, exact. Maar het is niet onze verantwoordelijkheid.

**De JGZ moet daar zijn eigen verantwoordelijkheid in nemen?**

Ja, en ik denk ook dat beleidsvoerders, lokale overheden en ook de grote overheid in Den Haag, deze verantwoordelijkheid ook niet moet proberen om op de verkeerde plek weg te leggen. Op het moment dat er in Nederland iets fout gaat, vindt al gauw een Tweede Kamer lid het een mooie taak voor ‘de school’.

**Jeugdgezondheidszorg, preventie, wat weet je van de taken van JGZ? Waartoe zijn wij op aarde?**

Nou, om te beginnen ga ik er nog steeds vanuit dat jullie taak is om een goed beeld te houden van hoe ‘het erbij staat’ met de gezondheid van onze jeugd in brede zin. Dus niet of iemand een gebroken been heeft, maar ook hoe het staat met de complete gezondheid van het kind. Dus de ‘gezonde geest in een gezond lichaam’. Dat in zijn totaliteit. Dat vind ik ook uitstekend, dat die monitor er is.

Dat geeft de maatschappij allerlei mogelijkheden om wat aan die gezondheid te doen. Namelijk concreet, als er daadwerkelijk aanleiding is om een kind te behandelen, maar ook in bredere zin. Dat er op deze manier ook beleid gevormd kan worden, lokaal als landelijk, om bepaalde richtingen op te werken. Bijvoorbeeld wat op dit moment enorm in de belangstelling staat, want we erven alles van Amerika, is ‘obese’ natuurlijk. Dat zijn wel heel belangrijke zaken en doordat jullie daar statistieken over bijhouden, kun je goed zien hoe zich dat ontwikkelt. Dan kun je bij wijze van spreken, als je de metingen van 10 jaar terug pakt, dan zien jullie de weegschalen zwaarder doorslaan. Dat kan niet anders. Dus die rol, die vind ik heel belangrijk. En ik heb ‘obese’ genoemd als voorbeeld, maar ik weet dat er ook hele andere, en misschien wel zorgwekkendere zaken, ook spelen die meer aan de geestelijke gezondheidskant staan. Ik denk dat we daar ook het een en ander van in ontwikkeling zien in onze maatschappij, dat verwacht ik. Dat is een inschatting.

Nou, dat vind ik van onschatbare waarde.

Om de twee redenen, die heb ik eigenlijk nu genoemd.

**Dat is prachtig wat je zegt, klopt ook, maar nu zitten wij met de vraag ‘is het de goede manier van JGZ bedrijven’, kan dat door periodieke onderzoeken te doen bij kinderen?**

Nee. Wat jullie nu aan het doen zijn, met projecten zoals M@ZL, zeg je niet alleen tegen beleidsvoerders ‘er moet wat mee’. Je neemt ook initiatief. Wat zou je dan nog meer kunnen wensen als je beleidsmaker bent en je merkt dat die club die jou moet informeren en die ook iets doet aan de gezondheid, ook nog eens aan de proactieve kant gaat proberen te staan. Hoewel ‘proactief’ natuurlijk een betrekkelijk woord is, maar toch.. Proactiever dan we waren. Dan zou ik zeggen ‘daar moet je blij mee zijn’. Dus ik vind dat jullie niet berusten in de rol die je had en dat vind ik ook prima. Jullie nemen je verantwoordelijkheid, jullie zijn niet volgzaam. Je pakt het aan. Perfect.

**Wil je nog wat kwijt over ziekteverzuim? Begeleiding? De rol van de gemeente? Ik zeg maar wat..**

Ehm..

Wat we merken is dat het ook erg afhankelijk is van… we hebben hier natuurlijk te maken met diverse ambtenaar leerplichtzaken. Dat gaat, als ik het goed heb begrepen, veranderen. Er wordt nu gezocht naar schoolcontactpersonen, waar je langer mee kan samenwerken. Dat vind ik een grote plus. Zo kun je een relatie opbouwen en een ‘workground’ kiezen die bij je past in plaats van 4 of 5 verschillende. Dus dat vind ik goed.

Ik zou ook willen nadenken over, ehm.. als ik even kijk naar mijn personeel, daar hebben we de ziekmeld-drempel gemaakt op een manier dat ze mij moeten bellen. Dat is altijd even iets lastiger dan de administratie bellen. Dus zoiets zou misschien ook wel interessant zijn om te kunnen kijken van ‘wat wordt nou de drempel om je ziek te melden’.

**Heel mooi dat je dat zegt. Misschien is het een goed idee om je bij de mentor te laten ziekmelden?**

Die zal daar zeer zeker niet blij mee zijn.

**Nee, maar het verhoogt wel de drempel.**

Ja, we zouden ook kunnen bedenken dat iemand aan de telefoon daartoe wordt opgeleid. Ze doen dat hier niet zo slecht, ze stellen ook wel eens vragen, doorvragen etcetera. Maar als er bijvoorbeeld iemand zou zijn die… Ik zou me dat kunnen voorstellen. Dat er toch op dat punt ook… Misschien niet zozeer per functionaris, want jij spitst het toe op een persoon. Maar ook, hoe kun je nu inhoudelijk, als de ziekmelding er is, de juiste vragen stellen.

**En mensen misschien ook motiveren?**

Bijvoorbeeld. Ik heb het ook meegemaakt, dat mensen zich uiteindelijk toch niet ziekmelden, dat gebeurt. Of korter. Zo van ‘ja, ik denk dat ik het deze week niet meer red. Zullen we dan morgen nog een keer bellen’.

**Dat zijn dan heel belangrijke dingen die je ook met kinderen en hun ouders kunt afspreken.**

Misschien zouden jullie daar vanuit de JGZ aan kunnen werken.

**Dat is misschien een hele goede.**

**Maar de verzuimdrempel die zal, dat klinkt misschien onaardig, die zou door school toch opgepakt moeten/kunnen worden. Want daar komt het verzuim binnen.**

Dat is ook zo, maar omdat jullie nu vanuit.. Ik bedoel niet dat ik het op jullie wil afschuiven, maar omdat jullie hier zo nadrukkelijk mee bezig zijn en op al die scholen komen, zou je daar die ‘vlek’ kunnen wegzetten.

**Je zou inderdaad eens kunnen nadenken over ‘welke vragen stel je nu, hoe kun je die drempel op het moment dat het gemeld wordt, op kunnen krikken’. Daar gaan we mee aan de slag. Nog een laatste ding. Ik zag jou net heel snel nee schudden toen ik vroeg naar ‘is het doen van periodiek onderzoek een manier om JGZ te bedrijven’. Hoe zouden we het wel moeten doen?**

Nou, dat periodiek onderzoek vind ik wel goed, maar dat zou niet de enige taak moeten zijn. Dan zou je in de regie ‘de volgzame zin’ erin gaan staan, zo van ‘oké, die onderzoeken moeten gebeuren, dus we meten de lengte en wegen het gewicht, kijken even in de poppetjes van zijn ogen en doen een gehoortest, weer een stukje statistiek gewonnen’. Als het dat alleen maar zou zijn, dan zou dat doodzonde zijn. Als je dus met dat hele scala niets doet. Je kan natuurlijk zeggen, ‘we zijn alleen maar de intermediair en we geven het af aan de gemeente die er iets mee kan’ of ‘in een bepaald geval verwijzen we door’. Dat zou ik doodzonde vinden. Ik denk, met respect voor al jouw collega’s die dit in het verleden gedaan hebben, dat dat veelvuldig het geval is geweest. Die periodieke onderzoeken.. afwerken, ik ga weer naar die school… en de assistente speelde daar een heel belangrijke rol in. Dat weet ik, want ik heb ze zelf ook voorbij zien komen, ben er zelf ook ooit geweest. En dat zou het tekort doen. Maar ik zou het ene niet willen weggooien voor het ander.

**Bedankt.**

**Interview 7. M@ZL onderzoek Datum: 20-12-2011**

**Aanwezig: Yvonne Vanneste (onderzoeker), Marlou van de Loo (semi-arts)**

**Zorgcoördinator school 2 / S2**

**Variant: first route**

**Algemene gegevens**

Het gesprek vindt plaats op school, in de kamer van de zorgcoördinator. Marlou start met het gesprek! Eerst nog even spannend, maar gesprek verloopt daarna goed. Nu ik het gesprek zelf doe, merk ik pas hoe inspannend en vermoeiend het is om zelf een interview af te nemen. Het is fijn dat Yvonne later kan bijvallen en we het gesprek samen af maken.

**Verslag interview**

**Vragen/opmerkingen door interviewster dikgedrukt**

Antwoorden/opmerkingen door geïnterviewde in normale opmaak

**Kunt u uzelf voorstellen?**

Ik ben AdK, ik ben hier zorgcoördinator van de onderbouw. Officieel is het zo dat er op elke school 1 zorgcoördinator is, maar wij hebben dat verdeeld en ik doe de onderbouw. Mijn collega, die normaal daar zit, doet de bovenbouw.

**Bent u al lang zorgcoördinator?**

Ik weet nooit hoe lang, maar wel al een jaar of 7 of 8. Ik weet het niet precies.

**Ben je daar zo ingerold?**

Nee, ik ben hier 21 jaar geleden komen werken als docent Engels en Aardrijkskunde. Na een paar jaar ben ik een studie erbij gaan doen voor ‘remedial teaching’, om dat hier op school vorm te gaan geven. Van dat ben ik zo van het een in het ander gerold. Toen werd ik coördinator van de eerste klas, maar dat was toen een hele andere taak. Op een gegeven moment werden hier allerlei opnieuw bekeken en uitgebreid en toen ben ik daar zo ingerold. En ik geef nu nog maar heel weinig les, nog maar 6 uurtjes, en voor de rest heb ik dus allerlei andere taken.

**Het project M@ZL, wanneer heeft u daar voor het eerst van gehoord, hoe is dat begonnen?**

Ja, wij deden al heel lang mee met het oude verzuimproject van de GGD, ik weet eigenlijk niet hoe lang, echt al heel lang, daar zijn wij toen ook als een van de eerste scholen volgens mij ingestapt.

*Yvonne: Ja, tien jaar misschien al.*

Ja. En toen kregen we op een gegeven moment van de GGD-arts te horen dat het project zoals het toen was ging stoppen en er een ander project voor in de plaats kwam. En dat is toen geweest toen het opgestart is. We doen M@ZL nu voor het tweede jaar, dus nu denk ik twee jaar geleden dat we dat dan hoorden. Toen ben ik naar zo’n voorlichtingsmiddag geweest op Prinsentuin, daar moest Yvonne iets vertellen over het project en wat er anders was aan hoe dit project in elkaar zat, in vergelijking met het oude project. En toen zijn we ermee gestart.

**En hoe ging dat? Werd dat van de een op de andere dag gestart binnen de school? Wie is daar allemaal bij betrokken?**

Ja, omdat we dat oude project hadden, is dat hier niet echt een revolutie geweest ofzo. We zijn gewoon door gegaan, zo van ja, ‘we hebben een GGD-project’. Alleen, waar ik net al een beetje aan refereerde, in de bovenbouw, dat andere project was daar vanwege kosten, want hier draagt het samenwerkingsverband aan bij in de kosten, daar zitten VSV-gelden in enzo. En dat andere, dat werd gedeeltelijk door de gemeente geloof ik gesubsidieerd en gedeeltelijk door de school, maar dat was voor ons veel duurder. Dus toen is op een gegeven moment gezegd: ‘we gaan het alleen in de onderbouw doen, in klas 1 en 2’. Omdat we in klas 3 en 4 vaak toch wel weten wat de problematiek van kinderen is en dat de mensen die vooral in de bovenbouw zitten, daar de meerwaarde wat minder van inzagen. Maar toen we met dit project zijn begonnen, hebben we wel weer gezegd ‘dit gaat weer gelden voor de hele school’. Ook omdat het uit VSV-gelden betaald wordt en VSV speelt eigenlijk meer in de bovenbouw natuurlijk, hè, voortijdig schoolverlaten, dan in de onderbouw.

Maar dat is wel een beetje, dat is wel moeizaam. Klas 3 en 4 komen ook elke keer van ‘ja, maar dit hoeven we toch niet door te geven aan de GGD’. Jawel, we hebben afgesproken dat we alle leerlingen doen, tenzij er…

**En wie komen daarmee?**

Mentoren. Ja, en het is ook wel, dat R. (*Marlou: de bovenbouw coördinator)* en ik er ook een beetje anders in staan. Ik ben best wel netjes, zo van ‘nou, dit moeten we doen, dus dat gaan we doen’. En ik trek dan die kar in de onderbouw op een andere manier dan R. Hij is dus van de bovenbouw en die kijkt daar toch een beetje anders tegenaan. Die vindt het dan ook moeilijker, als je er zelf niet helemaal 100% achter staat, is het ook moeilijker om andere mensen, die ook al sceptisch zijn, daar in mee te trekken. En in de onderbouw is dat wat gemakkelijker, ook omdat het al zo lang in de onderbouw gewoon gebruikelijk is dat we dat doen.

**En die andere mensen die sceptisch zijn, dat zijn de mentoren?**

De mentoren ja, maar R. ook wel een beetje.

**Is dat een verschil tussen mentoren in de onderbouw en bovenbouw? Waar ligt dat aan, dat die mentoren zo sceptisch zijn?**

Ja, dat weet ik eigenlijk niet. Geen idee. Ik denk dat het er wel mee te maken heeft, dat we het in de onderbouw altijd zijn blijven doen, met dat oude project ook. Want daar zaten toch wel wat haken en ogen aan, maar dat we het altijd zijn blijven doen, voor die mentoren van klas 1 en 2 is het ‘het hoort er gewoon bij, het is gewoon zo’. Die maken zich daar ook niet meer zo druk over.

Ze hoeven er ook helemaal niet meer zoveel werk aan te doen, want eigenlijk alles wordt voor ze gedaan door de computer, door mij en door R. Het enige wat ze moeten doen, is af en toe, maar dat vind ik heel normaal, ouders bellen en wat informatie aan mij doorgeven. Maar de hele verdere verwerking dat doen wij. Terwijl ze daar vroeger zelf een grotere rol in hadden. Dus.. ik weet het niet. Het is wel dat soms collega’s, en dat snap ik ook wel, zich afvragen van ‘wat is nu de meerwaarde van het advies wat we krijgen’. ‘Wat kunnen wij als school, wat levert het ons op’. En dat snap ik ook wel een beetje, er zijn van die kinderen die gewoon 4 jaar lang altijd veel ziek zijn, waar je ook geen vinger achter krijgt. Dat je denkt ‘het is een beetje flauwekul’, maar ze blijven wel gewoon ziek. Dit hele project gaat daar toch een beetje aan voorbij. Die mensen gaan dan braaf naar de GGD, gaan daar braaf praten, maar melden hun kind gewoon net zo makkelijk weer ziek. En dat duurt vier jaar lang zo. Dan kun je je afvragen, op een gegeven moment gaan de ouders ook weerstand krijgen van ‘ja, moet ik daar elk jaar naartoe’. Wat wij willen is dat die ouders daar dan is over na gaan denken, van ‘nou, misschien, hè..’. Maar dat gebeurt dan niet. Dat verandert niet veel in hun gedrag zal ik maar zeggen. Dat is ‘een groep ouders’. En dat is nou juist de groep ouders waarvan wij eigenlijk willen dat we daar wat meer grip op krijgen. Die hun kinderen naar ons gevoel heel gemakkelijk ziek melden en heel gemakkelijk thuis houden, maar ja, dat is een beetje ongrijpbaar.

**Is dat een bepaalde groep ouders die bepaalde kenmerken heeft of is dat heel verschillend?**

Nee, dat weet ik niet. Dat zit in alle… Nee. Dat zit eigenlijk in alle lagen van de bevolking, zal ik maar zeggen, zitten van deze ouders. Die dat makkelijk doen dan.

**Zijn er momenten dat je wel de meerwaarde ziet van de jeugdarts?**

Ik zie wel meerwaarde, maar ik kan me voorstellen dat, en daarom zeg ik ‘dat speelt in de bovenbouw anders’, dat deze groep waar ik het net over had, die zo ongrijpbaar is. Waarvan de mensen ook zeggen ‘moet ik daar nu weer naartoe, ik vertel daar elke keer hetzelfde verhaaltje’. En wij zien dan ook geen verandering. Ik kan me voorstellen dat, vooral in de bovenbouw, want die zien dus in ons systeem staan van ‘ja, die zijn al zo vaak geweest, die hebben al die adviezen al gehad, mensen hebben daar niets mee gedaan en nou moet ik daar toch weer met die ouders over in gesprek’. Dus, ik vind, je moet niet opgeven om het te proberen, maar ik zie wel de meerwaarde ja.

**Want hoe groot is de groep waar je eigenlijk geen meerwaarde ziet ten opzichte van de groep waarbij het wel nut heeft?**

Dat durf ik zo niet te zeggen, nee, dat durf ik echt niet. Dan zou ik uit jouw getallen ofzo.. Want er zijn kinderen die worden gewoon 2 of 3 keer in een jaar aangemeld voor M@ZL of het oude project, 2 of 3 keer in een jaar, dat is veel, dan ben je veel ziek geweest. Waar die ouders soms wel, maar soms ook niet gaan, ‘van ja, nou, ik vind het gewoon onzin’. Die melden we netjes het jaar daarop weer aan en dan gaan ze ook weer wel of niet. Die kinderen blijven gewoon veel te veel ziek. Zonder dat daar heel erg een aanwijsbare reden voor is. Maar daar waar, en zeker nu we een goede arts hebben, die goede adviezen geeft, dan zie ik echt wel een meerwaarde.

Van de week hadden we nog een meisje waarvan ook de moeder zelf al.... De mentor had contact gezocht met de moeder, een allochtoon meisje, een Marokkaans meisje waar dat toch denk ik al wat moeilijker ligt, die vaak thuis bleef vanwege menstruatieproblemen. Dan heeft S. (*Marlou: de jeugdarts)* toch wel dat gesprek met die moeder gehad en dan blijkt uit het advies dat te verwachten valt dat dat probleem is opgelost. Nou, dat vind ik echt heel knap, als je dat bij een Marokkaans meisje voor elkaar krijgt. Want daar zal toch iets van de pil, of hormonen of iets, denk ik, in ieder geval een rol spelen. Zoveel inzicht heb ik daar niet in, maar dat lijkt mij, dat is toch altijd de manier om van die klachten af te komen.

**Wat verwacht je dan? Dat het ziekteverzuim echt af gaat nemen?**

Ik denk het wel bij haar. Het is nu nog te kort, want het advies is pas deze week binnen gekomen. Maar dat soort adviezen, dan denk ik ja, dat kan ik aan ouders uitleggen van ‘kijk eens hier, het heeft wat opgeleverd ook, dat jullie daar zijn geweest’. En het kan ook voor ons soms eh.. Als wij dan bijvoorbeeld horen dat, het is nooit onderzocht, maar er was hier een lokaal waar veel kinderen ziek werden. Nou hebben wij wel een luchtzuiveringssysteem, daar is veel geïnvesteerd, dus daarna is het niet meer onderzocht, maar er was een tijdje sprake van ‘in dat lokaal is iets’. Dus dat is ook naar ons toe, het werkt twee kanten op. Dat probeer ik ouders ook altijd uit te leggen. Maar het blijft heel moeilijk, ik denk dat het ook uit jouw onderzoek zal blijken, het blijft heel moeilijk of het ook echt meetbaar is dat het terugloopt.

Maar van sommige adviezen zie ik echt wel de meerwaarde van in.

**En zien de mentoren dat dan ook? Praat je daar wel eens over?**

Ja, doordat onze directeur hier (J.O.) heel weinig nu op de vestiging zit, zijn hier wat taken veranderd en heb ik niet meer het wekelijks overleg met de mentoren zelf. Omdat ik wat taken van hem heb overgenomen, maar normaal bespreken we dat in het mentorenberaad. Maar dat doet een collega van mij nu.

**Nog even over die mentoren. De computer geeft de melding, het enige wat zij moeten doen is een telefoontje naar de ouders om te vragen wat er aan de hand is?**

Ja, en om te vertellen ‘nou, dit komt eraan’. Want dat is wel een van de dingen die we vorig jaar niet goed deden, ouders informeren, en dan kregen we heel vaak boze ouders kregen van ‘ja, moet ik hier nu naartoe, wat een flauwekul, hadden jullie me niet eens kunnen waarschuwen’. Dus we proberen nu altijd het contact met de ouders te zoeken voordat de melding de deur uitgaat. Dus dat is wel een verbetering denk ik ten opzichte van vorig jaar.

**En die mentoren, als ze de ouders bellen, is er dan een soort structuur die ze aanhouden of waar ze naar vragen?**

Nee, dat is toch ook een beetje eigen.. Ik wil dat verder ook niet heel erg structureren. Zo lang als ik de informatie krijg die ik wil hebben om door te geven en ouders op de hoogte zijn, vind ik dat verder wel prima.

**En hoor je ook iets terug over die telefoongesprekken? Hoe ouders reageren?**

Ja, dat is ook heel wisselend. Sommige ouders blijven gewoon echt zeggen ‘ik ga hier niet aan meewerken’. Het typische is dat ze dat niet tegen die mentor zeggen, maar dat ze dan mij bellen. Dan gaan ze eerst zitten wachten totdat ze die brief krijgen, de uitnodiging. Ik denk dat ze anders denken ‘het zal z’n vaart niet lopen ofzo’, ik weet het niet, en dan gaan ze mij bellen. Ik heb gisteren weer twee gesprekken gehad van ouders die zeiden ‘ik wil daar eigenlijk niet naartoe’.

**En probeer je ze dan nog te overtuigen?**

Dat ligt eraan met wat voor verhaal ze komen. Gisteren was het eerste verhaal, dat was een meisje dat voor de 4^e^ keer ziek was in 12 weken, en de moeder zei in eerste instantie, terwijl ik van de mentor iets anders had gehoord, ‘ze heeft gewoon iets niet goed uitgeziekt, we hebben haar steeds weer te snel naar school gestuurd en dat hebben we nu wel in de gaten, dat moeten we niet meer doen’. Dat kan.

Maar toen was ik met haar nog aan het praten, want ik had ondertussen op de computer de aanmelding open gemaakt en daarin zag ik dat het meisje onzeker was en daardoor veel spanning opbouwde en dat vond ik iets heel anders dan ‘een griepje niet goed uitgeziekt’. Dus toen vroeg ik haar daarnaar en toen zei ze ‘ja, dat klopt ook, ze is ook onzeker. Maar wat kan de GGD-arts daaraan doen?’. Nou, die zou eens kunnen kijken waar die onzekerheid vandaan komt, waar heeft dat mee te maken, is het faalangst of is het in een sociale onzekerheid… En toen begon die moeder nog meer te vertellen en toen bleek dat het meisje ook heel veel uit de weg ging. Zo van ‘als ik het maar niet doe, dan hoef ik me er ook niet druk over te maken’. Maar dan bouw je juist veel meer spanning op! Dus die moeder vertelde eigenlijk een heleboel, waarvan ik dacht ‘ja, daar kan wel..’. Maar ze bleef ervan overtuigd, ze zag geen meerwaarde van een bezoek aan een arts en ze vond het ook eigenlijk onzin dat het kind daardoor wéér, dat is wel iets, weer een les zou missen. Uiteindelijk voelde ik aan die moeder ‘die gaat dit helemaal niet doen’. Ik ben blijven zeggen dat ik het belangrijk vind dat ze wel gaat, want wij willen graag weten wat voor een hulp we moeten bieden om haar van dit probleem af te helpen, maar dan moet je wel eerst weten wat precies het probleem is. Ik denk dat de GGD daar juist wel een bijdrage in kan leveren. ‘Nee! Mijn dochter gaat daar niets zeggen, die gaat alleen maar zeggen dat het goed gaat en dat ze het naar haar zin heeft op school’. Ze zou het nog een keer met haar dochter bespreken. Toen heb ik gezegd: ‘ik hoor graag van u terug dat u van de afspraak / het aanbod gebruik gaat maken. Als u dat niet doet, ga ik wel hier op school verder kijken’. Dus dan gaan we een test doen (S.S.A.T.), is om uit te zoeken of kinderen faalangst hebben of vermijdingsgedrag. Ik hoorde uit het verhaal van die moeder het vermijdingsgedrag, zo van ‘alles uit de weg gaan en daags voor een proefwerk pas beginnen en niet meer weten hoe ze het aan moet pakken’. En dan kreeg ze buikpijn. Dus dat kan ik hiermee uitzoeken en dan kunnen we daar wel een training op zetten.

**En dat ga je dan zelf doen?**

Ja, dat ga ik dan zelf doen. Maar ik zou dat wel liever ondersteund zien, dat heb ik ook tegen moeder gezegd. Want het meisje klaagt wel ook over buikpijn en ze blijft er van thuis, dat doen niet alle kinderen met dit soort problematiek, dus ja.

**Dus daarin zie je de meerwaarde van de jeugdarts?**

Ja, dus ik heb echt met die mevrouw 25 minuten aan de telefoon gezeten, want ik vind dat echt belangrijk, maar ik heb haar alleen denk ik niet kunnen overtuigen. Maar ik heb het nog niet gehoord, ze zou me nog terugbellen.

En een andere moeder, dat was een meisje, die heeft kinkhoest gehad en is daarvan heel erg lang thuis geweest. Dus die was dat andere criterium. En bij dat andere criterium snap ik vaker waarom ouders zeggen ‘ik ga daar niet naartoe’. Want dan is meestal heel erg duidelijk, en dan zijn er ook al heel veel contacten geweest tussen mentor en ons, dan is vaak wel heel erg duidelijk wat er gespeeld heeft. En dan zie ik ook niet altijd de meerwaarde. Dit meisje, ja, ze komt nu weer naar school, ze gymt niet mee, er is een goed programma opgesteld om alles in te halen en de mentor heeft daar intensief contact over. Die moeder zei ook dat dat allemaal prima gegaan is. Dan zie ik het niet zo. Dus die mevrouw heb ik snel ‘laten gaan’, zal ik maar zeggen. Daar heb ik niet zoveel moeite in gestopt als in die andere mevrouw. Maar dat ‘4^e^ keer ziek’ vind ik eigenlijk altijd, dan moet je gewoon gaan. Want het is heel raar als je 4 keer in 12 weken ziek bent, dat blijf ik vinden. Maar ja, nu had ik uit diezelfde klas waar dat meisje in zat met die kinkhoest, een jongen die 12 dagen ziek is geweest. Ik kreeg een brief je van moeder van: ‘hij is 12 dagen ziek geweest, verkoudheid en astma’. Toen dacht ik ‘ik werk hier al best lang, maar 12 dagen ziek van een verkoudheid en astma, dat vind ik heel erg lang’. Dat heb ik tegen die mentor gezegd, van ‘ja, dat andere meisje, die kinkhoest dat snap ik en ze komt ook weer naar school’. Maar dit is een verhaal, daar lees ik ook in, verkoudheid en astma, astma komt vaker terug, dan wil ik wel een advies van de arts. Want ja, als hij regelmatig zo lang ziek blijft vanwege zijn astma, dan gaat dat op school wel problemen opleveren. Dus daar wil ik dan wel.. Dus ja, zo probeer ik wel een beetje te kijken van ‘wanneer heeft het nut’.

**Dan kom je eigenlijk een beetje bij het nut van ziekteverzuimbegeleiding. Wat zijn de belangrijkste gevolgen?**

Ik vind het een goede zaak om dat gesprek met die ouders die veel te gemakkelijk ziekmelden aan te gaan. Ik vraag me ook altijd af of ouders die veel werken enzo, maar goed, daar heb ik niet zo heel veel zicht op, of die niet te gemakkelijk zeggen als hun kind naar moeder belt, die in de file staat, dat hij zich echt niet lekker voelt, dat moeder zegt ‘ik bel wel naar school, blijf maar thuis’. Dat weet ik niet helemaal. Dat vind ik goed dat ouders daarop gewezen worden. Van ‘het is niet zomaar wat als je je kind ziek meldt’. In dit soort gevallen als die jongen met dat astma-verhaal, iets wat heel vaak terug gaat komen, wil ik wel dat we als school weten hoe we daarmee om moeten gaan wat we eventueel kunnen doen om dat te laten verminderen. Als we bijvoorbeeld, ik kan me voorstellen, ik ben zelf ook allergisch voor het een en ander, ik loop wel eens lang het technieklokaal als ze daar aan het figuurzagen zijn enzo, echt verschrikkelijk, daar krijg ik het ook benauwd van. Dus ik kan me voorstellen dat je dan met zo’n jongen, ik weet niet, dat we daar als we dan adviezen krijgen van ‘misschien moet je dat soort situaties proberen te vermijden en dan kan hij wel vaker naar school’. Dat vind ik dan wel het nut van ziekteverzuimbegeleiding. Dus dat soort dingen, daar zie ik..

En ook naar ons toe. Soms komt daar toch uit dat kinderen zich hier op school niet prettig voelen. In de klas of bij een docent, terwijl wij dat niet weten. Terwijl wij denken ‘dit kind heeft het gewoon naar zijn zin op school’, maar dat de drempel om thuis te blijven vrij laag is, omdat ze gepest worden, maar dat nooit hebben durven zeggen. Dat vind ik altijd typisch, dat dat dan daar wel boven water komt. Dus het is naar ouders toe en naar ons toe, vind ik, wel belangrijk dat wij weten hoe we er samen voor kunnen zorgen dat het minder wordt.

**Want wat zijn de gevolgen voor het kind als hij zo lang thuis is of vaak ziek is?**

*Nou ja, kinderen die vaak ziek zijn, sociaal hè. Want ze maken een hoop in de klas niet mee. En ook qua stof missen ze. Sommige kinderen kunnen het allemaal maar met moeite bijbenen. Die hebben al problemen met het plannen van hun huiswerk, en als ze dan ook nog eens veel ziek zijn, dan wordt dat heel erg lastig. Docenten moeten daarmee dealen. Daarom heeft verzuim altijd veel gevolgen niet alleen voor de leerling maar ook voor de docenten. S_2a_* Dus dat vind ik voor kinderen wel twee hele belangrijke. Vooral dat sociale ook. We hebben nu een manneke, uit de eerste klas, die is in de eerste week ziek geworden en die komt nu nog steeds niet volledig naar school. Het is vooral psychisch bleek later, maar de eerste klachten waren lichamelijk, maar dat is geleidelijk aan psychisch geworden. Hij komt nu nog steeds niet volledig naar school, maar de drempel om weer naar school te komen, werd ook steeds hoger, omdat hij ook dacht ‘ja, mijn klasgenootjes zullen ook wel denken en leraren gaan misschien dingen van mij vragen die ik niet kan’. Daar ging hij zich nog meer druk over maken, dus het werd nog moeilijker om terug naar school te komen. Dus bij hem is heel duidelijk, ook omdat het toevallig aan het begin van het schooljaar klas 1 was. Nieuwe school, wist van ‘toeters nog blazen’, hij heeft allemaal boeken, maar weet niet wat hij er mee moet. Dus voor hem werd het een hele onoverzichtelijke toestand. Zijn psycholoog zei: ‘hij heeft het idee dat hij op het station staat en dat de trein vertrokken is en dat hij niet weet hoe die in die trein moet komen’. ‘Er achteraan rennen heeft geen zin’. Ja.. We hebben hem op de trein hoor, hij hangt een beetje in de laatste coupé nu.. (lacht).

Hij gaat nu een half programma naar school nu. En hij zou lichamelijk nu hele dagen aankunnen, maar dat is nog een stap te ver, omdat hij nog zoveel moet inhalen. Dus hij is nu thuis bezig met wat voor zichzelf, dus hij kan het geestelijk nog niet aan om hele dagen naar school te gaan. Lichamelijk eigenlijk wel. Maar ik denk dat we hem na de vakantie.. Als hij niet in de vakantie weer een dip krijgt, want dat kan natuurlijk, dat hij er toch weer tegenop ziet.

**Heeft de jeugdarts daar dan iets in kunnen betekenen?**

Ja, zeker. Hij was ´onze eerste die we konden aanmelden´ (die jongen met al die buikpijn). Ja, zeker. Zij heeft gesprekken met hem gehad, met zijn ouders en er is contact geweest tussen die psycholoog en de jeugdarts. We hebben hier uiteindelijk nog een groot overleg gehad, want op een gegeven moment was hij zo ver dat hij wel weer een beetje naar school kon komen en toen hebben we achteraf gezien eigenlijk de verkeerde strategie gekozen. In overleg met de psycholoog hebben we er toen voor gekozen dat hij steeds het 3^e^ uur zou komen, dan hoefde hij niet zo vroeg op te staan. ´Dan kom je gewoon de hele week het 3^e^ uur en volgende week het 3^e^ en 4^e^ uur..´. Maar ja, dan had hij een les gevolgd, die hij niet snapte, want hij wist absoluut niet wat eraan vooraf gegaan was, en dan kreeg hij die pas de volgende week weer. En daar tussendoor was al weer een heleboel gebeurd. Dus daar werd hij weer helemaal paniekerig van. Toen hebben we gezegd ´we gaan het anders doen, we gaan hem vakken geven´. Dan krijg je wel een beetje een ´gatenkaas-rooster´, maar je gaat nu deze vakken volgen. Zodat je voor die vakken in ieder geval ‘alweer op de trein zit’, zal ik maar zeggen. En dat heeft goed gewerkt. Dat hebben we in intensief overleg met elkaar gedaan.

**Wat goed.**

Omdat bij hem dat verhaal zo heftig was, want bij andere kinderen is het minder heftig. Je kon goed zien dat hij echt de angst had van ‘ja, in de klas zullen ze ook wel iets raars van mij vinden’.

**Was dat ook zo?**

Nee, maar ja, hij kende die kinderen natuurlijk …

**Waren er reacties op school?**

Niet zo ver ik weet. Ze maakten zich wel zorgen, sommige kinderen die hij van de basisschool kende, hadden natuurlijk wel contact met hem. Ze zitten altijd toch wel met een paar vriendjes van de basisschool in de klas. Dus die hadden wel contact met hem. Er was een ander jongetje, die had in die eerste week zijn voet gebroken, of weet ik wat, dus die is in die eerste week ook een paar dagen niet geweest en is toen met een rolstoel gekomen. En dat was toevallig zijn vriendje.. Dus die twee hebben wel contact gehad, maar het is hem zo boven het hoofd gestegen. Terwijl, toen hij terug kwam heeft de klas hem eigenlijk meteen heel erg liefdevol weer opgevangen. Dus dat gevoel was eigenlijk ook wel snel weg. Dat probleem was eigenlijk niet zo groot. Maar de stof, daar zag hij toch wel het ergste tegenop. Van ‘hoe moet ik nou…’.

**Dus de jeugdarts heeft hier een goede rol bij gespeeld?**

Ja.

**Hoe is verder uw beeld van de jeugdgezondheidszorg? Wat weet je daarvan af?**

Ehm.. Ja, wij hebben vooral met de jeugdarts te maken binnen dit project en binnen het ZAT. Dus het zorgadviesteam, daar zit de jeugdarts ook in. Dus dat zijn twee taken waar we veel mee te maken hebben. Dan hebben we ook vaak te maken met S.Z. vanuit ‘zorg voor jeugd’ en dat zijn allemaal verschillende rollen. Dat M@ZL project is heel erg op ziekteverzuim gericht, bij het ZAT praten we over kinderen met multiproblematiek en dan vragen we op een andere manier advies. Van ‘goh, als we het hele plaatje van het kind zien, hoe kunnen we het kind weer op de rails krijgen’. Dat hoeft niet perse te zijn dat er dan ziekteverzuim is, maar dan kan toch wel vaak een arts adviseren. Bijvoorbeeld een meisje met drugsgebruik ofzo.. Dus dat is een andere rol. En wat S. doet met ‘zorg voor jeugd’ is dan het organiseren van een groot overleg en het voorzitten enzo, dat iedereen het verslag krijgt. Dus dat is een andere rol. Waar wij als school ook wel eens in het verleden gebruik van maakten, ik weet eigenlijk niet of we dan nog vaak doen en of het nog kan, wat we wel graag willen in ieder geval dat het kan. Maar dat doet de bovenbouwcoördinator vooral. Is meisjes die al zeer jong seksueel actief zijn en daardoor een beetje in de problemen of in paniek geraakt zijn, dat we dan snel contact proberen te zoeken met de jeugdarts. Dat deed S.G. (jeugdarts) heel erg goed, die belden wij, van ‘nou, we hebben er weer een’ en dan zei ze ‘nou, ik heb vanmiddag wel ergens tijd’. En die ging dan ook altijd, en dat zal een huisarts niet zo gauw doen.. een huisarts zegt ‘nou, hier, een morning-after pil en wegwezen’. Wat een aantal jeugdartsen goed deden en sommigen wat minder, om daar iets tussendoor te plannen, maar dat die echt het gesprek aan ging met zo’n meisjes van 13. Van ‘goh, hoe sta je nou in seksualiteit en hoe ga je daar mee om’. En dat die kinderen dat toch altijd wel echt als heel prettig hebben ervaren, ik bedoel, die hadden toch een beetje een ‘nare ervaring’. Het seksuele gedeelte hoefde niet naar te zijn, maar in ieder geval wat erna kwam van ‘oei, nu ben ik misschien wel zwanger of heb ik misschien wel een hele enge ziekte eraan over gehouden’. En dat de jeugdarts daar toch op een andere manier gesprekken over heeft dan een huisarts. En dat vinden wij heel prettig en belangrijk dat dat ook gewoon kan.

**Maar dat doen jullie nu niet meer?**

Ik heb de laatste tijd niet zo vaak bij de hand gehad. Of het in de bovenbouw.. We proberen dat ook altijd niet zo groot te maken in de school. Niet de hele school hoeft altijd te weten van.. Maar als het voorkomt, proberen we het altijd wel ja. Liever dan bij een huisarts.

**En jullie hebben het voordeel dat het vlakbij is hè, ze hoeven maar over te steken en daar zit de GGD. Dat is wel een groot voordeel.**

Ja, in dit soort gevallen is dat echt een groot voordeel.

**Vinden de gesprekken dan op school plaats of daar?**

Daar bij de GGD. Maar dat kan heel gemakkelijk. Een belletje en dan ‘nou het kan nu wel, ik heb nu tijd’. Dan konden ze daar meteen op dezelfde dag nog terecht.

**Dus omdat het zo dichtbij is, is het contact ook heel goed? Dat is wel een voordeel.**

Ja, zeker.

Wat weet ik nog meer van de jeugdgezondheidszorg…

Ja, wat het laatste jaar ook een beetje op de achtergrond is verdwenen, maar wel belangrijk is, is voorlichting. Nu was er weer een aanbod, ook rondom seksualiteit. We zijn wel aan het kijken hoe we dat in het programma kunnen passen. Dat zie ik ook wel als een taak van de jeugdgezondheidszorg.

**En periodieke onderzoeken in de 2^e^ klas? Wat weet je daarvan?**

Dat vind ik echt…. We probeerden het voorheen altijd te koppelen aan dat ziekteverzuim. Als ze dan daar geweest waren, zorgden ze wel dat ze op de een of andere manier niet nog een keer hoefden te komen. Of als ze toevallig aangemeld werden rondom dezelfde tijd. Daar zag ik niet zo heel erg de meerwaarde van in. Heel af en toe kwam er dan eens uit van ‘dit kind hoort eigenlijk niet zo goed’. Ja, dat kwam er dan wel eens uit, wat niemand wist. Dat vond ik dan ook wel heel bijzonder, dat een kind het ook niet wist dat hij niet goed hoorde, en de ouders niet en wij niet. Maar goed, dat kwam er dan heel af en toe… Dat waren de enige dingen die eruit kwamen waar iemand iets aan had, naar mijn gevoel, van dat oren en ogen nog eens goed bekeken werden. Verder kwam daar, het kostte heel veel tijd, het was hier ook een heel gedoe met al die kinderen die.. Want het was altijd maar heel kort. In een lesuur gingen er 5 of 6 kinderen zo heen en weer. Ik zag daar niet zo heel veel meerwaarde van in, nee. Dus.

**En denk je dat je dan vanuit M@ZL meer kan bereiken?**

Nou ja, als mensen daar naartoe gaan en het serieus nemen, dan wel. Kijk, dat periodiek onderzoek was natuurlijk vooral heel erg ook gewoon om ´in kaart te brengen´, denk ik. Van ´hoe gaat het met de jeugd´. En misschien ook wat preventiefs, dat weet ik eigenlijk helemaal niet. Maar bij zorg is toch wel vaak… Je hoeft eigenlijk vind ik pas zorg te bieden als er zorg is. Heel veel van die kinderen hadden wij dan geen enkele zorg over, ouders ook niet, het ging hartstikke goed. Ja, moet je daar dan zoveel tijd en mensen op zetten. Terwijl er ook wel goede dingen uit zijn gekomen, hoor. Maar dat is uit alles natuurlijk. Alleen de vraag is of dat het meest effectieve is. En ook daar gingen heel veel kinderen niet naartoe, volgens mij, toch?

**Nee, het is op zich vrijblijvend.**

En wat ik daar ook van vond. Daar mochten kinderen ook alleen naartoe, zonder ouders. Ik vind het nu wel goed van M@ZL.. En dat was bij dat oude project ook, daar mochten kinderen ook alleen naartoe zonder ouders. Ik vind het nu bij M@ZL heel goed dat ouders er echt heel actief in betrokken worden.

**Weet je iets over de opkomst als jullie verwijzen naar de jeugdarts? Hoeveel ouders met kinderen dan ook gaan?**

Nee, ik hou dat niet procentueel ofzo bij. Dat doet Yvonne denk ik (lacht).

**Waarom vind je het zo belangrijk dat ouders erbij betrokken worden?**

*Nou, het zijn de ouders die hun kind ziekmelden, sowieso. Ik vind het ook belangrijk om de zorg te delen als het niet goed gaat met een kind. En als wij dat signaleren, vind ik het ook heel belangrijk dat de ouders daar toch een rol in hebben. S_2b_* Het heeft soms ook wel een nadeel dat kinderen net ietsje minder open zijn, denk ik, als hun ouders erbij zitten. Want een kind, kijk, als wij zien, een van die vakjes om aan te strepen is ‘leefstijl gerelateerd’. Als wij zien van ‘nou, dit kind zit misschien toch wel eens af en toe teveel te blowen of zit heel de nacht te gamen, terwijl ouders…’. Dat zal een kind niet zo gemakkelijk zeggen als ouders erbij zitten. Aan de andere kant, denk ik wel dat het echt belangrijk is dat ouders weten dat die, maar dat ze ook weten wat voor soort gesprek daar gevoerd wordt. Ik heb ook nooit begrepen dat die ouders, ik bedoel dan gaat het over de onderbouw, 12- en 13-jarige kinderen, dat ouders daar niet mee naartoe gingen. Dat heb ik nooit begrepen. Maar er zit aan allebei voor- en nadelen.

**Jullie hebben nu M@ZL compleet gekocht. Dat was het aanbod. Nu hebben we ook M@ZL-smal ontwikkeld (school bepaalt zelf criteria, gaat zelf eerst in gesprek en verwijst eventueel door naar de jeugdarts). Als je nu opnieuw zou mogen kiezen, waar zou je dan voor kiezen en waarom?**

Kijk, ik denk dat ik toch zou blijven bij hetgeen zoals we het nu doen. Ten eerste: wie moet dan die gesprekken gaan voeren? Als ik dat moet gaan doen, daar heb ik geen tijd voor. Dat is heel simpel. Want ik kan dat niet van 16 klassen. Als ik dat aan mentoren over moet laten, zonder mijn mentoren nou heel erg af te vallen, maar dan vraag ik me af in hoeverre die gesprekken goed gevoerd worden. Want je kunt een gesprek voeren op heel veel verschillende manieren en de een zal dat heel erg gemakkelijk afdoen en de ander zal daar toch…. Dus dat is een ding. *En het feit dat ik nu heel erg makkelijk aan ouders kan uitleggen ‘het geldt voor iedereen’, ‘iedereen moet dit gewoon en wij zijn geen arts’. S_2e_* Want dat vind ik ook moeilijk. Als je een gesprek met ouders aangaat over ziekteverzuim. Ja, ik ben geen arts. Dus als ouders zeggen ‘van ja…’, dan denk ik ‘het zal wel’. Dat vind ik toch heel moeilijk. Ik vind het heel moeilijk als wij op de stoel van de dokter gaan zitten. Dus ik zou denk ik toch voorstander zijn. Maar goed, ik ga niet over het geld. Ik weet dat ze bij de bovenbouw eerder voor zo’n constructie zouden kiezen. Is het mogelijk om daar een combinatie van te maken?

**Dat gaan we allemaal nog bekijken.**

Want dat zou denk ik…

**Eigenlijk denk ik dat een interventie het best werkt als je het zo goed mogelijk afstemt op wat de school wil. Alleen is mijn eerste reactie dan dat je dat heel goed zou moeten communiceren.**

Maar ja, ik denk, want nu is er in de bovenbouw veel weerstand, of veel, in ieder geval meer dan in de onderbouw. Van ‘goh, moet het allemaal’.

**Hoe komt dat toch? Waarom zijn ze daar zo sceptisch?**

Omdat hij niet ziet wat het oplevert, denk ik.

**En hoe komt dat?**

Weet ik niet.. Geen idee. Dan zou je met hem een afspraak moeten maken..

**Dat kan. Maar heeft dat met verleden van doen denk je?**

Ja, nee.. Nee, hij heeft dit altijd gehad. Vanaf dat we ermee gestart zijn al. Toen wij gestart zijn hadden we, met dat oude project, afgesproken dat we het in alle 4 leerjaren zouden doen. Toen kwam hij snel tot de conclusie dat het in klas 4 niet werkte, ik weet niet waarom. Dus toen hebben we gezegd: ‘dan doen we klas 1, 2 en 3’. En toen is hij eigenlijk ook vrij snel in 3 afgehaakt. Van ‘ja, we kennen al die kinderen wel en we weten wel wat er speelt’, laat maar. Dus dat is zo’n beetje zijn…

**We weten wat er speelt, we kennen ze al?**

Ja.. Terwijl ik, ik kijk daar toch wel anders tegen aan. Bij ons mogen ze dan als ze ziek geweest zijn een ‘blauw briefje’ halen als ze daardoor hun huiswerk niet hebben kunnen maken. Dan hebben ze dus toestemming om dat op een andere manier in te halen. En als ik dan zie dat kinderen, die hier in de 4^e^ klas zitten, dat ik die gewoon al 4 jaar lang, regelmatig, bij mij voor zo’n briefje zie komen. Dat zeg ik ook wel eens ‘goh, je bent wel vaak ziek hè?’. ‘Nee hoor, dat valt wel mee’. Nou, dan ga ik toch eens kijken in PARS en dan zie ik ‘ja, het is gewoon echt nog steeds heel veel’. Alleen ik kan me wel voorstellen, wat ik daarstraks ook al zei, dat je dan denkt ‘ja, we hebben er alles al aan gedaan, want in klas 1 is het een paar keer gemeld bij de GGD, zijn ouders geweest, en in klas 2’. Moet je die strijd dan aan blijven gaan? Dus dat gedeelte snap ik wel een beetje. Maar aan de andere kant, ik zie ook kinderen bij wie het dan toeneemt nog in klas 3 en klas 4. Die drempel wordt steeds lager voor kinderen.

**Vooral als er niets gebeurd natuurlijk..**

Ik weet niet of dat perse met ‘iets gebeuren’ te maken heeft, want ze zijn uit klas 1 en 2 gewend dat ze dan opgeroepen worden. Dus.. En wij vertellen niet in de klassen van ‘nou, in 3 en 4 loopt het hier een beetje moeizamer’. Dat weten de kinderen en ouders niet. Maar ja, kinderen merken wel van ‘ja, ze krijgen er toch niet echt grip op’. Vorig jaar hebben we, toen dat ook nog strenger was met de leerplichtambtenaar, hebben we een aantal kinderen ook doorgestuurd naar de leerplichtambtenaar op advies van de GGD. En dan hebben ze zelfs daar een gesprek gehad en ouders melden dan dat kind gerust 2 dagen later weer ziek voor een verkoudheid. En daar snap ik wel van, van die groep ouders, daar krijg je geen grip op. Dus op die kinderen ook niet. En dat verzuim zal alleen maar toenemen, want die kinderen zijn steeds gemakkelijker in thuis blijven denk ik. En dat komt van hun leven niet meer goed denk ik. Die gaan ook als ze straks werken, gemakkelijk zeggen van ‘och, ik blijf eens een dagje thuis’. Daar ben ik van overtuigd.

**Zijn dat juist de slimme kinderen, die het dan toch wel halen, ook al missen ze zo veel?**

Nee… Die zijn er wel, maar het is niet dat ik nu moet zeggen ‘dat is de groep’. Nee. Als ik nou aan een manneke moet denken, wat ik net zei van dat ‘blauw briefje’. Die zit nu in de 4^e^, heeft hier wel in de mavo/havo klas gezeten, maar heeft dat niet gehaald en het is nu nog maar de vraag of dat hij zijn diploma gaat halen dit jaar, want zo goed gaat het allemaal niet. Maar hij blijft wel heel gemakkelijk thuis.

**Je begon net even over de mentoren, dat er zoveel verschil is als de gesprekken moeten voeren? Maakt dat heel uit? Mentoren doen heel veel taken en er zit natuurlijk overal verschil in.**

*Ja, de ene mentor die is weer wat ‘vierkanter’ of ‘korter door de bocht’ dan de andere. En dat is met alle taken die je hebt natuurlijk. Dat is overal waar je werkt, de een neemt zijn taken heel erg serieus en gaat daar eens uitgebreid voor zitten. S_2c_*

*Maar ik denk ook dat sommige mentoren dan heel bang zijn, van ‘nu moet ik een heel naar gesprek aangaan, want u als ouder meldt uw kind te gemakkelijk ziek.. of dat is het idee wat wij hebben’. Zo voelen ouders dat toch wel een beetje. Dus ik denk dat sommige mentoren daar ook een beetje bang voor zijn en ook een beetje vermijdingsgedrag gaan vertonen zelf (lacht). Van ‘nou, dit ga ik maar even uit de weg’. ‘Ik zal het wel even tussen neus en lippen door noemen, maar ik ga er maar niet te diep op in, want dan wordt het gesprek ook niet te moeilijk’. S_2d_*

**Kunnen ze bijvoorbeeld daar nog in gestuurd of opgeleid worden? Een cursus?**

We hebben al heel veel trainingen en cursussen voor gespreksvaardigheden enzo gedaan.

**Voor mentoren?**

Ja, voor mentoren. Maar ziekteverzuim is toch wel echt iets moeilijks denk ik ook. Kijk, als een kind gespijbeld heeft of zich misdragen heeft, dat is natuurlijk echt heel erg onze taak. Zo van ‘jouw kind is onbehoorlijk geweest naar een collega van mij en ga daarover met u in gesprek, we willen niet meer dat het voorkomt’. Maar om als docent over ‘ziek zijn’.. Ik vraag me dat af. Ik vraag me ook af hoe het op die scholen gaat die dat nu doen. Hoe dat dan loopt, daar zou ik dan wel benieuwd naar zijn. Wie die gesprekken dan ook doet, of dat mentoren zijn of dat het zo gaat zoals je in gedachten hebt.

**Wat ik dan wel frappant vind is dat, als je vanuit de zorg voor een leerling denkt, dan kun je ook zeggen dat het ‘heel vreemd is dat mijn kind al zo vaak ziek gemeld is en die mentor belt niet eens een keer op en heeft daar een gesprek met mij over’.**

Maar dat bellen, dat doen we dus wel. Maar om nou echt rond de tafel.. Ik bedoel, bellen is dan nog even wat anders. Maar om echt face-to-face over iets ingewikkelds in gesprek te gaan.

**Het wordt toch als ingewikkeld ervaren denk jij?**

Ik denk het wel ja, ik heb het daar nooit met ze over gehad natuurlijk. Want we hebben het nu niet.

**Dat is interessant hè. Zijn het dan bijvoorbeeld ook de mentoren die zelf veel ziek zijn? Dan zeg ik misschien iets wat ik niet mag zeggen, maar..**

Nou, wij hebben hier niet zo heel veel ziekteverzuim. Dit jaar zeker niet. En als we ziekteverzuim hebben, is het meestal heel langdurig. Dus wij hebben hier niet zo heel veel collega’s die zelf heel vaak.. bij wie zelf die drempel laag ligt. Maar ik weet dus bijvoorbeeld niet hoe zij met hun eigen kinderen.. ik bedoel, hè.

**Nee, het is misschien toch wel eens interessant om eens wat mentoren bij elkaar te zetten en daar gewoon eens over te babbelen. Wat zijn nu de plussen en minnen van zo’n project. We hebben zelf het gevoel dat daar wel de grootste hobbel zit. Ja, want waarom ligt het zo gevoelig?**

Ja, ik moet heel eerlijk zeggen. Ik trek hier nou die kar en volgens mij doe ik dat dan best wel enthousiast enzo. Maar ik ben ook niet iemand die graag over ziek zijn praat.

**Nee, waarom niet?**

Ik ben voor mezelf al heel.. Ik hou niet van dokters (lacht). Ik ben ook nooit ziek, ik ga ook nooit naar een dokter. Ik heb nu een dokter en weet niet eens hoe die eruit ziet, of het een man of een vrouw is. Echt waar, mijn huisarts, ik weet het echt niet.

**Dat is ook een goed teken, toch?**

Ja, ik denk het wel. Ik ben ook wel gezond. 3 a 4 jaar terug kreeg mijn vader kanker, dat vind ik dan heel ingewikkeld. Het praten over ziek en dood enzo.. Dat ging ik ook uit de weg. Dat vond mijn vader dan ook wel prima, want heel veel mensen gingen daar dan heel erg in mee. Terwijl ik altijd probeerde van ‘nou, we hebben nog een half jaar ofzo, we gaan zorgen dat het leuk is’. Maar ik praat niet graag over ziek en dood. Dat past helemaal niet bij mij.

**Onbekend terrein?**

Wat dat betreft is het heel bijzonder denk ik dat ik dat hier zit te doen.

**Wat is je ervaring nu je er wel vaker over praat?**

Nou, het meeste wat ik erover praat is natuurlijk met ouders over kinderen. Meestal gaan die gesprekken over die kinderen die zo maar eens af en toe een paar dagen ziek zijn. Dat kan ik nog wel aan. Daar ben ik ook, ja, ik blijf zelf niet zo gemakkelijk thuis, eigenlijk nooit, ik ben nooit ziek, ik vind mezelf ook niet ziek genoeg om thuis te blijven. Als ik wat snotter, denk ik, dan kom ik toch gewoon naar school. Alhoewel ik wel merk, ik geef nu maar 6 uur les, dat is wel iets anders dan wanneer je een volledige week les geeft, als je de hele dag moet praten en je hoofd zit vol, omdat je grieperig bent, dan kan ik me voorstellen dat je zegt ‘dit trek ik vandaag even niet’. Terwijl ik hier een beetje zit te typen en dan doe ik wat administratieve dingen en dan kom ik die dag wel weer door. Dus dat maakt het ook wel gemakkelijker voor mij om ook altijd op school te zijn. Maar zo over griepjes enzo dat vind ik niet erg. Maar als het echt akeliger wordt, dan vind ik dat toch wel moeilijk.

We hebben hier een jongen op school, die is terminaal – dat klinkt zo, maar dat is het eigenlijk wel – dat is hij al 4 jaar. En hij zit nu in de 4^e^ klas, hij gaat nu eindexamen doen. En dat vind ik echt heel moeilijk.

**Dat is natuurlijk ook heel moeilijk.**

Ja..

**Zou u daar nog een rol zien voor de jeugdarts?**

Dat weet ik niet. Dat is ook voor iedereen allemaal heel persoonlijk. Ik ga het ook niet heel erg uit de weg hoor. Tenminste, ik zoek het niet op en als het op mijn pad komt, regel ik het ook wel. Bij die jongen, die heeft dan ook zo’n REC beschikking en iemand die daar dan regelmatig over komt praten en met die ouders hebben we natuurlijk heel veel gesprekken gehad. Dat zit ik ook te doen.

**Toch wel..**

Ja, dat hoort ook bij mijn taak. Dat hoort er gewoon bij. Maar daar moet ik wel voor mezelf wat in overwinnen. Het liefste denk ik dan ‘laat iemand anders dat maar even doen’.

**Praten over ziek zijn.**

Maar dat past niet bij iedereen.

**Al helemaal misschien niet bij mensen uit onderwijs?**

Weet ik niet..

**Waarom vinden we het toch zo moeilijk om met ouders en leerlingen over het ziekteverzuim van een kind te praten?**

Maar het raakt ook die andere persoonlijk.

**Hoe dan?**

Ja, wat ik merk is met ouders, als ik dan praat, dan merk ik weerstand omdat ze niet naar de GGD willen. Dat ouders vinden dat wij in hun privésfeer zitten te ‘roeren’ zal ik maar zeggen. Een beetje aan het bemoeien zijn. Dat wij ons bemoeien met dingen.. Kijk, als we een kind dat zich hier op school niet gedraagt of dat spijbelt of slechte cijfers haalt, daar mogen wij ons mee bemoeien, dat is onze expertise. En de rest dat regelen ouders wel met hun huisarts of de specialist of weet ik wat.. En zij vinden dat wij daar niet zoveel mee te maken hebben. En ‘als ik zeg dat mijn kind ziek is, dan is het ziek, punt’. En ik vind dan ook heel moeilijk. Want het is wel, ja, het is niet voor niets dat medische informatie ook altijd wordt afgeschermd. En ouders zijn, dat heb ik trouwens ook gemerkt, er zijn ook ouders die bang zijn, dat als ze naar de GGD moeten, dat dan daar gevraagd wordt naar iets in de trant van ‘kindermishandeling’. Of ze zijn ook heel erg bang voor allerlei dossiers die worden aangelegd. Dat zijn allemaal wel dingen die ik hoor. En ik kan dat niet zo goed bij die mensen wegnemen, zo’n gevoel. Ik doe wel mijn best van ‘daar gaat het helemaal niet om’.

**Dat zijn redenen waarom ze niet naar de GGD willen?**

Bij sommige ouders speelt dat ja.

**Zijn er nog meer redenen waarom ze niet naar de GGD willen?**

Omdat ze vinden dat het niet nodig is, ‘ik weet zelf wel of mijn kind naar school kan, ja of nee’. Dan denk ik dat zij vinden dat wij in hun privésfeer een beetje zitten te rommelen. En dat wij, misschien ook dat ze dan vinden, dat wij hun als ouder een beetje een ‘brevet van onvermogen’ geven. Zo van ‘ja, jij bent als ouder niet eens in staat om je kind te..’. Hè, dat denk ik. En dan worden ze boos. En zeggen ze ‘nee, ik weet heel goed, mijn kind is te ziek om naar school te gaan en daar hebben jullie niets mee te maken. Als ik zeg dat hij ziek is, dan is hij ziek.’ En dan moet je ook wel veel in huis hebben om door die weerstand heen te kunnen breken. Ik kan me voorstellen dat dat voor mentoren, die vinden dat toch moeilijk*. Het bespreken van ziekteverzuim roept weerstand op bij ouders. Dat maakt het moeilijk. S_2f_*

**Want het is prima dat ze hun kind ziek melden, alleen het is nu zo vaak dat je denkt dat het zorgelijk is.**

Sommige ouders vinden dat niet eens vaak.

**Dus dat zou ook al een reden kunnen zijn om daar eens een goed gesprek over te hebben. En te zeggen dat het wel vaak is.**

Ik had vorig jaar een meisje die al 7 keer ziek was gemeld, toen hadden we dat systeem nog niet zo mooi op orde. Ze was toen bij de arts geweest, daar was een advies uit gekomen dat er eigenlijk niet zoveel aan de hand was, ze moest gewoon naar school kunnen. Ze was daarna vervolgens weer een aantal keren ziek geweest en het was nog niets kerstmis. En toen hebben we gezegd ‘nou, dan gaan we eens richting leerplicht’. Toen ging ik daar met haar eerst over in gesprek van ‘dat zit er wel aan te komen, want je bent wel heel erg veel ziek’. Toen zei ze ‘ben ik nou zoveel ziek?’. Ja, je bent wel heel erg veel ziek geweest, nu al 12 keer en het is nog niet eens kerst. ‘Nee, zo vaak…’. Dat hebben ze ook helemaal niet in de gaten.

En ik merk de laatste tijd, want vorig jaar heb ik dat helemaal niet gemerkt, ik merk de laatste tijd dat veel ouders ook wel weerstand hebben door die vragenlijst. Want die vragenlijst slaat in hun gevoel niet op het ziektebeeld wat hun kind dan heeft. ‘Ik zie hier allerlei vragen, dat heeft helemaal niets te maken met dat mijn kind een paar keer de griep gehad heeft’.

**Dat geloof ik. Dat moet ik ook wel eens uitleggen als ik mensen na bel. Het gaat er veel meer om de kinderen die zich zo vaak ziek melden goed in beeld te krijgen. Dan begrijpen ze het wel en vullen ze het ook in. De arts legt het ook allemaal uit. Dan begrijpen ze wel dat wij met die vragenlijsten willen aantonen wat er aan de hand is, met een duidelijk instrument.**

Is het niet een idee om, ik weet niet of dat dan ook werkt hoor, maar.. nu krijgen ze die met de brief meegezonden. Van ‘je moet dan en dan op gesprek komen en dan zit er zo’n vragenlijst bij die naar hun gevoel niets te maken heeft met hun kind’. Kan die vragenlijst niet op de een of andere manier pas in het gesprek worden meegegeven? Zodat dat wat wordt toegelicht, voordat ze al die gegevens. Want die vragen komen bij die mensen wel echt binnen ook, ‘nou, ze willen nogal wat weten’.

**Ja, het is wel iets wat ik al eerder heb overwogen, maar nu ik dat zo hoor, denk ik dat we dat nog goed moeten heroverwegen. Het nadeel is dat het nogal wat vraagt om hem in te vullen en de jeugdarts vaak de ingevulde vragenlijst ook gebruikt in het gesprek. Dus als we het pas naderhand geven, is de angst ook van ons dat we daardoor veel minder terugkrijgen. Want nu laten ze vaak extra vragenlijsten invullen, terwijl de jeugdarts nog even bezig is en dan hebben we ze. Want je wil van zoveel mogelijk kinderen een beeld hebben. Maar ik heb dit inderdaad al eerder gehoord. We moeten daar maar eens heel goed over nadenken.**

Ik weet de oplossing ook niet, maar het is wel iets wat ik hoor inderdaad van ouders.

**Nou, misschien dat we dat vanmiddag nog eens aan de artsen kunnen terugkoppelen. Wat wij ook merken is dat in de informatiebrief staat dat het niet verplicht is om aan het onderzoek mee te doen en dat ze dat interpreteren als ‘het onderzoek bij de jeugdarts’. We moeten nagaan of die vragenlijst de reden is dat ouders vaak niet komen.**

Ja, ik weet het ook niet. Nogmaals, vorig jaar heb ik het geen enkele keer gehoord, want ik wist niet eens dat hij meegezonden werd.

**Toen was het ook nog niet. Dit is echt voor het wetenschappelijk onderzoek (uitleg). We willen onze hypothesen verifiëren met een instrument.**

**Hoe kijk je aan tegen wet- en regelgeving rondom ziekteverzuim? Voor ongeoorloofd verzuim is er een leerplichtambtenaar, een wet. Dat is per wet bepaald dat je moet registreren en doorzetten. Hoe zie jij zoiets met betrekking tot ziekteverzuim?**

Dat is natuurlijk toch heel moeilijk, want er zijn gewoon echt kinderen, net als zo’n manneke waarvan het een wonder is dat hij hier na 4 jaar nog steeds gewoon rondloopt en zijn eindexamen dit jaar gaat doen. Ja, dat is natuurlijk ook een hele grote uitzondering, maar.. Ik zou wel goed vinden als de wet daar wat strenger in werd. Net als, maar goed, dan blijft het nog steeds redelijk vrijblijvend denk ik.. Net als een bedrijfsarts, want dat is het eigenlijk, dit verhaal, dat het wel gevolgen heeft. Maar het blijft natuurlijk toch ook heel erg moeilijk om daar, denk ik, ik weet niet of dat in een wet te vangen is.

**Nou, parallel aan de bedrijfsgeneeskunde, dat is wel door de wet geregeld. Een werkgever is verplicht om een arbo-dienst in dienst te hebben.**

Maar ja, of dan, iemand die heel vaak gewoon voor ‘iets niet zo ingewikkelds ziek blijft’, of dat nou hele grote gevolgen heeft, volgens mij ook niet uiteindelijk. Letterlijk gezien, denk ik. In de bedrijfsgeneeskunde. En dat is met kinderen natuurlijk nog veel moeilijker denk ik.

**Waarom is dat nog moeilijker bij kinderen?**

Ja, ze zijn leerplichtig, ik kan ze niet van school sturen. ‘Je bent te vaak ziek, dus ik stuur je van school’. Of ‘je bent nu al voor de 7^e^ keer ziek gemeld, weet je wat, ik kom je uit je bed halen’. Dat kan toch niet. Ik kan er weinig sancties tegenover zetten.

**Je kunt ze niet van school sturen..**

Nee, dat zou ik ook belachelijk vinden. Want je wil juist dat ze meer naar school komen.

**Daar zit wel een heel belangrijk punt.**

Als jij als bedrijf, en dat ligt ook een beetje aan hoe je bedrijf is vormgegeven en ook daar zitten beperkingen aan. Want je mag iemand niet ontslaan vanwege ziekte, dus. Maar je kunt wel zeggen ‘je bent zo vaak ziek, dus je krijgt je promotie niet, of weet ik wat’. Dat kunnen wij toch allemaal niet. Kinderen moeten naar school en als ze gewoon hun cijfers halen, gaan ze gewoon over en halen ze hun diploma. Dan kunnen wij niet zeggen ‘weet je wat, je bent zo vaak ziek geweest, dus je krijgt je diploma niet’. Dat kan toch niet.

**Nee, maar daar zit wel iets heel belangrijks.**

Want als je het in de wet gaat vastleggen, dan zit er ook een sanctie of een maatregel in ieder geval aan, als het niet gaat zoals je wil. Ja, dan is de enige sanctie of maatregel die je kan bedenken is dat je ouders gaat beboeten ‘u meldt uw kind te vaak ziek’. Ja…

**Dat is denk ik ook wel een complicerende factor.**

Maar wat ik, maar goed, dat is iets heel ingewikkelds. Een tijdje terug hadden wij hier vanuit het CJG een voorlichting over de wet op de privacy in relatie tot kinderen. Dat was heel interessant trouwens. Die vertelde ook, hier in Nederland is alles rondom jeugdzorg heel erg geproblematiseerd. Als jij naar bureau jeugdzorg moet of als de school aangeeft dat er zorgen zijn rondom een kind. Dan is dat een heel groot probleem. En in Scandinavië bijvoorbeeld is het veel meer geaccepteerd dat ouders zeggen ‘goh, ik wil hulp bij hoe ik met mijn kind verder moet’. En daarom is daar bijvoorbeeld ook alles rondom de jeugdhulpverlening en jeugdzorg veel laagdrempeliger en goedkoper. Maar hier vinden ouders een ‘brevet van onvermogen’ krijgen als de school zegt ‘nou, we willen toch dat hier eens naar gekeken wordt’.

**Goedkoper, hier is het ook gratis toch, hulp vanuit bureau jeugdzorg.**

Nee, maar dat wordt toch wel betaald door de staat.

**Ja, maar voor ouders..**

Ja, voor ouders. Maar voor de staat is het veel goedkoper. Want de hulp die hier geboden wordt, is heel specialistisch, dan is het al zo ver uit de hand gelopen, dat het heel specialistische hulp is. Terwijl je met bijvoorbeeld kleine opvoedcursussen ofzo vaak al heel veel kunt bereiken. En in Scandinavië is dat volgens die mevrouw, ik heb het niet onderzocht, maar zij had daar dan wel ervaring mee, is dat daar heel normaal. Eigenlijk is het daar heel raar als je geen bemoeienis hebt met mensen die in wat voor vorm dan ook bij jeugdzorg betrokken zijn.

**Er wordt vaak naar die landen gekeken. Ze hebben ook hele andere regels rondom ouderschapsverlof en dat soort zaken. Ze vinden het ook normaal dat een moeder het eerste jaar thuis is en niet hoeft te werken. Want je moet een band opbouwen met je kind. Ze investeren enorm in het ouderschap. Er is gewoon een hele andere kijk op ouderschap, op hoe je daarmee om gaat.**

En dat is hier, want dat is, als wij dan tegen ouders zeggen, want dat is het signaal dat we afgeven, ‘je meldt je kind eigenlijk te gemakkelijk ziek’. Ja, dan gaan ouders toch een beetje van ‘oh, dus jij vind dat ik het niet goed doe’.

**In de aanval..**

Ja. En ook nog dat ze er dan nog bij bedenken van ‘ja, misschien gaan ze dan wel vragen naar kindermishandeling, er wordt een dossier gemaakt’.

**Ze zien het niet als ‘fijn dat je belt, inderdaad, ik heb mijn kind een paar keer moeten ziek melden, fijn dat we die zorgen kunnen delen’. Zo zien ze het niet.**

Er zijn gelukkig ook gewoon mensen die zeggen ‘inderdaad, ik maak me ook zorgen’. Maar die ouders die je dus niet vangt met dit soort projecten, dat zijn ouders die er zo wel een beetje tegenaan kijken.

**Of moeten we gewoon blij zijn met de ouders die we wel vangen en hulp kunnen bieden?**

Maar ik denk ook wel dat staat of valt met wie en wat en hoe het wordt gedaan. Maar dat is altijd.

**Ik heb verschillende dingen langs horen komen, de jeugdarts, het CJG. Werkt dat allemaal samen of juist niet?**

We moesten pasgeleden een enquête voor CJG invullen en ik vind dat het CJG hier compleet gezichtloos is. Dat werkt hier in ieder geval niet goed naar mijn idee. Misschien voor hele kleine kinderen, dat dat dan laagdrempeliger is, maar wij hebben weinig contact met CJG. We horen er weinig van. Ik ben toen netjes geweest toen het geopend werd, ballonnen opgelaten enzo en daarna is het eigenlijk heel erg stil geworden. Tot we een paar weken terug ineens een enquête kregen, toen dacht ik ‘ja, ik kan hier nou niet heel positief over gaan zitten doen’. Terwijl we met de GGD wel, want die is nu wel partner in CJG, maar met de GGD hebben we wel goede contacten. En met Surplus, die verzorgen hier binnen school het SMW, die zijn ook partner van het CJG, hebben we ook goede contacten. Maar met CJG zelf, nee.

**Dat is overal heel verschillend.**

Ja, dat is ook hoe het vormgegeven is hè.

**Iedere gemeente doet dat weer op zijn eigen manier.**

Ik weet dat het samenwerkingsverband van de scholen, die willen eigenlijk dat bijvoorbeeld in het ZAT alleen een ‘super-CJG-er’ aanwezig is.

**Een ‘school-CJG-er’?**

Ja. Die dan in zijn eentje, terwijl wij eigenlijk heel graag willen dat daar en de SMW en de arts en de leerplicht.. dan heeft het meerwaarde. Naar ons gevoel. En gelukkig gaat de gemeente Oosterhout daar nog steeds niet in mee. Maar in Breda speelt dat wel.

**Ja, we hoorden het vanochtend. Alle SMW stop vanaf 1 juli en dat worden allemaal school-CJG-ers.**

Ja. Die zitten dan dus in hun eentje.. Wij hebben hier op school elke week zorgteamoverleg. Daar zitten dan de bovenbouwcoördinator en ik en nog 2 mensen die veel betrokken zijn bij de zorg hier in de school bij en de SMW. En iemand van de directie. We hebben elke week overleg. En dan 1 x in de 6 weken hebben we ZAT. Ja, als dat diezelfde persoon is, wat heeft dan het ZAT voor meerwaarde op ons zorgteam. Plus, bijvoorbeeld de politie heeft vaak informatie die zo’n CJG-er echt niet heeft. En bij ons zit de politie er wel bij. En die informatie ben je dan wel kwijt. En een leerplichtambtenaar heeft ook echt een hele andere pet op dan iemand van het CJG, toch? Maar het is wel iemand waar je heel veel mee te maken hebt als je het hebt over zorg voor kinderen.

**Hoe zie je dat in de toekomst? Gaat dat veranderen?**

Wij proberen het zo wel te houden zoals het nu is. En in die enquête bijvoorbeeld heb ik dat ook echt aangegeven. Dat wij het niet willen dat dat verandert. Maar ja, dat is natuurlijk ook een kostenverhaal. Want als de gemeente zegt ‘ja, wij gaan niet meer onze leerplichtambtenaar faciliteren om 1x in de 6 weken op al die scholen bij zo’n overleg te zitten’, dan is het jammer, maar dan houdt het op.

**Als je de ontwikkeling ziet, ‘zorg in en om de school’. Wat vind je van de uitspraak ‘wij willen als school geen zorginstelling worden’?**

Dat kun je wel willen, maar dat is niet reëel. Dat is onzin. Het is natuurlijk, straks komt passend onderwijs er ook nog aan, dus dan krijg je nog meer zorg op je bordje. We zijn geen zorginstelling, we zijn een school. Maar die zorg blijft wel gewoon een heel groot onderdeel van wat je doet. En die zorg is hier ook wel, want dat is dan wel iets wat ik wel zie. Ook die mentoren, vakdocenten, ja, dat stukje zorg is hier wel, vind ik, goed ingebed.

**Dat is ook een taak van de school? Een verantwoordelijkheid van de school?**

Ja, dat vind ik wel. Je moet zorgen dat het.. Kijk, het is niet reëel om te zeggen ‘daar doen we niet aan’. Want zo’n kind is natuurlijk een totaalplaatje. Als een kind niet goed in zijn vel zit of als er grote gezondheidsproblemen zijn of er is een ernstige, ingewikkelde stoornis. Dan kun je wel zeggen ‘daar doen we niets aan’, maar dan gaat het ook niet goed komen met zo’n kind. Dus wij proberen daar wel.. Zorginstelling gaat wel heel ver natuurlijk.

**Hoe ver wil je gaan als school?**

Nou spreek je me aan als ‘school’, maar ik zit hier maar in mijn eentje hè.

**Nou, hoe ver zou jij willen gaan? Je bent een belangrijk deel van deze school.**

Het is wel persoonlijk.

Ik vind dat wij al best wel ver gaan in wat wij doen voor kinderen. We hebben een flink aantal leerlingen met rugzakjes enzo en we proberen dan echt te kijken in hoeverre wij hier zorg kunnen bieden. Maar ik merk wel dat er ook een grens zit aan wat docenten aan kunnen. Op een gegeven moment, als je teveel zorgleerlingen in een klas hebt, dan gaan docenten ook zeggen van ‘ja, nu moet ik voor deze leerling een proefwerk met een speciaal programma voor dyslexie aanbieden’. Dat is een speciaal programma op de computer en die docenten hoeven daar niet zoveel aan te doen, maar dan moeten ze een proefwerk naar mij opsturen en ik zorg dat het klaargemaakt wordt, zodat het door dat programma kan worden ingelezen. Dus dat is 1 leerling. Een andere leerling is doof, dus die moet met luistervaardigheidsoefeningen… En weer een andere heeft ADHD, dus die moet ik eigenlijk zo weinig mogelijk prikkels in de omgeving. Ja, je moet best wel veel. En ouders, en dat is wel, ouders gaan steeds meer eisen dat we dat ook doen. Van ‘ja, maar hij heeft ADHD, dat kun je toch niet van hem verlangen..’. Ja, maar we zitten wel gewoon.. Wij zijn een reguliere school, er zitten 28 kinderen in een klas, maximaal 30. En met een groot gedeelte van die kinderen is iets.

**Over problematiseren gesproken.**

Ja. Nou, ik ben nogal actief op twitter en rondom het passend onderwijs, dat heb ik een tijdje in de gaten gehouden, ik ben daar ook op een gegeven moment mee gestopt. Aan de ene kant vind ik niet, ik bedoel, een kind met Asperger, dat is echt een probleem. En dan mag van mij de minister en Ton Elias van de VVD enzo, die mogen van mij niet zeggen dat die stickers zomaar op kinderen geplakt worden. En dat kinderen een REC beschikking krijgen, gewoon voor niets. Want daar gaan echt drama’s aan vooraf. Zeker REC4, dat is heel dramatisch voor ouders om die een te krijgen en voor kinderen ook. Je wordt half voor gek verklaard en eer dat je die stempel hebt, en die vragen ouders echt niet voor niets aan. Ik vind het heel erg hoe daar in de politiek over gepraat wordt, over dat soort kinderen. Maar ook over die zieke kinderen, als ik dan hoor, want ook REC3 staat heel erg onder druk. Als ik dan denk wat wij hier voor die kinderen allemaal doen om ze naar school te kunnen laten gaan. Ik bedoel, je zal het maar hebben, zo’n kind dat dus eigenlijk zit te wachten tot hij dood mag. Want daar hebben wij het dus hier met dat manneke over. Die is er eigenlijk.. ja. Je zal het maar meemaken als ouders, dat is toch een drama. En dan zitten ze in de politiek te doen alsof je een geldwolf bent, omdat je daar hulp bij wil hebben. Ja, daar snap ik helemaal niets van, daar word ik ook heel boos over.

Aan de andere kant zie ik wel, vooral bij dyslexie, we hebben hier een 1^ste^ klas mavo/havo en meer dan de helft van die kinderen heeft een dyslexie-verklaring. Dat klopt niet.

**Dat is wel heel veel.**

Dus daar zit wel een.. Maar met een dyslexie-verklaring valt ook wel wat te halen. Want dan krijg je op een andere manier, wordt je CITO voorgelezen, je krijgt tijdverlenging. Dus heel veel ouders kunnen daarmee toch wel hun kind op een iets hoger niveau krijgen dan ze eigenlijk aankunnen. Daar ben ik wel van overtuigd.

**En wie geeft die verklaring dan af?**

Een orthopedagoog. Maar ja, daar is ook een beetje een wildgroei in denk ik. Een dyslexie-verklaring is denk ik wel te koop.

**Ja, vanochtend hadden we een presentatie over een screening voor dyslexie en daar kwam een flinke spellingsachterstand uit en dat had niets met dyslexie te maken. Er is dan waarschijnlijk wat anders aan de hand. Het is een stempel of een ‘afkorting’, daar valt ook wel wat te halen. Dan krijg je wat ruimte, wat geld.**

En er zijn ook wel scholen, zoals LWOO, is dan ook binnen het vmbo, dan kun je als school geld krijgen. Wij zijn ook een school die LWOO-gelden krijgen. Daar kun je op verschillende manieren mee om gaan. Dus ik snap wel dat de politiek daarvan ziet dat er steeds meer geld naar al die zorgleerlingen gaat, maar om dan juist die groep die het aller kwetsbaarst is, de REC3 en REC4, om die zo ongelofelijk.. Die zetten ze echt heel erg negatief neer in de media, vind ik.

**Ik denk soms wel eens dat ze gewoon helemaal niet weten waarover ze praten.**

We hebben voor REC3 een meisje met de ziekte van Crohn, doet het hartstikke goed hier op school, maar heeft wel gewoon echt heel veel last daarmee. Heeft dus een REC3 beschikking gekregen om daar ondersteuning in te krijgen en wij krijgen dus extra geld om dat meisje extra te begeleiden. Dan denk ik, ja, moet je nou tegen ouders gaan zeggen ‘ja, dit is eigenlijk een beetje flauwekul wat jullie hier zitten te doen’.

**Je helpt wel zo’n kind op het regulier onderwijs, om die erdoorheen te halen.**

Een meisje met een spina bifida, zit in een rolstoel. Van dat geld kunnen wij hier.. Het invalidentoilet was niet goed ingericht voor haar, dat hebben wij aangepast. Dat moet toch kunnen.

**Dat is ook wel heel concreet dan. En mooi om dat te kunnen doen.**

Maar dat is dus allemaal geld wat straks op de tocht komt. Al die rugzakjes gaan weg. En vooral ook die ambulante begeleiding gaat weg. En dat is wel vaak voor ons, ik begin nu elke keer over dat manneke die terminaal is, maar die ambulant begeleider weet de wegen van een psycholoog die gespecialiseerd is in praten met kinderen met deze problematiek. Ik zou niet weten waar ik daarvoor moet zijn. Dan zijn ouders en het kind ook, die zijn heel blij dat zulke hulp geboden kan worden. En dat gaat straks allemaal minder worden. Dat vind ik jammer.

**Onder de paraplu van passend onderwijs.**

Ja, onder de paraplu van passend onderwijs. Ik snap niet dat niet heel Nederland heel erg hard aan het protesteren is, want het raakt alle ouders van kinderen. Want al die kinderen die nu op speciaal onderwijs zitten, daarvan moet een heel groot gedeelte gewoon het regulier onderwijs in. Waar ouders nu al wel eens zeggen ‘die van mij krijgt niet genoeg aandacht, omdat er, want dat horen we ook wel eens terug.. Er zitten zulke druktemakers in de klas en die hebben van alles en die van mij heeft niets, maar die heeft ook aandacht nodig’. Ja, natuurlijk heeft die ook aandacht nodig. Maar dat wordt allemaal erger. En dat ouders van kinderen die hulp nodig hebben, straks minder daar zelf een keuze in hebben. Want als een kind straks hier aangemeld wordt en wij zeggen ‘dit is eigenlijk problematiek die wij niet aankunnen’, dan geven wij het dossier aan het samenwerkingsverband en dan zeggen die ‘nou, hier is een school voor uw kind’. En dan moet je daar als ouder in mee. Ja, ik vind dat heel ver gaan. Kijk, ik vind het wel belangrijk dat wij moeten kunnen blijven zeggen ‘dit kunnen we niet’, want daar wordt niemand gelukkig van als wij hier problematiek binnenkrijgen waar we niets mee kunnen. **Als ik hoor wat jullie allemaal doen, kunnen jullie heel veel. Goed hoor.**

Ja, ik snap nooit dat andere scholen het niet kunnen eerlijk gezegd. Zoveel moeite is het niet, denk ik. (lacht) Als je een goed hart voor kinderen hebt, en dat hebben wij als school wel, dat vinden wij wel belangrijk. Wij vinden kinderen belangrijker dan de stof zal ik maar zeggen.

**Dat komt ook een paar keer naar voren als je vertelt.**

Maar ik denk dat het op veel meer scholen veel anders zou kunnen lopen, maar goed, daar heb ik geen invloed op.

**Vind je het terecht dat het van VSV-gelden betaald wordt?**

Nou, die link zie ik niet helemaal. Maar goed, als dat geld er dan is..

Kijk, VSV problematiek die speelt hier eigenlijk helemaal niet zo.

**Omdat?**

Kinderen zijn sowieso nog leerplichtig, dus daar zit toch nog de wet ook heel erg achter. En het speelt pas veel vaker op het MBO. Ja, dan wordt er gezegd, dat zeg ik ook wel eens tegen ouders hoor: ‘er is een link tussen ziekteverzuim en vroegtijdig schoolverlaten’, maar of die er echt is, ik weet dat eigenlijk niet. Dat zou jij dan kunnen onderzoeken denk ik.

Maar wij hebben hier denk ik, en dat is wel de kracht ook een beetje van onze school, want wij hebben ook heel weinig ongeoorloofd verzuim. De leerplichtambtenaar heeft hier om de andere week op maandag een spreekuur voor kinderen waarvan we denken ‘het gaat misschien uit de bocht vliegen’ en dit jaar is er pas 1 leerling geweest. Dat is zeker sinds we PARS hebben ook wel.

**De controle is groter?**

Ja, als een kind.. dat heb ik jou toen laten zien. Wij hebben, als ik les geef, moet ik via het systeem melden of alle kinderen die voor mijn neus horen te zitten, of die er zitten. Op het moment dat ik zeg ‘deze is er niet’, dan krijgen ze hiernaast *(bij de administratie)* een mailtje ‘hee, die zit niet in de les’. Dus die kunnen meteen naar ouders gaan bellen of uit gaan zoeken ‘weten we iets van dit kind’. Dat is af en toe ook wel.. Afgelopen vrijdag was het een beetje raar.

(Nog een anekdote met telefoontje naar ouders van kinderen die ziek waren, terwijl er al toestemming van directie was voor afwezigheid ivm glazen huis)

Toen bleken ze dus wel toestemming van de directie te hebben. Meestal zijn ouders dan helemaal niet boos, dan zeggen ze juist ‘wat fijn dat jullie het goed in de gaten houden’.

**Dat is toch zo, dat zeggen ze van het ziekteverzuim niet..**

Nou, meestal als zo’n ouder dan belt zegt hij wel ‘ik snap jullie zorg wel, maar het is niet nodig’. En later uit het gesprek blijkt dan eigenlijk dat ze het echt onzin vinden. Ze beginnen wel met ‘ik snap jullie zorg wel’, maar ondertussen is de onderliggende boodschap dat ze vinden dat wij een beetje flauw doen.

**Heb je nog iets wat je kwijt wil, ons mee wil geven bij het onderzoek?**

Nou, dat van die vragenlijst wilde ik gezegd hebben. Verder.. nee.

**Interview 8. M@ZL onderzoek Datum: 23-01-2012**

**Aanwezig: Yvonne Vanneste (onderzoeker), Marlou van de Loo (semi-arts)**

**Directeur school 8 / P_8_**

**Variant: second route**

**Algemene gegevens**

Het gesprek vindt plaats op school, in de kamer van de directeur. Hij is erg vriendelijk, geeft wel aan dat hij beperkt de tijd heeft, omdat hij dan naar een volgende afspraak moet. Hij heeft het gesprek goed voorbereid en rondgevraagd bij de leerling-coördinatoren wat er speelt. In het interview komt duidelijk naar voren dat hij goed over het probleem heeft nagedacht. Ondanks de ‘tijdsdruk’ verloopt het gesprek rustig en neemt hij de tijd om zijn antwoorden te verduidelijken. Er komen nog steeds nieuwe meningen/ervaringen/gedachten naar boven uit het interview die bruikbaar zijn voor het onderzoek.

**Verslag interview**

**Vragen/opmerkingen door interviewster dikgedrukt**

Antwoorden/opmerkingen door geïnterviewde in normale opmaak

**U bent gestart met M@ZL, wat was voor u de reden om dat te doen? Wat heeft u daarbij overwogen?**

Ik heb hieraan meegedaan, omdat ik vind dat in ieder geval bij een aantal leerlingen, en zeker bij een aantal specifieke leerling-groepen, het schoolverzuim groot is. Te groot is. En omdat er niet meer de vanzelfsprekende ‘controle’ of ‘aansturing’ is vanuit een gezinssituatie, waarbij er gezegd wordt ‘kom, hup, naar school, morgen is het weer over’.

**U zegt dat het schoolverzuim bij een aantal leerlingen zo groot is. Viel dat op? Hebben jullie dat geregistreerd en kwam dat naar voren?**

Wij registreren al het ziekteverzuim, alle afwezigheid, daar zijn we heel kien op. En dan blijkt dat in een aantal situaties, leerlingen zich erg gemakkelijk ziek melden. Maar bijvoorbeeld ook met mededelingen komen, waardoor ze niet aan de gymles mee kunnen doen. Met name meisjes, allochtone meisjes, vertonen nog al eens dit gedrag, waarbij ze blijkbaar volledig worden gesteund door ouders. Nou, dat vonden wij aanleiding genoeg om met dit onderzoek mee te doen. Want hier zou, door een wat andere aanpak, zou winst geboekt kunnen worden.

**En waar denkt u dan aan? Winst?**

Als je er aandacht aan besteedt, dan wordt het minder. Dan wordt het gegarandeerd minder. Als je er geen aandacht aan besteedt, blijft het hetzelfde of wordt het erger.

**Hoe ging dat dan voor M@ZL?**

Niet op deze manier. Althans, veel minder. We hebben altijd veel contact gehad met een schoolarts. Omdat wij een school zijn voor praktijkonderwijs, vmbo. De GGD is altijd vertegenwoordigd in de ZAT’s. Noem het maar, we zijn ook vanuit de historie, een school voor speciaal onderwijs en nu weliswaar geen school meer voor speciaal onderwijs, maar wel met heel veel leerlingen met hulpvragen op divers gebied in huis. Dus onze lijn naar de schoolarts is altijd wel heel klein geweest en die schakelden wij ook wel in. Maar op deze specifieke problematiek zou dit ‘een’ aanpak, zou dit efficiënt kunnen zijn. En dan moet er toch soms gedacht worden aan wat andere, minder conventionele, vormen van aanpak. Waarbij de sturingsmechanismen toch wat groter worden dan in het verleden. Ik weet dat de GGD al jaren bezig is om instrumenten te ontwikkelen om sneller risicoleerlingen in beeld te krijgen. Ja, die kun je aanwijzen op het moment dat ze vanaf het consultatiebureau, je kent de situaties. Daar is heel veel over te doen. Het tast van alles aan enzovoorts, nou, ik ben inmiddels, en dat is het voordeel als je wat ouder wordt. Ik ben al dat soort stadia van privacy en van stigmatisering al lang voorbij. Zo is de realiteit. Wat kun je beter doen, en dat vind ik wel reëel in deze tijd, dan dat je heel efficiënt je inzet pleegt. Je pleegt je inzet daar waar je de grootste winst kan boeken. Om het maar even zo te vertellen. En daar hoort een risicoprofiel bij. Je hebt de gegevens, je weet wat je kunt doen om interventies te plegen, los van het feit of dat uiteindelijk succesvol is of niet. Dat is een tweede. Maar inzet op die plek waar het het meest nodig is. Wij moeten wat af van het ‘hele softe gepamper’ en het ‘toestemming’.

**Harder aanpakken?**

Nou, nee, niet harder. In de zin van ‘de beuk erin’ of ‘zero tolerance’, daar heb ik het helemaal niet over. Maar ik zou wel wat meer daadkracht willen zien. En dat heeft niets met harder te maken. Je moet soms ter wille van het belang van een kind of een leerling of, en dat geldt in de situaties van een ouderpaar voor hun eigen kinderen ook, soms een stevige maatregel nemen. Die je pijn doet als je hem moet nemen, maar waarvan je eigenlijk, waarvan je de overtuiging hebt dat die op termijn beter is. Dat heeft niets met ‘hard’ te maken. Dat is misschien wel ‘heel zacht’. Omdat je uiteindelijk altijd het belang van leerlingen of kinderen of het belang van je eigen kinderen voor ogen houdt. Een wat zakelijkere benadering, daar zou ik, daar ben ik wel voorstander van. Een klein voorbeeldje om dat te illustreren in het kader van ziekteverzuim. We zijn bezig met het ontwikkelen van ideeën rondom een wat andere aanpak van onze techniekopleiding. Techniekopleidingen die hebben het overal hartstikke moeilijk en ik ben erover in gesprek geraakt met wat bedrijven die daar met mij over meedenken. En die zeiden ‘je moet dadelijk gewoon het volgende doen, jongens’. Kinderen in een omgeving ontvangen waar ze serieus worden genomen enzovoorts, net zoals je ook volwassen mensen benadert. Weliswaar op hun eigen niveau. Als daar iemand, kinderen zijn van 8.30 tot 16.30 op school, net zoals iemand anders van 8.30 tot 16.30 werkt en dan heeft hij alles gedaan wat hij die dag moet doen. En als hij zich ziek meldt, dan staat er in de loop van de ochtend iemand van de arbodienst, komt even langs, drukt op de bel, niet alleen maar om te controleren, maar ook..

Dat vond ik een hele frisse benadering. Ik dacht ‘ja, waarom zouden we dat niet doen’.

**U zegt ‘niet alleen om te controleren, maar ook…’**

Bij zo’n benadering hoort ook belangstelling voor hetgeen er aan de hand is. En hoe een school of een bedrijf hier in dit geval, er iets voor zou kunnen betekenen. En tevens ook controle, daar is niets mis mee. Want je helpt daarmee ook structuur te brengen in een bestaan. En dat is weer gekoppeld aan dit hele traject. Het gebrek aan aansturing in gezinnen, het gebrek aan structuur in gezinnen, de vrijheid die veel leerlingen krijgen, jongeren krijgen. Helemaal zelfstandig hun grenzen bepalen. Dat vind ik een slechte ontwikkeling.

**Dat ziet u gebeuren de laatste jaren?**

Ja, daar zijn die jongeren niet mee gediend. Dat is moeilijk, die krijgen daar last van als ze in systemen moeten functioneren als ze volwassen zijn. Ik zie dat wel gebeuren ja. De ontwrichte gezinnen, veel echtscheidingen, veel gebroken gezinnen die het allemaal voor de kinderen geweldig geregeld hebben en op school zien we hier de problemen die dat met zich meebrengt. Het gebrek aan structuur, het gebrek aan veiligheid, het gebrek aan een plek waar je altijd terecht kunt. Dat is een groot gemis, als er dat niet is.

**Dat is steeds meer zo?**

Ik denk dat het wel meer is dan dat het vroeger het geval was, ja.

**Is M@ZL daar de oplossing voor?**

Nee, M@ZL is daar niet de oplossing voor. Maar een aanpak zoals M@ZL, waarbij je snel actie onderneemt, helpt wel om de nadelige effecten zo klein mogelijk te maken. Noem het een soort opvoedingsondersteuning. Heel gericht op specifieke situaties. En dan kan het iets bijdragen ja. Als dat allemaal goed functioneert enzovoorts. Maar dan kunnen daar dit soort initiatieven aan bijdragen.

**Hoe loopt M@ZL nu binnen de school?**

De ervaringen zijn wisselend. Ik noem even wat ik hier heb gekregen. Twee van mijn afdelingsdirecteuren hebben de ervaring dat er na de aanmelding een slechte terugkoppeling is. Die ervaren weinig toegevoegde waarde. Wel als ze zelf meer werk moeten doen. Dat er ook in een aantal gevallen geen verdere actie is ondernomen. Een andere reactie van een andere locatie hier, is wel tevreden over de goede terugkoppeling. Dus de reacties zijn wisselend.

**Is dat dan echt puur de terugkoppeling zelf of is dat hetgeen dat teruggekoppeld wordt?**

Ja, ik denk dat, ik ga ervan uit dat het om het laatste gaat. Dat zo’n zaak wordt opgepakt, want wij zijn serieus in datgene wat we doen. We volgen het protocol zal ik maar zeggen, we voeren het gesprek op school en de terugkoppeling is, want dan komt er op enig moment een gesprek tussen ouders en de schoolarts, en het traject daarna…. En of het dan, welk element precies.. Ja, dan moet u dat met een enquêteformulier navragen bij de mensen die er daadwerkelijk mee werken. Want dan gaat het echt om de specialistische inhoud, de uitvoering. Nogmaals, daar heb ik alleen maar een globaal idee van.

**We hebben daar vanmiddag ook een bijeenkomst over.**

Blijkbaar komen deze zaken.. Zit er een verschil in beleving. En de mensen die dit hebben aangegeven, dat zijn twee hele, een ervan zit er vanmiddag bij, dat zijn twee hele bewogen mensen die echt ‘niet mopperen vanwege het mopperen’.

Toch te weinig toegevoegde waarde, bij wat ze zelf al deden. Dat kan dus ook iets zeggen over wat ze vooraf zelf al deden. Bijvoorbeeld, we hebben een afdeling praktijkonderwijs, daar zijn we heel alert. Daar worden heel snel ouders opgeroepen, daar wordt heel snel contact opgenomen met ouders. Dus het is moeilijk om dat in zijn algemeenheid te zeggen, omdat de toegevoegde waarde altijd uitgaat van de situatie zoals die voorheen was. Nou, die beginsituatie wisselt per school, wisselt in ons geval per locatie en per afdeling. Dat is bij de ‘zorg en welzijn’ anders dan bij de ‘techniek’. Dus er zitten nogal wat variabelen bij, die het gevoel ‘of het iets toevoegt’ beïnvloeden. Dat maakt het onderzoekstechnisch heel ingewikkeld.

**Daar heeft u gelijk in.**

**Wat is nu, u zegt ‘ze leveren zelf al heel veel zorg’, wat is nu de meerwaarde van de arts?**

Ehm… De toegevoegde waarde.. Laat ik het in eerste instantie maar heel plat vertellen. Het feit dat er een gesprek met de arts volgt, geeft een aantal ouders een steuntje in de rug om zelf daar ook wat alerter in te zijn. Je mag ook zeggen, anders geformuleerd, het klinkt wat ‘officiëler’ dan alleen een gesprek met de school. Van ‘hee, er is blijkbaar toch wel iets aan de hand’. Dat kunnen twee zaken zijn: ‘moet ik me zorgen maken’, dan wel ‘goh, ik moet daar zelf ook iets in betekenen’. De invloed die een arts kan hebben op ouders, is groter dan die de school kan hebben op ouders.

Je hebt zo vaak contacten met ouders, je voert zo vaak gesprekken. Je blijft proberen om als school, zeker als het gaat om lastige situaties of om situaties waarin we zorg hebben, de kracht van ouders en school met elkaar te verbinden. Dit is een unieke, niet zo vaak voorkomende, combinatie. Waarbij een derde, van een onafhankelijke statuur… de schoolarts heeft bij zich het beeld van deskundige, maar ook van onafhankelijk deskundige. Die toch voor een spiegel naar ouders kan zorgen. Die kan ook de situatie beter inschatten. Maar er zit ook iets anders in. En dat heeft alles te maken met onze eigen beeldvorming. *Op enig moment interpreteren wij gedrag van leerlingen, daar verbinden wij conclusies aan. En die conclusies, dat beeld, dat wordt doorgaans alleen maar steviger. Want wij noteren en wij zien vooral wat we willen zien. Wat past in dat eerder opgebouwde beeld. Noem het maar ‘de tunnelvisie’. En een kind waarvan op enig moment het beeld in ontstaan dat ze ‘faket’, dat wordt moeilijk om dat te doorbreken. P_8b_* En ook in dat opzicht kan een schoolarts als onafhankelijke persoon, die zonder die voorgeschiedenis een situatie bekijkt, van grote waarde zijn.

**U zegt nu ‘een onafhankelijk persoon’, maar dat moet wel een arts zijn? Of kan dat ook gewoon iemand van buiten de school zijn? Wat is de meerwaarde van de arts, van de medische achtergrond?**

Als ik nou van een kind het idee heb dat zij zich vaak ziek meldt en op school zie ik eigenlijk niets, kortom het is ‘flauwekul’ en dat beeld gaat zich bij mij vastzetten. ‘Weer ziek, ik wil naar huis’. Ik ben ervan overtuigd dat zij langzamerhand in mijn beeldvorming iemand is die allerlei smoesjes bedenkt om maar naar huis te gaan of thuis te blijven. Als dan de ‘buurman’ mij komt vertellen dat er toch echt iets aan de hand is met die leerling, raak ik daar niet zo van onder de indruk. Hij zal mijn beeld niet snel doen veranderen. ‘En de tante van Jantje ook niet’. Maar een schoolarts, met haar/zijn expertise, op dat gebied, die zal ik toch wel snel erkennen.

**Maakt het verschil over welke leerrichting of welk leerjaar de leerling zit? In de aanpak of noodzaak om het aan te pakken?**

Tja…. (lang stil)

Daar zit voor mij een preventief en een curatief element in. Het curatieve element helpt, denk ik, het snelst als leerlingen wat ouder zijn. 14/15/16 jaar oud. Omdat ze ook dan dominant ander gedrag gaan vertonen. Zeker ten aanzien van langdurige afwezigheid. Preventief, dan zit je toch op de jongere leeftijd, maar dat begint volgens mij al in groep 7 en 8 van de basisschool.

Omdat dan, althans dat schat ik in, de fase van beïnvloeding tussen je 10^e^ en 14^e^ wat groter is dan wanneer ze eenmaal 15 of 16 jaar zijn.

**Dat ‘wij’ ze nog kunnen beïnvloeden?**

Wij? Ik denk ouders… Dat de rol van ouders daar in ieder geval nog, al is het maar een kleine beweging, kan dan zijn effect nog hebben. Dat geldt voor de school precies hetzelfde.

Het meest acuut wordt volgens mij nu ingepast, en zo zou ik het zelf ook doen, bij de leerlingen die wat ouder zijn. Omdat die vaker dit gedrag van veelvuldig ziekmelden laten zien.

**De hogere leerjaren.**

Ik weet niet of het zo is hè, want ik heb geen enkel lijstje gezien van hoeveel leerlingen er aangemeld zijn, maar gevoelsmatig.. Als ik dit nu zou moeten doen, ja, dan zou ik toch deze interventie sneller inzetten bij wat oudere leerlingen. Terwijl het effect misschien wel groter zou zijn als je dat op jongere leeftijd doet.

**En in het laatste jaar, het examenjaar. Als je ook denkt aan vroegtijdig schoolverlaten?**

Ja, daar zie ik niet zoveel heil in, als ik eerlijk moet zijn. Nee. *Want als het gaat om vroegtijdig schoolverlaten, daar spelen zoveel factoren een rol. P_8c_* Dat een interventie hier, ik zou bijna zeggen.. Ik zal het voorzichtig formuleren. Ik vraag me af wat een dergelijke interventie zou kunnen bijdragen. Omdat daar hele andere zaken een rol spelen in zo’n laatste jaar. In zo’n laatste jaar vindt een oriëntatie plaats op een nieuwe toekomst. Van doorslaggevende betekenis is of een leerling een keuze maakt die uiteindelijk bij zijn/haar affiniteit past. Daar speelt een ‘peer group’ een zeer belangrijke rol in. Daar speelt de ontvangende omgeving een rol in. Allemaal zaken die bepalen of een leerling afhaakt of niet. Percentages laten zien dat het een stevig probleem is, maar waar een geïsoleerde interventie van de schoolarts weinig mee kan doen. Want ook hier spelen ouders nauwelijks meer een rol. Ouders hebben geen invloed meer op die leeftijd. 16 / 17 jaar, dan bepalen de leerlingen zelf. Dus er zijn zoveel factoren die een rol spelen of een leerling afhaakt of blijft zitten of zo’n opleiding.. Nee, ik geloof niet dat dit een grote toegevoegde waarde zou hebben. Maar dat is allemaal aanname hè, het is niet wetenschappelijk bewezen. Maar gevoelsmatig zou ik hier weinig in investeren.

**En ziet u wel een link tussen het ziekteverzuim en het vroegtijdig schoolverlaten?**

*Veelvuldig schoolverzuim heeft alles te maken met de situatie waarin een kind zich bevindt. De thuissituatie is daar voor mijn gevoel dominant in. P_8a_* De thuissituatie, de keuze of die al of niet ‘de straat als een nieuw thuis omarmt’. De stabiliteit die het kind kan hebben of ervaren in zijn eigen omgeving of op school. Het gaat om die drie gebieden. Thuis, op straat, school. En op straat noem ik dan ook ‘het gebied van de voetbalvereniging en dat soort zaken’. De school als instituut is niet sterk genoeg om leerlingen te binden. Er zijn maar heel weinig jongeren, weinig kinderen en toch zeker in onze categorie, die in de huidige systematiek van het onderwijs ‘vrijwillig en met heel veel enthousiasme naar een school gaan’. Waar ze wél voor komen is, omdat ze hier leeftijdsgenoten ontmoeten. Maar de school als leerinstituut, als onderwijsinstituut is te weinig bindend, te weinig aantrekkelijk. Dat geldt voor deze vorm, geldt doorgaans ook voor vervolgopleidingen. Intrinsieke motivatie omdat mensen willen leren, dat is …. Scholen kunnen dat heel moeilijk zelf doen. Dus, met name de positie die jongeren hebben vanuit hun thuisomgeving, waarbij het vanzelfsprekend is dat je naar school gaat, vanzelfsprekend is dat je je best doet, dat er aandacht voor is enzovoorts. Bepaal daar de succesfactoren. Als er al sprake is van langdurig schoolverzuim of veelvuldig schoolverzuim in de middelbare school periode, is de kans verschrikkelijk groot dat het misloopt in het vervolgtraject. Want daar wordt de controle nog kleiner. Ik denk dat we een aantal van dat soort kinderen gewoon kwijt zijn.

**Een klein groepje…**

Ik denk niet dat het zo’n klein groepje is. Ik denk dat wij in die periode veel jeugdigen missen. Die uiteindelijk zich niet willen of kunnen conformeren aan de structuur van school of aan de structuur van werk of aan de structuur van een gezin. En die maken andere keuzes. Dat wil niet zeggen dat die niet ooit weer wel eens ergens terecht kunnen, dat is iets anders. Maar voor het ‘reguliere lijntje’ van ‘je gaat naar een middelbare school, dan een vervolgopleiding en dan ga je werken’, dat gaat bij deze leerlingen niet zo.

Ja, en daar, afijn, de ‘taskforce jeugdwerkloosheid’ is daar een aantal jaren geleden heel intensief mee bezig geweest, om met name deze groep voortijdig schoolverlaters toch nog erbij te trekken. Dat was ook op een mislukking uitgelopen. De conclusie is: je bent te laat als je het wil doen als ze 15 of 16 jaar zijn. Terug, je moet de lijn proberen te leggen als ze jonger zijn. En als je er dan voor zorgt of voor kunt zorgen dat je die structuur, die stabiliteit, dat perspectief kunt bieden… En dat kan zijn, een school, werk of thuis, een vervangende situatie. Dan heb je kans op succes.

**U noemde net die thuissituatie en het ziekteverzuim. Uiteindelijk melden de ouders hun kind ziek toch?**

Ja, doorgaans wel. Soms doen leerlingen dat zelf, proberen ze de stem van ouders of het handschrift van ouders te imiteren. Zoals we dat uit onze eigen jeugd ook kennen. Maar ouders melden ziek ja.

**Wie is er nu de probleemeigenaar van het ziekteverzuim?**

Er is maar één probleemeigenaar, dat zijn de ouders.

**En waarom?**

Omdat ouders in eerste instantie verantwoordelijk zijn voor het grootbrengen van hun kind. En het kind voor te bereiden op een perspectiefvolle toekomst. Het is heel verleidelijk om de school overal probleemeigenaar van te maken. De school is probleemeigenaar van te dikke kinderen, van voortijdig schoolverlaten, van …. Ik zie dat anders. Als een kind te dik is, ligt het in eerste instantie aan ouders. Of er zijn medische factoren, dat is… We willen daar best iets mee doen, aan meewerken, maar wij zijn geen eigenaar van het probleem. Als leerlingen afhaken doordat ze andere keuzes maken, drugs gaan gebruiken of vinden dat ze perse met een mes naar school moeten komen, dan is dat een probleem van de leerling en de ouders. En niet van de school.

**Maar u zegt ‘de school speelt daar wel een rol in’.**

Een school speelt daar een rol in, een school zal meewerken. In ieder geval, onze school. Voor zover het kan, aan het oplossen van het probleem. Of aan het ondersteunen van diegenen die dat probleem op moeten lossen. Maar wij zijn geen probleemeigenaar. Nee.

Ik vind, kijk, onze school, ons type scholen, en voor onze school geldt dat zeker, het praktijkonderwijs / vmbo, wij zijn een verzameling geworden van maatschappelijke problematiek. Overal waar de maatschappij geen antwoord op weet, kwesties van de multiculturele samenleving, die vindt je in ‘bijna vergrote vorm’ terug in scholen als deze. Daarmee zijn wij nog geen probleemeigenaar. Uiteindelijk is de probleemeigenaar de maatschappij zelf.

**Ziet de politiek dat ook?**

De politiek is hierin niet geïnteresseerd. Nee..

De politiek is met andere dingen bezig. Het hele idee rondom passend onderwijs, ook zo’n.. het is een prachtige manier om sturingsmechanismen, financiële sturingsmechanismen te ontwikkelen, waardoor je controle houdt op de hoeveelheid geld die je ergens aan besteedt. Maar het gaat in de politiek niet om ‘het belang van onze doelgroep’. Die stellen electoraal niets voor, spelen geen enkele rol, schrijven ook geen dure brieven. Niet interessant. Dat staat in geen enkel verkiezingsprogramma, even voor de helderheid. Daar staan prachtige woorden.. ‘de zwakste’.. Maar.

De vraag is ook of dat dat kan, het kan wel, maar dan moeten er rigoureus andere keuzes worden gemaakt, die de overheid in de mondiale plek die ze innemen en in het maatschappelijk radarwerk. Je kunt niet zomaar ineens een hele andere beweging gaan maken als Nederlandse overheid of als politieke partij of wat dan ook.. Je moet mee in het radarwerk van het internationale proces. Maar deze doelgroep brengt niets, voegt ‘macro-economisch gezien’ weinig toe aan de samenleving. Kost eigenlijk alleen maar geld.

**Dat kan juist een reden zijn om er meer zorg en ondersteuning op te zetten.**

Daarom zegt het CPS ook keurig ‘het heeft allemaal niets uitgehaald’, ‘al dat extra geld in het onderwijs heeft niet geholpen, in de zorg heeft het niet geholpen’. ‘De CITO-score is niet beter - want dat is dan het criterium.. allemaal weggegooid geld’. En in deze tijd van bezuinigingen en van rationalisatie zijn dat sterke argumenten, vinden ze. En je schrijft, dat is misschien tegen de wetenschap, maar je kan elk onderzoek ‘naar de uitkomst toe schrijven’. En dat gebeurt dan ook massaal. De onderzoeker is ‘heel toevallig’, want Nederland valt van onderzoeken ondersteboven en altijd op het juiste moment, komen de juiste onderzoeken naar boven. Als je maar lang genoeg ‘googled’, dan heb je altijd wel ergens een onderzoek die jouw lijn of stelling onderschrijft. Ofwel bestrijdt, die zijn er ook, maar die heb je dan toevallig niet gezien. Dus ik ben daar niet zo.. Ik sta daar wat realistischer in. Ik vind wel dat we vooral alles moeten doen wat in onze macht ligt om voor deze groep iets te doen en daar hoort zoiets (M@ZL) bij, hoe klein het ook is. Dit maakt het verschil. Een prachtig praatje tijdens een politieke manifestatie doet niets, maar zoiets wel. Wij proberen nu te doen, om ook de techniek richting wat … Degenen met veelvuldig schoolverzuim, kijken we of we die op de een of andere manier naar een baan kunnen leiden. Maar naar een baan, waar werk ligt. Niet naar een baan die ze misschien wel graag zouden willen, want dat is even niet meer aan de orde. We hebben bijvoorbeeld in de techniek, ligt volop werk. ‘Daar heb ik een baan voor je, dat proberen we zo te organiseren’. Werk, inkomen, zorgt voor structuur. Dat is, vind ik, zo wezenlijk. Los van welke zorggelden, niets geen zorg, weg met die zorg. Niet ‘pamperen’ en ‘nathouden’, maar ‘doen’. Bij ons op school kiezen teveel leerlingen voor een opleiding, bijvoorbeeld in de economie, handel/adminstratie enzovoorts, met name allochtone leerlingen doen dat nogal. Waar absoluut geen enkel toekomstperspectief in zit, dat is weggegooid geld! Nou weet ik wel, mensen veranderen nog vaak van hun loopbaan, maar dan begin je al met niets. Dan begin je al perspectiefloos. Dat is een slecht begin. En dat doen wij allemaal.. Want iedereen mag kiezen.

**Iedereen mag doen wat hij leuk vindt.**

Zeker. En we weten dat daar, uit zo’n groep, daar komen de eerste tientallen vroegtijdige schoolverlaters. Dat doen we zelf! We creëren zelf onze eigen problematiek. En dan ben ik liever voor een hele pragmatische aanpak, van ‘nou, hoe kun je daar nou iets mee doen, hoe kun je dat nou beïnvloeden’. Zorg in de verengde zorg van ‘klein houden’ zal ik maar zeggen, daar zie ik absoluut helemaal niets in. Zorg moet voor mij altijd zijn juist ‘groter maken, sterker maken, weerbaar’. Want ‘je hebt het te doen in de wereld’. Jij hebt, met jouw kwaliteiten, met jouw karakter, jij hebt je plek te vinden, te bevechten in deze wereld. En dáár moet je zo goed mogelijk op toegerust worden. Niet door mensen in te pakken, daarom is voor mij ook niemand zielig. Deze school niet, deze leerlingen niet. Maar ze moeten het wel allemaal zelf doen.

**Ik heb nog een paar andere vragen.**

**Wat moet er hier op school nog gebeuren om M@ZL beter te laten lopen? Om het te implementeren binnen de school?**

Voorwaarde is dat er voldaan wordt aan de verwachtingen die mensen hebben. Als ik nu hoor ‘weinig toegevoegde waarde, slechte terugkoppeling’. Kijk, die toegevoegde waarde, daar zit en subjectief element in, omdat daar nog een andere variabele in kan zitten. Het is maar net wat je deed, of je vindt dat dat toegevoegde waarde heeft. Maar zaken als ‘geen terugkoppeling’, dat moet je wel zien te vermijden, want anders haken mensen heel snel af. Die afspraak heb ik ook gemaakt hier, want niemand zit te wachten op ‘extra werk’ en op ‘extra bureaucratie’. Na een jaar bepalen we wat we wel of niet doen. Als het helemaal niets toevoegt, dan stoppen we ermee. Dus, met name dat soort zaken: doe wat je belooft. Als het om die terugkoppeling gaat, dat zijn de makkelijkste zaken.

**Yvonne: ja, ik ga daar achteraan. Ik weet wel dat er op het praktijkonderwijs heel weinig opkomst is bij het consult van de jeugdarts.**

Ouders niet geweest, dat staat hier ook op mijn lijstje.

**Dat gaan we vanmiddag samen bekijken.**

Mensen moeten het idee hebben dat je iets doet met hun inzet.

En ook benoemen, je weet dan waar.. Kijk, als dit het punt is, neem dan contact met elkaar op!

Want de wil is er wel, even voor de duidelijkheid, want anders hadden we niet mee gedaan.

**Zijn er nog andere dingen die u denkt van ‘dit moet er nog gebeuren’?**

Ja, dat geldt voor ‘het project als initiatief’, geldt in ieder geval dat de terugkoppeling goed moet zijn. Daar mag het niet op mislopen. Ik vind, als je onderzoeker bent, dan mag het op een heleboel gebieden tegenvallen. Het kan niet opleveren wat je gedacht had. Maar het mag niet foutlopen op dit soort zaken. De elementaire basis, zal ik maar zeggen.

En voor de rest heb ik in brede zin een beeld geschetst van de manier waarop ik kijk naar zorg, begeleiding. Als onderdeel van dit project.

**Misschien nog een laatste vraag.**

**School heeft nu M@ZL-smal, gaat zelf eerst gesprek aan. We hebben ook variant waarbij leerlingen bij bepaalde criteria altijd naar de jeugdarts gaan. De school hoeft dan zelf geen verzuimgesprek aan te gaan met leerling en ouders. Hoe kijkt u daar tegenaan?**

Ja, ik ben daar wat ambivalent in. Uiteindelijk vind ik dat de school wel zijn rol heeft. Voor de school is het wel makkelijk, zo van ‘6 keer ziektemelding, hup, naar de schoolarts’. Dat is een, dat is iets wat mij.. Dan zeg ik ‘nee, hier wordt de positie van school, los van het feit dat een school geen probleemeigenaar is, dat blijft zo, maar we hebben wel onze verantwoordelijkheid vind ik’. Dat is 1. Het tweede wat ik vind is dat 6 keer een ziekmelding bij Pietje iets anders kan zijn dan 6 keer een ziekmelding bij Jantje. We hebben het nu gemerkt.. Dus daarom ben ik niet voor dit soort mechanismes. Wij hebben het hier op school gemerkt bij te laat komen. We hebben een prachtig administratiesysteem, daar bewaren we allemaal brieven in. Dan wordt er ook genoteerd hè.

Dus ‘bij 6 keer te laat, komt er zo’n pop-up schermpje dat je brief 1 moet versturen’. ‘Geweldig’. Daar zijn we van terug gekomen! Want dat systeem, je wordt op een gegeven moment geleid door het systeem, door ‘een systeem dat zegt 6 keer’. Er werden brieven gestuurd die eigenlijk helemaal niet verstuurd hadden moeten worden. En nu zijn we terug naar ‘er komt wel een waarschuwing, maar uiteindelijk bepaalt de teamleider naar wie een brief wordt gestuurd’. Dat is eigenlijk hetzelfde als wat daar gebeurt. Bovendien vind ik dat een gesprek met de ouders, het contact met de ouders, van wezenlijk belang is.

**Voor de school?**

Ja, niet alleen voor de school, maar ook voor de ouders zelf. Toch zeker om te benadrukken dat het in het belang is van het kind, van de ouders, dat het géén straf of controle is, maar dat je het beste met het kind voor hebt. Dat is de voorwaarde waarop de schoolarts het gesprek kan voeren, die is dan veel gunstiger dan wanneer het een soort ‘9 keer niet geweest, dan moet je voor straf naar de schoolarts’. Ik denk dat het effect van het gesprek - in de situatie wanneer er op school een gesprek aan vooraf is gegaan - naar mijn idee, veel groter. En ik vind, ook in deze tijd, efficiëntie, dat is wel iets waar je naar moet kijken.

Interview 9. School 4 / P_4_ en S_4_	Datum 25-1-2012.

**Aanwezig: Directeur en Zorgcoördinator, Interviewer (Yvonne) en onderzoeker (Marlou).**

Algemeen:

**Er is een prettige en ontspannen sfeer. Gedurende het gehele interview wordt er heel vaak instemmend ja gezegd naar elkaar, de directeur en de zorgcoördinator, toe.**

Kunnen jullie vertellen wat het nut van ziekteverzuimbegeleiding is?

**Z: Vorig jaar heeft het zeer zeker zijn nut bewezen omdat je zag dat rond februari-maart het aantal ziekmeldingen afnam.** *En ik ben er ook van overtuigd dat het ziekteverzuim af neemt. P_4b_*

**D: Ja.**

**Z: Nu is dat effect er nog niet, maar ja wie weet gaat dat toch weer komen. Dit jaar zijn het andere kinderen die zich ziek melden dan vorig jaar dus in die zin heeft het ook effect. Dat geldt niet voor alle kinderen maar het gros zijn eigenlijk allemaal nieuwe meldingen.**

Het kost veel werk. Waarom moeten we dit doen?

**Z:** *Het is een glijdende schaal naar beneden, op het moment dat een kind merkt dat hij zich gemakkelijk kan ziek melden is de volgende keer de stap weer wat kleiner. Op die manier ga je toch heel langzaam maar zeker richting voortijdig schooluitval.S_4a_*

Nog meer redenen waarom je aan ziekteverzuimbegeleiding moet doen, als school?

**Z: Nou, ja, ik vind dat met name ouders zich misschien maar bewust moeten worden van het feit dat kinderen toch wel heel snel en gemakkelijk zeggen van ‘ik ben ziek’.**

Dus je wilt een bewustwording op gang brengen? Bij ouders alleen?

**Z: Ook bij kinderen.**

Bij nog meer mensen?

**D: We hebben ook een maatschappelijke opdracht. Je leidt uiteindelijk toe naar goed burgerschap en als je straks in een werksituatie terecht komt en je hebt in het verleden alleen maar meegemaakt dat je je heel gemakkelijk ziek kunt melden dan heb je daar ook straks in de werksituatie last van.**

Wie heeft daar last van?

**D: Daar heeft in eerste instantie je werkgever last van maar als het goed is krijg je daar op termijn zelf ook last van. Het is een beetje zoals bij medewerkers. Op wat voor soort verzuim kun je invloed uitoefenen? Dan staat inmiddels wel vast dat je als leidinggevende, en vergelijk dat even met school die nu op een zelfde soort wijze met het verzuim van leerlingen omgaat, dan kun je invloed uitoefenen op het frequent en kortdurend verzuim. Dat zijn de meeste gevallen. Het opent soms de ogen als je mensen laat zien dat dat veel is, 4 keren per 12 maanden. Mensen beseffen dat vaak niet eens. Leg eens uit. Hoe komt dat nou? Wat is er aan de hand? Wat kunnen we doen? Want als er echt iets aan de hand is kunnen we er rekening mee houden of kunnen we er misschien iets aan doen. Of is er niets aan de hand en is de verzuimdrempel heel erg laag. En dat zelfde zie je ook bij ouders en zeker bij kinderen die opgroeien, in de leeftijd die wij hebben. Ik voel me niet lekker, en ik ga niet naar school, en dat je denkt ach blijf jij maar lekker thuis een dagje, het kan wel lijden. Je moet dit eigenlijk niet willen met elkaar. En in dat kader hoop ik dat het effect heeft.**

**Z: ouders zeggen dat ook vaak, we hebben ons niet gerealiseerd dat wij ons kind zo vaak ziek melden, dat krijgen we terug van ouders.**

**Hoe zijn de reacties van ouders?**

**Z: Vorig jaar over het algemeen positief, dit jaar heb ik al 3 keer meegemaakt dat ze weigeren om te komen – al veel naar dokter geweest, dit voegt niets toe, een zonder reden, een tgv fouten in de planning van de GGD. Dat kan gebeuren, ouders waren wel heel boos. Daarom moet je het niet alten, ik vind wel dat je er mee door moet gaan.**

**D: Wat ik ook belangrijk vind, en ik hoop dat dat straks ook blijkt uit de resultaten, dat aan de voorkant het effect moeten hebben dat de verschillende partijen die betrokken zijn, de school gaat soms op de stoel van de dokter zitten om te achterhalen wat er aan de hand is. Daar zijn we niet van, daar zouden anderen naar moeten kijken, in dit geval de schoolarts, leerplicht die niks doet want die zegt voor ons is er een wettelijke melding dus er is eigenlijk niks aan de hand en de GGD die wij konden adviseren aan ouders om naar toe te gaan, maar dat advies konden ouders even vrolijk in de wind slaan.**

**Z: En nog een partij: collega’s die roepen van ‘och ja die is altijd ziek’…**

**D: Ja. Maar al die partijen, ook die collega’s doen mee want die kan zijn werk ook beter doen als die kinderen er allemaal zijn, zouden hun werk beter moeten kunnen doen door deze samenwerking. Wat mij niet helemaal duidelijk is is of we dat doel wel bereiken. Daar heb ik nog geen zicht op. Los van het aantal meldingen, …**

Dan is het doel beter samenwerken, kortere lijntjes. Wat bedoel je daar dan mee?

**D: Leerplicht doet niets bij een ziekmelding – hangt van lpa af-, terwijl transparanter moet worden nu door deze aanpak, of er inderdaad sprake van ziekte is waardoor een kind geen onderwijs kan volgen of dat dat er iets anders aan de hand is waardoor duidelijk wordt dat men die leerling onttrekt aan de leerplicht en als dat duidelijk is dat de leerplicht actie kan ondernemen. Ik weet niet of men leerplicht merkt dat men vaker in actie moet komen vanwege het feit dat we nu met M@ZL werken. Ik weet niet precies of wij al wat meer geholpen zijn dus wat minder de dokter of Sherlock Holmes moeten uithangen, nu ouders met leerlingen bij de schoolarts komen.**

**Z: Vooropgesteld dat ik nooit op de stoel van de dokter ben gaan zitten of heb willen zitten…**

**Ik vind wel heel prettig dat je op je op zo’n formulier bv ook kunt aangeven ik heb het vermoeden dat het ziekteverzuim niet helemaal uit ziekte bestaat maar dat er andere dingen spelen en dat je dan ook kunt aangeven of je dat wel of niet met de ouders hebt gedeeld. Dat vind ik wel heel prettig dat iemand daarin meekijkt en dan ook kan concluderen je hebt gelijk of je hebt geen gelijk want er is wel degelijk wat aan de hand.**

Dat is de meerwaarde van dat je het door kan zetten naar een arts?

**Z: Ja, en dat is ook wat ik richting collega’s zeg van ‘Wij roepen wel heel hard van ze is er weer niet maar er is wel degelijk een reden voor’.**

Dan hebben we het over beeldvorming?

**Z: Ja, het beeld wat een docent van een leerling heeft kan dan ook veranderen en bijgesteld worden.**

Dat kun je ook zien als een meerwaarde, winst?

**Z en D: Ja.**

Jullie hebben M@ZL-compleet, je zet door naar de jeugdarts als een leerling voldoet aan een van de criteria. We hebben ook M@ZL-smal, eerst zelf een verzuimgesprek voeren. Zouden jullie, met alle ervaringen, opnieuw kiezen voor M@ZL-compleet?

**Z: Als ik mag kiezen zeg ik ja. Op het moment dat je een verzuimgesprek gaat voeren met ouders en je gaat zeggen ‘u meldt uw kind toch wel heel vaak ziek’ is er kans dat de ouders in de verdediging schieten wel heel erg groot, terwijl nu als de mentor belt en zegt ‘de zoveelste ziekmelding, mag ik vragen wat er aan de hand is? en we gaan dit doorzetten naar de GGD zodat zij met ons mee kunnen kijken of wij de goede dingen voor uw kind doen’ dat is een heel andere insteek dan wanneer je zegt ‘hij is al de zoveelste keer ziek vertel eens wat is er aan de hand?’** *Als ik het eerste verzuimgesprek aan mentoren over moet laten, zonder mijn mentoren nou heel erg af te vallen, maar dan vraag ik me af in hoeverre die gesprekken goed gevoerd worden. Want je kunt een gesprek voeren op heel veel verschillende manieren en de een zal dat heel erg gemakkelijk afdoen en de ander zal daar toch….S_4b_*

En dat werkt goed?

**Z: Ja, over het algemeen werkt dat goed.**

Hoe reageren de mentoren?

**Z: Die bellen gewoon naar huis, ik zou niet weten hoe ze anders moeten reageren. Ze kunnen zeggen we doen het niet maar dat is natuurlijk niet de bedoeling.**

**D: Die hadden natuurlijk toch al als mentor de opdracht om bij langdurige afwezigheid vanwege ziekte contact te onderhouden.**

**Z: Dat hoort er gewoon bij.**

**D: Alleen er zit nu iets bij en belangrijk is dat de mentor dan goed op de hoogte is wat M@ZL inhoudt en het ook brengt in de aanbodsfeer, we zijn er allemaal bij gebaat dat het kind naar school gaat of dat we kijken wat we kunnen doen om uw kind zo snel mogelijk weer naar school te laten gaan of wat op school nodig is om dat zo goed mogelijk te laten verlopen en minder dat we langskomen om u de les te lezen. Is dat laatste nodig dan is er ook aanleiding voor, maar dat wordt dan ook maar duidelijk. Het is veel meer vanuit de ondersteunende kant omdat we er allemaal belang bij hebben dat het kind naar school toe gaat. Dat valt niet overal zo maar dat heeft ook te maken met de situatie waarin ouders zitten of het kan van alles zijn waardoor ze de boot afhouden of waarom ze zeggen ‘nou, dat is wel een hoop gehijg in de nek’ , daar ben ik toch als ouder verantwoordelijk voor, waar bemoeien ze zich allemaal mee.**

Ja, die ouders kom je tegen, wat doen jullie daar dan mee?

**D: Ja, ik heb hem toch ziek gemeld, wat zitten jullie nou te zeuren?**

Wat doen jullie als ouders niet willen gaan? Of je krijgt teruggekoppeld dat ze niet geweest zijn?

**Z: Dan is het in principe zo dat er een tweede uitnodiging komt, maar ik heb nu pas de eerste waar dat advies gegeven wordt. Komen ze dan weer niet en er komt een ziekmelding dan gaat het leerplicht in beeld komen.**

**D: Is het zo dat er aan de voorkant een afweging is of je wel of niet meldt?**

**Z: In principe niet, in een heel enkel geval wel, maar dat is zeer uitzonderlijk.**

**D: Ik begrijp dat dat de afspraak niet is, maar kijkend naar de praktijk, werkt het dan ook zo? Zijn er toch gevallen waar de overweging geldt we gaan niet melden en waarom dan niet?**

**Z: Dat is heel uitzonderlijk.**

En wanneer is dat dan bijvoorbeeld?

**Z: Een meisje loopt ziekenhuis in en uit, die zou ik consequent moeten melden maar dat heeft niet zoveel zin, want daar voegt M@ZL niets aan toe.**

**D: Als het een herhaling van zetten wordt en iedereen weet hoe de vlag erbij hangt dan zeg je in zo’n geval meld ik niet.**

Is er in je afweging nog iets wat dan een rol speelt?

**Z: Ja, er komt bij dat er een goed contact met ouders is, dat het kind naar school komt op momenten dat het wel gaat en dat er heel goede afspraken zijn gemaakt en dan is het helder en is het oké. Ik wijk niet voor ouders die zeggen ‘Dat is belachelijk en dat ga ik niet doen’ dan zeg ik dat is aan u, ik meld het en als u niet gaat dan zijn er consequenties en die zijn voor u en dan gaan ze. De consequentie van niet gaan is melding bij de leerplichtambtenaar.**

**D: Dat was mijn vraag. In hoeverre hebben we daar ervaring mee?**

**Z: Er zijn door Jeanette nu 2 of 3 leerlingen zo ver dat ze gemeld moeten worden, maar ik nog niet dus wat dat betreft hebben we gewoon heel meewerkende ouders.**

Je bent heel tevreden over hoe het nu gaat?

**Z: Ja**

Ben je ook tevreden over de terugkoppeling?

**Z: Ja, want daar staat heel duidelijk het advies in wat we moeten doen, er staat ook in, als iets langdurig is, dat er contact wordt onderhouden door de schoolarts, ja dat vind ik wel prettig.**

**D: Weegt de investering op tegen het rendement, het resultaat? Of zeg je dat moeten we over een langere tijd bekijken….**

Wat is het rendement dat we willen hebben? Wat is de opbrengst?

**D: Ik denk aantoonbaar minder ziekmeldingen, of in ieder geval minder frequent kortdurend verzuim.**

Waar zou je ook tevreden mee zijn? Welk rendement wil je nog meer zien?

**Z: Dat is een beetje moeilijk, kijk, als mensen zich bewust worden van het feit van oh, als mijn kind zegt ik heb hoofdpijn of ik ben ziek, dat hij een toets heeft of omdat hij iets moet inleveren, ook dat zou heel fijn zijn als daar effect in te zien is, en hoe je dat moet weten weet ik niet, maar dat zou wel heel prettig zijn.**

Dan kom je weer bij wat je in eerste instantie zei, we hebben een maatschappelijke opdracht, we willen dat bewustzijn…

**D: Ja, die verantwoordelijkheid ook stimuleren als die er onvoldoende zou zijn, die verantwoordelijkheid die hoort bij het ouder-zijn. De verantwoordelijkheid als ouder en dat heeft ook te maken met ouderbetrokkenheid bij school en bij alles wat er gebeurt, bij het belang dat kinderen naar school gaan en er ook een hele grote rol in hebben. Het kan niet zo zijn dat kinderen naar school moeten en dat ouders zeggen, ja dat zal wel en we zien het wel en het stimuleren van die ouderbetrokkenheid in z’n algemeen en dit is daar wel een onderdeel van, ja die kan daarmee vergroot worden. En ik denk dat dat toch een zaak is, en dat merken we ook best wel, dat het niet vanzelfsprekend is dat die ouderbetrokkenheid er is. En daar kunnen allerlei oorzaken voor zijn en omstandigheden zijn, het is niet in de veroordelende sfeer waardoor die er soms te weinig is.**

Vind je in M@ZL de ouders tegenover je of naast je als school?

**D: Wat ik begrijp over het algemeen naast je. En dat moet het ook zijn.**

**Z: Ja.**

Dan heb je iets waarmee je de ouderbetrokkenheid ook kunt vergroten**.**

**P: Ja, je bent elkaars bondgenoot, ouders, leerling en school trekken aan dezelfde kant van het touw streven, dezelfde dingen na. Ook voor als leerlingen uitvallen omdat ze verkeerde keuzes maken en op het verkeerde pad terecht komen, dan is dat ook voor de leerling en ouders een probleem, en niet alleen voor de school. We zijn samen verantwoordelijk. En dan verlangen we ook wel iets van het handelen om die rol ook goed samen te spelen en zolang je daar in de relatie met elkaar goed over kunt hebben, je hoeft het niet in alles met elkaar eens te zijn, maar dan houd je elkaar wel vast. Als je op enig moment als school tegenover de ouders en leerling komt te staan dan komen er uit die strijd over het algemeen alleen maar verliezers. Daar wordt niemand beter van. Dus het is ook van belang vind ik om duidelijk te zijn in wat je wilt en wat je verwacht, maar ook die relatie goed te houden omdat je beiden hetzelfde belang dient. Maar dat veronderstelt iets van de rol die je als ouder speelt. Ik denk dat je dat ook merkt, als ouders die rol goed spelen zullen ze hun kind ook niet om de haverklap ziek melden als hij niet ziek is, want dan hebben we dat belang beiden goed voor ogen.**

**Z: Of misschien melden ze het kind wel ziek maar dan zeggen ze er meten bij ‘Ik krijg hem niet naar school, weten jullie niet wat wij kunnen doen? Dan heb je dat effect ook.**

**D: Prima.**

**Z: Open kaart spelen, kun je me helpen, mogelijk speelt er wat.**

Stel dat je een collega-directeur in het land tegen komt, jij vertelt over M@ZL en hij reageert van ‘nou, dat heeft voor mij helemaal geen prioriteit, ik heb wel andere dingen aan mijn hoofd, andere dingen in mijn top 10 staan die ik moet aanpakken.

**D: Dat kan zomaar zo zijn.**

Staat het bij jou wel in je top 10?

**D: Ja, want anders waren we er niet mee begonnen. Dan hadden we het niet moeten doen.**

Hoe ga je in gesprek met zo’n collega?

**D: Of hij moet aantoonbaar kunnen maken, laat het maar zien, dat die ziekmeldingen op die school, dat je zegt daar is ook helemaal niets aan de hand, er is weinig kortdurend frequent verzuim, het is acceptabel, kinderen verzuimen nauwelijks, ja als dat aan de orde zou zijn, dan kun je je afvragen hoe hoog de prioriteit is. Maar ik kan me bijna niet voorstellen dat de wereld 6 straten verder er zo compleet anders uit ziet dan hier.**

Dus je zegt eigenlijk als je net zoveel ziekteverzuim hebt als wij hier dan zou het in je top 10 moeten staan**.**

**D: Ja.**

Maar waarom moet het in je top 10 staan?

**D: Omdat je ook daadwerkelijk hoopt en verwacht dat het het gewenste resultaat oplevert. Als je niet geloofd in wat je er mee wilt bereiken dan werkt het niet. Dus je kunt ook zeggen ‘eerst zien en dan geloven’, Laat ze eerst overal maar eens doen en als dan aangetoond is dat het werkt dan wil ik het ook wel eens gaan doen. En hier ga je er vanuit ik geloof er in en we zullen het laten zien. Dan draai je het om. En dan weet ik ook niet hoe het uit gaat pakken, en daarom is het goed dat we dat monitoren en aantonen dat het ook echt iets oplevert en dat de inspanningen die er zijn, want het kost veel tijd en inspanning, ook in verhouding staat met elkaar, dat is een afweging die je met elkaar op enig moment moet maken. Je kunt wel zeggen we schuiven iets op, maar als het bovenmatig veel inspanning vraagt moet je je toch ook afvragen of we er mee doorgaan.**

**Z: Ik ken scholen die het door de administratie laten afweten, dat is in ieder geval niet mijn keuze, want doordat je er als zorgcoördinator en mentor zo mee bezig bent vergroot je ook de betrokkenheid naar het kind toe en andersom geef je ook de boodschap aan ouders ‘wij maken ons zorgen, wordt het niet eens tijd dat jullie die zorgen met ons gaan delen?’**

**D: Ja, dat onderschrijf ik. Het is geen administratief verhaal, dus je moet dat in je begeleiding meenemen en in de rol van school ten aanzien van de zorg. Daar hoort het thuis. Ten tweede, als aangetoond wordt dat het effect heeft en dat deze wijze van verzuimbegeleiding wat oplevert dan zou het natuurlijk mooi zijn als dat inderdaad op enig moment in de plaats komt of een plek krijgt in het basistakenpakket van de GGD. Omdat ik denk, maar goed, dat dat die met die pollepel, zoals ik dat vroeger ook heb gehad, en die hand in je broek, dat dat wel een keer klaar is, dat dat minder gegevens en resultaat oplevert dan dit verhaal en dat datgene wat daar uitkomt op deze manier ook wel naar voren komt.**

Daar is in andere interviews al heel veel over gezegd, wat jij nu met deze zin onderschrijft, dus ik ga er nu liever niet verder op in….

De leerlingen worden aangemeld bij de jeugdarts. Wat verwachten jullie van zo’n consult?

**Z: Ik verwacht in eerste instantie dat de jeugdarts in gesprek gaat. De school heeft je aangemeld, vertel, waarom ben je hier, hoe komt het dat je je zo vaak ziek meldt in zo’n korte tijd, wat is er aan de hand? Ik verwacht nog niet eens direct dat lichamelijke onderzoek, ik weet niet of ze dat doet, dat maakt me verder ook niet zo heel veel uit, maar dat zij op zoek gaat naar de oorzaak van dat verzuim en dan de juiste adviezen geeft aan ouders, kind maar ook aan ons want het kan ook zomaar zijn dat wij in een bepaalde periode veel te veel toetsten plannen waardoor kinderen denken ik red het niet meer dus… Dan doen we als school iets niet goed, dan moeten wij gaan nadenken. Het kan zijn dat het een kind is dat heel veel moeilijkheden uit de weg gaat, dan kun je ook het advies geven ga er eens mee praten en ga eens ondersteunen in het plannen van..**

Hebben jullie standaard terugkoppeling in het ZAT?

**Z: Ja**

**D: Wat bedoel je met de terugkoppeling in het ZAT in relatie tot dit verhaal?**

Ik ben aan het zoeken naar de verwachtingen van jullie als school ten aanzien van de JGZ.

**Z: Er zijn een heleboel kinderen die wel bij M@ZL aangemeld worden en niet in het ZAT besproken worden.**

Het gaat er vooral om de leerling in het ZAT te bespreken als ze niet geweest zijn of als er een hulpvraag is, maar goed dat kun je ook buiten het ZAT om doen. Vragen naar het vervolg, is er iets op gang gekomen?

**Z: In het ZAT staat bij ons toch veel meer de vraag centraal van wat doen we met elkaar, het adviseren aan elkaar, het weten van elkaar wie wat wanneer doet, de afstemming, terwijl de kinderen die bij M@ZL niet geweest zijn, die zijn nog helemaal niet aan het bespreken in dat ZAT toe.**

Dus de terugkoppeling vindt meer plaats op het niveau jeugdarts-zorgcoördinator?

**Z: Ja.**

Uiteindelijk merken we wel dat vooral het traject nadat ze gemeld zijn, het monitoren, dat dat nog wel lastig is. Klopt dat?

**Z: Ja.**

Wat gebeurt er uiteindelijk met het advies dat is gegeven?

**Z: De terugkoppeling gaat sowieso naar de mentor en de leerjaarcoördinator en dan ligt het er ook aan wat daar in staat. Staat daarin ga met ouders in gesprek ja dan gaat dat gebeuren. Staat daar in er is niets aan de hand, het kind kan gewoon naar school, ja dan komt dat kind gewoon naar school.**

**D: Leggen wij die gegevens vast in Editis?**

**Z: Of die in Edictis staan dat weet ik niet. Ik weet wel dat ik alle M@ZL-aanmeldingen en terugkoppelingen in een aparte map in mijn kantoor heb staan, op papier en in mijn mailbox op de computer.**

**D: Even los van privacygevoeligheid, het zou wel handig zijn als het in je leerlingvolgsysteem daar iets van te hebben staan al is het maar dat de leerling is aangemeld. Waar zet je het neer, ook in het kader van dossiervorming, anders krijg je het op allerlei plekken.**

Hoor je ook wel eens iets terug van de leerling of de ouders over het consult bij de jeugdarts?

**Z: Alleen in die gevallen waar er ook serieus medisch iets aan de hand is.**

**D: Doen jullie ook interviews met ouders?**

Nee, dat nemen we nu niet mee. Mogelijk straks in een stakeholdersonderzoek.

**D: Het zou wel aardig zijn om te weten hoe ouders het ervaren, deze ondersteuning die in het kader van M@ZL geboden wordt. Zijn die daar tevreden over?**

Ze krijgen in het kader van het onderzoek nu op 3 momenten een vragenlijst. Daarin worden dit soort vragen ook gesteld.

**D: Hoe is de respons?**

De respons van de nameting valt nu nog wat tegen.

**D: Daar was ik al bang voor.**

Bij een heel lage respons moeten we wel wat gaan verzinnen, bv toch telefonisch navragen. Er volgt uitleg over de onderzoeksprocedure…. Veel mensen zijn slecht bereikbaar.

**D: Inderdaad, herkenbaar.**

We hebben een aantal jaren geleden M@ZL ontwikkeld, het begon met het regelen va de instroom, vervolgens de inzet van de jeugdarts en de terugkoppeling. Nu merken we dat dat alleen niet voldoende is. Je moet het ook binnen de school implementeren. Als we even teruggaan in de tijd. Je hebt ziekteverzuim van leerlingen en je wilt het als school aanpakken hoe zou je dat dan doen? Kun je dat alleen, heb je daar de JGZ voor nodig?

**Z: Ik denk niet dat je dat alleen kan.**

**D: We hebben het jaren bijna alleen gedaan.**

**Hoe hebben jullie dat toen gedaan?**

**D: met hooguit een verwijzing naar de GGD bij vermoeden van onrechtmatig ziekteverzuim en actie richting leerplicht. Ik denk dat het ook landelijk gezien, in het kader van VSV, veel meer op de agenda is gekomen dan een groot aantal jaren terug.**

Ook ziekteverzuim?

**D: Ja, ook ziekteverzuim.**

Ja, hoor je wel eens dat ze het er over hebben? Landelijk gaat het volgens mij alleen maar over ongeoorloofd schoolverzuim**.**

**D: Ja, maar er zit natuurlijk heel veel ongeoorloofd verzuim in het ziekteverzuim, i het verzuim dat als ziekteverzuim wordt gemeld. Ik denk dat er veel meer aandacht voor dat totale verzuim is gekomen. Dat geldt voor het verzuim van medewerkers ook, dat zit een beetje in de tijd, en ook om na te denken over hoe we er invloed op kunnen uitoefenen. En dat wil zeggen dat je het ook tot je verantwoordelijkheid bent gaan rekenen. Je kon vroeger ook zeggen ja wij zijn school en als ouders ziek melden dan zijn we er mee klaar. En misschien is dat verzuim, er moet natuurlijk ook aanleiding zijn om er zo naar te kijken en er anders mee om te gaan, misschien is het ook wel toegenomen. Want als dat niet was hadden we er met z’n allen ook niet aan gedacht om er iets aan te gaan doen. Maar dat weet ik niet hoor.**

Heeft de inspecteur ook aandacht hiervoor?

**D en Z: Nee. In de kwaliteitsspiegel die wordt gemaakt op de portal heb ik heel bewust het document van M@ZL in gehangen, bij die kwaliteitsindicator.**

Je vindt het een kwaliteitsindicator als je aan ziekteverzuimbegeleiding doet, als je structureel aandacht besteed aan…

**Z en D: Ja. We moeten aandacht besteden aan de kwalitatieve inhoud van ons onderwijs, aan de activiteiten die we doen en die we tot de onderwijstijd rekenen, maar we hebben in dit land ook bedacht dat kinderen in de onderbouw 1040 uren naar school moeten. En als je door verzuim, dat je als ziekteverzuim meldt en dat voor een deel misschien geen ziekteverzuim is, dat jouw kind daar niet aan komt, dan mist hij dus onderwijs. Voor mij is het een duidelijke zaak dat dat ook een onderdeel is van de verplichting die we hebben om niet alleen goed onderwijs te verzorgen maar ook de omvang, dat kinderen voldoende goed onderwijs krijgen.**

Niet alleen kwaliteit maar ook kwantiteit van het onderwijs telt?

**D: Ja, maar ik heb de inspecteur er trouwens nog nooit over gehoord.**

Wat is jouw top 10 als directeur, waar je je het meeste druk om maakt op dit moment?

**D: Het zijn er meer dan 10 maar dat maakt niet uit. Het akelige is dat we ze ook vastleggen in ons meerjarenbeleidsplan en in ons jaarplan en dat is ook weer gerelateerd aan het strategisch beleid van het ROC, die hebben ook een top 7 en als zij een top 7 hebben dan hebben wij ook minstens die top 7. Dus die focuspunten van beleid staan wel degelijk op een rij. Dat is voor onze school dat we goed kijken dat we onze basiskwaliteit op orde hebben, door de bril gezien van de inspectie in verband met de beoordeling van het onderwijs.**

Dan heb je het over de kwaliteit van onderwijs geven?

**D: Ik weet niet of dat hetzelfde is. Die indicatoren dat is natuurlijk niet allemaal onzin, dat heeft wel met elkaar te maken, maar je wordt er wel steeds meer op afgerekend, op kwaliteitskaarten, je rendement onderbouw en bovenbouw, de opbrengsten, centraal examencijfers, schoolexamencijfers.**

Cijfers van het slagen? Het slagingspercentage? Is dat het rendement?

**D: Het slagingspercentage doet niet eens mee. Het staat wel vermeld. Bij de opbrengsten gaat het over datgene wat je realiseert met kinderen want we zijn uiteindelijk een opleidingsfabriek, hoe je het ook went of keert, daar is wel heel veel aandacht voor. Taal, rekenen, wiskunde, Engels, achterstanden wegwerken, dus als je kijkt naar het akkoord dat nu gesloten is tussen de vo-raad en het ministerie over wat we de komende jaren voor elkaar moeten boksen dan valt op dat sommige resultaatverplichtingen wel heel concreet zijn, bv 46% van de leerlingen met LWOO zou natuur-scheikunde moeten kiezen. Aantoonbaar moeten de resultaten van Nederlands wiskunde en Engels omhoog met zoveel punten. Er is heel veel accent op die kant van het onderwijs en dat is politiek gestuurd. We zijn natuurlijk toch een beetje afhankelijk van de politiek. We hebben jaren gehad kinderen worden te dik, onderwijs doe eens wat. Allerlei dingen waarvan je soms de vraag kon stellen of dat tot de core business van het onderwijs horen zijn door diezelfde politiek de afgelopen jaren keurig naar het onderwijs geschoven. Op dit moment zegt men heel nadrukkelijk ne-wi-en is slecht en vervolgens wijst iedereen naar beneden, het hbo wijst naar het mbo, het mbo naar het vmbo, het vmbo naar de basisschool, hoe je het ook went of keert, de norm moet omhoog. Dat is een. Verder zit er in dat hele toezicht, naast die rendementen, je kwaliteitszorg. Het pedagogisch-didactisch klimaat, hoe ga je met kinderen om, schoolveiligheid, sociale veiligheid, het doet allemaal mee, het omgaan met verschillen. Zijn docenten in staat om te gaan met verschillen aan de pedagogische kant. Kinderen komen met heel veel leer- en andere bijzonderheden binnen en van de docent wordt verwacht dat hij er allemaal mee om kan gaan.**

Dan heb je het eigenlijk over passend onderwijs?

**D: Dat heeft absoluut te maken met passend onderwijs maar die situatie is zoals die is en verandert niet zo. En dat heeft vervolgens implicaties op de vakdidactiek, want als je het pedagogisch kunt dan zul je het in je vakdidactiek ook nog eens zo moeten kunnen organiseren dat je kinderen het goede aanbod doet en dan hebben we het in het kader van de zorg daar waar het LWOO leerlingen betreft handelingsplannen, dan moet je ook nog handelingsgericht werken en daarmee vraag je in alle eerlijkheid van die professional heel veel. Wie zijn die leerlingen? Hij moet zijn klas kunnen handelen, om kunnen gaan met verschillen,didactisch een goed aanbod hebben en ook nog weten oh ja die heeft dat en die…. Dat moeten dan toch allemaal witte raven zijn. Je vraagt echt heel veel van die professional. Dat komt heel nadrukkelijk in alle beleidsstukken terug, dat men heel veel energie wil steken in professionalisering, het op peil houden of brengen van wat dat vraagt van de mensen voor de klas, en dat is nooit gemeengoed geweest. In onderwijsland in ieder geval niet.**

**Z: Tot een jaar of 10 geleden was dat eigenlijk ook niet nodig. Je ging voor de klas staan, je keek eens boos en dan was het zo.**

**D: Ja dat klopt.**

**Z: Maar die tijd is voorbij.**

**D: Ja, absoluut voorbij.**

Hoe komt dat?

**D: De wereld verandert. Als je kijkt naar wat wij tegenwoordig niet benoemen aan problematiek in het kader van leren en gedrag, het onderkennen en diagnosticeren. Vroeger was het simpel, als je goed kon leren zat je voor in de klas, en als je het allemaal niet kon zat je achterin de klas. Een docent had gezag. Als je op school op je donder kreeg en ze hoorden het thuis dan kreeg je thuis nog 3 keer op je donder. Dat is veranderd. Dat is wezenlijk veranderd. En dat maakt dat je in een andere situatie terecht komt en dus is die noodzaak om te professionaliseren echt aanwezig omdat je anders ongelukkige mensen in je school krijgt of mensen die verzuren en de oorzaak van de problematiek vooral buiten zichzelf neerleggen en te weinig kijken naar wat kan ik er aan doen? En als je sowieso binnen elke schoolorganisatie kijkt, 80% van iedere euro van Hare majesteit gaat iedere maand rechtsreeks naar het salaris van de medewerkers, 20% zit in de materiële component. Als ik bedenk hoe gestructureerd we omgaan met die materiële component en hoe zeer we er al die jaren van uit zijn gegaan dat die 80% wel vanzelf rendeert, komen we nu wel tot het besef dat dat niet zo is. En daar moet je op anticiperen. Of mensen moeten veel meer werkzaam in het onderwijs of misschien wel moeten doen, want de noodzaak was er veel minder, zich af blijven vragen doe ik de goede dingen, haal ik er energie uit of kost het me alleen maar energie, hoe lang moet ik nog werken en blijf ik dit doen en als ik dit blijf doen wat is daarvoor nodig of als je tot de conclusie komt ik had er nooit voor moeten kiezen of ik met het in ieder geval niet de rest van mijn leven blijven doen, wat doe je dan als organisatie maar ook als medewerker zelf om te zorgen dat er een andere weg in wordt geslagen. Tot nu toe kom je soms nog of misschien toch wel regelmatig in de krant als je met pensioen gaat en je hebt 45 jaar lang op dezelfde school gewerkt, maar je kunt zeggen dat is eigenlijk een veeg teken, dat je 45 jaar lang op dezelfde plek hebt gezeten. Er is dus iets veranderd.**

Maar hoe past ziekteverzuimbegeleiding daar in?

**D: Ziekteverzuimbegeleiding moet er mede voor zorgen dat die kinderen op school zijn, dat ze onderwijs krijgen, dat die docent daarmee zijn werk goed kan doen, als de leerling ziek is geweest mist hij dingen, dat moet weer allemaal ingehaald worden, dus het draagt er in die zin aan bij.** *Het draagt er aan bij in het veranderende normbesef in de hele samenleving dat het er toe doet, dat je rechten en plichten hebt, en als je leerplicht hebt dan moet je naar school P_4a_* **en als je straks werkt dat je de verplichting hebt naar je werkgever om daar je stinkende best voor te doen. En als wij in onze samenleving alleen maar prinsjes en prinsesjes opvoeden, als dat straks de kurk is waar de hele samenleving het van moet hebben of dat dan wel goed gaat komen.**

U zegt ziekteverzuimbegeleiding kan er toe bijdragen dat ze meer op school zijn waardoor de docent zijn werk kan doen en kan…

**Z: Als je het heel scherp stelt, er is een samenhang tussen voortijdig schoolverlaten en criminaliteit, dat is aangetoond. Op het moment dat er veel ziekteverzuim is is de kans op voortijdig schoolverlaten groot, dus de kans op criminaliteit ook. En in die zin ben je gewoon verplicht iets aan dat ziekteverzuim te doen. Richting ouders en kinderen. Want ik geloof ook dat ouders niet altijd bij machte zijn om dat kind, dat perse niet naar school wil omdat hij zijn Duits opdracht niet af heeft, om die dan toch naar school te sturen.**

Dus je kunt zo ouders ook steunen in hun opvoedingstaken?

**Z: Ja.**

**D: Eerlijk gezegd denk ik dat wij op school de afgelopen jaren alleen maar strenger zijn geworden en dingen meer en beter in beeld hebben gekregen. Als ik het even afzet tegen mijn eigen middelbare schooltijd en hoe ik die heb ervaren, dat was een andere tijd, dat snap ik wel, maar we hebben heel wat in de kroeg gezeten en er was geen haan die daar verder naar kraaide.**

**Z: Dat is het verschil tussen stad en dorp hoor, kees!**

**D: Ja, dat snap ik wel, maar ik heb van de ene kant de indruk dat we daar veel meer zicht op willen hebben, daar consequenties aan verbinden en het ook belangrijker vinden dat we dat doen, maar kennelijk is er ook een noodzaak toe.**

**Z: De noodzaak tot opleiding is in deze tijd ook groter. Vroeger, als je niet naar school ging, dan ging je bij een baas werken en dan kwam je er toch wel, maar dat is niet meer.**

**D: Sterker nog, we kunnen het ons niet meer veroorloven om straks heel veel jongeren tussen de wal en het schip te laten vallen, want er zal dan een generatie zijn…**

Dus je valt nu tussen wal en schip als je…

**D: Ja, en een generatie die het verdient, als je kijkt naar de leeftijdsopbouw in onze samenleving, dan ben ik daar niet zorgenvrij over. Over zoveel jaar hebben we zoveel meer ouderen en hebben we zoveel minder werk. Dus de noodzaak om jongeren goed op te leiden, die straks ook de kurk kunnen zijn waarop de samenleving drijft, die is gewoon hartstikke noodzakelijk aanwezig.**

En dat heeft ook te maken met dat de eisen die aan jongeren worden gesteld anders zijn….

**D en Z: Ja.**

Want jij zat in de kroeg en bent toch goed terecht gekomen, hé kees?

**D: Ja, daarom. Op enig moment heb ik toch het licht gezien.**

Ik hoor ook wel eens mensen zeggen och houd toch eens op met boven op die kinderen te zitten, het komt allemaal wel goed. Maar die overtuiging hebben we niet meer?

**Z: In de tijd dat wij zijn opgegroeid kon je dat je ook nog permitteren. Er is een hele tijd geweest dat als je geen baan had je de hand maar hoefde op te houden en dan kwam er toch geld. Ze konden allemaal leven. En dat is nu gewoon klaar.**

Dus niet alleen de gezinssituatie thuis maar ook de maatschappij is verandert en stelt andere eisen en kinderen raken tussen wal en schip als ze zonder diploma van school komen.

**D en Z: Ja. En de kans om straks aan de bak te komen wordt minder als je voortijdig de school verlaat, en dus de weg naar de criminaliteit, dat is een open deur, die kans wordt ook groter. Dus het helpt allemaal wel. het is een beetje analoog aan het verhaal dat we aandacht besteden aan de verkeersveiligheid. Dat is weinig meetbaar, of dat dat ook bijdraagt tot ander verkeersgedrag, maar een ding weet je zeker. Als je niets doet helpt het in ieder geval niet.**

En jij hebt aan je morele verplichting, zoals je dat voelt, voldaan? Ik doe in ieder geval als directeur wat ik kan, wat binnen mijn vermogen ligt om mee te geven.

**D: ja, dat telt wel mee.**

**Z: Zo doen we ook dingen met alcohol. Ik vind dat toch ook wel bij een middelbare school horen.**

**D: Ook daar is veel veranderd. De ouderavond heeft commentaar over het aanbod in de kantine. We hebben daarover wel afspraken gemaakt, er komt een catering maar geen frituur, bij voorkeur toch ook gezond aanbod, bruine broodjes en geen witte broodjes, maar goed er liggen in het assortiment ook saucijzenbroodjes, etc. die minder gezond zijn. Prijstechnisch is het dan ook nog zo dat die ongezonde producten goedkoper zijn dan die gezonde producten. Wij zijn bezig met een nieuwe aanbesteding en we willen in het contract dat we als school meer invloed kunnen hebben op het assortiment dan nu het geval is bij de huidige cateraar. Daarmee ben je er niet want het hoort een verhaal te zijn, het stimuleren van kinderen om de goede keuzes te maken, dat het helemaal niet erg is als je een keer een saucijzenbroodje eet maar dat dat in verhouding moet staan. En we kunnen ook niet Roomser zijn dan de Paus, want als wij het niet verkopen fietsen ze naar de Macdonald een eindje verderop om het daar te halen. We moeten dan ook werken aan dat bewustzijn en dan nog moet je je afvragen of het allemaal helpt maar als je niets doet weet je zeker dat het niet helpt.**

**Interview 10. M@ZL onderzoek	Datum: 26-01-2012**

**Aanwezig:	Yvonne Vanneste (onderzoeker), Marlou van de Loo (semi-arts)**

**Directrice school, zorgcoördinator School 9 / P_9_ en S_9_**

**Variant:	second route**

**Algemene gegevens**

Het gesprek vindt plaats op school, in de kamer van de directrice. Marlou start met interview. Goede sfeer. Af en toe wat rommelig, willen allebei graag hun verhaal doen. Duidelijk dat er een uitgebreid zorgnetwerk aanwezig is en veel enthousiasme over M@ZL.

**Verslag interview**

**Vragen/opmerkingen door interviewster dikgedrukt**

Antwoorden/opmerkingen door geïnterviewde in normale opmaak.

Directrice = D, Zorgcoördinator = Z.

**Waarom hebben jullie ervoor gekozen om aan het project M@ZL mee te doen?**

Z: We zijn al heel lang betrokken bij M@ZL, er was ooit vanuit Tessenderlandt, waar wij een onderdeel van zijn, zaten we met veel kinderen die ziek zijn. Er was contact met de GGD, we hadden vroeger altijd standaard de schoolarts, van ‘hoe kunnen we dat nou tackelen’. Toen is er vanuit Tessenderlandt en de GGD, voor zover ik weet, een soort plannetje gemaakt, ‘van laten we is proberen om het zus en zo te doen’.

**Jullie hebben aan de pilot mee gedaan.**

Z: Ja, een soort pilot. Om te kijken of een aantal interventies gaan helpen. En toen heette het nog anders volgens mij, een werknaam. Dus vanaf dag 1 zeg maar, zaten wij, en toen werd het op een gegeven moment M@ZL en voor ons was het toen al een heel bekend project eigenlijk, waar al vingeroefeningen voor gedaan waren. Alleen werd het dan weer een wetenschappelijke benadering. Werd er aangegeven. Dus zo zijn wij daar automatisch mee door gegaan.

**En had u daar wel een rol in, want u zegt het kwam van ‘Tessenderlandt’.**

Z: Mijn collega uit de onderbouw, de leerlingbegeleider, die heeft altijd bij die besprekingen en vergaderingen gezeten. Om het vorm te geven en te kijken.. Kijk, in de onderbouw ligt ook vaak het accent met al die kinderen, met het ziekteverzuim. Ik weet niet of dat in percentages hoger is, maar daar komen kinderen binnen de school en daar lopen ze heel snel tegenaan. Dus het is vanuit klas 1 en 2 eigenlijk gegroeid, van ‘daar moeten we iets mee’.

D: Daar begin je eigenlijk ook met je zorgen, in die eerste klas al. Als je merkt dat kinderen regelmatig niet komen of ziek zijn, dan moet je wel starten.

Z: Precies, en vaak heb je informatie van de basisschool dat er ook al best veel verzuim was, dus zit je daar meteen bovenop. En als kinderen hier dan in klas 3 en 4 komen, is er al van alles gedaan, stappen gezet en soms heb je nog een groot ziekteverzuim, maar…

**Blijven dat dezelfde kinderen?**

Z: Ja, dat blijven dezelfde kinderen. Nee, het zijn echt wel patronen. Je hebt kinderen waar dat verzuim altijd een rol speelt en al gespeeld had. Mijn idee is dat het ook vaak in de gezinsstructuur zit. Dus het is niet, die kinderen die zo lang verzuimen, dat is geen ziekte ‘in de medische zin van ziekte’. Die noemen zich heel snel ‘niet goed’, en dan blijven ze thuis. Ik heb van de week nog een gesprek met zo’n meisje, moeder meldt ziek en dan zeg ik ‘wat is er dan’. Dan zegt ze ‘ik voel me niet goed’. Ik zeg ‘dat snap ik, maar dat komt niet omdat je keelpijn/buikpijn/hoofdpijn hebt, maar omdat je opziet tegen de schooldag’. Ja, dan voel ik me gewoon niet goed.. dan blijf ik gewoon een dag in bed.

En dat zijn kinderen die je door al die jaren heen ziet lopen.

**Dat blijft.**

Z: Ja.

**En kunnen we daar iets aan doen met M@ZL?**

Z: Nou, bij een aantal gevallen, die kinderen stuur je dan naar de schoolarts en dan zijn ze vaak de dag erop weer beter. Dan komen ze weer of als je het gesprek hebt gevoerd al. Of ze zijn daar geweest en dan komen ze de week erop of de dag daarna weer naar school. Als we dan een gesprek hebben gehad, denken ze ‘nu gaat er nog iemand zich mee bemoeien’ en dat vinden ze niet zo heel gezellig. Dan weten ze weer ‘ohja, ik moet mijn kind toch naar school sturen’. Dus in die zin helpt het wel denk ik, dat je het als systeem hebt. Bij signaalverzuim van ‘ja, nu moet je toch eens naar de dokter’. Bij een aantal gevallen, maar ik zou het in percentages echt niet weten.. En bij een aantal levert het niets op en die worden keer op keer besproken in het zorgteam, jarenlang, ook met de schoolarts en lpa erbij. En er verandert niets. Die hebben we ook.

D: Als je nu zegt, langs die onderbouw. Daar kun je natuurlijk, als je er heel vroeg bij bent, kun je wel iets doen. Niet bij allemaal, want je hebt zware gevallen en we hebben natuurlijk een school met kinderen waar bij elk kind wat aan de hand is, dus dat scheelt ook. Maar als je er heel vroeg bij bent, kun je soms toch wel sturen. Niet bij allemaal, nogmaals. Maar bij een heel groot aantal wel natuurlijk. Dan kun je op tijd ingrijpen. Of de leerplicht, of wijzelf, of de schoolarts. Stel dat een kind verzuimt doordat hij in een klas zit waar hij gepest wordt, dan kun je ook school ook iets doen, als je er op tijd bij bent. *En door M@ZL kunnen we de leerling doorsturen voor verdere behandeling, waardoor zo’n kind niet in psychische nood komt. Deze leerling krijgt van ons wel ondersteuning, maar niet de medische ondersteuning die hij misschien nodig heeft. P_9a_*

**Hoe zien jullie de rol van ouders daarin?**

Z: Hoe bedoel je?

D: Ik vind de ouders wel een belangrijke partner.

Z: Nou, er zijn ouders van kinderen die vaak ziek zijn, die niet ziek zijn. Maar dat benoem je dan en dan zeggen ze ‘ja, dat klopt’, dus die komen ook niet bij een dokter. Ouders dekken vaak snel toe dat een kind zich niet lekker voelt, niet goed voelt. Er zijn ook ouders die met hun dochter lekker een dagje thuis in bed TV liggen te kijken.

D: Zakje chips en een cola op de bank.

Z: Filtersigaretje erbij. Daar krijg je het niet veranderd. Want ouders vinden dat eigenlijk goed. Die hebben zoiets van ‘ja, je voelt je niet lekker, dat snap ik, blijf lekker thuis, gezellig bij mij’.

D: Dan moet je misschien ook iets aan de ouders doen.

**Het heeft een meerwaarde voor de ouder om het kind thuis te houden.**

Z: Bij die langdurige zit het in het systeem van het gezin. Het is een manier zoals ze dat in een gezin doen. En kinderen weten dat, moeders krijgen hun dochter dan niet naar school.

D: Nou, vaak, die moeder vindt het wel lekker dat het kind thuis is. Samen met mama, gezellig.

Z: Die zijn er ook, een dagje winkelen en ziek melden. Maar ik denk wel dat als je het als systeem hebt in je school van ‘luister, dan en dan nemen wij contact met u op, na zoveel keren ziek of een enkele dag ziek’. Eerst belt de mentor, dan de leerlingbegeleider en dan hebben we als systeem dat je altijd naar de schoolarts gaat. En vervolgens koppelt die dat terug naar de leerplicht. Dat is natuurlijk wel helder voor ouders, want dan weten ze hoe het gaat. En dat moet steeds meer voor het voetlicht komen. Dat vertellen we ook ieder jaar wel aan de nieuwe ouders die komen in de voorlichting, het staat ook op de website enzo, maar het moet nog steeds meer bij ouders… Zeg maar, dat ze tegen elkaar ook zeggen van ‘god, ja, ze bellen inderdaad na 3 dagen’. Daarvan verwachten we op de lange termijn, maar dat is lange adem, effect. Die ouders moeten ervan doordrongen zijn, dat als je een kind thuis houdt, dat je ook onderwijs onthoudt. En dat ‘ziek’, niet altijd ‘ziek’ is. En dat is lastig. Want er zijn heel veel ouders die zeggen ‘ja, hij is ziek, ik heb toch gebeld’. En die vinden het heel logisch dat, als een kind zegt ‘ik voel me niet lekker’, dat je dus gewoon thuis blijft.

D: En dat is 1 keer per jaar niet zo erg. Dat doen we allemaal wel eens een keer, denk ik. Ik denk dat heel veel ouders… Bij mijn eerste kinderen was ik superstreng, maar bij mijn dochter was ik minder streng, omdat die lichamelijk ook echt vaak iets had.

Z: De energie is op.

D: Dan had ik ook wel eens.. Dan zag ik haar en dacht ik ‘och, blijf maar een dag in bed’. Maar die ging de volgende dag wel weer naar school. Je was als ouder lang niet die verantwoording. Maar er zijn, en dat is het, veel ouders die ja.. Onvoorwaardelijk in hun kind geloven. Dat is ook wel een bepaalde populatie hoor.

Z: Kijk, als ouders dan zouden zeggen ‘de energie van mijn kind is op en hij voelt zich niet goed’, dan vind ik het ook prima. Als ouders daar helder in zijn, dat is ook prima. Maar ziek melden en achteraf zeggen ‘ik heb toch gezegd ziek’, terwijl iedereen op zijn klompen aanvoelt dat ze of samen zijn gaan winkelen, want soms worden kinderen ook gezien door andere leerlingen of docenten die toevallig de stad in zijn.

D: Nou, ik denk, het is maar een inschatting, maar als ik het daar nu over heb, dan heb ik zelfs beelden bij die mensen. ‘Een beetje gezellig, een frikadelletje eten bij de passage’. Ja.. Ik zie ze gewoon.

Z: Ik denk dat het heel veel met niveau van opleiding te maken heeft.

D: Ja, hun eigen opleidingsniveau.

**Waarom vinden we het nu zo belangrijk om daar aandacht aan te besteden?**

D: Ja, ook omdat kinderen juist niet zien dat ze ‘boven hun ouders uit moeten komen’. Dat is een beetje stom gezegd, dat gaat ook niet altijd lukken.

Z: Dat gaat nooit lukken. Kijk, wij geven onderwijs, je wil dat kinderen hier zijn. Er is een leerplichtwet. Als de ouders hun kinderen vaak thuis houden, want die ‘diehards’ houden ze vaak thuis, dan onthoud je ze ook hun ontwikkeling, je onthoudt ze onderwijs.

D: Dus bedoel ik… Ze blijven op het niveau van hun ouders hangen.

Z: Dat is een heel moeizaam traject. Soms zitten we wel eens van ‘is dit nu geestelijke mishandeling, moeten we geen zorgmelding doen bij een aantal van die kinderen’. Want je onthoudt een kind ontwikkeling en onderwijs. We hebben hier in school wel een aantal voorbeelden. Bijvoorbeeld een meisje dat ook al veel bij jullie is geweest, een veelbesproken leerling. Die zijn heel tactisch verhuisd naar België, daar val je niet onder de leerplichtwet enzovoorts. Maar dan wordt er onderwijs onthouden. Dat kind is eindeloos ziek en als ze bij jullie vandaan komen, is het ‘medische stuk van de knie helemaal behandeld, maar heeft ze ineens diarree gekregen’. Met zijn allen, met alle volwassenen en deskundigen hier, staan we daar al drie jaar op een rij en we krijgen haar niet naar school.

**Wat doe je dan?**

Z: Nou, een zorgmelding werkt niet in België.

**Dan ga je wel ver als ouder.**

Z: Ja, je ziet soms ook patronen dat mensen van camping naar camping gaan. Dan heb je iedere keer een nieuwe gemeente en nieuwe leerplichtambtenaar. Iedere keer komt er een andere lpa. En de leerplicht begint altijd op 0. En wij weten die patronen wel, ja, uiteindelijk moet je dan een zorgmelding doen, dat doen we ook wel eens.

**Bij het AMK?**

Z: Ja, bij het AMK. Maar mijn ervaring in al die jaren is, dat het niets oplevert. Daar ben ik heel sceptisch over.

D: Ook nog bij een recent geval.

Z: Zodra ouders zeggen ‘ja, maar wij gaan morgen naar een psycholoog of we hebben ergens hulp, dat is vaak nog waar ook’. Dan zegt het AMK: mensen hebben hulp gezocht, dus die zetten vrijwillig stappen. Dan krijg je keurig een brief terug dat het is afgesloten.

D: Dan wordt het afgesloten. Wat ik ook nog wel een punt vind, en dat is niet om jullie beroepsgroep te… Ik vind, en daar moet je goed naar me luisteren, ik vind huisartsen in deze gevallen soms waardeloos. Als een kind snottert, zeggen wij ‘je moet wel met het kind naar de dokter gaan, als het zo ernstig is dat hij nu al een week thuis is’. Gaan ze naar de dokter en die zegt ‘ja, kind snottert, blijft nog een weekje thuis en dan moet het wel gaan’. Dan voelen ouders zich al rap gesteund door zo’n huisarts. ‘Maar de huisarts zegt… Hij is ziek, want de huisarts vindt het goed’. Als je het verifieert zegt de huisarts leuk ‘daar hebben jullie niets mee te maken’.

Z: Daar zijn we geen partij voor.
D: Ik vind huisartsen ook heel makkelijk in het thuis laten van kinderen. En er zijn echt ouders die met elk akkefietje naar zo’n huisarts gaan en die huisarts is dan alweer vergeten dat hij de vorige keer ook had gezegd dat het kind een week thuis mocht blijven. En nou is het een gekneusde voet, ja, een weekje met je been omhoog… En zo slijt er nog wel eens een patroon in. Dat huisartsen elke keer, 4 of 5 keer op een jaar, een kind thuislaten vanwege het feit ‘dat ze iets mankeren’.

**Want die informatie krijgen jullie via de ouders?**

D: Maar ik verifieer wel hoor. Ik durf te bellen naar huisartsen.

Z**:** *Nou, als ik de huisarts bel, krijg ik nooit een antwoord. Dat is terecht ook, denk ik. Maar daar zijn we als school geen partij voor. S_9a_*

D: Dat is ook jammer, dat wil ik jullie meegeven.

**Nou, dat is ook een meerwaarde van de jeugdarts neem ik aan. Die is wel een partij voor de huisarts.**

D: Ja, precies. En dat moeten we meer doen.

Z: In praktijk loopt dat ook nog niet zo. ‘Die was er toen niet, en toen dit en dat’. De communicatie loopt nog niet zo goed.

**Nee, het is moeilijk. Ik heb binnenkort een overleg over de bereikbaarheid onderling. Dat wordt nu ook inzichtelijk.**

Z: Want dan heb je een zorgteam en 6 à 7 weken later heeft de schoolarts nog geen contact kunnen leggen met die medici. Dan heb je nog geen beeld of dat klopt.

D: Kijk, een gebroken arm is geen reden om niet naar school te komen. Ja, de eerste week misschien, maar dat zijn allemaal van die dingen. Een arts kijkt vanuit de medische hoek, die kijkt echt anders, is mijn ervaring. Kijk, ik zei altijd over de huisarts als ik ging met mijn zoon ‘luister, we luisteren wat je hebt, maar niet wat hij zegt’. Want die zei altijd ‘zo, die astma, ik zou hem toch echt een week in bed houden’. Dan dacht ik ‘dat gaat niet gebeuren, hij gaat gewoon morgen naar school, want ik bed gaat die astma niet over’. Dus mijn ervaring als moeder zelf, is dat ze heel makkelijk…

Z: En een ouder die daar het gewicht legt..

D: Ja, die kijkt alleen vanuit de medische hoek.

**Die kijkt gewoon alleen maar naar diagnose, behandeling.**

Z: Ouders die daar gevoelig voor zijn, die zeggen zomaar ‘oh, twee weken’, want het is na 1 weekje nog niet goed en de dokter zei dat je toch wel heel goed beter moest zijn.

D: Zo is het. Of zelfs op voorhand. We hebben dat met collega’s nog wel. Bij een operatie zeggen ze op voorhand ‘ik word geopereerd en de huisarts zei dat het wel minstens twee maanden ging duren’. Dan duurt het ook twee maanden!

**Het is maar de vraag of de huisarts dan ook bedoelt dat hij ook twee maanden thuis moet zijn. Het is het verhaal van de ouders / collega dat je terug krijgt van wat de huisarts gezegd zou hebben. Maar de ouders weten ook dat jullie geen informatie krijgen.**

D: Wat ik eigenlijk wil zeggen is dat het gesprek dat jullie met de huisartsen aangaan, dat het een cruciaal punt is.

**Dat is heel goed, staat genoteerd.**

Z: Nou, hij moet beseffen wat voor consequenties eraan vastzitten. Van een goedbedoelde opmerking misschien en hoe ouders luisteren. En zeker bepaalde ouders die, waar we het toen straks over hadden, die doelgroep, die het ontzettend gezellig vinden als ze hun dochter thuis hebben.

**Zien die ouders het belang van naar school gaan wel?**

D: Nee, ja. Sommige ouders zeggen ‘ja, ik heb zelf ook geen school gedaan’.

Z: Kijk, je vertelt ze wel dat het ernstig is en ze lopen achter en inhalen… Dat vertel je ze heel duidelijk.

**Maar dan?**

Z: Een is dat ouders heel vaak teruggeven dat ze niet de baas zijn over hun kind. ‘Ik kan wel zeggen dat hij moet gaan, maar dat doen ze niet’. Dat is een belangrijke. Ouders hebben niet meer het gezag. Ook niet over ’s avonds naar bed gaan en over slaaptijden enzovoorts. Ouders zijn dan over puberkinderen niet de baas. Althans, die ik dan spreek. Die zeggen ‘ja, dat klopt, dat vinden wij ook, maar we krijgen het niet voor elkaar’.

D: Ja, die heb je ook.

Z: Dus dan moet je eigenlijk opvoedingsondersteuning hebben. En er is een groep ouders die zoiets hebben van ‘ja, ik heb school ook niet afgemaakt of daar werd ik ook onterecht behandeld of daar zaten ze ook te zeuren’.

D: Het negatieve schoolverleden.

Z: Dat dragen ze mee. En dan zie je vaak in die gezinnen dat er eigenlijk niemand een opleiding afmaakt. Ja, ach, daar liggen ze niet wakker van. Of ze wel of geen diploma halen. ‘Dan zien we wel’.

D: Je hebt die pedagogische onmacht, die ouders, die zitten in alle rangen en standen hoor.

Z: Ja, absoluut.

**Zie je dat meer bij de groep die zich vaak ziek meldt? Is daar een relatie?**

D: Die zie ik ook wel.

Z: Ja, want die slapen heel slecht. Ouders die hun kinderen niet naar bed krijgen, bijvoorbeeld zitten te gamen tot 3 uur vannacht.

D: Of zitten te ‘chillen’.

Z: Die melden zich vaker ziek, kunnen ’s ochtends niet naar school, komen ook vaak te laat.

D: Die zetten ouders onder druk. Zeggen ‘ja, ik ben nu ziek, ik ben nog niet wakker hoor’. Dan denken ouders ‘dat durf ik niet tegen de school te zeggen, dus ik meld ze maar ziek’.

Z: Eigenlijk moeten die mensen opvoedingsondersteuning. Dat advies geef je dan ook regelmatig. Maar dat gaan ze zelden doen.

**Ja, dat vindt die zoon niet goed.**

Z: Ja, dat vinden die ouders ook lastig.

D: Toen ik die oudercursus gaf, dat weet ik nog wel, dan kwamen ze thuis en zeggen die kinderen ‘je hebt zeker gisterenavond oudercursus gehad’. Dan kwamen de ouders met goede zin thuis en dachten ze..

Z: Nu gaan we het eens zetten, belonen en straffen.

**Dat werkt niet. Je zou eerder moeten beginnen, denk ik.**

Z: Ouders vinden het vaak ook een brevet van onvermogen. Die willen niet dat jeugdzorg daar binnen komt, want dan falen ze als ouder om iets aan te pakken. En dan blijft het verborgen. En wij weten het, je bespreekt wat ze moeten doen. ‘Ja, nee, maar jeugdzorg.. dan komen er allerlei smoezen’. En dat is die groep ouders die heel moeilijk te bereiken is, tot een kind niet over kan of zegt ‘nu moet je van school af’. Dan wordt het vaak wel ineens een ‘item’ en dan komt er leerplicht bij en krijgen ze nog een straf of proces-verbaal. En dan zie je soms wel wat beweging komen. In die gevallen ben ik blij als de rechter zegt dat er bijvoorbeeld een verplicht iets moet komen. Ik ben altijd blij voor die dwingende kant, want de rest is allemaal vrijwillig. Dan gaat er vaak niets gebeuren. En als de rechter zegt ‘je hebt zo verzuimd en je hebt al 2 keer een verbaal gehad, je moet nu naar de psycholoog’, er zijn wel eens van die trajecten die uitgezet worden. En dan gebeurt er wat.

D: En daarin kan zo’n M@ZL project natuurlijk een rol spelen. Die arts kan ook onderschrijven dat het kind niet echt ziek is of ‘in de juiste zin van het woord’, maar dat er allerlei andere dingen aan de hand zijn. Dan heb je dus meerdere partijen die naar zo’n situatie kijken.

Z: En die de doorverwijzing ondersteunen. Als je denkt ‘hier zou de jeugdarts eens naar moeten kijken, want dat kind, dat patroon, er klopt iets niet’. Dan vermoeden wij het als school wel eens, maar dan moeten de ouders naar de huisarts waar ze hun eigen verhaal moeten vertellen in hun eigen woorden. Soms geven we een brief mee als ouders dat willen waarin wij beschrijven wat we vinden. En dan moet je verwachten dat er een doorverwijzing komt, soms denkt een huisarts daar anders over, dat kan natuurlijk. Maar als zo’n schoolarts ook al denkt van ‘ja, je moet toch eens kijken naar hulpverlening thuis als jullie het niet voor elkaar krijgen’ en de school zegt dat ook.. Dan kun je misschien toch wat meer druk uitoefenen. Dat daar toch eens een tijdje iemand via jeugdzorg in de opvoeding kijkt. Van ‘hoe maak je afspraken met pubers en hoe doe je dat’.

D: Bij die jongere kinderen…

**Want via een zorgmelding werkt het eigenlijk niet echt?**

Z: Nee, daar ben ik heel sceptisch over.

**Heb je ervaringen dat het via de leerplichtambtenaar wel werkt? Dat is een andere route die je kan doorlopen hè.**

D: Ja, nog eerder volgens mij als via het AMK.

Z: Ja, via de leerplichtambtenaar..

D: Maar wij hebben ook niet zo’n sterke leerplichtambtenaar.

**Zo’n lpa zou dan een proces-verbaal kunnen opmaken en dan krijg je toch ook mogelijkheden.**

Z: Ja, voordat hij een verbaal schrijft, kan hij ook in de gesprekken aangeven van ‘jullie moeten toch eens dit en dat doen, ga eens naar de jeugdzorg’. Nou, dan bieden wij hulp met ons SMW om die zorgvraag te formuleren met de ouders, eventueel mee naar jeugdzorg te gaan als ze het zelf niet kunnen. Maar het hangt heel erg van de lpa af, dat is heel verschillend. We hebben gemeenten daar zitten ze erbovenop. Daar gaan ze aan huis staan bellen als een kind er weer eens niet is. Die gaan het met de ouders helemaal oppakken (bijv alphen-chaam, zundert enzovoorts), maar hier in Breda, hebben ze een beleid van ‘niet te strikt en niet te snel straffen, maar meer ouders langzaam de goede kant op proberen te bewegen’. Nog eens een gesprek, nog een gesprek en nog eens een waarschuwing.

**Wat jullie eigenlijk ook al gedaan hebben.**

D: Eindeloos, dat hebben wij natuurlijk allemaal al gedaan.

Z: Maar goed, we hebben hier met het hoofd van de lpa zitten praten, dat is hun beleid. Oké, daar hebben wij geen invloed op.

D: En dan bepaalt zo’n leerplichtambtenaar eigenlijk of ze ziek is, ja of nee. We hebben een leerling en die moeder houdt ze dus voor elke smoes thuis en ze komt ze hier ook regelmatig halen als ze ‘weer ziek is’. Niemand kan aangeven dat ze ‘echt ziek is’.

Z: Die moeder is 1 keer bij de schoolarts geweest, daar ruzie gekregen, die maakt met iedereen ruzie, want die moeder is zelf een psychiatrische patiënt. Dus die zegt ‘ik ga nooit meer naar die akelige mensen’. Die heeft daar flink ruzie gemaakt, dus die komt niet meer. Daar moet de leerplicht dus iets doen. Dan blijkt dat ze bij de kinderarts in het ziekenhuis te zijn. Uiteindelijk blijkt dat ze gesprekken heeft met een psycholoog, dus begeleiding, dus gebeurt er niets meer. Maar het verzuim is even groot. Maar ze heeft ondertussen gesprekken met een psycholoog…

D: En dat is het, ze verleggen het probleem.

**De leerplicht gaat dan wel naast moeder staan?**

D: Ja, in dit geval was dat. Wij hebben als school niet gereageerd op die brief van die moeder. Alleen ben ik daarvoor wel bij de leerplicht geweest.

Z: Kijk, die schakel, die schoolarts – leerplicht – school, dat moet natuurlijk een hele goede driehoek zijn. En anders hebben ouders heel snel door ‘waar je kunt ontsnappen’.

D: Je moet een front maken ja, dat klinkt heel hard, maar dat is gewoon zo. We moeten samen uitdragen. Nu is één van die partijen een beetje, ja, bang van die moeder denk ik. Maar goed, die is aan de kant van die moeder gaan staan. Nou, als je het verzuim van dat meisje ziet, elke keer een andere pijn. En bij de jeugdarts verschrikkelijk ruzie gemaakt. En hier op school ook. Ja, tijden zijn veranderd.

**Zit de leerplichtambtenaar bij jullie ook in het ZAT?**

Z: Ja, die heeft ook met de schoolarts steeds de schakel. Maar ik zeg al, hun beleid, en dat is ons nog eens uitgelegd, is toch meer op het ‘preventieve, het goede gesprek’ gericht. Maar dat doen wij hier al. Andere scholen weet ik niet. Maar wij investeren heel veel in… via leerlingbegeleiding, dat zijn we van oudsher gewend, want wij waren vroeger speciaal voortgezet onderwijs, dus je investeert heel veel in die dingen.

D: Goede gesprekken.

Z: Maar als dat niet meer werkt, moet iemand anders zeggen van..

D: Ho, stop.

Z: En dat merk je dus, ik had vorig jaar een meisje bij Alphen-Chaam, en die mevrouw riep ze iedere week nog op daarna. Of het liep en dit en dat.

D: Controle.

Z: Ja, er bovenop zitten.

D: En in dit geval, wat ik zei.. Ja, bij dat andere geval heeft moeder gewoon het gevoel dat ze gelijk heeft gekregen van de leerplichtambtenaar. Dan kun je helemaal niets meer.

**Zou de jeugdarts in dat stukje nog meer kunnen betekenen?**

D: Nou, in dit geval zou ik er als jeugdarts niet aan beginnen. Maar in andere gevallen..

**Want de jeugdarts zegt natuurlijk dat hij niet van de controle is. Die hoort eigenlijk bij de lpa thuis. Die moet controleren.**

D: Wij hebben een hartstikke goede jeugdarts. Kijk, onze jeugdarts doet wat ze kan natuurlijk. Die is best straight daarin.

Z: Ook als je weigert vind ik, om daar te komen. Die moeder die zegt ‘ik ga daar niet meer naartoe’, dan moet het eigenlijk bij de leerplicht komen. Dan bespreken we in het ZAT.

D: En dan laat de lpa zich inpakken door die moeder..

Z: Die zegt ‘ik ga daar nog een gesprek aan’. Maar ik was daar ook al gaan praten..

**Er zitten veel verschillen tussen lpa’s?**

D: Ja, bij de leerplicht zit echt wel een puntje.

**Maar ook een verschil binnen de school hoe het wordt opgepakt door mentoren?**

Z: Ja, dat hebben we nu afgesproken. Binnen de criteria, de mentor doet dan dit, dan dat. Dus een kind dat drie keer een dag ziek is in een periode X, dan gaat de mentor bellen. Is een kind meer dan 5 dagen ziek, gaat hij ook bellen. En die legt dat vast. Dan komt de leerlingbegeleider.. Daar hebben we nu dus een heel protocol voor. Dus dat doet iedere mentor, die loopt dat af. Dat was naar aanleiding van ‘maak zelf de criteria’. Dan moet je nog wel eens tegen mensen zeggen van ‘god, had je in de gaten dat dat kind zoveel ziek was’. Ze krijgen iedere week of iedere 14 dagen een overzicht van het verzuim van zijn leerlingen, van zijn klas. Dus die kan meteen zien van Z = ziek, LA = te laat. Zo’n uitdraai. Dus iedere mentor, die moet er ook voor tekenen dat het klopt, want soms is het niet goed genoteerd in het klassenboek of een docent wist niet waar een leerling was. En dan moet hij het controleren, checken of de codes kloppen en zo nodig verbeteren. Maar dan heeft hij dus ook meteen gezien van ‘hee, mijn leerlingen van deze week, oh, die is wel vaak ziek’.

**Hoe wordt het opgepakt door de mentoren? Hoe vinden zij dat het gaat?**

Z: Nou ja, die hebben zoiets van ‘het is best lastig’. Ze moeten er veel achteraan. Maar dat hoort bij de mentortaak. We hebben afgesproken ‘je belt, je gaat niet in discussie over dat ziek zijn, maar zegt ‘goh, meneer of mevrouw, ik merk dat uw dochter al voor de 3^e^ keer een dagje ziek is en steeds op maandag’. Heel lastig, vervelend, komt ook achter te liggen. En in eerste instantie reageren ze begripvol en kijken ze of je hulp kunt bieden. Is er iets waardoor we kunnen zorgen dat ze.. Is er op maandag iets, gymles misschien, dat het probleem is? Of een kind dat al de hele week ziek is, bellen ‘goh, kunnen we misschien lesstof’, misschien is ze al aan de beterende hand, dat ze toch al wat werk kan maken. Anders lopen ze nog meer achter. Daar zit natuurlijk wat cynisme achter. Maar oké. En als het patroon blijft, dan gaat de leerlingbegeleider die mensen uitnodigen. Dan zeg ik of mijn collega van ‘luister, beste ouders, we moeten toch eens rond de tafel’.

**De leerling-coördinator doet dat dan of de mentor zelf?**

Z: De mentor heeft ze dan al gesproken en die ziet ze op de ouderavond. Dus de leerling-coördinator doet het dan. Dan geef je meteen de boodschap dat de volgende stap de jeugdarts is. Dat leggen we dan even uit, dat het is vanuit zorg en dat een kind zo goed mogelijk de les kan volgen. Dat is het doel van ‘wij bieden hier les en onderwijs en dat stokt gewoon’.

**En hoe gaan die gesprekken? Is het moeilijk om ze te voeren?**

Z: Nee, tenminste. Dat doen wij al heel lang, dus ik denk dat we ‘door de wol geverfd zijn’. Je kent die patronen ook. Die mensen proberen allerlei andere wegen uit. Dat weet je van je collega al en dan doen we bijvoorbeeld met 2 mensen een gesprek, met de afdelingsleider erbij. En dan nog…

**Dan sta je sterker.**

Z: Dan begin je een gesprek dat een kind ziek is vanwege een operatie aan de knieën en dan zeggen ze ‘ja, maar ze heeft ook nachtmerries’, weet ik wat. Dan zeg je dat ze toch naar de schoolarts moeten, dan krijg je eerst allerlei verhalen. Uiteindelijk gaan ze dan toch, pas 6 weken later door allerlei smoesjes (allemaal niet waar), naar de schoolarts. Uiteindelijk gaan ze dan, bellen ze me terug als ze daar geweest zijn, boos… ‘Ik moest tien minuten wachten, belachelijk, ik ga er nooit meer naartoe, bovendien kenden de mensen het dossier niet goed want ze stelden allemaal weer vragen’. Die mensen wilden niet terug vertellen, die had zoiets ‘bekijk het maar’. Dan zeg ik ‘ja, ik stuurde jullie voor die nachtmerries’, dat was de reden dat ze overdag niet naar school kon, omdat ze uitgeput was ’s ochtends. Ik zeg, dan kun je toch schoolwerk maken via de computer als je thuis in bed ligt, daar hebben we een systeem voor. ‘Nee, want ze lag dan tot half 2 uitgeput op bed ’s middags’. Dan vraag je of ze dat nu hebben besproken bij de jeugdarts. ‘Ja, maar, komt er een heel verhaal.. GGZ, dat willen ze niet’. Ik zeg, maar maandag komt ze. Bellen ze toch maandagochtend op dat het meisje diarree heeft gekregen..

D: Dan durf je toch wel, hè.

Z: Dat kind is zo dik, die blijft haar hele leven nog in bed liggen bij mama. Enigskind. Mensen hebben alle twee geen opleiding. Die man is ook werkloos, weer een baantje, dan weer ontslagen. Daar komen wij nooit een steek mee verder.

D: In zo’n systeem niet.

Z: Daar ligt al een medisch onafhankelijk advies.. We hebben er dus een paar in een school, zo’n 4 of 5 van die trajecten, dat ga je nooit veranderen. Ik denk dat in zo’n school van 370 kinderen, heb je er 5, dat ga je met heel hulpverlenend Nederland niet redden. En die kosten ons wel heel veel energie, dat is eigenlijk het vervelende.

D: Weet je wat ik wel vind. Je moet het wel zo zien.. Als je niets doet, dan komen ze helemaal niet.

Z: En het is ook een verkeerd signaal naar anderen.

D: En dan heb je nog meer.. Als het maar laat, dan denk die andere ‘nou, dat kan makkelijk, ik blijf ook eens een dagje thuis met de chips en cola’.

**Want waarom moet je aan ziekteverzuimbegeleiding doen op school?**

D: Nou, ik denk omdat kinderen recht hebben op ontwikkeling en scholing voor hun toekomst. Dat denk ik, misschien is dat ook wel heel bizar gedacht en moet je denken ‘ja, we gaan allemaal dood, dus waarom zouden we studeren’. Ja, zo kun je denken, maar dat is wel heel kort door de bocht. Ik denk, een stukje ontwikkeling, een toekomstperspectief. Je hoeft niet allemaal huisarts te worden, maar..

Z: Die kinderen zijn kansloos als ze geen MBO diploma niveau 2 halen. Dan kom je nergens op de arbeidsmarkt. Dan ben je tot je 21 nog een goedkope arbeidskracht die ze nog inzetten in de winkel of het magazijn en als je dan 21 bent, moet je een volwassen loon krijgen en knikkeren ze je eruit. Dan hebben ze weer een nieuwe van 16 jaar die niets heeft. Zo gaat het, die kinderen zijn vanaf hun 21^e^ kansloos. Dat is gewoon zo.

**En dan krijgen ze kinderen.**

Z: Die gaan heel hun leven zo..

D: En dan krijg je weer dat.

Z: Dus je wil toch dat ze dat niveau halen. En er is een leerplichtwet.

D: Er zitten soms zo’n slimme en in potentie ook leuke kinderen bij, waar je ‘iets mee kunt’. Kinderen zijn altijd leuk, maar ik bedoel dat je.. Bijvoorbeeld een kind uit een achterstandswijk dat erg slim is, creatief en dan zegt moeder ‘hij stopt ermee, hij gaat werken’. Die kwam uit zo’n systeem, dan heb ik het wel over 20 jaar terug, misschien kon dat toen nog, die moest gewoon werken. Zo jammer. Uiteindelijk zit hij weer in dat systeem van die ouders. En hoewel ik daar gepraat heb en hij het achteraf zelf jammer vond, zei die moeder toch dat hij niet meer naar school mocht.

Z: De loyaliteit naar de ouders toe is altijd groter dan naar de school. Dat is nu eenmaal zo. Dat blijkt ook uit de psychologie. Die is ijzersterk. Dus die kinderen, ook al weten ze wat ze moeten doen, als moeder heel snel zegt van ‘blijf maar thuis en ach..’, dan gaat het niet gebeuren.

**De ouders hebben veel meer invloed dan de school.**

D: Dan wordt dat de waarheid. De waarheid is wat je moeder of vader zegt.

Z: Dus dan zou je, als die ouders bij de jeugdarts op bezoek komen, het accent moeten leggen op de ontwikkeling van het kind. Dat die heel erg belangrijk is, want school betekent ook ontwikkeling.

En dat betekent perspectief. Hoe meer je mist…

D: Daar moet je dus ook op bouwen tijdens de voorlichting.

Z: Dat de ouders daar de verantwoordelijkheid in gaan voelen. Het moet bij de kant van de ouders liggen van ‘god, wat doe ik mijn kind aan’. Het is een systeem.

D: Dat is eigenlijk wat we van het begin al zeggen.

Z: En die andere filteren we er wel uit met ‘zoveel keer ziek naar de dokter’. Laatst ging een mentor ook bellen naar een kind dat voldeed aan de criteria en toen bleek dat het kind werd gepest op de andere locatie. Moeder had het kind wel ziek gemeld. Daar komen dan hele verhalen uit. Dan blijken er nog allerlei dingen, dat het thuis ook niet goed gaat in de gezinsrelatie. Oké, dan steken we daarop in, dat is prima, fijn dat we het weten, dan gaan we daar gesprekken over voeren.

**Maar dan ben je er wat sneller bij.**

Z: Door dat bellen, door het stappenplan. Maar moeder heeft hem wel ziek gemeld. Die had het ook afgedekt. Dat weet ze, ja, dat is jammer.

D: Met die mensen kun je wel verder komen..

Z: Pas na 14 dagen komt moeder pas met het echte verhaal. Dat zal bij jullie ook voorkomen denk ik. Dat ouders zeggen ‘ja, het klopt, ze is eigenlijk ook niet echt ziek, maar’. En dan kun je stappen zetten.

D: Als mensen zo ver zijn, dan kun je er natuurlijk wat mee.

**Je zei net al ‘ja, dat was 20 jaar geleden’, is er sindsdien wat veranderd?**

D: Ja, nee.. Ik vond toch dat je meer met de ouders kon. Of ik kon meer met de ouders, dat weet ik niet, want ik bemoei me er nu niet meer mee. Voor mijn gevoel kon je… was je als school ook nog meer een autoriteit. En kon je dus, als je op huisbezoek kwam of je liet ouders komen, dan dachten ze ‘wauw, moeten we naar school komen en we gaan over mijn kind praten’. Het verzuim was sowieso veel minder. Dus ja, als school had je meer invloed, je was ‘iemand’.

Z: We gingen altijd op huisbezoek, dat doen we niet meer. Ja, er zijn er nog wel die het doen, maar niet meer als onderdeel van je werk. Dat kon niet meer toen de normjaartaak ingevoerd werd.

D: Maar goed, je hebt nu ouderavonden.

Z: Maar op die huisbezoeken was je wel meer een gezicht, je ziet het systeem thuis, je raakt met elkaar vertrouwd. Toen was het wel zo dat wat de juf of meester zei, dachten ouders ‘god, dat heeft de juf of meneer gezegd, dus dat moeten we wel gaan doen’.

D: Dat bedoel ik, je had meer autoriteit als school. Nu wordt er wel geroepen ‘de school moet mee opvoeden, is medeverantwoordelijk’. Ik vraag me altijd af of ouders dat ook vinden of dat ze denken ‘het is wel makkelijk dat ik het bij school leg’. En het onze verantwoordelijkheid maken. En ik vond wel dat ik het gevoel had vroeger dat het onze gezamenlijke verantwoordelijkheid was als een kind niet naar school kwam. En het voorbeeld dat ik net gaf, dat was heel opvallend, omdat dat eigenlijk nooit voorkwam. Toen ik hier kwam werken, ik was 25 jaar, en ik zei ‘ik wil dat er gezond gegeten wordt, ik wil dat jullie bruine boterhammen eten op de eerste ouderavond’, dat wil ik elke middag zien. En het kwam. En nu denken ze ‘ja, zeg, daar heb jij niets mee te maken’. Een zak chips en twee bananen is ook goed. En daar zit ook wel een verschil.

Z: Nou, ik heb het gevoel dat ouders minder te zeggen hebben over hun puberkinderen. En dat botste vroeger ook, dat waren flinke scheldpartijen, maar er gebeurde nog wel wat de ouders zeiden. Zoals naar bed gaan..

D: Ja, de ouders hadden meer te zeggen.

**Is dat dan de oorzaak van dat ouders het nu ook maar bij school leggen?**

D: Ja, ik snap wat je bedoelt.

Z: Ik heb twee jaar terug een ouderavond gehad en toen had ik 4 of 5 gesprekken op een rij die eigenlijk alleen maar gingen over opvoeding. Ze hadden slechte cijfers enzovoorts, maar dan bleek dat die kinderen gewoon niet naar bed gingen ’s avonds. TV kijken, gamen. En in alle gevallen hadden ouders, dat waren er 4 op een rij zo, niets over te zeggen. ‘We gaan zelf maar naar bed, want hij luistert toch niet’. Dan zeg ik ‘ja, je kunt een nijptang pakken en de stekker eraf knippen van de computer of tv’, maar dat kan niet. Ouders geven het dan maar op en ze laten het bij school liggen. Dan komen wij ‘zeuren’.

**Bang voor ruzie?**

D: Ja, heel bang, je krijgt geheid ruzie in de tent.

**Ouders willen leuk gevonden worden.**

D: Precies.

Z: Dat lees je wel eens hè, de weinige tijd die ze doorbrengen, moet leuk zijn. Gezellig.

D: Ouders kunnen geen ‘nee’ meer zeggen.

Z: Dat geldt ook voor studie. Huiswerk maken, dat gebeurt niet in heel veel gezinnen. Als je dan zegt dat je je kind moet aanspreken, krijg je terug ‘ja, dat zeg ik wel eens, maar hij doet het niet’. En dan? Dan houdt het op.

D: Want anders hebben ze elke avond ruzie. En ouders die zich daar ook zorgen over maken, waarmee wij wel in gesprek gaan, dan kun je zeggen ‘ja, jullie moeten ook ouder zijn, je moet geen politieagent zijn’. Sommige ouders vinden dat ook gewoon moeilijk. Sommige kinderen zijn ook echt vervelend thuis, dan snap ik het ook wel. Het kind is gewoon ‘lastiger’ dan 20 jaar geleden.

**Dan kun je samen met school een oplossing zoeken.**

Z: Nee, kinderen zijn niet lastiger. Er zijn andere mogelijkheden voor kinderen. Allemaal een computer en televisie op de kamer.

D: We leven in een andere wereld.

Z: En het pingen (gratis sms’en). Kinderen pingen elkaar en verwachten ook weer een reactie terug. Ze liggen ’s avonds, gerelateerd aan ziekteverzuim, te wachten totdat iemand weer een berichtje terug stuurt. Vooral meisjes worden heel onzeker als ze geen reactie terug krijgen: ben ik niet belangrijk? En als je dan ook nog een computer en TV op je kamer hebt. Er zijn andere mogelijkheden gekomen voor kinderen.

D: Ook nog muziek in je oren.

**Wat erg..**

Z: En ’s ochtends schelden ze elkaar hier uit, dan hebben ze ruzie over wat er gepasseerd is. Vooral met pubermeisjes heb ik dat soort gesprekken.

**Een enorme sociale druk.**

D: Dat vind ik wel heel erg. Op alles. Ze weten alles van elkaar. Het is ongelofelijk. Ze zetten alles op facebook of msn.

Z: Die mogelijkheden die er zijn voor de jeugd.. De jeugd is niet anders, daar ben ik altijd van overtuigd. En de ouders staan anders in het leven.

D: Kinderen hebben een ander sociaal leven.

Z: Ouders stellen gewoon minder eisen thuis. Dat heeft te maken met kinderen naar school sturen, met huiswerk, met al die dingen. Dat is allemaal lastig.

**Als we kijken naar M@ZL, dan hebben we al een paar valkuilen genoemd, bijvoorbeeld over het samenwerken. Zijn er nog meer valkuilen? Dingen waar we op moeten letten? Waar we scholen voor moeten waarschuwen als ze beginnen?**

Z: Het enige wat ik bedenk is dat je aan de ouders vooraf goed uitlegt dat het uit zorg is, want we hebben in het begin, of afgelopen jaar in het traject ook wel eens, dat mensen kwaad zijn als ze naar de schoolarts moeten. Dat zien ze als een straf. Je gaat ze als school opeens naar een dokter sturen. Wie ben jij wel? Ik heb toch gezegd dat ze ziek is, dus.. Dus als je van tevoren heel goed uitlegt dat het preventief is en uit zorg. Ik zeg altijd ‘ik ben geen dokter, gelukkig, dus dat mag iemand anders vaststellen en daarom stuur ik u daar naar toe’. Dat is uit zorg, want uw kind heeft te weinig les.

Je moet dat heel goed bij ouders inmasseren. Dan voorkom je een hoop weerstand. Want als ze bij de schoolarts komen en je moet eerst die weerstand nog overwinnen, dan ben je de eerste 20 minuten van je gesprek al kwijt. Dan beginnen ze op school te schelden en op bijvoorbeeld die stomme leerkracht.

D: Of een goede folder…

Z: Dus dat is nog steeds een punt van aandacht. Ouders heel goed uitleg geven en aangeven dat het vanuit een positieve insteek is. Uit zorg.

D: Een folder die je ouders meegeeft.

**En heb je daar de gelegenheid voor?**

Z: Ja, wij ontvangen ieder jaar alle nieuwe ouders. En daar is dat een onderdeel. Maar goed, of dat blijft hangen.. Ik denk dat je het moet herhalen. Dat het steeds weer.. Of een goede folder die je meegeeft aan die mensen.

**Ja, dat is een goede.**

D: Je moet daar mee bezig blijven denk ik.

Z: Een goede folder is wel belangrijk. Vriendelijke folder, maar wel met precies het stappenplan. Met de risico’s.

**En dat doel vooral van het project denk ik.**

Z: Maar vooral het doel, uit zorg.

D: Het doel, waar je terecht kunt komen.

Z: Als je dat als ouders hoort of in de schoolgids leest en je kind is niet ziek, dan heb je dat niet nodig. Maar misschien heb je het 1 of 2 jaar later wel nodig als je puberkind dwars ligt. Dan herinneren ze zich niet meer dat de school die stappen zet uit zorg. Dat ze ouders het niet als een straf zien, maar als een systeem van de school. Net als de sociale vaardigheidstrainingen.

**Maakt het voor de implementatie nog uit of het een kleine of grote school is?**

D: Een grotere school werkt weer met meer leerlingbegeleiding en meer zorgmensen.. evenredig verdeeld over het aantal leerlingen. Ik denk niet dat het iets uitmaakt.

Z: Sommigen hebben zelfs een speciale verzuimcoördinator. Die hebben weer een ander systeem. Je moet het systeem daarop afstemmen.

D: Wij kennen onze kinderen hier wel beter, ja, niet beter.. We hebben intensiever contact. Omdat de leerlingen elke week worden besproken. Daar hebben wij ook meer uren voor, omdat we een soort zorglocatie zijn. Ze komen ook anders binnen, er zit een ‘zwaarder kaliber’.

Z: Wij hebben met alle ouders een intakegesprek als ze hier komen, van ongeveer 1 uur. Daar komt alles aan bod, dus dat is een ‘startdocument’. Ouders vullen zelf ook nog een heel document in over de eigenschappen van hun kind, maar ook over ziekte, medicijngebruik. Je hebt informatie van de basisschool. Dus we beginnen met een heel uitgebreid dossier. Het eerste contact met ouders is er altijd al geweest. Je weet soms al dat een kind iets medisch heeft..

D: En tijdens het onderzoek doen we ook een stukje persoonlijkheid bekijken en daar komen ook dingen uit. Bijvoorbeeld angstgevoelens voor school, buikpijn bij een proefwerk. Dat zijn allemaal signalen, die je eigenlijk op voorhand al weet. Daar zouden we wel iets meer mee kunnen denk ik. Met die open vragenlijsten. Vaak zeggen kinderen daar al dingen waar we attent op kunnen zijn. Misschien dat we daar iets meer de nadruk op kunnen leggen.

Z: Ja, in het kader van verzuim. Dus wij weten best veel van leerlingen als ze komen.

**De jeugdarts minder? Zou je dat kunnen verbeteren als de jeugdarts daar misschien ook inzicht in heeft..**

Z: Ja, ze mogen alles inzien. Ze zitten ook bij het zorgteam.

D: En de rapportages, daar staat uiteindelijk alles in.

Z: Ik denk dat wij wel voldoende informatie aanleveren aan de jeugdarts. Ook hoe een kind zich gedraagt en waar de spanningen liggen, dat weet ze allemaal. Dus die informatie is denk ik meer dan op een reguliere school.

D: We hebben een zwaarder kaliber kindjes en we zetten er ‘meer geschut’ op. Dus we hebben wel meer met lesverzuim te maken, denk ik, als je het afzet tegen een reguliere school.

**Dat zagen we ook in de pilot, en de afname van het verzuim was toen net zo groot. Maar jullie kwamen van hoger.**

D: Dat is wel goed.

**De conclusie was toen dat het al door het aandacht besteden aan.. van alles gebeurt.**

D: Natuurlijk. Van de 25 kinderen die verzuimen, heb je 3 kinderen waar je niet doorheen geprikt komt en dan heb er toch 22 over en daar zijn er misschien 15 waar je redelijk snel de vinger aan de pols krijgt en de rest, daar heb je wat meer werk mee. Zo moet je het ongeveer zien.

**Zien jullie zelf ook dat effect? De afname van het ziekteverzuim?**

Z: Dan zou je getallen moeten hebben.

D: Daar kan ik ook niets van zeggen. Ik geef geen les, ik merk ook niet als kinderen.. Ik weet het wel van de docenten (lacht).

Z: Maar ik zou wel kunnen kijken, de administratie kan dat wel bijhouden. Die kunnen dat uitzoeken. Maar goed, die kinderen zaten toen in klas 1 en die schuiven door. Die groep schuift door, dus je zou dan iedere keer in klas 1 moeten peilen.

**Of hoe die groep het 4 jaar lang blijft doen?**

Z: Het aandacht besteden, het in je systeem hebben, ouders het voordeel uitleggen.

D: Dat werkt ook wel hoor.

Z: Ik zeg altijd ‘het kan ook in jullie voordeel werken’. Stel dat de schoolarts zegt dat je inderdaad niet de hele dag naar school kan, nou.

D: Nou, als ouders weten dat ze én gebeld én naar de schoolarts én naar dit.. Dat werkt wel om minder snel dat kind thuis te houden. Want dan zeggen ze ja ‘je gaat mooi naar school, want morgen staat de leerplicht weer aan de deur, daar heb ik geen zin in, hup naar school’. Dus controle werkt altijd. Dat werkt ook bij docenten. Vroeger moesten die zich bij mij ziekmelden, dat was voor heel veel mensen een vervelende drempel! Je blijft dan niet zomaar voor ‘een scheet’ thuis. Nou is dat niet meer hoor, want ze moeten nu bij de afdelingsleider, misschien is dat ook vervelend. Maar ik weet wel dat ze er een hekel aan hadden om zich bij mij ziek te melden.

**Zouden we dat bij leerlingen ook kunnen doen?**

D: Ik denk voor leerlingen, doordat er meer controle is, dat ouders hun kind minder snel thuis houden. Want die vinden dat lastig, als de school er zo bovenop zit.

Z: Ik heb vooral met de 3^e^ en 4^e^ jaars te maken en zeker de 4^e^ jaars, die vaak ziek zijn, die laat ik mij persoonlijk bellen. Ouders bellen de administratie en de leerling belt mij. Als hij dat niet doet, is het ongeoorloofd verzuim. Je krijgt mijn 06-nummer en je belt mij zelf. Om die drempel te verhogen. En dat werkt soms ook. Dat werkt, tenminste dat is mijn ervaring, met kinderen die zo rond de 15 à 16 jaar zijn. Dan kun je daar die verantwoordelijkheid neerleggen. Moeder moet ook melden, maar ‘jij’ ook, aan mij persoonlijk. Niet via de schoollijn.

D: En dat werkt. Je moet zelf maar eens iemand moeten bellen.

Z: Want dan stel ik een vraag van ‘wat is er dan’ en ‘kun je dan niet vanmiddag komen’.

**Dan maak je het onafhankelijk van hoe zij met de ouders thuis omgaan.**

D: Je spreekt ze op hun eigen gedrag aan.

Z: Dat kan op die leeftijd ook wel denk ik. Dat gebeurt nu zo, ik denk dit jaar, bij 2 kinderen. Soms sms’en ze dan ook en zoeken ze een soort uitweg. Dus dan moet je het weer aanscherpen.

D: Dat vind ik zelf ook al. Als ik iets lastigs heb, doe ik dat liever via de mail als dat ik bel.

Z: Maar goed, dat was mijn manier om ze te controleren. Volgend jaar zitten ze op het MBO, dan moet je verantwoordelijk gemaakt worden voor je eigen handelen. Dat vind ik belangrijk op die leeftijd.

D: Zeker, ook als ze stages gaan doen, dan kun je ook niet elke keer zeggen ‘ik kom niet’.

**En kinderen bij de mentor laten ziek melden?**

D: Dat zou kunnen…

Z: Ja, maar niet iedereen..

D: Niet elke mentor zit erop te wachten.

Z: We hebben niet allemaal een bedrijfsnummer of een telefoon van de zaak. Ik ook niet.

**Dat is praktisch gezien niet mogelijk?**

Z: Nou, dan moet je weer doorverbinden via de administratie, daar schiet je niets mee op.

D: Dat is geen drempel, ook voor docenten niet.

**Nee, maar een mentor wel?**

D: Ja, wel meer.

**Maar dan moeten ze allemaal een mobiel van het werk hebben?**

Z: Ja, het kan ook niet als die in de les is. Dan moet er al voor schooltijd gebeld worden, nou, dat gaat allemaal niet gebeuren. In de praktijk gaat dat niet goed werken.

D: Er zijn individuele docenten die dat prima vinden, maar er zijn er ook die niet zomaar gebeld willen worden bij nacht en ontij.

Z: Dus afhankelijk van de leeftijd, kun je kinderen ook meer zelf verantwoordelijk maken.

**De inspecteur komt bij jullie om te controleren hoe jullie dat allemaal doen hier. Hoe jullie de kwaliteit garanderen. Speelt M@ZL daar een rol in? Is dat iets wat je binnen je kwaliteitssysteem kan aanhalen/wegzetten?**

D: Ja, maar toen de inspecteur de laatste keer kwam, waren we nog net niet ingestapt het M@ZL project.

Z: Nou, we deden al jaren mee..

**Maar stel dat hij nu komt, jullie worden eigenlijk op een aantal zaken ‘afgerekend’ en beoordeeld.**

Z: Ja, ik zit even te denken of zij ernaar vroeg. Op kwaliteitssystemen en dat soort dingen.

D: Ja, ze weet wel dat wij dat doen.

Z: Ik weet niet of het besproken is, want ik heb ook altijd een gesprek met hun over zorgleerlingen, handelingsplannen.. Dus het zou wel kunnen.

**En dat valt onder je kwaliteit van zorg leveren? Ziekteverzuimbegeleiding?**

D: Ja, natuurlijk, daar valt het wel onder. En wij hebben daar een goed systeem voor. We hebben het goed bijgehouden en kregen daar een compliment over.

Z: Ze vragen meer of je een systeem hebt, iets als Magister en hoe je dat doet enzovoorts. Maar of ze echt naar M@ZL vragen..

D: Als ze nu komt, zou je dat meenemen. Dat is logisch toch.

**Jullie hebben als school toch een plicht om te signaleren.**

Z: Ja, absoluut, dat is een plicht die je hebt. Een signaleringssysteem.

**Ja, er zijn ook directeuren die zeggen ‘het staat helemaal niet in mijn top 10, ik word daar niet op bevraagd’.**

D: Jawel, dat vragen ze hier wel. Omdat het ook een zorgschool is. Ze vragen welk systeem je hebt, wat je doet.

**Maar ziekteverzuim is natuurlijk geoorloofd..**

Z: Ik zie dat anders. Zo kan je het zien. Maar wij zien dan vaak ‘er zit iets anders achter’. Achter een deel van het ziekteverzuim. Ik denk best een groot deel. Dus is het een deel van je zorginstrument. En in het samenwerkingsverband Breda – Ettenleur – Oosterhout, waar alle scholen in vertegenwoordig zitten, is afgesproken dat je 9 punten van basiszorg levert. En signaleren is ook een van de punten. Dat is niet precies uitgesplitst.. Maar in die zin vind ik het daaronder vallen, dat je signaleert dat een kind regelmatig ziek is. Je signaleert van allerlei andere dingen, dat hij sociaal onhandig is en weet ik wat. En met al die signalen moet je natuurlijk aan de slag, daar moet je een systeem voor hebben. Je hebt leerling-besprekingen, scorelijsten en je hebt je ziektemelding, daar moet je ook achteraan. Uit zorg.

**Maar het is niet op elke school zo belangrijk.**

D: Bij ons is ziekteverzuim toch een punt van zorg. Wij moeten proberen om de kinderen die al weinig hebben, dat ze …

**Zou het niet op alle scholen een punt van zorg moeten zijn?**

Z: Kijk, een week griep, dan ben je ziek, dan hoef je niets.

D: Als je ziek bent, ben je ziek. Dat geldt voor iedereen.

Z: Maar wij gaan achter het verzuim aan, waar je twee keer een week ziek bent, en nog eens, of iedere vrijdag of maandag. Het ‘signaalverzuim’. Die zijn niet echt ziek.

D: Dat doen wij wel ja.

Z: Dus dat zou een onderdeel van je signalering binnen het zorgteam moeten zijn.

D: Dat hebben wij eigenlijk ook.

**Ik zoek een beetje…**

D: Ik denk dat het voor de inspectie geen ‘hot item’ is. Daar is meer de urenroosters, of wij alle lessen geven..

**Er is ook geen percentage van het aantal lessen wat leerlingen moeten volgen? Bijvoorbeeld op professioneel gedrag.**

D: Dat is hier niet.

Z: Het MBO wel, die hebben ook een percentage.

D: 80% dacht ik.

Z: Het is iets hoger, ik geloof iets van 85%. Moet je aanwezig zijn en anders dan wordt je van de opleiding afgestuurd, zoiets. Maar in het VMBO is dat niet. In het hele VO niet. Kinderen moeten naar school tot hun 18^e^, hebben leerplicht.

**Zou dat helpen?**

D: Dan geef je ze ook een ‘vrijbrief’ om 15% niet te mogen komen.

Z: Kijk, als dat zou helpen, dan kun je dus nu bij een aantal kinderen al zeggen ‘jullie kunnen hier niet slagen’. Wat heeft het dan nog voor zin om te komen, dan is de prikkel al helemaal weg. ‘Jij kan de opleiding niet afmaken, want je bent maar 50% geweest…’

D: In het hogere onderwijs kan dat, omdat de kinderen niet meer leerplichtig zijn. Het is hier gewoon 100% verplicht om te komen.

Z: In het MBO hebben ze ook deels een leerplicht tot 18 jaar. Maar als wij zouden zeggen ‘dan kun je je opleiding niet halen’, dan kunnen we nu al kinderen naar huis sturen.

**Het heeft dus geen zin.**

Z: Alle prikkels zijn dan weg.

**Zijn er nog dingen die jullie kwijt willen?**

Z: Wat voor mij van heel groot belang is, dat het heel goed kortgesloten wordt met de leerplichtambtenaar. Jullie zijn allebei een gemeentelijke instelling en ik kan me niet voorstellen dat dat niet een veel sterker stuk moet zijn, nu de gemeente begonnen is om alle zorg te regelen (CJG), en alle gelden krijgt voor de jeugdzorg, dat daar niet meer vanuit de gemeente de regie op komt. Het strandt vaak na het stukje schoolarts vind ik, dan komen wij in het moeras. Dat stukje is nog helder en dan… En als de ouders niet komen ook, ja, wat dan. Als ze zeggen ‘ik ga er niet naartoe’. Dan kom je in het ‘drijfzand’.

D: Dus het is heel goed als jullie actie gaan ondernemen naar de leerplicht en naar de huisartsen.

Z: Voor ons is dat het grootste belang in heel dit traject.

**Interview 11. M@ZL onderzoek	Datum: 26-01-2012**

**Aanwezig:	Yvonne Vanneste (onderzoeker), Marlou van de Loo (semi-arts)**

**Directeur school, zorgcoördinator School 5 / P_5_ en S_5_**

**Variant:	first route**

**Algemene gegevens**

Het gesprek vindt plaats op school, in vergaderkamer. Marlou start met het interview. Het is lekker rustig op school, want de leraren staken vandaag. Rustig gesprek, directeur en zorgcoördinator vullen elkaar goed aan, prettige sfeer.

**Verslag interview**

**Vragen/opmerkingen door interviewster dikgedrukt**

Antwoorden/opmerkingen door geïnterviewde in normale opmaak.

Directeur = D, Zorgcoördinator = Z.

**Hoe ging het hier voor M@ZL is gestart?**

D: Nou, ik schat even in dat we toch wel een jaar of 6 geleden zijn begonnen met het ziekteverzuimproject. Voordat M@ZL kwam, deden we al mee aan het project van de GGD, waarin we in principe volgens bepaalde spelregels keken naar afwezigheid, ziektefrequentie van leerlingen. Het sterke daarvan was dat we zeiden ‘we kunnen op een neutrale, objectieve manier, of er nu wel of niet iets aan de hand is’, in ieder geval aan het voetlicht brengen van ‘daar gaan we eens over praten’. Want het is eigenlijk heel gek dat, als je 3 of 4 x per jaar afwezig bent, dat het niet opgemerkt wordt. En laten we heel eerlijk zijn, de situatie daarvoor was, dat we af en toe wel zagen dat kinderen ziek waren, maar als ze ziek waren, dan namen we dat voor lief aan. En we hebben altijd, in ieder geval gedacht dat we, op die manier, toch achter een stukje problematiek konden komen bij kinderen waar we misschien daarvoor geen zicht op hadden. En ik herinner me van die eerste jaren dat we ieder jaar 1 keer evalueerden op basis van een analyse en dat we een jaar of 8 geleden al begonnen te zien dat er, als kinderen ziek waren, er ook heel vaak sprake was van meervoudige problematiek. En vervolgens kwam de GGD met ‘we gaan M@ZL doen’ en ik begreep dat er nog steeds twee vormen zijn (smal en compleet), maar toen wij tegen M@ZL aanliepen was het voor ons.. Wij waren tevreden over het project dat we deden en zoals het bij ons gekomen is, was er ook geen alternatief.. Ja, er was een alternatief om het niet te doen. En wij vonden hetgeen dat we hadden de moeite waard en dat wilden we niet kwijt. Daar hebben we vervolgens wel financieel met de gemeente een deal kunnen maken, dat we uit een stukje onderwijskansen-beleid of hier in Oosterhout met de gemeente samen financieren, denk ik. En een stukje binnen het samenwerkingsverband.

**VSV.**

D: Ja.

**Maar de oude manier van ziekteverzuimbegeleiding is gestopt, omdat de gemeentes de stekker eruit trokken. Toen moesten de scholen het zelf gaan betalen.**

D: Ja, dat is het kip en ei verhaal een beetje. Voor mij, iets verder afstaand van de materie, is wat ik nu hoor, als ik jullie presentatie bij het directieoverleg zag, dan zit er niet zo veel verschil in wat wij met het oude project al deden.

**Kun je zeggen wat het verschil wel is?**

D: Nee.

Z: Ik denk dat een van de verschillen is, dat er een nauwere samenwerking of verbinding is tussen de jeugdarts en de leerplicht. En hun rollen binnen het ZAT daarin. Je krijgt wat.. Kijk, eerst stelde je vast dat een kind ziek was en dan werd hij opgeroepen door de jeugdarts en eventueel nog een keer. En of er dan wel of geen gehoor aan werd gegeven, dat werd dan vastgesteld en daar hield het op. Nu krijg je toch meer dat als ouders op een gegeven moment ‘geen gehoor geven aan’, dan lopen ze de kans om de leerplicht binnen te krijgen. Dat was toen niet. Dat is denk ik ook een van de sterke kanten van M@ZL. Kijk, in het financiële is er denk ik wat ons betreft, dat de gemeente zich terugtrekt in het geheel, in het subsidieverhaal, dat loopt ook al jaren zo het loopt.

D: Maar als ik nou van een afstandje kijk hè, en ik zit niet bij het ZAT, dan hoor ik maar zelden dat er in het ziekteverzuimproject zo ver gegaan wordt dat er een leerplichtambtenaar ingeschakeld wordt.

Gebeurt dat?

Z: Ja, zeker. Het is gelukkig geen.. Dat zou slecht zijn, als het een wekelijks terugkerend fenomeen zou zijn. Maar ik denk dat ik toch wel 2,3,4 voorbeelden heb van mensen die dan gewoon geen gehoor geven aan zo’n oproep en dat tot 2 x toe en dat ze dan naar de leerplicht moeten. Die zijn er wel. En we zitten zelfs met 1 ouder die principieel weigert. Daar heb ik nu weer een aanmelding van teruggetrokken, omdat ik anders die ouders zo gigantisch tegen mij in het harnas jaag. Maar waarbij we wel met de jeugdarts afgesproken hebben dat die contact opneemt met de huisarts. Daar hebben we ook toestemming voor van de ouders. Om daar toch een keer het verzuim dat die leerling vertoont in beeld te hebben. *Dus ja, de lijntjes zijn kort. En het wordt ook aan elkaar gekoppeld ja. Dat is wel een verschil met voorheen. En dat vind ik ook wel sterk. S_5d_*

**Heeft het ook nut als ze naar de leerplicht zijn geweest?**

Z: Ja, kijk (lacht).. Er zitten heel veel voordelen aan M@ZL, maar met al dit soort projecten, ja, een nadeel wil ik het niet noemen, maar wat altijd lastig is… Je bent van een heleboel schijven afhankelijk. Dat begint al bij de aanmelding. Dan zijn we afhankelijk van de mentoren hier in de school dat die hun werk goed doen. In het geval dat er verwijzingen zijn naar de lpa, ben je ook afhankelijk van de doortastendheid van een leerplichtambtenaar. En dat gaat de ene keer wat soepeler dan de andere keer. Ik verwacht op een gegeven moment dat een lpa ook, als er werkelijk iets aan de knikker is, dat die dan ook doorpakt en het niet weer teruglegt op het bordje van bijvoorbeeld de school. Maar dat komen we ook in andere trajecten tegen. Op het moment dat het echt heel erg ‘heet’ wordt, dan beginnen we toch een beetje te krijgen van ‘terugschuiven die hete aardappel’ en dat is een menselijk trekje denk ik soms wel eens. We kunnen het op papier allemaal heel netjes met elkaar afspreken en vinden dat het zo moet. Maar er is een verschil tussen dingen op papier vastleggen en het dan werkelijk in de praktijk, als het erop aan komt, ook doen. Want je krijgt ook te maken met ouders die niet altijd even meewerkend zijn. Daar moet je wel tegen kunnen. Waarom moet ik naar de jeugdarts? Ik ga toch naar de huisarts, of ik heb een traject lopen bij het ziekenhuis. Dat is nog wel lastig om daarop te reageren, maar daar kan een jeugdarts wel een meerwaarde hebben. *En een jeugdarts bespreekt dit soort zaken toch anders dan een huisarts. S_5c_* Dus ja, ik ben over het algemeen best wel tevreden over zoals het hier in het ZAT loopt en hoe de verschillende mensen hun rollen invullen. Maar als het écht heel heet wordt, wordt het heel erg moeilijk. Dat is gewoon zo.

D: Daar zou je het meeste rendement willen verwachten? Of daar zou je het een zwaarder gewicht willen laten hebben dan.

Z: Nou, ik vind dat ik gewoon een, of ik ervaar het, laat ik het zo zeggen, op allerlei terreinen als een probleem. Het is vergelijkbaar met een geval waarbij we met leerling vastlopen en we spreken hier in het samenwerkingsverband met elkaar af dat we ‘open moeten staan voor andere mogelijkheden’, dat is allemaal prachtig, maar op het moment dat het er écht op aankomt en ik met een leerling zit waarmee ik niet verder kan, dan wordt het toch in een keer een ‘hele hete aardappel’ en dan komen er andere ‘krachten’ los. En dat gebeurt hier ook. Maar je praat hier over de ‘incidenten, de excessen’. Maar gewoon het… Ik krijg bijvoorbeeld maar zelden, want dat hoorde ik in het begin nog wel eens, dat ouders naar de school gingen bellen (van een andere school gehoord) van ‘wat doen jullie nu, de GGD op mijn nek, hoe komen jullie erbij’. Ik heb dat maar zelden gehad, dat ouders op die manier reageren. Die gaan gewoon netjes naar die oproepen toe en die ervaren dat gewoon als positief. Geen probleem.

D: Dat hebben we in het begin ook wel eens gehad hè. Met name, ‘het hier op school dat gaan doen’, daarvoor is er een centrale plek geweest bij de start van het ziekteverzuimproject. Met name dat de schoolarts hier op school zat, dat was om het wat laagdrempeliger te maken. Want anders kwamen ouders in het begin toch ook wel eens, kinderen nog halen en dan moesten ze van hieruit weer naar een andere plek om die schoolarts te bezoeken.

Z: Als ik het me goed herinner, is dat ook een van de zorgen geweest toen M@ZL om de hoek kwam, omdat dit dus weer wat het bezoek aan de jeugdarts betreft, dat het weer buiten de school zou zijn. En in hoeverre dat voor mensen een belemmering zou zijn, die drempel ligt dan inderdaad wat hoger. Maar dat is eigenlijk alles meegevallen.

**En de gedachte daarachter is voor jullie wel duidelijk, waarom we dat doen?**

Z: Ja. Wat dat betreft.. Over het algemeen heb ik daar eigenlijk wel een heel goed gevoel bij en draag ik het ook wel, op een aantal deelaspecten na. Je bent toch afhankelijk van alle schijven, die moeten wel hun werk goed doen.

**En wat zijn dan de valkuilen?**

Z: Nou, ik vind vooral het knelpunt, waar we het hier vanmorgen nog over hebben gehad. Kijk, mentoren, we proberen er alles aan te doen om te zorgen dat.. Mentoren zijn hier de spil in de begeleiding, dat zijn degenen die verantwoordelijk zijn voor hun klasje om te zorgen dat dit soort zaken op tijd gemeld worden. Die worden op allerlei manieren gevoed, met uitdraaien uit Magister, dat leerlingen in aanmerking komen voor een aanmelding, het aanmelden zelf wordt voor ze gedaan. Ze hoeven alleen maar tegen een medewerker hier te zeggen van ‘joh, meld deze leerling bij M@ZL’. En dan nog zie ik, het wordt wel beter, maar er glippen er bij mij nog steeds doorheen. En dat vind ik een zorgpuntje, voor ons.

**En is het dan een kwestie van tijd, druk, of er niet mee bezig zijn?**

Z: Vaak wordt wel gebruikt van ‘we hebben het al zo druk en dit komt er allemaal maar bij’. Dat is een gevoel. Daar moet je doorheen.

D: Ja, en ik neem het ook wel voor ze op. Want ik denk dat ze het niet altijd te druk hebben, maar die mensen zijn – en dat vergeten we wel eens – niet het onderwijs ingegaan om papierwerk en dit soort dingen te doen. Die willen wel een babbeltje maken met die leerlingen of een keer opmerken dat… Maar het liefst houden ze het daarbij. En dat is af en toe wel lastig ja. Want het is niet alleen maar de aanmelding van het ziekteverzuimproject, dat is maar een klein dingetje, we hebben zo nog 10 van die dingen.

Z: Ja, maar daar start het wel. Als de aanmeldingen goed lopen, dan…

D: Ja, maar daarom is dat ook het lastige. Wij hebben in het kader van het ziekteverzuimproject en het kader van een stukje ondersteuning, een onderwijsassistent weggezet die een hoop administratief werk doet. Maar dan nog is het iets heel anders dan lesgeven en alles wat direct te maken heeft met jouw lessen.

**U zei net dat mentoren alleen hoeven te signaleren en het dan neerleggen bij de administratie. Nemen zij ook nog contact op met de leerling of de ouders?**

Z: Ja. Kijk, in die aanmeldingsformulieren om te melden, die vullen ze zelf met de pen in. En een van de dingen die ze moeten bevestigen is dat er oudercontact is geweest. En vervolgens gaan ze naar de administratief medewerker en die verwerkt verder de digitale aanmelding. Maar het oudercontact is er dan geweest ja.

**En moet de mentor dan ook nog informatie opschrijven over de leerling?**

Z: Ja, dat staat ook op dat formulier. Er staat eigenlijk gewoon hetzelfde als het digitale formulier. De mentor vult dus in dat hij een vermoeden heeft van bijvoorbeeld een griep of iets anders. Dus dat wordt ingevuld en overgenomen op het digitaal formulier. Op die manier verloopt die melding. Maar vervolgens gaat de melding naar de GGD en als de leerling dan is opgeroepen en er is een terugkoppeling die centraal binnenkomt bij de administratie en degene die dat verwerkt zorgt dat de terugkoppeling naar de mentor gaat en dat er een hier wordt opgeslagen. Dus de mentor krijgt altijd de terugkoppeling te zien met wat eventueel van hem/haar verwacht wordt. En verder gebruiken wij het in het ZAT natuurlijk.

D: Speelt de factor tijd daar nog een rol? Uit de beginfase herinner ik me dat het moment van aanmelden en terugkoppelen, dat het nog wel eens lastig was om daar de relatie nog tussen te zien. Dan zaten er een paar weken tussen met aanmelden, moesten ze nog naar de jeugdarts en dan… Speelt dat nog steeds?

Z: Dat gaat eigenlijk wel een stuk beter. Ik ben er vorig jaar nog wel een keer tegenaan gelopen, dat er een aantal echt veel later binnen kwamen.

D: We hebben / willen dat vaak in het onderwijs. Als we het moeilijk hebben met een leerling of met een groep en we willen vandaag iets doen, daar nog wel een keer over praten, maar uiterlijk volgende week rendement.

**Yvonne: daar wil ik toch wel op inhaken. Bij M@ZL moet binnen zoveel tijd kinderen opgeroepen worden, wordt ook gedaan en dan moet er op de dag zelf teruggekoppeld worden.**

Z: Dat gebeurt ook niet altijd hoor.

**Dat is iets wat de jeugdarts moet doen. Ze worden binnen 10 schooldagen opgeroepen, veel eerder heeft geen zin. Regelmatig worden afspraken verzet, daardoor is er ook een vertraging. Uitstelgedrag ook wel. Maar we hebben afgesproken dat een jeugdarts meteen terugkoppelt.**

Z: Maar er is ook een tijdje geweest dat we niet onze vaste jeugdarts onze leerlingen zag. Dat had dan ook met de organisatie bij jullie intern te maken. Dan lopen dingen ook net wat anders. Maar ik wil niet met een beschuldigende vinger wijzen, want het is voor mij een prachtig traject. Maar er zitten een aantal schijven in en als die allemaal precies op het juiste moment.. Dan loopt het perfect. Maar zo werkt het niet, zo zit het leven ook niet in elkaar. Dus ik snap het wel. Ik heb wel eens de indruk dat ik onze jeugdarts minder zie, dan dat ik die zou willen zien.

**Dingen bespreken.**

Z: Ja, ook niet in het ZAT.

**Als het niet zo goed loopt, spreek elkaar daar dan op aan. Dat mag, dat hebben we ook afgesproken.**

**En de terugkoppeling zelf? Is die nuttig?**

Z: Ja, dat is wisselend natuurlijk. Maar het is bijvoorbeeld heel erg nuttig als een jeugdarts vaststelt dat een leerling gewoon een volledig lesprogramma kan volgen en dat als het verzuim aanhoudt, er een melding bij de leerplicht geadviseerd wordt. Dan heb je dat in ieder geval in handen.

**En kunnen docenten daar iets mee?**

Z: Nou, in dit geval is het denk ik meer voor ons als ZAT en binnen die club om dit soort dingen.. Aan de andere kant kan het ook zijn dat een jeugdarts bevestigt dat een leerling terecht wat vaker naar het toilet moet, ik noem maar iets. Nou, daar kan een mentor / vakdocent iets mee. Ik vind het moeilijk om daar.. Het is afhankelijk van wat er geadviseerd wordt of wij er iets mee kunnen.

*Maar het effect zit hem misschien ook wel gewoon in het feit dat ook ouders in de gaten krijgen hoe vaak ze hun kind ziek melden S_5f_* en van ‘ik kan mijn kind..’. Er zijn ouders die laten hun kinderen thuis ‘als het bij het oorlelletje zeer doet’. En daar kunnen wij als school iets van zeggen, maar nu zegt er ook een keer een jeugdarts iets van. Ouders hebben dan in de gaten dat ze hun kind niet zomaar ongestraft thuis kunnen laten. Want daar worden ze op aangesproken, niet alleen door de school, maar ook door de GGD. En die GGD gaat ook nog zeggen van ‘als ik dit blijf doen, dan gaat de leerplicht in beeld komen’. Daar gaat een preventieve werking van uit, denk ik.

D: Met name dat hè. Dat is altijd een van de dingen geweest. We hebben ook voor nieuwe ouders in mei al een ouderavond, als leerlingen aangemeld zijn en als leerlingen 3 weken hier op school aan de bak zijn, dan hebben ze al 2 x gehoord dat wij een ziekteverzuimproject hebben. En dat ouders dus niet gek moeten staan kijken, als ze een keer gebeld worden. Dus er toch zoiets..

En dan vergelijken ouders dat wel eens met hun eigen traject. Dan zeggen ze ‘oh, het is een soort arbodienst’. Nou, prima, dat was ook een beetje de bedoeling van M@ZL dacht ik.

*En als je dat inderdaad gewoon vanaf het begin heel duidelijk communiceert met ouders en zij het dan ook zien als een extra hulp en niet als een of ander boosaardig controlemiddel, S_5a_ dan voorkom je ook - in de tijd dat het zich voordoet - allerlei boze telefoontjes van ‘wat zijn jullie nu met mijn kind aan het doen’. Want dat is dan al gecommuniceerd.* *S_5e_* Wat dat betreft hebben we daar niet zoveel last van. Een geaccepteerd fenomeen hoor.

**Dat scheelt al een heleboel denk ik.**

Z: Ja, en het is ook… Het is elkaar versterken in het begeleiden van kinderen vind ik. Gewoon in hele brede zin. Het onderwijs en de zorg, dat kan wat mij betreft niet dicht genoeg bij elkaar staan. Niemand heeft de wijsheid in pacht, samen ben je sterk. Dat geldt voor mij, maar ook voor het SMW en voor al die fenomenen. Gewoon een club en samen het kind begeleiden. En daar is de GGD, het gezondheidsaspect, een onderdeel van. En een wezenlijk onderdeel.

**Waarom is het zo belangrijk om die kinderen met ziekteverzuim te begeleiden?**

Z: Nou, kijk, in mijn ogen en dit bedoel ik niet zo negatief als dat het overkomt.. Maar onderwijsmensen zijn ‘eigenheimers’ en die denken dat ze overal verstand van hebben. En ‘wie gaat mij vertellen wat ik met die leerling moet doen’. En als er nu op het medische of sociaal-emotionele vlak vanuit een andere hoek en vanuit een ander perspectief adviezen of tips komen, dan vind ik dat heel erg waardevol. En ook voor mensen uit het onderwijs, die op dat vlak.. Ik denk dat ik er zelf ook op het vlak van het sociaal-emotioneel benaderen van kinderen een heleboel van geleerd heb. Van de mensen die hier van buiten het onderwijs de school binnengekomen zijn. Daarom zeg ‘samen ben je sterker dan alleen’. En het onderwijs kijkt met een bepaalde blik en vanuit een bepaald perspectief naar leerlingen. Maar een leerling is meer dan alleen een vat waar een hoop kennis in gepompt moet worden, daar zitten ook wat andere aspecten aan. En die mogen wij niet uit het oog verliezen.

Daarom vind ik het belangrijk om de zorg de school binnen te trekken. Dat denk ik. En ik zie daar gewoon heel duidelijk de meerwaarde van. En dat voel ik ook iedere dag.

**En ze zeggen wel eens ‘de school is geen zorginstelling’, hoe ver ga je in die zorg binnen de school trekken?**

Z: (fel) Als school, en dat is een gesprek wat ik tig keer in de maand voer denk ik, we hebben als school de belangrijkste taak om een kind een diploma te bezorgen. Linksom of rechtsom, dat is onze core business. Alleen ja, in dat traject daar naartoe, kun je allerlei problemen tegenkomen. Van alles en nog wat, in de breedste zin van het woord. Ja, dan zou het wel erg kortzichtig zijn als ik als onderwijsman zou zeggen ‘dat kan ik allemaal zelf wel oplossen’. Dat kan ik dus niet! En ik weet nu, omdat ik een aantal zaken in de rand heb zitten die ik uit de kast kan trekken als een kind in de problemen komt, dat ik die in kan zetten om in ieder geval te zorgen dat ik uiteindelijk dat kind aan een diploma help. Dus wat mij betreft houdt de zorg nooit op, totdat ik erachter kom ‘en hier.. mijn arm heeft ook een einde, mijn hulparm’. Als mijn grens bereikt is, ‘ik ben handelingsverlegen’, dan stopt het. Maar dat kan een heel eind, wat mij betreft.

D: Het is geen doel hè. Als onderwijs eigenlijk, hebben we de kant van de medaille, die gaat om prestaties. Daar worden we in principe op afgerekend. *Uiteindelijk worden beoordeeld op het aantal geslaagde leerlingen. P_5a_* En je kan alleen maar tot prestaties komen, als je op een gezonde manier tot leren kunt komen. Daar heb je een lokaal voor nodig, soms een digibord, of een krijtje. Maar je hebt ook kinderen nodig die met een bepaalde belangstelling en ook met een bepaald gestel naar school komen. Als er daar iets gaat rammelen, dan moet je iets in die voorwaardensfeer doen. Daar zit ook dat ziekteverzuimproject natuurlijk. Dat helpt ons om te zorgen dat we wel dat onderwijs kunnen laten gebeuren. En het is een eerste laagje denk ik van hulp.

Wij (D + Z) zijn beiden vanuit de zorg doorgegroeid in de school, maar wel langzamerhand ook met een andere pet op. Ik zeg ook wel eens tegen Z, er is ook een grens. En bepaal die grens. Je moet ook een dingen die je buiten school kan laten, buiten school laten. Want in toenemende mate moeten wij ook die kant van de medaille van die prestaties steeds harder maken. Daarom is het goed dat het er is en daarom is het goed dat het ook aan de rand van de school staat. Voor een stukje denk ik. Maar je kunt niet met kinderen tot leren komen als er een aantal dingen niet kloppen. Dat geldt ook als er een hoop trammelant in een gezin zit, dan moet je ook durven zeggen van ‘nu is dat leren even niet zo belangrijk’. Dan zijn er maar wat minder prestaties, alleen als dat te grote vormen aanneemt, dan houdt het wel ergens op.

Z: Maar het is ook gewoon vanuit het oogpunt van kinderen. Ik bedoel, als ze hier zitten.. Als ik geen alternatief heb of als er geen alternatief is, dan wil ik en moet ik hem nog steeds binnen deze school houden. En dan is toch het doel om hem toch wel een diploma te bezorgen. En dan is het wel zoeken naar alternatieven, maar als ze er niet zijn, moet ik er mee verder. Klaar. Ik denk dat we daar ook tegenaan lopen. Ik ben ervan overtuigd dat een aantal kinderen hier uiteindelijk hun diploma halen, dankzij een stuk zorg die we er extra in hebben kunnen stoppen. En als die er niet was geweest, dan hadden ze dat diploma waarschijnlijk niet gehaald.

D: Soms heeft die zorg te maken met gezondheid. Want dat is het hè. Er zijn een hele hoop terreinen waar het ziekteverzuimproject niet eens aan raakt, maar in een aantal gevallen wel. Volgens mij zien jullie dat in de statistieken toch ook wel heel vaak dat er gezinsproblematiek speelt. Dan heeft het niets met het fysieke te maken, maar dan is het psychosociaal of somatisch.

**Het uit zich soms wel in klachten, waardoor ze thuisblijven.**

Z: Maar wij hebben gewoon, wat dat betreft, iedere vorm van zorg nodig. Ik bedoel, er zijn hier ook kinderen die, en dat zeg ik gerust, dankzij een schoolcoachingstraject hier uiteindelijk de eindstreep halen. En dat is een andere vorm van hulp. Ik durf nog wel verder te gaan. Er zijn erbij, maar dat zijn er maar heel weinig, die het dankzij de wijkagent het hier halen. Dat die een keer eens tegen een leerling zegt van ‘joh, jij zit op het verkeerde spoor, dat moet toch echt anders’. Het komt toch anders over als een man met zo’n pet op dat een keer zegt in plaats van meneer de directeur van een school. Zo werkt dat, denk ik. En als je elkaar daar, als je dat spel samen goed speelt, dan is dat alleen maar een meerwaarde voor het kind. En daar gaat het uiteindelijk om.

**En de ouders? Waar zitten die in het verhaal?**

Z: Wij doen niets stiekem, laat ik het zo zeggen. Het is altijd in overleg met ouders. Daar heb je soms de overtuigende vorm voor nodig, soms de overlegvorm, soms de adviserende vorm. Maar je betrekt er altijd de ouders bij. Je kunt en je mag niets stiekem. Dat moet ook niet. We hebben het hier altijd over de driehoek school – ouder – leerling. En in dat plaatje past iedere vorm van hulp die we aanbieden.

**En de ouders staan in het algemeen wel samen met jullie?**

Z: Ja, over het algemeen wel. Natuurlijk. En we voeren ook wel eens wat lastigere gesprekken, waarbij het woord overtuiging om de hoek komt kijken. Vaak is het adviseren en zijn mensen blij dat er aandacht wordt besteed aan het probleem waar ze mee zitten.

D: Zijn er mensen die – want het is voor mij ook wel leerzaam – op principiële gronden zeggen of op basis van eerdere ervaringen ‘mijn kind zal en gaat niet naar de GGD, want…’. Die hebben we ook gehad bij het SMW. Mensen die zelf in die hoek werkzaam waren en zeiden, ‘ja, maar ik ga met mijn kind niet eens aan zo’n traject beginnen, want ik wil niet dat hij daar ook maar in het bestand voorkomt’, vanwege eerdere negatieve ervaringen.

Z: Ja, klopt. Die hebben wij met de GGD 1 nu. Maar dat zijn echt excessen hoor.

D: Dus dat is te verwaarlozen.

Z: Ja, dat is echt te verwaarlozen. En ook de telefoontjes die ik binnen krijg van mensen die tegen mij zeggen ‘wat maak je me nou, ik krijg een uitnodiging van de GGD en wat moet ik daar gaan doen’. Als ik die op jaarbasis 2 krijg, dan is het veel.

**Ook een beetje van ‘waar bemoei je je mee?’**

Z: Wat ik tot nu toe meegekregen heb, is er meer van ‘wat moet ik daar gaan doen, het is toch duidelijk’. Van ‘het is helemaal duidelijk, mijn kind heeft zijn knieschijf gebroken en heeft een aantal weken in het gips gezeten, mocht en kon niet naar school’. We hadden toen een huiswerkregeling en toch is hij daar op een gegeven moment terecht gekomen. Omdat hij voldeed aan criteria, maar die begon al te bellen. Van ‘waar hebben jullie het nu over?’.

**Wat heb je toen gedaan?**

Z: Toen heb ik gezegd ‘neem contact op met de GGD, leg jullie verhaal uit’. Want dan zal daar nog wel uitgelegd worden wat er misschien nog moet gebeuren. En ik heb niet meer scherp op het netvlies wat er toen is gebeurd, maar volgens mij is het met een sisser afgelopen. Dat zijn wel de lastige zaken.

D: Het klinkt ook wel als een stukje bemoeizorg. Als je kind in het hele medische traject, met school in overleg goede huiswerkregeling heeft en er fysiek echt iets aan de hand is.. En je krijgt dan nog van een derde partij te horen dat er misschien nog iets meer gebeuren.

Z: Maar je komt op het snijvlak te zitten, en dat begrijp ik ook wel, wij moeten niet op de stoel van een arts willen gaan zitten. Dat moeten we gewoon niet willen. En het gaat ook niet om het corrigeren van ouders, daar gaat het niet om**.** *Maar gewoon dat iemand met een medische achtergrond gewoon bevestigt dat datgene wat er gebeurt is, dat het goed is zo. Daar gaat het om. Maar ook als iemand met een medische achtergrond vaststelt dat daar toch wel onterecht lang thuis gehouden is. Van ‘uw kind heeft wel in het gips gezeten, maar die had met een rolstoel misschien best wel een les kunnen bezoeken, waarom heeft u hem zo lang thuis gehouden?’. Dat soort gesprekken hè. Dan gaat het niet eens om verwijten, maar wel om die mensen op dat moment op scherp te zetten. S_5b_*

D: Ja.

**Inzicht geven.**

Z: Ja.

D: Dat zijn dezelfde spanningsvelden als dat een collega’s bij de huisarts zijn geweest, dat het fysiek allemaal niet lekker gaat en ze worden opgeroepen bij de bedrijfsarts. Dan heb je ook wel eens wisselende verhalen.

Z: Hetzelfde verhaal.

**Maar wat ze nu binnen de bedrijfskunde zeggen ‘praten mag, maar werken moet’. Dat is hier hetzelfde. Je mag best wat mankeren en we gaan hulp zoeken, maar ondertussen kun je gewoon naar school.**

Z: Maar soms zijn dat soort gesprekken ook gewoon eye-openers voor ouders zelf hè. ‘Ja, natuurlijk’.

**En een behandelend arts heeft daar eigenlijk geen oog voor. Die is bezig met een diagnose, behandeling en een genezingstraject. Die is niet bezig met de participatie van het kind. Dat is denk ik de meerwaarde.**

Z: Ja, en als ik het als onderwijsman tegen zulke ouders vertel. Die zeggen tegen mij, ‘ja, heb jij er ook al verstand van dan’. Dus ik moet dat niet vertellen. Daar zit gewoon de meerwaarde van dit soort trajecten denk ik.

**Zijn er nog dingen die jullie binnen M@ZL missen of die jullie anders hadden aangepakt? Verbeterpunten?**

Z: Wat ik in het begin ook zeg, het zijn van die trajecten… Kijk, op papier is het allemaal geweldig. Alleen de praktijk is vaak wat weerbarstiger dan het papier. En je zou willen dat ieder radertje precies op het juiste moment in elkaar grijpt. Dat is een utopie, dat gaat ook nooit gebeuren. Maar ik ben blij dat wij dit zo hebben en dat we doordat we elkaar regelmatig zien, dat je ook gaandeweg met elkaar dingen kunt bijstellen. Dat je zegt ‘dit moeten we de volgende keer anders doen’. Maar er zijn geen dingen die ik echt mis. Nee, volgens mij niet.

D: Je doet ook een soort bijvangst hè. Want doordat je M@ZL hebt, zie je ook structureel en consequent je schoolarts in je overleg. En ik weet nog uit eerdere tijden dat we het niet altijd acuut over een aantal onderwerpen concreet over die leerling hadden, maar ook over een aantal zijdelingse zaken, waar je wel je voordeel mee kon doen. Het is ook je netwerk, wat je daardoor hebt.

Z: Ja, wat ik bijvoorbeeld ook doe. Wij hebben onze ZAT’s die elke 2 weken bij elkaar komen, alleen in de grote bezetting inclusief jeugdarts is dat om de 6 weken. Maar wat ik wel iedere keer doe bij ieder ZAT, is dat we een bestand rondsturen van wat er besproken gaat worden. En de jeugdarts krijgt die ook en soms krijg ik dan een reactie van ‘hee, die heb ik gezien en ik heb daar het volgende over, bespreek dat bij jullie’. Dus in die zin is die er wel bij betrokken. Dat zijn de korte lijntjes toch wel. Ja.

**Maakt het voor het project nog uit in welk leerjaar de leerlingen zitten? Meer effect of is het harder nodig in een bepaald leerjaar?**

Z: Wij hebben toen, alleen ik weet niet hoe dat nu precies zit, want ik zag er van de week weer een passeren van leerjaar 4, terwijl wij volgens mij de afspraak hebben dat we uit bezuinigingsoogpunt alleen leerjaar 1, 2 en 3 melden. Maar ik denk wel, als het gaat om het begeleiden van leerlingen en ook vooral ouders in hun opvoedende taak, ligt voor mij de essentie wel in de onderbouw ja. Leerjaar 1 en 2. Daar kun je nog sturen en kneden en opvoeden. In de bovenbouw wordt dat toch wat lastiger. Waarbij ik niet zeg – want wij lopen in de bovenbouw ook tegen leerlingen aan, waarvan wij zeggen ‘dit ziekteverzuim deugt niet’, en dan kom je ook heel vaak in onmachtige ouderposities terecht – maar het is wel goed dat er dan ook een arts vaststelt van ‘deze leerling kan het onderwijs fysiek gezien gewoon volgen hoor, beste ouders’. Dan hebben wij, in ieder geval ook naar die ouders toe, een verhaal van ‘luister eens, dat vertellen wij niet, dat vertelt een arts, dus hij kan naar school’.

D: Maar zeg jij dan dat je het project, zoals dat met de afspraken staat, kunnen doen in een onderbouw met ‘we proberen 100% van wat we zien aan uitval volgens de criteria te melden’. Maar in de bovenbouw, komen we ook situaties tegen waarvan we zeggen als ZAT of, omdat we in het kader van de leerlingenzorg tegen problemen aanlopen, die zouden wij wel eens daarín willen melden. Dat is een andere systematiek van zorgen dat ze binnen het project komen. De een komt binnen het project omdat hij bepaald afwezigheidgedrag laat zien, heel consequent, rücksichtslos, zelfs als we denken van ‘nou ja, die enkel, maar dan toch maar in het project’. Soms loop je ook tegen leerlingen aan, dat je zegt van ‘nou, die zouden wij daar wel in willen stoppen, want daar is dat bezoek, en misschien wel niet op basis van de criteria, maar daar is dat bezoek aan die schoolarts functioneel’.

Z: Ja, maar die afspraak ligt er al.

**Dag mag hè.**

D: Nee, maar omdat je het had over ‘waar moet je nu systematisch op inzetten’, wanneer heeft het nu de meest preventieve werking. En het is goed om bij ouders tussen de oren te krijgen dat het kind naar het voorgezet onderwijs en als er eens een keer wat is ‘jongen, gewoon naar school’. Want dat is ook de bijwerking.

Z: Ik denk dat, als het gaat over het meest effectieve, dan zeg ik ‘bestook dan de leerlingen en ouders in de onderbouw’, want daar zit toch de categorie die voor het eerst te maken krijgt met voortgezet onderwijs. Dat die gewoon heel snel leren hoe dat dit soort trajecten lopen en wie er zich allemaal zorgen maakt. Stel dat je de eerste 2 jaar niets doet en je zou zeggen ‘laten we er in de 3^e^ maar eens mee beginnen’. Dan zeggen die ouders ‘waar bemoeien jullie je mee, wat ben je nu aan het doen’. Terwijl, als ze vanaf het begin weten dat het er gewoon bij hoort, dan wordt het gewoon. Het is een klein beetje vergelijkbaar met ouders die de eerste 16 jaar niet naar hun kinderen omkijken en dan als ze 16 worden, zeggen van ‘ja, maar jij gaat niet uit, dat gaan we niet doen’. Dan zeggen ze ook ‘pa en ma, waar waren jullie die andere 16 jaar dan’.

Mensen moeten weten dat dit gewoon normaal is, dat er dit bij hoort.

Ik ben ook blij dat het hier in de onderbouw wat mij betreft, als het gaat om melden, wat soepeler en makkelijker loopt dan in de bovenbouw. Je krijgt in de bovenbouw toch wel, dat is ook een verschil en dat moet er ook zijn hoor, vind ik. Het verschil in begeleiden van leerlingen in de onderbouw of in de bovenbouw. Er wordt toch verwacht dat de leerlingen die hier in de bovenbouw terecht komen, die hebben nog 2 jaar te gaan en dan gaan ze dat ‘grote, koude bad van het MBO in’. En als wij blijven ‘pamperen’, dan gaat dat niet goed. Dus het pamperen moet wel wat afnemen. Dat wil niet zeggen dat we wel alert moeten zijn op onder andere leerlingen die voor dit project in aanmerking komen. Maar daar heerst een andere kijk op het begeleiden van leerlingen. Op allerlei vlakken hoor.

**Verantwoordelijkheid, ouders, leerlingen?**

Z: Dat is ook weer het samenspel. Ouders, kinderen, de docenten die daar staan.

D: Docenten in de bovenbouw vinden wat gemakkelijker dat die ouder en het kind er zelf maar voor moeten zorgen.

Z: Daar heerst nog meer de overtuiging van ‘het resultaat halen’. Hij moet over 2 jaar zijn examen halen. Dus ik moet ‘niet lullen, maar poetsen’. Er moet gewerkt worden hier.

D: In het verleden hadden we die basisgedachte, van we proberen ons met het onderwijs maximaal aan te passen aan een kind dat binnenkomt in de onderbouw. In de bovenbouw vinden we dat het kind zich maximaal moet proberen aan te passen aan wat de school allemaal te bieden heeft. Daar zit een stukje zorg bij, daar mag je op rekenen, maar daar ligt het accent wel iets anders. Die basis..

**Ter voorbereiding?**

D: Ja, ter voorbereiding.

Z: Ik denk dat dat goed is. Ik denk dat het zo ook moet zijn. Ik zou het heel erg zorgelijk vinden als ons zorgsysteem van de onderbouw tot in de finesses doorgevoerd zou worden tot leerjaar 4. Dan bereiden wij kinderen denk ik op een verkeerde manier voor op de volgende stap.

**En hoe loopt het nu in jaar 4, u zei net dat het daar helemaal niet loopt?**

Z: Nou, er is toen een afspraak gemaakt dat wij leerjaar 1, 2 en 3 zouden melden. Dat gebeurt ook over het algemeen. En in 4, maar ik zag er nu toevallig wel een terugkoppeling over een leerling ‘die er doorheen geglipt is’. Maar ik vind overigens als wij in leerjaar 4 een leerling tegenkomen waar we zorgen hebben over een aspect dat te maken heeft met de gezondheid, dan moeten we hem gewoon kunnen sturen.

**Absoluut.**

Z: Ik denk dat de grootste dingen zitten in leerjaar 2 en 3.

D: Maar het ene kind in leerjaar 4 is het andere kind niet hè. Ontwikkelingstechnisch zitten er in leerjaar 4 misschien wel een paar tussen die eigenlijk leerjaar 2 zitten en er lopen er af en toe in het 1^e^ jaar de school binnen, dat we denken die kan met zijn uitlatingen, uitspraken en manier van doen betreft zo naar jaar 3 of 4. Daar zit ook een verschil. En daar hoort weer een bepaalde problematiek bij.

Z: Het is geen eenheidsworst.

**Maar als je nu met die andere visie op ‘hoe je omgaat met leerlingen, wat je mag verwachten’, die rit gedurende deze schoolperiode. Dan proef ik eigenlijk ‘onderbouw: vaste criteria, melden’. Zou je het op de bovenbouw dan een andere invulling kunnen geven?**

D: Voor mij is het hetzelfde project, maar misschien een iets andere signalering.

**Zoals?**

D: Dan zou je moeten beredeneren vanuit de problematiek of het zinvol is om het ziekteverzuimproject in te zetten.

**En wie gaat dat doen en hoe?**

Z: Dit is heel lastig hè. Je zou kunnen zeggen van, maar dan ga je weer voor mijn gevoel in ideaalplaatjes praten, ‘laten we nou voor de onderbouw de criteria zoals we die hebben hanteren’ en ervan uit gaan dat de ouders en leerlingen uit de onderbouw daarmee leren omgaan en ook daarnaar gaan handelen. Wat ik net zeg: ‘soms moeten bij ouders ook even de ogen opengezet worden’. En dan ervan uitgaan dat als ze in de bovenbouw komen, dat ze dit wel kennen en dat ze het weten en dat je dan zegt ‘nou, in de bovenbouw laten we de criteria los’, dat vind ik al link, maar als wij vaststellen van die is toch wel opvallend afwezig of vertoont opvallend verzuimgedrag om die dan te gaan melden. Maar ik denk, eerlijk gezegd, als je dat gaat doen en je gaat aan het einde van de rit naar het resultaat kijken, dat dezelfde leerlingen bij de GGD terecht komen hoor. Maar waar het mij om gaat is, dat een van de spillen waar wij van afhankelijk zijn, is de mentor.

En de mentor in de onderbouw kijkt met andere ogen naar een leerling en dit project en allerlei vormen van begeleiding dan een mentor in de bovenbouw. Voor mij is de mentor in de onderbouw meer op zorg gericht en daarna het onderwijs, want die wil de band met zijn klas, zorgen dat iedereen lekker in zijn vel zit, kijken of er kinderen bij zijn die een training nodig hebben, al dat soort dingetjes en vervolgens gaat hij onderwijs geven. En in de bovenbouw is het meer ‘ik ga lesgeven, want ik moet mijn examen halen direct, daar rekenen jullie mij op af’. En als Pietje in de problemen komt, ga ik het wel bij de zorgcoördinator vertellen, want ik heb daar geen tijd voor. Ik chargeer, maar dat zit er een beetje. En ik vraag wel aan diezelfde mentor van de bovenbouw of ze een leerling kunnen melden bij voldoen aan de criteria van M@ZL. Maar diezelfde mentor zegt ‘ik moet mijn exameneis halen’ en daar moeten wij iedere keer tegen zeggen ‘hier is er weer een die aan de criteria voldoet’.

D: En ziet die docent dan voldoende, want we hebben het 3^e^ jaar nu gemeld, ze doen gewoon mee, zijn er dan voldoende voorbeelden waarbij zo’n docent ook ziet dat zo’n melding ook uiteindelijk bijdraagt aan die exameneisen? Dat het met zo’n kind beter gaat. Want daar ligt een stuk legitimering of het project er wel / niet moet zijn.

Z: Ik denk dat hij daar te weinig van ziet.

D: Is dat omdat het er niet is of omdat we het niet zichtbaar kunnen maken? Of omdat we het niet zichtbaar maken?

Z: Soms is het ook heel moeilijk te meten. Het zijn van die trajecten die… net als de schoolcoaching, merkt een docent daar iets van?

D: Ik denk dat de schoolcoaching voor een leerling, met 15 à 20 gesprekken, dan denk ik dat het niet goed in elkaar zit als die schoolcoach tussendoor en aan het einde geen contact heeft gehad met die mentor.

Z: Dat heeft hij altijd.

D: Dus die mentor hoort van de schoolcoach wat er gebeurd is. En dat er met het kind gesproken is en uiteindelijk ook dat het linksom of rechtsom wat opgeleverd heeft. De leerling kan een aantal dingen opgelost hebben in de privésfeer, maar uiteindelijk denk ik dat een mentor toch kan aangeven dat schoolcoaching voor dat kind belangrijk is geweest. En dat vertaalt zich misschien niet in een hoger cijfer, maar wel van ‘god, dat was nodig voor dat ventje’. Nu is het de vraag of er voldoende doorwerking is van het project ziekteverzuim, dat mentoren in de bovenbouw zeggen ‘af en toe is het toch wel nodig dat ze bij die schoolarts zijn geweest, want ik merk dat….’. En als dat er niet is, kan ik me voorstellen dat het heel moeizaam gaat.

Z: Maar dat is met heel veel trajecten in die bovenbouw, dat ze daar het belang minder van zien. Daar heerst gewoon ‘niet lullen, maar poetsen’. En dan moet je de effecten bijna op hun schoot leggen en er met de paplepel ingieten. Voor mijn gevoel kijken die met een hele andere blik naar leerlingen dan mensen in de onderbouw dat doen.

D: Die willen graag de resultaten, Z. Die moeten er toch ook in het ziekteverzuimproject zijn.

Z: Maar ik weet ook nog, toen ik lang geleden een cursus RT heb gevolgd, toen werd tijdens een van de eerste lessen gezegd: ‘Het feit dat de leerling het gevoel heeft dat er aandacht is voor zijn probleem, is al 75% van de oplossing van het probleem’. En dan heb ik nog niets gemeten. Maar de effecten zijn ook vaak heel moeilijk meetbaar, terwijl ze er wel zijn. En dat vind ik van dit soort dingen ook. Het is niet altijd in glasharde cijfers te meten. Ja, een leerling vertoont minder ziekteverzuim, dat komt voor. Dat is meetbaar. En ouders die op een andere manier het gesprek ingaan met mentoren als het gaat over ziekteverzuim, dat is meetbaar. Je moet dingen ook wel wíllen zien en je moet er ook voor open willen staan, dat het zo is. En er is nog een verschil tussen een mentor en een gewone vakdocent, daar is het al helemaal een spelletje op de zijlijn voor.

D: Dat is het natuurlijk ook. We moeten het niet belangrijker maken dan dat het is. Het is een schil om het onderwijs heen.

Z: Kijk, voor mij is het belangrijk, als ik uit mijn rol als zorgcoördinator en ik zie een leerling waarbij het onderwijsleerproces in gevaar komt, om te doen wat ik kan doen om dat weer op de rit te krijgen en dan hoop ik dat ik het effect maar zie. En als diegene die les geeft dat niet precies ziet, dat is niet zo interessant, als ik het dan maar zie.

**Maar juist in de bovenbouw, is dat onderwijstraject zo ontzettend belangrijk.**

Z: Ja, maar daarom is het ook zo dat als we in de bovenbouw tegen problematiek aanlopen, waardoor het diploma in gevaar komt, dan gaan we aan de bel trekken.

**Dan gebruik je dat als criterium?**

Z: Ja, dat hoeft niet. Zo absoluut ligt het allemaal niet. Ik heb op dit moment een kind uit een hele penibele thuissituatie, daar is op dit moment nog geen signaal dat haar onderwijsleerproces in gevaar komt, maar daar gaan we wel aan de bel trekken. Het signaal komt dan op honderdduizend andere manieren, zelfs via familieleden die op school aan de bel trekken.

**Maar het is niet een mentor?**

Z: Die weet dit niet eens. Maar nu praat ik ook over excessen. Maar die zijn er ook.

Ik heb, door deze bezetting van ons zorgteam, 100% meer zicht op problematiek die hier binnen de school rondloopt. Omdat hier ook onderling de communicatie honderdduizend maal beter verloopt dan dat hij ooit gedaan heeft. Het kwalijke gevolg voor mezelf is wel dat ik heel goed weet wat er speelt, dan denk ik ‘jezus christus’, dan ken ik alle ellende van die kinderen. En dat weten niet alle mensen.

D: Maar soms kun je dus wel het ziekteverzuimproject ook eigenlijk gebruiken als breekijzer om iets bespreekbaar te maken. Dan is er dus toch effect van. Dan is het ‘gebruiken’ gewoon.

Z: Ja, dat doen we soms ook wel eens. Ik zeg soms ook wel in het ZAT dat de jeugdarts misschien een leerling kan oproepen om erop in te gaan.

D: En ‘breng jij dat eens ter sprake’, dat is heel anders als iemand van de school dat doet. Terwijl het dan toch in hetzelfde team wordt teruggekoppeld.

Z: Of ‘wil jij eens even naar die arts bellen’, want als ik bel, krijg ik niets. En als jij belt, wordt het geregeld. Dat gebeurt ook. Dat bedoel ik ook met elkaar helpen en elkaar versterken in dit soort trajecten. Dat zijn de positieve dingen.

**Is ziekteverzuim in het directeurenoverleg een item? Hebben ze het daar wel eens over?**

D: Nee.

**Geen onderwerp van gesprek.**

D: Met PvdE, maar daar heb ik het project Octo mee gedaan en HvH af en toe, maar die kom ik dan tegen in de stuurgroep van het samenwerkingsverband en de transfercoach. Daar waar het gaat om het voorkomen van uitval van leerlingen, dan kom je soms ook dezelfde mensen tegen. Dan heb je het wel eens over zaken die in elkaars verlengde liggen.

Z: Ik denk wel dat toentertijd, PvdE in het directeurenoverleg voor het eerst begonnen is over M@ZL, want het is daar begonnen als een experiment. Daar stak hij toen een loftrompet af over M@ZL. Dus in die zin..

D: Niet in het reguliere directeurenoverleg. Als ik met een aantal directeuren in een specifieke stuurgroep zit van bepaalde projecten, dan heb je het over een specifieke doelgroep en komen dit soort dingen gelukkig wel naar boven. Want het zijn allemaal onderdeeltjes in een bepaalde keten of schakel.

**Maar niet regio-overstijgend zeg maar.**

D: Nee. Wel als ik met mijn collega’s in het overleg met de gemeente zit, in het LEA. Als er vanuit het gemeentebeleid een groot gedeelte van de centen naar basisonderwijs gaat, maar ook een stukje naar het VO, dan hebben wij het er wel over dat we bepaalde zaken samen in stand willen houden. En onder andere op zo’n stuk ziekteverzuimproject, maar veel breder op het signaleren, preventief signaleren van zaken die bij kinderen spelen, waar ziekteverzuim een onderdeel van is, hebben wij hier bijna een fulltime medewerker apart als onderwijsassistent aangesteld. Ik kan gelukkig aan de gemeente Oosterhout nog duidelijk maken, dat ik dat soort dingen kan doen, omdat ik daar voor een stukje nog gestimuleerd en gesubsidieerd wordt. En dat is wat we met de gemeente Oosterhout nog doen. Dus daar is het onderwerp van gesprek en staat het expliciet in de afspraken die we maken.

**Gemeente Oosterhout is daarin een uitzondering hè.**

D: Ja, dat komt denk ik omdat we een paar mensen in die gemeente wel kunnen voeden en durven te zeggen van ‘als jullie het laten afweten en wij geloven hierin, dan hebben we toch een soort vertrouwensbreuk’.

(samenvatting)

Soms moet je mazzel hebben met je wethouder (veel verschil, jong-oud, ervaring).

Zorg voor Bredase verhaal, waarin gemeente zich handig onder een hele hoop dingen uitdraait.

**Dit is wel belangrijk. Er zijn ook gemeenten die ziekteverzuimbegeleiding wel belangrijk vinden en zeggen tegen de GGD dat zij daar iets aan moeten doen. Dan zit je ook met een probleem. Er moet dan ook een goede samenwerking met de school zijn. Vroeger nog wel betaald. Nu ook aan de scholen om te betalen, ergens de vraag ‘waar is de gemeente dan’.**

D: Ja, en wij moeten dat als scholen denk ik wel hard maken. En dat is mijn zorg ook wel. Je moet de gemeente blijven aanspreken op hun verantwoordelijkheden en zeggen van ‘het is heel simpel, als wij met een ziekteverzuimproject of transfercoaching of goed mentoraat of het terugdringen van spijbelaars, als wij daar door jullie niet in ondersteund worden….’. Ik moet het anders zeggen.

De gemeente heeft een bepaalde ambitie en bij die ambitie hoort een stukje ondersteuning. Als de gemeente die ondersteuning weglaat, moeten wij onze ambitie bijstellen. Beste gemeente, dan is het een kwestie van ‘nu wat besparen en dat straks 10 keer uitgeven’. Aan kinderen die toch onderweg verloren zijn. Kinderen die voor overlast zorgen buiten het onderwijs, kinderen en gezinnen die met een stuk bemoeizorg op latere leeftijd worden geconfronteerd, aan justitiële instellingen. Ik denk dat alles wat je preventief kunt doen, vele malen goedkoper is dan wat je later moet repareren.

En dat moet je voortdurend bij gemeenten onder de aandacht brengen. En ik vind het een hele slechte zaak, ik snap gemeenten ook wel, maar ik hoor vanuit mijn rol in de stuurgroep van het samenwerkingsverband, dat de gemeente Breda, dat heeft wel mijn zorg, ook voor het Oosterhoutse. Zo’n grote gemeente gaat wel een trend zetten. En ik hoor van de lpa’s dat er al regionaal gewerkt gaat worden straks, daar kan dat denk ik, want daar hebben we zelfs gepleit voor een goede ambtelijke instructie aan de lpa’s. Maar als gemeentes een terugtrekkende beweging gaan maken en daarmee een stuk van hun boekhouding kunnen repareren en zeggen ‘de grote broer doet het ook’. Dan zullen wij nog harder moeten roepen dat de gemeente Oosterhout dan blijkbaar ‘meer grote-steden-problematiek’ wil hebben.

Ik kan maar zo ver gaan en als langs alle kanten de geldstromen minder worden, ja, dan maak ik of mijn mensen gek of ik moet mijn ambitie bijstellen. En laten we daar met elkaar vooral duidelijk over zijn naar elkaar, wat we kunnen. Wij hebben hier in Oosterhout denk ik een traditie dat we met een aantal projecten ooit zijn gestart (de veilige school, de coaching), kleinschalig opgezet en vervolgens gebracht naar het samenwerkingsverband en daarna is het breder getrokken. Die cultuur hebben we hier wel.

**Je zegt eigenlijk ‘het hoort bij het ambitieniveau van de gemeente’. Zie je daar ook nog een stukje verantwoordelijkheid? Rollen die opgepakt moeten worden?**

D: Ik denk dat je als onderwijs altijd de gemeente aan moet blijven spreken op de verantwoordelijkheid die je samen hebt. Dan moet je het lef wel hebben om te zeggen ‘wij kunnen daar ook een stukje in’, maar een hele hoop dingen zul je samen moeten doen. En als de gemeente zegt ‘wij willen een hele hoop dingen niet’ en zich terugtrekt. Ik heb best begrip voor het feit dat er wat minder geld is, maar dan moet je daar met elkaar over praten. Je moet niet aan de kant gaan staan en zeggen ‘de ander moet het maar doen’.

Z: Het zijn ook politieke keuzes he.

D: Ja en nee. Ik denk dat er op dit moment gewoon minder geld uit te geven is in Nederland.

Z: Maar dan nog, is het een overweging: waar geef je het aan uit.

D: Maar de basis is dat je met elkaar, en het onderwijs staat niet los van de maatschappij, dat je samen dingen aanpakt en zoveel mogelijk probeert vroegtijdig dingen op te vangen. Want dat onderschrijf ik ook wel. Je moet met je inzet van zorg, om linksom of rechtsom de zwaardere zorg te voorkomen, moet je maximaal investeren in een lichtere vorm van zorg. En het ziekteverzuimproject is een hele lichte vorm van een stukje zorg.

Interview 12. **School 3 / P3 en S3	Datum: 2-2-2012**

**Aanwezig: Zorgcoördinator, lid raad van bestuur, Interviewer Yvonne**

**Algemeen: aangename sfeer, ontspannen. Beiden stemmen vaak met elkaar in met ‘ja’.**

Ziekteverzuimbegeleiding. Waarom zouden we aandacht moeten besteden aan het ziekteverzuim van leerlingen?

**R: Wat voor mij in ieder geval het belangrijkste is, is de stelling van Mischa de Winter over de rol van ouders in het onderwijs, dus je moet aandacht besteden aan ziekteverzuim van leerlingen an sich om het ziekteverzuim, maar met name omdat je samen met ouders, ouders, school en leerling, moet zorgen voor een veilige en goed mogelijke schoolloopbaan voor leerlingen,** *daar is het verzuim, en de situatie thuis en op school onderdeel van, dus door het ziekteverzuim goed te monitoren, goed terug te koppelen, ook naar ouders, heb je volgens mij een beter beeld in het welbevinden van het kind, zowel op school als thuis en vervolgens komt dat leerprestaties in ieder geval en schoolontwikkeling ten goede.* *P_3b_***. Dus dat is voor mij het belangrijkste.**

De ouderbetrokkenheid…

**R:** *Ja, en de verantwoordelijkheid, wat je dus ziet in onze maatschappij is, dat ouders in toenemende mate de verantwoordelijkheid voor de ontwikkeling van hun kind naar school schuiven, en dat is een compleet foute ontwikkeling. We hebben in onze ambitie ook staan dat we steeds meer, we willen ouders als steun zijn en bij de school betrekken. Dus ouders zijn onlosmakelijk onderdeel, logisch natuurlijk, van de opvoeding van hun kind, en de school kan daarbij ondersteunen, maar de school heeft andere taken. P_3a_***. Dus waarom is een kind ziek? Komt dat door school? Komt dat door thuis? Komt dat door een combinatie? Nou, M@ZL, door dat goed te monitoren en daarop in te zoomen denk ik dat je een beter beeld krijgt waarom een kind ziek is. We hebben het dan niet over een griep of verkoudheid, we hebben het dan over wat frequenter verzuim om een of andere reden.**

**Z: Dat is ook de reden waarom we uiteindelijk gekomen zijn bij 4 keren ziekmelding per 3 maanden en daar voldoen er nog al wat aan. Want over het algemeen, die leerlingen die langdurig verzuimen, dat zijn de leerlingen die in een ziekenhuis gelegen hebben of wat dan ook. Daar is vaak gewoon fysiek iets mee aan de hand. Het gaat over die anderen.**

**R: Ja, en ook daar kan fysiek iets mee aan de hand zijn…**

**Z: Ja, zeker.**

**R: bv migraine, maar ook dan is het de moeite waard om eens te kijken hoe komt dat nou. Het gaat niet zo zeer om registreren om het registreren, het gaat erom wat je er mee doet. Voor mij is de belangrijkste rol: in contact met die ouder en samen kijken naar hoe komt dit nu. Heeft het schoolse oorzaken, heeft het oorzaken thuis of zit het hem in de combinatie. Dat is voor mij de grote meerwaarde. Dat is een bovenliggend doel.**

U zie net veranderingen in de maatschappij, er verandert veel. Is dat zo, is de laatste tijd, jaren veel veranderd?

**Z: ja, helemaal duidelijk. Ja, bv ouders die je opbellen met straf jij mijn kind maar, want er is dit of dat gebeurd. En dan zeg ik, sorry, u bent ouder, wij niet!**

**R: Ja, en de tendens is ook anders. Laten we 40 jaar terug gaan. Als ik 40 jaar terug zeg als kind dat ik straf had gekregen van school dan zeiden mijn ouders ‘dan zul je het wel verdiend hebben’. Twintig jaar terug zette de trend zich een beetje in dat ouders dat niet zomaar meer zeiden. Die ouders kwamen dan naar school. Nu komen ouders niet meer zelf naar school, nee, die sturen de advocaat als het ware: ‘beste school, mijn kind is blijven zitten, wat doet u er aan om hem over te laten gaan? Of door te laten stromen? Want dit kost de maatschappij geld. En in het ergste geval, de politie komt op school, we hebben het al meegemaakt, om een docent te arresteren, die niet meer als zijn taak uitvoerde, wat onhandig misschien maar toch… Dus de tendens is heel erg veranderd. Ouders zijn niet meer als vanzelfsprekend die steunzender die je nodig hebt. Je moet steun hebben van ouders. Dat wil niet zeggen dat je rare dingen kan moet doen als school, natuurlijk moeten ouders voor hun kind opkomen, maar wel vanuit de gedachte we moeten het samen doen, het gaat om het welbevinden van het kind.**

Dus de meerwaarde van aandacht voor het ziekteverzuim van de leerling is dat je een manier vindt om samen met ouders te kijken naar het kind, of hoe moet ik dat zien…?

**R: Ook, maar ook om meer grip te krijgen op het welbevinden van het kind. Want ziek is niet de oorzaak. Een griepje, daar kun je niets aan doen, maar een kind dat inderdaad frequent verzuimt, ja..**

**Z: Daar is meer aan de hand.**

**R: Ja, daar is meer aan de hand. Het andere is natuurlijk logisch, het welbevinden van het kind staat natuurlijk altijd centraal, dat lijkt me evident, maar het andere is ook om die ouder daar meer bij betrokken te krijgen, met name als het gaat om problemen van kinderen.**

Want met ziekteverzuim krijg je probleemkinderen in beeld?

**R: Dat weet ik niet.**

**Z: Soms wel, soms niet. Ziekteverzuim geeft in ieder geval wel problemen. Kijk, want als ze op school zitten, dan is er structuur, dan is er regelmaat, en dan bestaat de kans dat ze op een bepaald moment enigszins op een goede manier in de ‘ongeregelde maatschappij’ terechtkomen. Die kans is dan groter. Daarom moeten we uitval bestrijden. School is gewoon een heel groot onderdeel van de dagelijkse regelmaat die kinderen gewoon moeten krijgen.**

En dus, wat geef je ze daarmee?

**Als ik ze regelmaat geef of aanbied, geef ik ze structuur, zodat ze straks hun leven misschien ook een beetje kunnen structureren. En als ze zomaar, ik bedoel, met name ons soort leerlingen, heeft toch wel erg veel last van structuurloosheid.**

‘Ons soort’ is VMBO-leerlingen?

**Ja, VMBO-leerlingen. En wij hebben dan ook nog relatief veel leerlingen met allerlei afkortingen om het zo maar eens te noemen. Dat is bij ons vele malen hoger dan op havo/vwo scholen. Wij hebben ADHD-ers, PDD-NOS, ADD-ers, noem ze maar op. Alle afkortingen hebben we in huis. Dus die zijn sowieso al vatbaarder voor structuurloosheid. Dus die moet je echt iets aanbieden. We hebben daar hier op school ook nog best veel moeite mee. Alle scholen hebben daar moeite mee. Omdat je natuurlijk steeds andere lessen, steeds andere docenten hebt. Dus je moet al proberen de schooldag als stuk van dat ritme te zien. Van ‘nou, oké, ik ga elke dag daar school, daar ben je. Dat is belangrijk voor je’. Ja, dat is regelmaat ja.**

**R: Ik denk dat hier kinderen op school het uitstekend doen en toch frequent ziek zijn, dat kan. Dat zou interessant zijn, hoe komt dat nou?**

**Z: Dat wordt meestal wel,** *met de terugkoppeling die we krijgen van de schoolarts, dan krijg je wat handvatten S_3a_***, bv van een leerling met buikpijn: als ze buikpijn heeft mag ze de eerste 2 uren thuis blijven maar vanaf het 3^de^ uur is ze dan op school. En dan is het niet meer de gewoonte, zoals het was, de hele dag niet naar school. En dat zijn afspraken die je in overleg met ouders en leerling als schoolarts maakt.**

Want het in gesprek gaan met ouders en leerlingen over het ziekteverzuim, het verschil tussen M@ZL-compleet en M@ZL-smal, is dat bij M@ZL-smal de school eerst zelf in gesprek gaat met ouders en leerling en door kan zetten naar de jeugdarts. Waar gaat jullie voorkeur naar uit?

**R: Dat is moeilijk. Dat eerst de school in gesprek gaat, dat is eigenlijk logisch.**

**Z: Ja, dat gebeurt nu ook al. Die filter wordt nu ook gemaakt.**

**R: Ja, laat ik een open deur pakken. Een kind is altijd ziek als er een belangrijke toets is, dan is het natuurlijk mogelijk dat het faalangstig is, dat zijn allemaal open deuren natuurlijk. Dus het is logisch dat je die als school er eerst uitfiltert. En niet alles zomaar naar de jeugdarts stuurt.**

**En dat kan de school ook?**

**Z: Ja, op een gegeven moment worden er ook keuzes gemaakt. Naar aanleiding van het programma dat we nu hebben en wat een stuk gemakkelijker is rollen er een aantal leerlingen uit en dan gaan de teamleiders in gesprek met de mentoren, is hier al wat meer van bekend, wat gaan we hiermee doen? Gaan we hier een aanmelding van maken of niet? Heb je al een gesprek gehad met die ouders? Ja. Oké wat is daar uit gekomen? Zijn er afspraken gemaakt? Er zijn bv wel afspraken gemaakt die niet worden nagekomen, dan heb je een stap van we spreken onze zorgen uit, want dat is wel waar de mentoren heel goed van doordrongen zijn. Aan de hand van die vragen met antwoorden van de GGD, dat je zegt ‘we hebben ons hierbij aangesloten en we hebben nu zorgen om uw kind’ en dat wordt steeds beter verwoord. Dat is een leerproces. Er zijn steeds minder ouders die zeggen ‘en ik ga niet’ en ook voor de mentoren: niet op ingaan, niet op reageren, gewoon zeggen ‘dat is uw keuze maar aan die keuze zitten wel consequenties’. Meer hoef je niet te zeggen. Geen welles-nietes van maken want dat helpt toch niet.**

En die gesprekken met de ouders vanuit school, gaan die over de telefoon?

**Z: Meestal wel, of over de mail. Er gaat heel veel over de mail, van en naar de mentor, ook tussen ouders en mentor.**

**R: Dat is ook inherent aan het onderwijs, we gaan ook mailen of over de telefoon omdat we simpelweg de tijd niet meer hebben, maar eigenlijk moet je dit in een gesprek doen. Je moet met mensen rond de tafel gaan zitten, maar er is meer te doen dan alleen maar dit. Dat is de ellende, en het ligt allemaal op het bordje van de mentor, de teamleider. En het is op jaarbasis toch een bepaalde groep leerlingen die extra aandacht behoeft. En het is eigenlijk niet acceptabel dat de ene mentor wel in gesprek gaat bij ziekteverzuim en de ander niet.**

**Z: Er zijn er al behoorlijk wat aangemeld deze periode.**

Dat is wat wij ook zeggen, dat gesprek moet face-to-face plaatsvinden. Daarom houden wij er ons op de GGD ook strikt aan houden dat ze wel mogen bellen om te verzetten maar niet om met de arts te bellen. Dan wordt het verhaal over de telefoon gedaan en loop je de kans om ze te spreken mis. We betrekken wel altijd e ouders er bij.

**R: Want naast de harde cijfers is ook altijd je gevoel. In het gesprek zie je meer. Bij het personeel heb je eigenlijk hetzelfde, dat je voelt hier is iets aan de hand, en dat gevoel krijg je niet per mail en niet per telefoon.** *Dus ik denk dat het met ouders praten over ziekteverzuim, dat valt te leren. S_3b_*

Het begint al met het een paar keer verzetten van de afspraak: uitstelgedrag?

Het structureel aandacht besteden aan ziekteverzuim vraagt veel inspanning. Op welk rendement wordt dit project afgerekend wat jullie betreft?

**Z: Op dit moment kunnen we alleen de aantallen van vorig jaar vergelijken met de aantallen van nu, dat is op dit moment ons enige referentiekader, meer hebben we niet want in het verleden hebben we die cijfers niet zo goed vastgelegd. Het is wel gaan leven binnen de school. We zijn eigenlijk al zo’n vier jaar geleden begonnen met op te merken dat er in bepaalde klassen zoveel leerlingen regelmatig afwezig waren. En dan gingen de mentoren wel een gesprek aan, soms met teamleider, maar daar kwam meestal niets uit. En dat heb je nu wel. Nu zijn de ouders ook een andere gesprekspartner omdat ze met iemand anders ook nog gesproken hebben. Dat maakt het verschil. Maar voor de rest, die cijfers van 2-3 jaar geleden zijn gewoon niet op te hoesten.**

**R: De vraag is of je er op ambitieniveau cijfers aan moet hangen. Als ik kijk naar onze nieuwe ambities dan zijn er twee die dit heel sterk raken. We willen toonaangevend zijn in de regio wat betreft pedagogisch klimaat, qua veiligheid, sfeer, aandacht. Nou, daar is dit een stukje van. En we willen die ouders meer bij het onderwijs betrekken. Daar is dit ook een stuk van. Als dit daartoe bijdraagt, en ja, is dat nou te meten. Als meetinstrument moet je de tevredenheid van ouders en leerlingen over een veilig schoolklimaat meten.** *De invloed van M@ZL is daar niet direct aan afleidbaar, maar het draagt er wel toe bij. P_3d_* **Het is niet meer dan een signaleringssysteem. Het echte werk begint na M@ZL, zal ik maar zeggen.**

Ja, in het vervolgtraject…

Maar zien ouders dat ook, dat aandacht voor het ziekteverzuim van hun kind ertoe bijdraagt dat…?

**R: Ik doe de gesprekken niet. Ouders vinden het denk ik alleen maar lastig.**

**Z: Ja, die denken moet ik weer naar school komen, nu hebben we die afspraak gemaakt en moet ik me er ook aan houden.**

**R: Mijn kind is nu eenmaal vaak ziek, heeft nu eenmaal migraine. Zo voelen de ouders dat.**

Ja, en de leerlingen ook? Die voelen zich gecontroleerd?

**R: Maar ik vind het niet verkeerd. We hebben hier ook voor medewerkers een systeem, dat als ze zich frequenter ziek melden dat ze dan naar de bedrijfsarts gaan. Hoe komt het dat je maandag na de hockeywedstrijd zo vaak verstek laat gaan- wat overigens heel invoelbaar is-. Dus ook voor leerlingen mag dat.**

Dus eigenlijk hebben jullie compleet en doen jullie smal?

**Z: Ja, eigenlijk doen we al smal. Omdat we er vanuit gaan dat de mentor op de hoogte is, dat die wel altijd contact heeft met de leerling, dat hebben we er zelf wel ingebracht, een gesprekje, ter voorbereiding op een aanmelding die gaat komen bij een volgende ziekmelding, mag ik van jou weten wat er iedere keer gebeurt als jij je ziek meldt? En sommigen mentoren zeggen, ik praat helemaal niet over M@ZL, ik ga gewoon, iedere keer als de leerling weer terug is op school, een gesprek aan.**

Verschilt dat nu per unit of locatie?

**Z: Dat weet ik niet, dat durf ik niet te zeggen.**

**R: Wat ik weet dat het op havo-vwo wat minder liep, dat het mogelijk ook meer aan de orde is op het vmbo dan op havo-vwo? Interessant om te onderzoeken.**

Vinden jullie dat M@ZL alleen maar belangrijk is voor vmbo-leerlingen?

**Z: nee, zeker niet.**

**R: Wat mij het meest puzzelt, moet je M@ZL blijven doen? M@ZL moet eigenlijk, net als andere kwaliteit, in de genen van de docent zitten. Er moet een antenne gaan, dit kind is toch regelmatig ziek. Het helpt wel om het een naam te geven, dat zet het bij mensen wel tussen de oren. Net als bij de kwaliteitsgraag ‘toetsing’. Gaan ze doordat je meet hoe ze omgaan met toetsen beter toetsen? Nee, maar het feit dat men weet dat er aandacht aan besteed wordt betekent dat het beter tussen de oren van mensen zit.**

Dus het ondersteunt binnen de school het implementeren van aandacht voor ziekteverzuim?

**R: Ja, om je dat beter bewust te zijn. En het zit nog niet op alle drie de units evenveel tussen de oren -ze kijken samen naar de lijsten met leerlingen die tot nu toe zijn aangemeld- er is nu nog maar 1 havo-leerling aangemeld. Dat geeft al aan…Het systeem van Frencken ondersteunt enorm.**

Inderdaad, havo-vwo wordt veel minder gemeld, dan is de vraag komt het omdat het minder tussen de oren zit of is er ook minder ziekteverzuim? We gaan dat wel allemaal meten, ook of er verschil tussen onderbouw en bovenbouw is. Hoe kijken jullie daar tegenaan? Er zijn scholen die er voor kiezen alleen M@ZL te doen in de onderbouw, in de bovenbouw heb je een andere relatie misschien met de leerlingen en ouders? Een andere verantwoordelijkheidsverdeling, en dit soort zaken.

**R: Ik weet het niet, volgens mij zijn er zoveel factoren die een rol spelen. Wat opvalt is dat naar mate een leerling langer op school zit, dat de score op tevredenheid over het algemeen wat afneemt. De leerling die weggaat scoort laag, maar vraag je ze een jaar later nog eens dan zeggen ze ‘het was zo verkeerd eigenlijk nog niet’. Misschien matcht dat hiermee wel. iemand die zegt ik heb het hier wel gehad op school die heeft misschien de neiging zich wat gemakkelijker ziek te melden.**

**Z: Daarom ben ik ook niet voor een verdeling OB en BB. Als ik het nu zie is de OB minder vertegenwoordigd dan de BB.**

**R: Jongens, meisjes?**

Er valt nog veel te meten. Maar goed, als je motivatie zo algemeen is als gezegd dan moet het eigenlijk niet uitmaken en dan telt het eigenlijk voor iedereen.

**R en Z: Ja.**

Krijg je de ouders steeds meer aan je kant?

**Z: Ja, laat ik zo zeggen, ik hoor minder reacties van de mentoren dan vorig jaar, dus dan ga ik er vanuit dat het over het algemeen gemakkelijker gaat. Nu nemen de ouders ook geen contact meer op met mij. Vorig jaar heb ik wel 7 of 8 ouders aan de lijn gehad van ‘nee, ik ga niet want, daar hebben jullie niets mee te maken’, noem maar op. Oké, dan wordt het gemeld als ongeoorloofd verzuim aan de leerplichtambtenaar. En dan zijn er een paar die eieren voor hun geld kiezen en een paar niet en als je dan de vervolgstap maar zet dan is dat helder.**

Ja, want als je dat niet doet, dat gaat wel heel snel zingen… We krijgen op de bijeenkomsten wel terug, dat instromen dat lukt wel aardig, dat is aardig op de rol, maar het vervolgtraject, dat blijft wat moeilijker. Dat is ook eigenlijk iets wat je samen met de GGD moet doen.

**Z: Ja, samen met de GGD en LPZ.**

**R: Wat lopen ze dan voor vervolgtraject?**

Als ze zijn geweest en de jeugdartsstelt het is eigenlijk een gezonde puber die niet meer dan gemiddeld hoeft te verzuimen, dan adviseer je bij de volgende ziekmelding sneller door te pakken en bv meteen door te zetten naar de lpa, je draait het om. Die adviezen moet je wel als school monitoren.

**R: Maar voor mij zou dat dus, het doel van M@ZL is dus eigenlijk het verzuim van leerlingen terugdringen, Dat is dan het gevolg van andere dingen inderdaad.**

**Z: Ja, we hebben het gezien.**

**R: Maar zou dat een doel op zich zijn?**

**Z: Nee, voor mij niet.**

De gemeente wil daar wel effect op zien. Daar worden we straks denk ik wel op afgerekend.

**R: dan is de gemeente gelukkig.**

Wat wil je voor effect meten? De mensen op school zeggen ‘het welbevinden van kinderen verhogen’. Maar telt dat ook voor de gemeenten? Voor andere partijen?

**Z: Nee, ik denk het niet. Ik denk dat de gemeente een ander belang heeft. Natuurlijk, wat je net zegt, het ziekteverzuim zo laag mogelijk houden. Dat ze zich straks in het bedrijfsleven niet ook zo snel ziek melden, of dat soort zaken.**

**R: Dan speelt ook op kortere termijn het hele vsv-verhaal. Een leerling die je blijft monitoren en waarvan het welbevinden kan toenemen, dan is de kans lager dat die uitvalt. Het vsv-percentage is wel iets gedaald maar niet zo veel. Ik heb de laatste cijfers net binnen.** *Misschien draagt M@ZL wel in een heel sterke mate bij tot het verlagen van het vsv, maar dat weet je pas over een aantal jaren.P_3c_* **En dat zou voor de gemeenten geweldig zijn. Dat zijn de mensen die later ook uitvallen. En dan gaat het geld kosten, noem maar op. We hebben het dan nog niet eens over het psychisch welbevinden van de mensen.**

**Z: Nee, voor de gemeente gaat het alleen maar over geld.**

Dat klopt, de gemeente wil vaak iets heel anders zien. Die hebben een heel andere focus.

Wat zijn de valkuilen als je M@ZL binnen je school wil implementeren?

**Z: Mentoren niet goed voorbereiden op de gesprekken, dat is echt een hele grote valkuil en het niet tijdig informeren van de ouders. Vorig jaar is het op verschillende units fout gelopen omdat een deel van de teams het hadden gemeld op de algemene voorlichtingsavond voor ouder en een deel niet. En dan krijg je hele grote strubbelingen.**

**R: Ja, je moet het goed wegzetten, met een zichtbare symbolische aftrap herkenbaar maken. We gaan hier proberen een omslag te bewerkstelligen en dat doen we om die en die reden. Je moet ook het doel aangeven. M@ZL zegt niemand iets.**

En wat is dan het doel?

**R: Het doel van school kun je aan je ambities koppelen. Maar wat is het doel voor ouders? Nou, het gaat over het welbevinden van uw kind, en dat niet op de langere termijn zetten maar in het hier en nu plaatsen. En als we er gezamenlijk iets aan kunnen doen dan moeten we dat doen, daar moeten we samen in optrekken. Voor de mentoren binnen je school betekent dit wat.**

Want het is aan mentoren om dit te doen?

**R en Z: Ja, dat hoort bij de mentoren.**

De directeur moet ja zeggen, de zorgcoördinator, de mentoren.

**R: Ja, en die eerste twee zeggen het snelst ja. Vooral als het weinig kost, het lijkt ons wel interessant, kijken wat er uit komt, maar die hebben het werk niet. Dus het gaat ook over het managen van verwachtingen. Dus je moet voor je mentoren duidelijk krijgen en maken ‘wat betekent het als we gaan deelnemen?’**

En wat krijgen de mentoren er voor terug?

**Z: Handvatten, dat wel. Nu weet je welke afspraken je kunt maken en wat je kunt verwachten van de leerling. Anders was het zo ‘hé, je bent niet op komen dagen, dus je hebt geen recht meer op die herkansing’. En nu wordt daar geen woord meer over vuil gemaakt. Dit is de afspraak en dat is helder.**

**R: Ja, kijk, op termijn zou het ook tijdswinst op kunnen leveren. Je investeert nu aan de voorkant in plaats van het plakken aan de achterkant. Straks gaat die leerling zich minder frequent ziek melden, die hoeft niet meer in te halen, al die docenten hoeven minder aandacht te besteden aan die leerling, dan verdient het zich terug. Maar goed, daar is een mentor nu niet mee geholpen.**

Nee, want die ziet het werk en die vindt het ook moeilijk denk ik, dat in gesprek gaan? Zie je dat ook?

**Z: Ja, die drempel is nu lager geworden.**

Hoe hebben jullie dat aangepakt? Wat kan een andere school daarvan leren?

**Z: Dat je aan de hand van jullie vragen-antwoordenlijst, in gesprek met de mentoren zelf, vraagt ‘Heb je hier voldoende aan?’ Als een ouder zegt ‘ik ga er echt niet naar toe’ wat zeg je dan? Hoe reageer je daar op? Zo’n lijst helpt.**

En een stukje professionalisering dan ook?

**Z: Ja, dat is het ook.**

**Want dit soort gesprekken kun je op andere momenten ook voeren, het is gesprekstechniek wat je aanleert.**

**R: Ja, dat klopt. De do’s en don’t’s.**

**Z: Alleen maar zorgen uitspreken, voor de rest niet. En ook duidelijk zeggen, dan kunnen we onderling afspraken maken, met u en met de leerling. Dat is alleen maar positief, ook voor u maar ook voor de leerling en voor ons.**

**R: Het belangrijkste zijn die tips. Een mentor is geen arts maar ook geen rechter. Op het moment dat je niet hoeft te bepalen of iemand echt ziek is en je hoeft er ook geen oordeel over te geven, je hoeft alleen maar te constateren.**

En wanneer zet je dan door naar de jeugdarts?

**Z: We zetten nu door als aan de criteria voldaan wordt, of, maar dat is tot nu toe maar een keer gebeurd. Nu wordt nog geen 1% niet aangemeld bij de jeugdarts. Maar bv een leerling heeft een longembolie gehad, die is tot 2 keren toe in het ziekenhuis beland, toen hebben we besloten deze niet aan te melden bij de jeugdarts. En dat doen we wel in onderling overleg.**

Ook in overleg met de ouders?

**Z: Ja, er wordt wel gecommuniceerd met ouders. Dat ze weten dat we hem hadden moeten aanmelden en dat we dat niet hebben gedaan om die reden. Stel dat hij nu regelmatig ziek gaat worden, dan wordt hij als nog aangemeld.**

Dus waarom meld je dan niet aan?

**Z: Omdat wij op dat moment denken deze leerling loopt in het ziekenhuis, is onder controle, en dat is bij ons allemaal bekend. Wat is dan de meerwaarde van de schoolarts?**

Ja, wat is de meerwaarde van de jeugdarts?

**Z: Wij zien die dan niet.**

Omdat?

**Z: Omdat hij al bij zoveel specialisten terecht kan en omdat wij dan van de ouders ook al handvatten krijgen.**

Dus je krijgt van ouders een handvat?

**Z: Ja, natuurlijk.**

Stel dat je dat niet kreeg?

**Z: Ja, dat weet ik niet.**

**R: dan veralgemeniseer je, maar ik kan me dus voorstellen dat, op het moment dat je weet waarom een kind verzuimt, dat dan de noodzaak om door te sturen er niet of nauwelijks meer is. Wanneer je weet dat een leerling altijd ziek is voor de toetsen en je weet dat het faalangst is, dan hoeft hij echt niet naar de jeugdarts.**

Ik heb een voorbeeld van een leerling die jarenlang heel intensief verzuimd heeft vanwege een bepaalde ziekte. Ouders vertellen dat aan school. Uiteindelijk is dat kind in het kader van M@ZL dan toch bij de jeugdarts terecht gekomen en daar blijkt dat hij die ziekte helemaal niet heeft. Er lijkt heel andere problematiek.

**R: over hoeveel procent van de leerlingen hebben we het hier nu? Denk je werkelijk dat wij daar aandacht aan gaan schenken?**

Ik probeer er achter te komen, er is denk ik meer waarom je niet doorzet. Het is niet alleen omdat je van ouders hoort wat er aan de hand is, maar ook omdat je een goed contact hebt met ouders, toch?

**Z: Nee, wat je nu zegt is pertinent niet zo.**

**R: wanneer je inderdaad twijfels hebt, natuurlijk zullen er mensen zijn die dingen onterecht gebruiken. We zitten hier in een omgeving dat we gemakkelijk praten hebben. Maar bv we zitten in de stad, of kinderen zonder vaste woon- of verblijfplaats, schipperskinderen bv., dan kan dat voorkomen en moet je daarnaar handelen, maar voor deze school is dat niet het publiek waar wij ons op richten. Ik hou er niet van iets te regelen op basis van uitzonderingen.**

Daar ben ik het helemaal mee eens, de uitzondering bevestigt de regel.

**R: Dus je moet doorsturen als je zegt, hé, dit is zo’n grijs gebied, dit is een beetje vreemd. Als je het zelf weet hoef je niet door te sturen. Of we weten het wel maar misschien heeft de arts nog een aanvullend advies voor die ouders, dat kan. Dan geef je de diagnose als het ware al mee. En dan vraag je, kijk er nog eens overheen, is dit het juiste advies? Dan is het consulteren voor verder advies en in het andere geval zeg je ‘we weten het niet, kijk er eens naar’.**

**Helder.**

**Het implementeren vraagt heel veel tijd en energie…**

**Z: Ja, dit moet je echt goed voorbereiden.**

Er zijn meerdere ontwikkelingen in onderwijsveld, denk bv. aan passend onderwijs. Past dit daar ook in?

**E: Ja, dat is natuurlijk een open deur. Dan wordt dit in toenemende mate belangrijk. We krijgen dan natuurlijk meer leerlingen hier met een indicatiestelling, die meer zorg behoeven en dat de kans ook aanwezig is dat het eerder fout of niet loopt zoals we allemaal wensen, en dat kan weer leiden tot uitval, enz.**

Het is een open deur, en in eerdere interviews ter sprake gekomen dus ik wilde even toetsten of dat inderdaad ook door jullie zo gezien wordt. Het feit of je een interventie accepteert moet natuurlijk passen in de hele ontwikkeling. Zijn er nog meer ontwikkelingen gaande waarvan je zegt, nou die zou je…

**R: ja, ik denk dan aan de ontwikkelingen op social media. Dat zijn allemaal dingen die je waarschijnlijk al tig keren gehoord hebt… Kijk, onze samenleving wordt steeds individueler, dus wellicht zijn er kinderen die het fijn vinden om met hun avatar, hun digitale persoon, dat vinden zij een veilige omgeving en ze sluiten zich van het werkelijk sociale gebeuren af. Gisteravond zag ik bij ‘man bijt hond’ over wat wordt nu de trend voor de toekomst? Die kinderen gingen mee naar de bingo in een bejaardentehuis en ze dachten eerst wat gaan we nu krijgen maar achteraf vonden ze het allemaal wel gezellig, die ouderwetse kneuterigheid. Vroeger ging je naar school voor een stuk contact. Vo-scholen zijn hormoongestuurde instellingen en ik denk dat met de ontwikkeling van social media er toe kan leiden dat kinderen, die dat minder hebben en meer op die digitale wereld zitten, dat die zich meer gaan terugtrekken en liever thuis blijven zitten achter die computer en met die avatar hun werkelijkheid in en dan zeggen ‘ik ben ziek’ want dan kan ik thuis misschien…., want er is thuis heel veel te doen. Die ouders zijn alle twee werken, je zet je computer aan en dan is het toch zalig..**

**Z: Ja, dat is wel zo en je hoort het ook aan de kinderen, je hoort die verhalen wel eens terug en dan vraag ik ‘was het nou gezellig?’, ‘nee, want er was niemand en ik kon tegen niemand spelen’, ja en dat komt omdat die allemaal op school zaten.**

Er zijn ook scholen die zeggen het is onze maatschappelijke verantwoordelijkheid om kinderen meer mee te geven dan een diploma, breder.

**R: Ja, dat vindt elke school.**

**Z: Ja, dat is ook een open deur.**

**R: En hoe is dat gerelateerd aan M@ZL?**

Dat je niet alleen rechten maar ook plichten hebt? Ter voorbereiding op goed burgerschap, meedoen?

**R: Ja, dat heeft zeker raakvlakken.**

Zijn er nog dingen die jullie me mee willen geven?

**R en Z: Nee, dit was het wel.**
