# Supplementary material for: Attitudes towards Addressing Medical Absenteeism of Students: A Qualitative Study among Principals and Special Education Needs Coordinators in Dutch Secondary Schools
Source: PLoS One. 2016 Feb 4;11(2):e0148427. doi: 10.1371/journal.pone.0148427 (PMC4742281; doi:10.1371/journal.pone.0148427)
Supplement: S2 File — (DOCX) [file pone.0148427.s002.docx]

**(vertaling van de) uitspraken behorend bij kwalitatief onderzoek M@ZL/(Translation of the) quotes “Attitudes towards addressing medical absenteeism of students: a qualitative study among principals and special education needs coordinators in Dutch secondary schools.”**

**Participants (Part.):**

**P** = Directeur (Principal)

**S** = Zorgcoördinator (SENCO)

| Part. | Dutch | Translation |
| --- | --- | --- |
| Reasons for paying attention to extensive medical absenteeism | | |
| Negative impact of absenteeism on the students | | |
| S2 | Kinderen die vaak ziek zijn missen in sociaal opzicht. Want ze maken een hoop in de klas niet mee. En ook qua stof missen ze. Sommige kinderen kunnen het allemaal maar met moeite bijbenen. Die hebben al problemen met het plannen van hun huiswerk, en als ze dan ook nog eens veel ziek zijn, dan wordt dat heel erg lastig. Docenten moeten daarmee dealen. Daarom heeft verzuim altijd veel gevolgen niet alleen voor de leerling maar ook voor de docenten. | *“Students who are often sick miss out on subject matter. Their performance declines. They also lose out on social aspects and miss a lot of things that happen in class. Some of our students already struggle to keep up and to structure their school work, and if they are often sick, it becomes even more difficult. Teachers have to deal with that. Therefore, absence always has significant consequences for both students and their teachers.” S_2a_* |
| P3 | Kijk, want als ze op school zitten, dan is er structuur, dan is er regelmaat, en dan bestaat de kans dat ze op een bepaald moment enigszins op een goede manier in de ‘ongeregelde maatschappij’ terechtkomen. Die kans is dan groter. Daarom moeten we uitval bestrijden. | See, if they are at school, there is structure, there is regularity; so there is a chance that at a certain point, they end up in the ‘disorderly society’ in a somewhat good way. The chances of this are bigger this way; that is why we have to fight students’ dropping out. |
| P7 | Als een kind thuis komt te zitten, een beetje gaat hangen en vervolgens toch buitenshuis vertier zoekt enzovoorts. Ja, dat is ‘kat op het spek binden’, daar komt alleen maar rottigheid van. En dan krijgen we dus alleen maar, steeds meer, ‘drop-outs’ in onze samenleving. Dus zorgen dat ze gewoon in een bepaald stramien blijven en dat stramien is gewoon ’s avonds, ’s morgens en laat in de middag thuis en de rest van de dag gewoon op school. Dat is de manier om leerlingen regelmaat bij te brengen. | If children end up staying at home they will look for entertainment outdoors. Well, that is ‘to set the fox to watch the geese’; it will only cause trouble. And this way, the number of drop-outs will only grow and grow. So, we make sure they stay in a particular framework, which is just being at home in the evening, in the morning, and late in the afternoon, and being at school for the remainder of the day. That is how to teach children regularity. |
| P3 | School is gewoon een heel groot onderdeel van de dagelijkse regelmaat die kinderen gewoon moeten krijgen. Als ze naar school gaan is er structuur en regelmaat. | School is an important part of children’s required daily routine. If they are at school, there is structure and routine. |
| To share their concerns with students’ parents | | |
| S2 | Nou, het zijn de ouders die hun kind ziekmelden, sowieso. Ik vind het ook belangrijk om de zorg te delen als het niet goed gaat met een kind. En als wij dat signaleren, vind ik het ook heel belangrijk dat de ouders daar een rol in hebben. | *“Well, it is the parents who report their child sick, in any case. I consider it important to share care with the parents when a child is not doing well.” S_2b_* |
| P4 | Ook voor als leerlingen uitvallen omdat ze verkeerde keuzes maken en op het verkeerde pad terecht komen, dan is dat ook voor de leerling en ouders een probleem, en niet alleen voor de school. | Also, if students drop out because they start making bad choices and going astray, then it is a problem for the students and their parents, and not only for the school. |
| S9 | Ouders geven heel vaak terug dat ze niet de baas zijn over hun kind. ‘Ik kan wel zeggen dat hij moet gaan, maar dat doen ze niet’. Dat is een belangrijke. Ouders hebben niet meer het gezag. Ook niet over ’s avonds naar bed gaan en over slaaptijden enzovoorts. Ouders zijn dan over puberkinderen niet de baas. Althans, die ik dan spreek. Die zeggen ‘ja, dat klopt, dat vinden wij ook, maar we krijgen het niet voor elkaar’. | Parents often respond that they are not their children’s bosses. ‘I can tell him to go, but he will not do it’. That is an important one. Parents do not have authority anymore. Neither when it is about going to bed and bed times etcetera. When it comes to that, parents don’t have authority over their children. At least the ones I speak with. They say ‘yes, that is right, we agree, but we cannot make it happen’. |
| P1 | …Nou, wat je heel vaak ziet is dat de thuissituatie dusdanig is, dat er geen controle is op de kinderen, of ze er nu wel of niet zijn. De ouders hebben hun handjes vol met zichzelf en weten maar amper, en dan zeg ik het heel zwart-wit, of de kinderen wel op tijd naar school gaan en of ze er wel of niet zijn… | … Well, you often see that a home situation is in such a state that there is no control over children, whether parents are at home or not. Those parents are so occupied with themselves that, and I put this very plainly, they hardly know whether their children go to school on time and whether they are there or not… |
| S7 | …Onze leerlingen hebben dat uit zichzelf niet zo. Zo van ‘nou ja, als er niets van gezegd wordt, dan eh…’. Dus daar moeten we achteraan. Dus heeft ziek-zijn en afwezig zijn voor de leerling, maar ook voor de school, altijd een lange nasleep… | Our students do nott naturally have that. Like ‘well, if nobody mentions it, well then…’. So we have to pick that up. Therefore, illness and absence always has quite some aftermath for both the student and the school. |
| P8 | Veelvuldig schoolverzuim heeft alles te maken met de situatie waarin een kind zich bevindt. De thuissituatie is daar voor mijn gevoel dominant in. De thuissituatie, de keuze of die al of niet ‘de straat als een nieuw thuis omarmt’. De stabiliteit die het kind kan hebben of ervaren in zijn eigen omgeving of op school. Het gaat om die drie gebieden. Thuis, op straat, school. En op straat noem ik dan ook ‘het gebied van de voetbalvereniging en dat soort zaken’. | *“Frequent medical absenteeism has everything to do with the situation a student finds himself in. I feel that the home situation is a dominant factor here.” P_8a_*  The home situation determines whether or not the student will ‘embrace the streets as his new home’. The stability a child can obtain or experience in his own environment or at school. There are those three areas. At home, on the streets, at school. And with ‘on the streets’ I also mean ‘the environment of the football club and those kinds of matters’. |
| S6 | Want de leerling kan niet lekker in zijn vel zitten, kan gepest worden | *“Because the student can feel bothered, be bullied.”S_6a_* |
| S7 | En ook leerlingen die bijvoorbeeld stelselmatig op maandagochtend ziek zijn hè, daar hebben we ook veel last van hier. Leerlingen die dus gewoon in het weekend, in het weekend zo de bloemetjes buiten zetten dat ze echt te gammel zijn om naar school te komen. | *“We also have a lot of trouble with students who are consistently sick on Monday morning; These are students who go out at weekends and party so hard that they are in no state to come to school.” S_7a_* |
| S3 | …Wij hebben leerlingen in huis, die sowieso al vatbaarder zijn voor structuurloosheid… | … We have students who are more susceptible to lack of structure in any way… |
| P3 | Ja, en de verantwoordelijkheid, wat je dus ziet in onze maatschappij is, dat ouders in toenemende mate de verantwoordelijkheid voor de ontwikkeling van hun kind naar school schuiven, en dat is een compleet foute ontwikkeling. We hebben in onze ambitie ook staan dat we steeds meer, we willen ouders als steun zijn en bij de school betrekken. Dus ouders zijn onlosmakelijk onderdeel, logisch natuurlijk, van de opvoeding van hun kind, en de school kan daarbij ondersteunen, maar de school heeft andere taken. | *“In our society, parents are increasingly pushing the responsibility for the students’ development onto school, and that is a completely wrong development. It is our ambition to increasingly support parents and involve them in the school. Parents are, logically, inextricably bound up with the development of their children, and the school can support that, but the school has different responsibilities.” P_3a_* |
| The wish to raise the threshold for reporting sick | | |
| S4 | Het is een glijdende schaal naar beneden, op het moment dat een kind merkt dat hij zich gemakkelijk kan ziek melden is de volgende keer de stap weer wat kleiner. Op die manier ga je toch heel langzaam maar zeker richting voortijdig schooluitval. | *“It is a slippery slope; when they realise they can easily report themselves sick, next time it will be a smaller step.” S_4a_*  That way, you are slowly moving towards premature dropping out. |
| S9 | …die kinderen die zo lang verzuimen, dat is geen ziekte ‘in de medische zin van ziekte’… Die noemen zich heel snel ‘niet goed’, en dan blijven ze thuis. | Those students who are absence for so long, that is no illness ‘in a medical sense’. They are very quick to say they do not ‘feel well’, and then they stay home. |
| P2 | Op het moment dat wij hier een arbeidsethos ontwikkelen door mensen zich minder gemakkelijk ziek te laten melden, zullen ze dat ook later in hun werk op een andere manier doen. | The moment we develop a work ethic by making it less easy for people to call in sick, they will also behave differently in their future jobs. |
| P4 | We hebben ook een maatschappelijke opdracht. Je leidt uiteindelijk toe naar goed burgerschap en als je straks in een werksituatie terecht komt en je hebt in het verleden alleen maar meegemaakt dat je je heel gemakkelijk ziek kunt melden dan heb je daar ook straks in de werksituatie last van. | We also have a welfare assignment. This will lead you towards good citizenship and if you later on end up in a work situation and you have only had the experience of easily calling in sick you will suffer from this in your work situation. |
| P4 | …Het draagt er aan bij in het veranderende normbesef in de hele samenleving dat het er toe doet, dat je rechten en plichten hebt, en als je leerplicht hebt dan moet je naar school en als je straks werkt dat je de verplichting hebt naar je werkgever om daar je stinkende best voor te doen. En als wij in onze samenleving alleen maar prinsjes en prinsesjes opvoeden, als dat straks de kurk is waar de hele samenleving het van moet hebben of dat dan wel goed gaat komen… | ….,  *“It contributes to the changing attitude to standards throughout society; that it matters, that you have rights and duties, and that if you are still of school age you have to go to school.” P_4a_*  and that if in the future you have a job, you have an obligation to your employer to work your very hardest. And if we only raise little princes and princesses in our society, they will be on whom the entire society will have to depend, and whether that will turn out well… |
| P9 | … omdat kinderen recht hebben op ontwikkeling en scholing voor hun toekomst… | Because children have a right to development and education for their futures. |
| P8 | …Op enig moment interpreteren wij gedrag van leerlingen, daar verbinden wij conclusies aan. En die conclusies, dat beeld, dat wordt doorgaans alleen maar steviger. Want wij noteren en wij zien vooral wat we willen zien. Wat past in dat eerder opgebouwde beeld. Noem het maar ‘de tunnelvisie’. En een kind waarvan op enig moment het beeld in ontstaan dat ze ‘faket’, dat wordt moeilijk om dat te doorbreken… | *“At any moment we interpret students’ behaviour; and we draw our conclusions. And these conclusions, this image, will only become stronger. Because we mostly see what we want to see – that fits the picture we have previously built up. You can call it ‘tunnel vision’. And, once an image has been formed that a student is ‘faking it’, it will be hard to break it.” P_8b_* |
| Experiences in applying MASS | | |
| Problems they experienced before | | |
| S2 | Ja, de ene mentor die is weer wat ‘vierkanter’ of ‘korter door de bocht’ dan de andere. En dat is met alle taken die je hebt natuurlijk. Dat is overal waar je werkt, de een neemt zijn taken heel erg serieus en gaat daar eens uitgebreid voor zitten. | *“Yes, one teacher might be ‘bolder’ or ‘more forward’ than another. And that is the case for all duties one has of course. It is like that at every work place; one person takes his duties very seriously and gets right down to it.” S_2c_* |
| P4 | …Ja, je bent elkaars bondgenoot, ouders, leerling en school trekken aan dezelfde kant van het touw, streven dezelfde dingen na, maar verlangen ook wel iets van het handelen om die rol ook goed samen te spelen en zolang je daar in de relatie met elkaar goed over kunt hebben, je hoeft het niet in alles met elkaar eens te zijn, maar dan houd je elkaar wel vast. Als je op enig moment als school tegenover de ouders en leerling komt te staan dan komen er uit die strijd over het algemeen alleen maar verliezers. Daar wordt niemand beter van. Dus het is ook van belang vind ik om duidelijk te zijn in wat je wilt en wat je verwacht, maar ook die relatie goed te houden omdat je beiden hetzelfde belang dient. Maar dat veronderstelt iets van de rol die je als school en ouder speelt. | Yes, you are each other’s allies, parents, student, and school are on the same side; they aim for the same things, but they also expect something from the act of playing that role well together, and as long as you can talk about this sufficiently in a relationship, you hold on to each other, and you do not have to agree on everything to do so. If at any time a school ends up opposing the parents and student, no one will win anything from it. No one will benefit from it. Therefore, I also consider it important to be clear on what you want and expect, but also to maintain the relationship because both serve the same interest. But that assumes something from the roles the school and parent play. |
| S4 | Als ik het eerste verzuimgesprek aan mentoren over moet laten, zonder mijn mentoren nou heel erg af te vallen, maar dan vraag ik me af in hoeverre die gesprekken goed gevoerd worden. Want je kunt een gesprek voeren op heel veel verschillende manieren en de een zal dat heel erg gemakkelijk afdoen en de ander zal daar toch…. | And I do not mean to let down my tutors, but  *“I doubt whether those conversations would be held properly. Because you can have a conversation in many different ways, and one person will deal with it more easily than another.” S_4b_* |
| P9 | Als een kind snottert, zeggen wij ‘je moet wel met het kind naar de dokter gaan, als het zo ernstig is dat hij nu al een week thuis is’. Gaan ze naar de dokter en die zegt ‘ja, kind snottert, blijft nog een weekje thuis en dan moet het wel gaan’. Dan voelen ouders zich al rap gesteund door zo’n huisarts. ‘Maar de huisarts zegt… Hij is ziek, want de huisarts vindt het goed’. Als je het verifieert zegt de huisarts leuk ‘daar hebben jullie niets mee te maken’. | If a student is sniffling, we say ‘we do have to take the child to a GP, if it is that serious for him to stay home for a week’. Then they visit the GP and he says ‘yes, he sniffles, keep him home for a week and then he will be OK’. Parents then feel supported by their GP. ‘But the GP said.. He is ill, because the GP has approved of it’. If you then verify this, the GP then blandly says ‘that is none of your business’. |
| S2 | Maar ik denk ook dat sommige mentoren dan heel bang zijn, van ‘nu moet ik een heel naar gesprek aangaan, want u als ouder meldt uw kind te gemakkelijk ziek.. of dat is het idee wat wij hebben’. Zo voelen ouders dat toch wel een beetje. Dus ik denk dat sommige mentoren daar ook een beetje bang voor zijn en ook een beetje vermijdingsgedrag gaan vertonen zelf (lacht). Van ‘nou, dit ga ik maar even uit de weg’. ‘Ik zal het wel even tussen neus en lippen door noemen, maar ik ga er maar niet te diep op in, want dan wordt het gesprek ook niet te moeilijk’. | *“I think that some teachers are scared at that moment, like ‘now I have to have a horrible conversation, because you as a parent report your child as sick too easily, or at least we feel that way’. So I think that some teachers then show avoidance behaviour themselves (laughs).”*  *“I’d better not deal with this now’. ‘I will just let it slip, and not go into it too much, that way the conversation will not become too difficult.” S_2d_* |
| S9 | Als je denkt ‘hier zou de jeugdarts eens naar moeten kijken, want dat kind, dat patroon, er klopt iets niet’. Dan vermoeden wij het als school wel eens, maar dan moeten de ouders naar de huisarts waar ze hun eigen verhaal moeten vertellen in hun eigen woorden. Soms geven we een brief mee als ouders dat willen waarin wij beschrijven wat we vinden. En dan moet je verwachten dat er een doorverwijzing komt, soms denkt een huisarts daar anders over, dat kan natuurlijk. | When you think ‘a youth health care physician ought to look at this, because that child, that pattern, something is not right’. Then we, as school, have our suspicions, but then parents have to visit the GP where they will have to tell their own story in their own words. Sometimes, if parents want us to, we give them a letter to take with them containing our opinion on the matter. And then you have to wait if there will be a reference; sometimes a GP thinks differently on this, this happens of course. |
| P2 | , maar omdat ik niet op de stoel van de dokter wil gaan zitten. Misschien zie ik iets met mijn beperkte medische kennis over het hoofd, wat die dokter niet over het hoofd ziet. | Because  *“I do not want to step into the physician’s shoes.*  *Perhaps, with my limited medical knowledge,  I would overlook something which a physician would not.”P_2a_* |
| S1 | Dan zou je het misschien niet doen, omdat je dan de confrontatie aan moet gaan. En dat is ook niet altijd leuk. Want mensen voelen zich zo snel aangevallen, terwijl het juist niet als aanval bedoeld is. | Maybe you would not do it then, because then you would have to confront. And that is not always pleasant. Because  *“People are so quick to go on the defensive,  even when no attack is intended.” S_1a_* |
| P8 | … We willen daar best iets mee doen, aan meewerken, maar wij zijn geen eigenaar van het probleem. Als leerlingen afhaken doordat ze andere keuzes maken, drugs gaan gebruiken of vinden dat ze perse met een mes naar school moeten komen, dan is dat een probleem van de leerling en de ouders. En niet van de school… | We are willing to work with that, to cooperate, but we are not the owners of the problem. If students drop out because they start making different choices, using drugs, or consider it necessary to show up at school with a knife, then it is the student’s and the parents’ problem, and not the school’s… |
| S7 | En via de ouders is de informatie toch al vaak heel erg gekleurd en niet erg betrouwbaar. | *“Information via the parents is often very biased and rather unreliable.” S_7b_* |
| P1 | Dan denk ik dat zij vinden dat er in hun privésfeer een beetje gerommeld wordt. En dat ze als ouder een beetje een ‘brevet van onvermogen’ krijgen. | “*I assume the parents think that their personal affairs are being interfered with, and that they are being accused of incompetence, as it were*.” *P_1a_* |
| S5 | Waarom moet ik naar de jeugdarts? Ik ga toch naar de huisarts, of ik heb een traject lopen bij het ziekenhuis. Dat is nog wel lastig om daarop te reageren, maar daar kan een jeugdarts wel een meerwaarde hebben. | Why do I have to go to the youth health care physician? I already go to the GP, or I am involved in a procedure at the hospital. It can be tricky to react to that, but there a youth health care physician can have a surplus value. |
| S9 | Nou, als ik de huisarts bel, krijg ik nooit een antwoord. Dat is terecht ook, denk ik. Maar daar zijn we als school geen partij voor. | *“Well, if I call the GP, I never receive an answer. And that is how it ought to be, I think. But that is not for us to do, as a school.” S_9a_* |
| S7 | …, want van een huisarts worden we meestal niet wijzer, als we er al informatie van krijgen… | Because  *“We are seldom any wiser after speaking to a GP,  if we get any information at all.” S_7c_* |
| Advantages of MASS: The approach in general | | |
| S2 | En het feit dat ik nu heel erg makkelijk aan ouders kan uitleggen ‘het geldt voor iedereen’, ‘iedereen moet dit gewoon en wij zijn geen arts’. | And the fact that  *“It is now very easy for me to explain to parents ‘this applies to everyone’, ‘everyone just has to do this and we are not doctors.” S_2e_* |
| P2 | Vóór de start van het project was het moeilijk aan ouders uit te leggen als je acties wilde ondernemen ivm het ziekteverzuim. | Before the start of the project it was difficult to explain to parents that you wanted to undertake actions regarding medical absenteeism. |
| S6 | Want dat gesprek met die ouders, als het gelijk naar de jeugdarts zou gaan, dan zien de ouders niet de link met school, dat school dat.. dat school ze in de gaten heeft, dat er een gesprek komt. Dan kun je ook zeggen het is een gesprek uit zorg. Daar komen ook nog ook andere dingen uit. Want de leerling kan niet lekker in zijn vel zitten, kan gepest worden, dat een kind eh.. en als deze problemen blijken dan kan de docent er zelf mee aan de slag. | Because the conversation with those parents,  *“If parents were to go to the YHCP straightaway, they would miss the connection with the school, that the school cares*,  that a conversation will take place. You can also put it that way:  *“It is a conversation based on concerns. Other matters will arise from it. Because the student can feel bothered, be bullied, a student can eh…, and if these matters turn out to be the cause of the absenteeism, teachers can take them up themselves.” S_6b_* |
| P2 | Ik ben ervan overtuigd dat een leerling die in een gezinssituatie zit waar bijvoorbeeld ouders gaan scheiden of er sprake is van een overlijden van een van de ouders, klachten kan ontwikkelen, die we met M@ZL vroeg ontdekken. | *“I am convinced that a student in a family situation where parents are getting divorced or where one of the parents has passed away can suffer from the situation and develop symptoms that we can identify with MASS at an early stage.” P_2b_* |
| S1 | ….dat zou er ook weer aan mee kunnen helpen dat een kind nog meer gaat verzuimen, want op het moment dat het kind dan op school is, wordt hij door tien docenten ineens bij z’n kladden gegrepen van ‘jij moet dit en dit nog inhalen en inleveren’ en dat kind ziet dan ook niet meer hoe hij dat voor elkaar moet krijgen. De stress wordt dan misschien zo hoog dat ze daardoor weer gaan verzuimen. | And that could cause for a student to be absent even more, because  *“Whenever the student went to school, there would be ten teachers grabbing him to tell him to ‘catch up with this and hand in that’, and the student wouldn’t be able to figure out how to manage everything. The stress might then become too much, resulting in more absence.” S_1b_* |
| Advantages of MASS: The collaboration with YHCPs | | |
| P2 | Als wij dat zeggen, zijn we volgens de ouders ‘de tegenpartij’ die een ander belang heeft. Wij moeten die kinderen binnen school houden. Op het moment dat een onafhankelijke derde, die ook nog arts is, heeft gestudeerd en een bepaalde status heeft bij ouders, er iets over zegt, komt dat anders binnen dan wanneer je dat als onderwijsinstelling doet. | And if we say that, then parents consider us the ‘opposition’ who have a different interest in it. We need to keep those students inside school.  *“If an independent third party, a qualified doctor, with a certain status in the eyes of the parents’, gives his opinion, this is received differently from when an educational institution does so.” P_2c_* |
| S2 | En dat de jeugdarts daar toch op een andere manier gesprekken over heeft dan een huisarts. | And that a youth health care physician has different conversations on those subjects than a GP. |
| P9 | En door M@ZL kunnen we de leerling doorsturen voor verdere behandeling, waardoor zo’n kind niet in psychische nood komt. Deze leerling krijgt van ons wel ondersteuning, maar niet de medische ondersteuning die hij misschien nodig heeft. | *“And because of MASS we shall be able to refer a student for more care, ensuring that the student will not end up in mental distress. School can provide this student with support, but not the medical support he might need.” P_9a_* |
| S5 | Nou, kijk, in mijn ogen en dit bedoel ik niet zo negatief als dat het overkomt.. Maar onderwijsmensen zijn ‘eigenheimers’ en die denken dat ze overal verstand van hebben. En ‘wie gaat mij vertellen wat ik met die leerling moet doen’. En als er nu op het medische of sociaal-emotionele vlak vanuit een andere hoek en vanuit een ander perspectief adviezen of tips komen, dan vind ik dat heel erg waardevol. En ook voor mensen uit het onderwijs, die op dat vlak.. Ik denk dat ik er zelf ook op het vlak van het sociaal-emotioneel benaderen van kinderen een heleboel van geleerd heb. Van de mensen die hier van buiten het onderwijs de school binnengekomen zijn. Daarom zeg ‘samen ben je sterker dan alleen’. En het onderwijs kijkt met een bepaalde blik en vanuit een bepaald perspectief naar leerlingen. Maar een leerling is meer dan alleen een vat waar een hoop kennis in gepompt moet worden, daar zitten ook wat andere aspecten aan. En die mogen wij niet uit het oog verliezen.  Daarom vind ik het belangrijk om de zorg de school binnen te trekken. Dat denk ik. En ik zie daar gewoon heel duidelijk de meerwaarde van. En dat voel ik ook iedere dag. | Well, as I see it, and I do not mean this as negative as it may come across… but people in education are introverts and they think they know about everything. And ‘who is going to tell me what to do with that student’. And if advices on medical of social-emotional level were to come from a different perspective, I would consider that highly valuable. And also for people working in education, who on that level… I think I have learned a lot from it myself when it comes to the social-emotional approach of students. From those people here who have entered school from areas outside education. That is why one says ‘united we stand strong’. And education has a certain view on students and regards them from a certain perspective.  But a student is more than just a barrel needing to be filled with knowledge; there are other aspects as well. And we ought to keep those in sight.  That is why I regard it important to include care in the school. That is how I see it. And I can see the surplus value of it very clearly. And I feel it every day. |
| S5 | En het gaat ook niet om het corrigeren van ouders, daar gaat het niet om. Maar gewoon dat iemand met een medische achtergrond gewoon bevestigt dat datgene wat er gebeurt is, dat het goed is zo. Daar gaat het om. Maar ook als iemand met een medische achtergrond vaststelt dat daar toch wel onterecht lang thuis gehouden is. Van ‘uw kind heeft wel in het gips gezeten, maar die had met een rolstoel misschien best wel een les kunnen bezoeken, waarom heeft u hem zo lang thuis gehouden?’. Dat soort gesprekken hè. Dan gaat het niet eens om verwijten, maar wel om die mensen op dat moment op scherp te zetten. | And it is not about correcting parents, that is not what it is about. But ….  *“It is just the fact that somebody with a medical background confirms that what is happening is correct. That is essential. But it also helps when somebody with a medical background ascertains that a student has been kept home for a long time unjustly and asks the parents: ’Why did you keep him home that long?’  It is not even about blame, but it is a matter of making people aware of the situation.” S_5b_* |
| S5 | En een jeugdarts bespreekt dit soort zaken toch anders dan een huisarts. | *“A YHCP discusses these subjects differently from a GP.” S_5c_* |
| S9 | Als je denkt ‘hier zou de jeugdarts eens naar moeten kijken, want dat kind, dat patroon, er klopt iets niet’. Dan vermoeden wij het als school wel eens, maar dan moeten de ouders naar de huisarts waar ze hun eigen verhaal moeten vertellen in hun eigen woorden. Soms geven we een brief mee, als ouders dat willen, waarin wij beschrijven wat we vinden. En dan moet je afwachten of er een doorverwijzing komt, soms denkt een huisarts daar anders over, dat kan natuurlijk. Maar als zo’n jeugdarts ook al denkt van ‘ja, je moet toch eens kijken naar hulpverlening thuis als jullie het niet voor elkaar krijgen’ en de school zegt dat ook.. Dan kun je misschien toch wat meer druk uitoefenen. | If you think ‘a youth health care physician should have a look at this, because that student, that pattern, something is not right’. Then we, as school, have our suspicions, but then parents have to visit the GP where they will have to tell their own story in their own words. Sometimes, if parents want us to, we give them a letter to take with them containing our opinion on the matter. And then you have to wait if there will be a reference; sometimes a GP thinks differently on this, this happens of course. But if a youth health care physician also thinks ‘well, you will have to consider assistance if you cannot manage it yourselves at home’ and the school says the same… Then maybe you can apply more pressure. |
| S3 | Dat wordt meestal wel, met de terugkoppeling die we krijgen van de jeugdarts, dan krijg je wat handvatten, bv van een leerling met buikpijn: als ze buikpijn heeft mag ze de eerste 2 uren thuis blijven maar vanaf het 3de uur is ze dan op school. En dan is het niet meer de gewoonte, zoals het was, de hele dag niet naar school. En dat zijn afspraken die je in overleg met ouders en leerling als jeugdarts maakt. | It often happens,  *“The feedback received from the YHCP gives you something to get hold of.” S_3a_*  *, f*or example a student with stomach aches: if she has stomach aches she can stay home the first 2 hours, but she will attend school from the third hour on. And there is no longer the habit, like it was before, of not attending school all day. And those are the rules a youth health care physician sets by mutual agreement with parents and student. |
| P7 | En ik maak een hele lange route om aan te geven dat het eigenlijk het proactieve en preventieve werk, op dit moment helemaal niet uit de voeten komt. Vandaar dat ik ook eigenlijk aardig gecharmeerd was van het M@ZL project, omdat dat min of meer een beetje op die route gaat staan. Bij signalen die nog helemaal niet uit de hand zijn gelopen, dus vroegsignalering van problemen, kun je op individueel niveau aan de slag. En dan kun je effect bereiken. Nou, daar zit spanning op. | And I take a very long route to indicate that at this moment, the proactive and preventive work actually cannot be achieved. That is why I quite liked the MASS project; because it basically takes the same direction. With signals that are not yet completely out of control, when problems are signalled in an early stage, steps can be taken on an individual basis. And then effects can be achieved. Well, that is tensed. |
| S5 | Dus ja, de lijntjes zijn kort. En het wordt ook aan elkaar gekoppeld ja. Dat is wel een verschil met voorheen. En dat vind ik ook wel sterk. | *“Since MASS, the connections are short and are really being made. That is a substantial difference with how it used to be.” S_5d_* |
| Advantages of MASS: a better understanding | | |
| P7 | Ik denk verschillende dingen. Ten eerste denk ik dat er veel meer begrip zou komen, niet alleen emotioneel begrip, maar letterlijk begrip, over situaties. Kennis over wat er speelt bij kinderen en wat redenen kunnen zijn om op school te verzuimen. Het effect daarvan zou moeten zijn dat we daar adequater mee om kunnen gaan en het uiteindelijk effect zou meetbaar kunnen zijn. | I think different things.  *“Firstly, I think there could be much more understanding, not just at an emotional level, but literal understanding, about situations. Knowledge about what goes on in students’ lives and what reasons there can be for not attending school. Students and teachers can learn how to deal with absenteeism more satisfactorily and, eventually, it decreases.” P_7a_* |
| P3 | … maar met name omdat je samen met ouders, school en leerling, moet zorgen voor een veilige en goed mogelijke schoolloopbaan voor leerlingen. Daar is het verzuim, en de situatie thuis en op school onderdeel van. Dus door het ziekteverzuim goed te monitoren, goed terug te koppelen, ook naar ouders, heb je volgens mij een beter beeld van het welbevinden van het kind, zowel op school als thuis, en vervolgens komt dat leerprestaties en in ieder geval de schoolontwikkeling ten goede. | But mostly because, together with parents, school and student, you have to provide a safe and probable school career for students.  *“Absenteeism, home situation, and school situation are all aspects of a school career. By monitoring medical absenteeism, giving good feedback, also to parents, I think you gain a better perspective of the well-being of a student, at school as well as at home, from which study results and definitely school development subsequently benefit.” P_3b_* |
| S3 | Het is wel gaan leven binnen de school. | It has become more important within the school. |
| Facilitators and obstacles when implementing MASS, including consideration of sustainability | | |
| Dialogue about medical absenteeism | | |
| S2 | Hè, dat denk ik. En dan worden ze boos. En zeggen ze ‘nee, ik weet heel goed, mijn kind is te ziek om naar school te gaan en daar hebben jullie niets mee te maken. Als ik zeg dat hij ziek is, dan is hij ziek.’ En dan moet je ook wel veel in huis hebben om door die weerstand heen te kunnen breken. Ik kan me voorstellen dat dat voor mentoren, die vinden dat toch moeilijk. Het bespreken van ziekteverzuim roept weerstand op bij ouders. Dat maakt het moeilijk. | Because they do not think it necessary, ‘I will decide whether my child is fit to go to school or not’. ‘I know very well that my child is too ill to attend school, and that is none of your business. If I say he is ill, he is ill’. And then you will have to stand your ground very firmly in order to break through such resistance. I can imagine this being difficult for mentors.  *“Discussing sickness absence often provokes resistance from parents. That makes it hard.” S_2f_* |
| S3 | En ik denk dat het met ouders praten over ziekteverzuim, dat valt te leren. | *“I think that talking to parents about medical absenteeism can be learned.”S_3b_* |
| Follow-up to the feedback | | |
| P7 | Nadeel zou zijn dat wij die expertise, waar ik het daar straks in het verhaal over had, bij mijn eigen mensen, niet in de school zou krijgen. Dus het verzuimcontact, dat kun je leren. Ik zie dat docenten het moeilijk vinden het advies van de jeugdarts te bespreken en uit te voeren. | *“The downside of the first route would be that our school would not develop its own expertise and learn to handle contacts about absenteeism. I see that teachers find it difficult to discuss and perform the advice of the YHCP.” P_7b_* |
| P1 | Dat moet natuurlijk sluitend zijn. Zeker op het eind van het traject. Als de leerplichtambtenaar een keer met de cijfers komt en het zou zo ver komen dat het bij de rechter komt en je gegevens zijn niet goed, dan zegt zo’n rechter ‘hup, wegwezen, zorg eerst dat je je huiswerk goed hebt gedaan’ en dan kijken we weer verder. Als je maar even kan weerleggen dat zo’n leerling wel op school is geweest, dan voelen leerling en ouders zich overwinnaar. En denken ze ‘zie je nu wel, niets aan de hand’. Dan is het klaar met de maatregel. Nu is het niet de doelstelling om de leerlingen voor de rechter te brengen, dat is niet wat ik zeg, maar het moet gewoon kloppen. | *“Absenteeism registration has to be accurate, throughout the time at school. Otherwise, if the student is referred to the school attendance officer and the case goes to court, a judge can say ‘Get lost, do your homework first and then we shall see.’ If you can't refute the contention that a student has attended school, some students and parents will feel victorious.” P_1b_*  And they will think ‘see, it is all fine’. And then the measure is expired. Of course, the aim is not to have the students appear in court, that is not what I am saying, but it has to be accurate. |
| S5 | En als je dat inderdaad gewoon vanaf het begin heel duidelijk communiceert met ouders en zij het dan ook zien als een extra hulp en niet als een of ander boosaardig controlemiddel, dan voorkom je ook - in de tijd dat het zich voordoet - allerlei boze telefoontjes van ‘wat zijn jullie nu met mijn kind aan het doen’. Want dat is dan al gecommuniceerd. | And indeed,  *“If the communication with parents is very clear from the beginning, and they see it as an extra aid and not as an evil means of control.”*  *S_5a_*  *, you avoid angry phone calls from parents saying ‘What are you doing with my child?.” S_5e_*  Because then that has already been communicated to the parents. |
| To achieve the full support | | |
| P2 | Wij denken ook wel eens dat het thuis niet goed gaat met een kind, we krijgen wel eens signalen die helemaal mis zijn bij kinderen thuis. Op het moment dat we die informatie doorspelen, zijn dat geen feitelijkheden, maar zijn dat veronderstellingen. Op het moment dat veronderstellingen door de jeugdarts dan ook naar ouders worden doorgegeven als zijnde ‘veronderstellingen van de school’, dan speelt een ouder ons tegen elkaar uit. Dat zijn dingen die we goed moeten doen met elkaar. | Sometimes, we also think that a student’s home situation is troubled; now and then we receive signals that the situation at home is objectionable. The moment we pass on that information those are not facts, those are assumptions. The moment assumptions are passed on to parents by a youth health care physician as if it were ‘the school’s assumptions’,  *“Parents are playing us off against each other. Those are matters we have to deal with together.” P_2d_* |
| S9 | Het strandt vaak na het stukje jeugdarts vind ik, dan komen wij in het moeras. Dat stukje is nog helder en dan… En als de ouders niet komen ook, ja, wat dan. Als ze zeggen ‘ik ga er niet naartoe’. Dan kom je in het ‘drijfzand’. | After the part of the youth health care physician it often fails, I believe. That piece is still clear, but after that… And if parents do not show up either, well, then what? If they say ‘I am not going’. You end up in the ‘quicksand’. |
| P3 | En het is eigenlijk niet acceptabel dat de ene mentor wel in gesprek gaat bij ziekteverzuim en de ander niet. | It is not acceptable that one mentor discusses the absenteeism  and the other does not. |
| P2 | En vervolgens moeten alle mentoren wel aan de slag met die terugkoppeling van de jeugdarts. | All teachers need to pick up on the feedback. |
| The sustainability of MASS | | |
| S1 | We moeten het gevoel hebben dat het wat oplevert. | *“We have to feel that it brings results.” S_1c_* |
| P2 | Het aantal ziekmeldingen wat wij krijgen, is door M@ZL in kaart gebracht en dat heeft onze ogen wel geopend. Er zaten daar wel ‘schellen’ voor, want het was enorm! Daar zijn wij zo van geschrokken, dat je er nadrukkelijker naar aan het kijken bent. | *“The number of sickness reports we receive has been mapped by MASS, and that has opened our eyes. We had been blind before, because the number was huge! We were so alarmed by it that we now treat it with greater emphasis.” P_2e_* |
| S5 | Maar het effect zit hem misschien ook wel gewoon in het feit dat ook ouders in de gaten krijgen hoe vaak ze hun kind ziek melden en van ‘ik kan mijn kind..’. Er zijn ouders die laten hun kinderen thuis ‘als het bij het oorlelletje zeer doet’. En daar kunnen wij als school iets van zeggen, maar nu zegt er ook een keer een jeugdarts iets van. Ouders hebben dan in de gaten dat ze hun kind niet zomaar ongestraft thuis kunnen laten. Want daar worden ze op aangesproken, niet alleen door de school, maar ook door de jeugdarts. En die jeugdarts gaat ook nog zeggen van ‘als ik dit blijf doen, dan gaat de leerplicht in beeld komen’. Daar gaat een preventieve werking van uit, denk ik. | But  ..*“The effect of it might be in the fact that the parents now also become aware of the frequency with which they report their children sick.” S_5f_*  and use ‘I can report my child..’. There are parents who report their children ill ‘if their ear lobes are hurting’. And we as a school can comment on that, but now a youth health care physician can comment on it too. Parents will then realize that they cannot keep their children from school unnoticed. Because they will be called to account for it, and not only by the school, but also by the youth health care physician. And the physician will tell them that ‘if I keep doing this, compulsory education will come into the picture’. I think a preventive effect comes from that. |
| P4 | En ik ben ervan overtuigd dat het ziekteverzuim af neemt. | *“ I am convinced that absenteeism has decreased since the application of MASS.” P_4b_* |
| P8 | Want als het gaat om vroegtijdig schoolverlaten, daar spelen zoveel factoren een rol. | “*Because when it comes to premature dropping out, there are so many factors of influence. P_8c_* |
| P3 | En dat zou voor de gemeenten geweldig zijn. Dat zijn de mensen die later ook uitvallen. En dan gaat het geld kosten, noem maar op. We hebben het dan nog niet eens over het psychisch welbevinden van de mensen. | And that would be great for local authorities. Those are the people who also drop out in the future. And then it will cost money, you name it. We are not even talking about people’s mental well-being yet. |
| P2 | Ik heb er geen bezwaar tegen om ook mee te betalen, maar we zitten natuurlijk allemaal in zwaar weer. | I have no objections against helping to pay for it, but all of us have our problems. |
| P5 | De gemeente heeft een bepaalde ambitie en bij die ambitie hoort een stukje ondersteuning. Als de gemeente die ondersteuning weglaat, moeten ze haar ambitie bijstellen. Het is een kwestie van ‘nu wat besparen en dat straks 10 keer uitgeven’. Aan kinderen die toch onderweg verloren zijn. Kinderen die voor overlast zorgen buiten het onderwijs, kinderen en gezinnen die met een stuk bemoeizorg op latere leeftijd worden geconfronteerd, aan justitiële instellingen. Ik denk dat alles wat je preventief kunt doen, vele malen goedkoper is dan wat je later moet repareren. De gemeente zou dus ook in M@ZL moeten willen investeren. | The local authorities have a certain ambition and this contains a bit of support. If the local authorities were to leave out that support, they will have to adjust their ambition. It is a matter of ‘saving a bit now and spending it times ten in the future’. On children whom we have lost along the way. Children causing nuisances outside education, children and families who are confronted with interference at a later age, by judicial institutions. I think that everything that can be done preventively is many times cheaper than what needs to be fixed in a later stage. Therefore, the local authorities ought to want to invest in MASS. |
| P3 | Misschien draagt M@ZL wel in een heel sterke mate bij tot het verlagen van het vsv, of minder ziek meldingen op het werk, maar dat weet je pas over een aantal jaren. | *“Perhaps MASS contributes greatly to decreasing dropouts, or less sickness reports at work, but you will find out only in a couple of years’ time.” P_3c_* |
| P5 | Uiteindelijk worden beoordeeld op het aantal geslaagde leerlingen. | *“Eventually, we are judged on graduation rates.” P_5a_* |
| P2 | Waarom moet de gemeente dat? Omdat als wij hier niets doen, zitten zij straks met de problematiek van mensen die uitvallen in het onderwijs. | Why do the local authorities have to? Because if we sit here and do nothing, they will be burdened with the issues of people dropping out of education. |
| P6 | Over die verantwoordelijkheid, aandacht voor ziekteverzuim staat niet op onze agenda. Maar ik er zo over nadenk, het vergroten van de ouderbetrokkenheid bij de school staat wel op de agenda en heeft een hoge prioriteit.  Op dit ogenblik is de ouderbetrokkenheid ook een van de 10 punten waar wij ons druk over maken. | *“Paying attention to medical absenteeism is not on our agenda. However, now that I think about it, increasing parental involvement in school is on schools’ agendas and has a high priority.”P_6a_*  At this moment, parental involvement is one of the 10 points we are concerned with. |
| P3 | De invloed van M@ZL is daar niet direct aan afleidbaar, maar het draagt er wel toe bij. | “*And I see that MASS can help us to achieve this.” P_3d_* |
